# Author Correction: Prospective individual patient data meta-analysis of two randomized trials on convalescent plasma for COVID-19 outpatients

**DOI:** 10.1038/s41467-024-48645-y

**Published:** 2024-05-22

**Authors:** Pere Millat-Martinez, Arvind Gharbharan, Andrea Alemany, Casper Rokx, Corine Geurtsvankessel, Grigorios Papageorgiou, Nan van Geloven, Carlijn Jordans, Geert Groeneveld, Francis Swaneveld, Ellen van der Schoot, Marc Corbacho-Monné, Dan Ouchi, Francini Piccolo Ferreira, Pierre Malchair, Sebastian Videla, Vanesa García García, Anna Ruiz-Comellas, Anna Ramírez-Morros, Joana Rodriguez Codina, Rosa Amado Simon, Joan-Ramon Grifols, Julian Blanco, Ignacio Blanco, Jordi Ara, Quique Bassat, Bonaventura Clotet, Bàrbara Baro, Andrea Troxel, Jaap Jan Zwaginga, Oriol Mitjà, Bart J. A. Rijnders, CoV-Early study group, Arvind Gharbharan, Casper Rokx, Carlijn Jordans, Corine Geurtsvankessel, Grigorios Papageourgiou, Bart Rijnders, Peter Katsikis, Yvonne Müller, Marion Koopmans, Susanne Bogers, Jelle Miedema, Henk Russcher, Cees Scherpenisse, Rene van Engen, Ayten Karisli, Hannelore Götz, Jelle Struik, Lotte Rokx-Niemantsverdriet, Nan van Geloven, Geert Groeneveld, Jaap Jan Zwaginga, Lisa Zwaginga, Josine Oud, Romy Meier, Erik van Zwet, Simon Mooijaart, Arjan Albersen, Francis Swaneveld, Ellen van der Schoot, Hans Vrielink, Leo van de Watering, Boris Hogema, Peter van Wijngaarden, Ronald van Etten, Adriaan van Gammeren, Nanda Maas, Betty van Ginneken, Jan den Hollander, Jose Verstijnen, Juliette van den Berg – Rahman, Faiz Karim, Siepke Hiddema, Kim van Elst, Elena van Leeuwen-Segarceanu, Annette Reitsma, Karin Molenkamp, Robert Soetekouw, Caterina Band, José de Droog, Jolanda Lammers, Lonneke Buitenhuis, Douwe Postma, David Koster, Michaèl Lukens, Thea Scholtens, Maartje van den Boomgaard, Machiel Vonk, Linda Kampschreur, Marit van Vonderen, Loes Vrolijk, Chantal Reusken, Johan Reimerink, Heli Harvala, COnV-ert study group, Andrea Alemany, Marc Corbacho-Monné, Dan Ouchi, Bonaventura Clotet, Oriol Mitjà, Gèlia Costes, Mar Capdevila-Jáuregui, Pamela Torrano-Soler, Alba San José, Zahida Jiménez, Ferran Ramírez-Viaplana, Susana Ferrer, Mireia Gallardo, Maria Ubals, Camila González-Beiras, Martí Vall-Mayans, Miquel Angel Rodriguez-Arias, Clara Suñer, Jordi Puig, Aroa Nieto, Ivan Galvan-Femenia, Xavier Comas-Leon, Pere Millat-Martínez, Quique Bassat, Bàrbara Baro, Ignacio Blanco, Jordi Ara, Glòria Bonet Papell, Maria Delgado Capel, Beatriz Díez Sánchez, Maria Pons Barber, Cristian Gonzalez Ruiz, Laura Navarrete Gonzalez, David González García, Ainhoa Vivero Larraza, Victor Carceles Peiró, Clàudia Roquer López, Magí Ferrer, Pierre Malchair, Sebastian Videla, Vanesa García García, Carlota Gudiol, Aurema Otero, Jose Carlos Ruibal Suarez, Alvaro Zarauza Pellejero, Ferran Llopis Roca, Orlando Rodriguez Cortez, Pablo Casares Gonzalez, Gemma Arcos Vila, Begoña Flores Aguilera, Graciela Rodríguez-Sevilla, Macarena Dastis Arias, Anna Ruiz-Comellas, Anna Ramírez-Morros, Judit Roca Font, Katherine M. Carrasco Matos, Glòria Saüch Valmaña, Carla Vidal Obradors, Joana Rodríguez Codina, Rosa Amado Simon, Silvia Tarres García, Margarida Curriu Sabatès, Raquel Nieto Rodríguez, Joan-Ramon Grífols, Anna Millan, Enric Contreras, Àgueda Ancochea, Rosa Línio, Miriam Fornos, Natàlia Casamitjana, Eva Alonso, Núria Martinez, Laura Analía Maglio, Laura Comellas Fernandez, Nadia Garcia, Luis Hernández, María Isabel González, Anna Bravo, Yolanda García, Núria Prat, Joaquim Verdaguer, Thatiana Vértiz Guidotti, Sergio Benavent, Andrea Sofia Bianco, Ney Nicanor Briones Zambrano, Maria Viozquez Meya, Anna Forcada, Josep Vidal-Alaball, Montserrat Giménez, Alexa París, Gema Fernández Rivas, Cristina Casañ Lopez, Águeda Hernández, Antoni E. Bordoy, Victoria González Soler, Julian Blanco, Edwars Pradenas, Silvia Marfil, Benjamin Trinité, Francini Piccolo Ferreira, Mireia Bonet, Jordi Cantoni, Michael Marks

**Affiliations:** 1 Fight AIDS and Infectious Diseases Foundation, Badalona, Spain; 2 ISGlobal, Hospital Clínic, Universitat de Barcelona, Barcelona, Spain; 3 Department of Internal Medicine, Section of Infectious Diseases and department of Medical Microbiology and Infectious Diseases, Erasmus MC, University Medical Center, Rotterdam, The Netherlands; 4 Hospital Universitari Germans Trias i Pujol, Badalona, Spain; 5 Facultat de Medicina-Universitat de Barcelona, Barcelona, Spain; 6 Department of Viroscience, Erasmus MC, Rotterdam, The Netherlands; 7 Department of Biostatistics, Erasmus MC, University Medical Center, Rotterdam, The Netherlands; 8 Department of Biomedical Data Sciences, Section of Medical Statistics, Leiden University Medical Center, Leiden, The Netherlands; 9 Department of Infectious Diseases and Acute Internal Medicine, Leiden University Medical Center, Leiden, The Netherlands; 10 Unit of Transfusion Medicine, Sanquin Blood Supply, Amsterdam, The Netherlands; 11 Department of Experimental Immunohematology, Sanquin Research, Amsterdam, The Netherlands; 12 Hospital Universitari Parc Taulí I3PT, Sabadell, Spain; 13 Universitat Autònoma de Barcelona, Barcelona, Spain; 14 Bioclever-CRO, Barcelona, Spain; 15 Emergency Department, Bellvitge University Hospital, L’Hospitalet de LLobregat, Barcelona, Spain; 16 Clinical Research Support Unit (HUB-IDIBELL: Bellvitge University Hospital & Bellvitge Biomedical Research Institute), Bellvitge University Hospital, L’Hospitalet de Llobregat, Barcelona, Spain; 17 Pharmacology Unit, Department of Pathology and Experimental Therapeutics, School of Medicine and 33 Health Sciences, IDIBELL, University of Barcelona, L’Hospitalet de Llobregat, Barcelona, Spain; 18 Unitat de Suport a la Recerca de la Catalunya Central, Fundació Institut Universitari per a la recerca a l’Atenció Primària de Salut Jordi Gol i Gurina, Sant Fruitós de Bages, Spain; 19 Health Promotion in Rural Areas Research Group, Gerència Territorial de la Catalunya Central, Institut Català de la Salut, Sant Fruitós de Bages, Spain; 20 Universitat de Vic—Universitat Central de Catalunya (UVIC-UCC), Vic, Spain; 21 Salut Catalunya Central, Hospital de Berga, Berga, Spain; 22 Blood Bank Department—Banc de Sang i Teixits (BST), Barcelona, Spain; 23 IrsiCaixa AIDS Research Institute, Germans Trias i Pujol Research Institute (IGTP), Badalona, Spain; 24 Metropolitana Nord Laboratory, Institut Català de la Salut, Badalona, Spain; 25 Gerència Territorial Metropolitana Nord, Institut Català de la Salut, Barcelona, Spain; 26 Centro de Investigação em Saúde de Manhiça (CISM), Maputo, Mozambique; 27 Pg. Lluís Companys 23, ICREA, Barcelona, Spain; 28 Pediatrics Department, Hospital Sant Joan de Déu, Universitat de Barcelona, Esplugues, Spain; 29 Consorcio de Investigación Biomédica en Red de Epidemiología y Salud Pública (CIBERESP), Madrid, Spain; 30 Department of Population Health, NYU Grossman School of Medicine, New York, NY USA; 31 Department of Haematology, Leiden University Medical Centre, Leiden, The Netherlands; 32 CCTR, Sanquin Blood Supply, Amsterdam, The Netherlands; 33 Lihir Medical Centre—InternationalSOS, Lihir Island, Papua New Guinea; 34 Erasmus MC, Rotterdam, The Netherlands; 35 GGD Rotterdam, Rotterdam, The Netherlands; 36 Gezondheidscentrum Mathenesserlaan, Rotterdam, The Netherlands; 37 Leids Universitair Medisch Centrum, Leiden, The Netherlands; 38 Sanquin Blood Supply, Amsterdam, The Netherlands; 39 Amphia Hospital, Breda, The Netherlands; 40 Maasstad ziekenhuis, Rotterdam, The Netherlands; 41 Groene hart hospital, Gouda, The Netherlands; 42 St Antonius hospital, Nieuwegein, The Netherlands; 43 Spaarne Gasthuis, Hoofddorp, The Netherlands; 44 Isala Hospital, Zwolle, The Netherlands; 45 Universitair medisch centrum Groningen, Groningen, The Netherlands; 46 Medical Center Leeuwarden, Leeuwarden, The Netherlands; 47 Centrum voor Infectieziektebestrijding, RIVM, Bilthoven, The Netherlands; 48 NHS Blood and Transplant, London, UK; 49 Barcelona Institute for Global Health, ISGlobal, Barcelona, Spain; 50 Hospital Universitari Germans Trias i Pujol (HUGTiP), Badalona, Spain; 51 Hospital Universitari de Bellvitge, L’Hospitalet de Llobregat, Barcelona, Spain; 52 CUAP Manresa, Manresa, Spain; 53 Hospital Comarcal de Sant Bernabé, Berga, Spain; 54 Gerència Territorial Metropolitana Nord, Badalona, Spain; 55 Gerència Territorial Catalunya Central, Sant Fruitós de Bages, Spain; 56 Metropolitana Nord Laboratory, Badalona, Spain; 57 IrsiCaixa AIDS Research Institute, Badalona, Spain; 58 London School of Hygiene and Tropical Medicine, London, UK

**Keywords:** Viral infection, Randomized controlled trials, SARS-CoV-2

Correction to: *Nature Communications* 10.1038/s41467-022-29911-3, published online 11 May 2022

In the original version of this article the name of author ‘Grigorios Papageorgiou’ was incorrectly spelled as ‘Grigorios Papageourgiou’. This has been corrected in both the PDF and HTML versions of the article.

