# Supplementary material for: Prospective individual patient data meta-analysis of two randomized trials on convalescent plasma for COVID-19 outpatients
Source: Nat Commun. 2022 May 11;13:2583. doi: 10.1038/s41467-022-29911-3 (PMC9095637; doi:10.1038/s41467-022-29911-3)
Supplement: Supplementary file 1 — Supplementary Information [file 41467_2022_29911_MOESM1_ESM.pdf]

# Supplementary material COMPILE$_{home}$ study

Version, 14th March 2022

## Table of contents


1 Supplementary figures ........ 2

2 Supplementary tables ........ 9

3 Supplementary methods ........ 11

COnV-ert study (NCT04621123) ........ 11

CoV-Early study (NCT04589949) ........ 12

Neutralizing antibody testing and inter-laboratory comparison ........ 13

# 1 Supplementary figures

**Supplementary Figure 1.** Identification and inclusion of potential studies

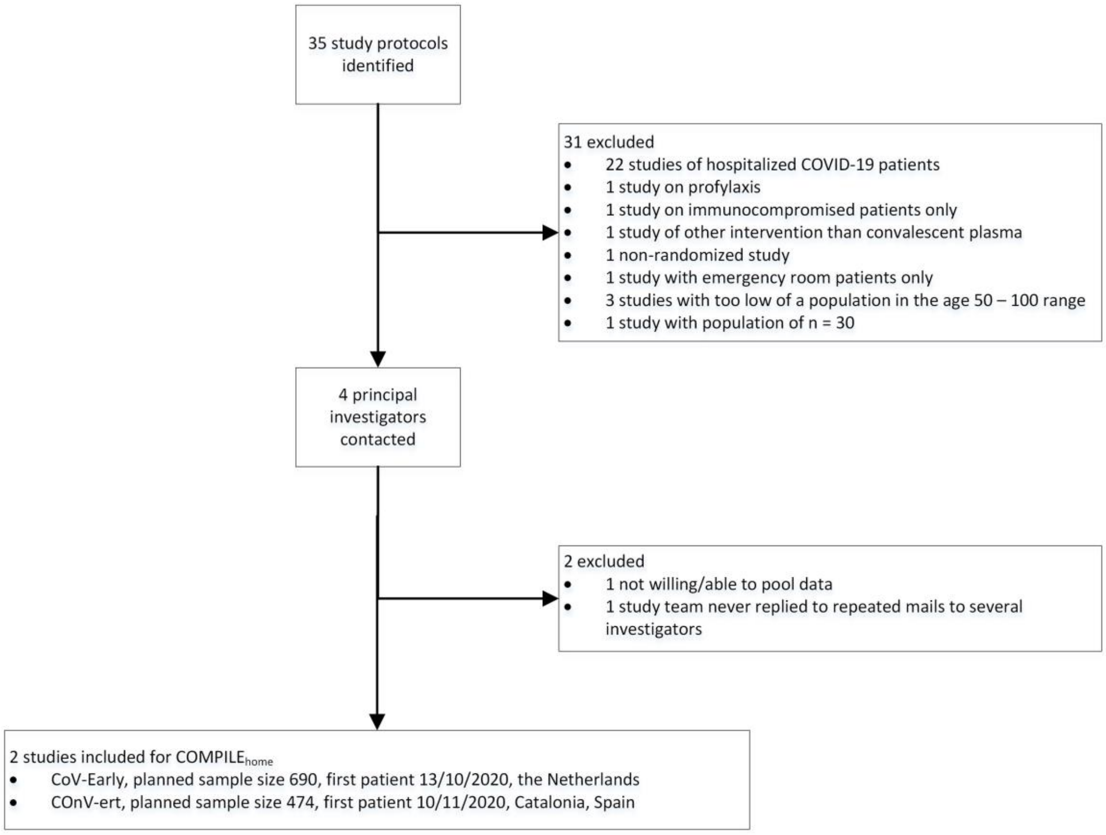


**Supplementary Figure 2:** Weekly inclusion rate in CoV-Early and COnV-ert study

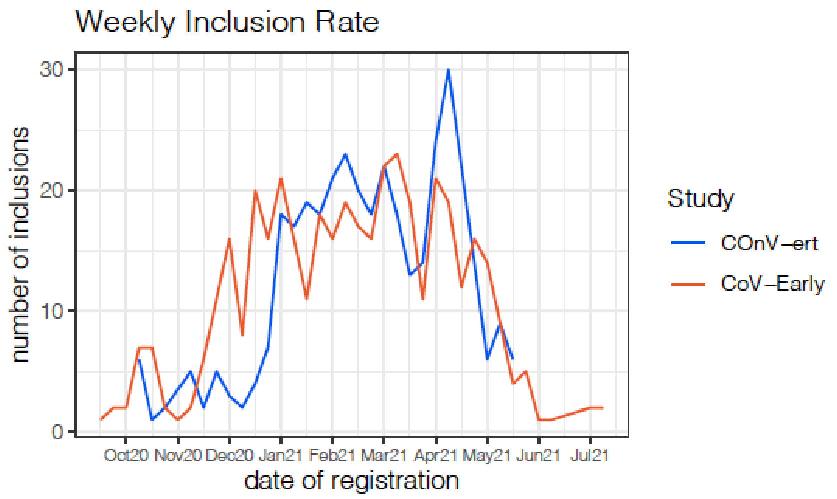

**Supplementary Figure 3.** Circulating variants in the Netherlands during recruitment in the CoV-Early study.

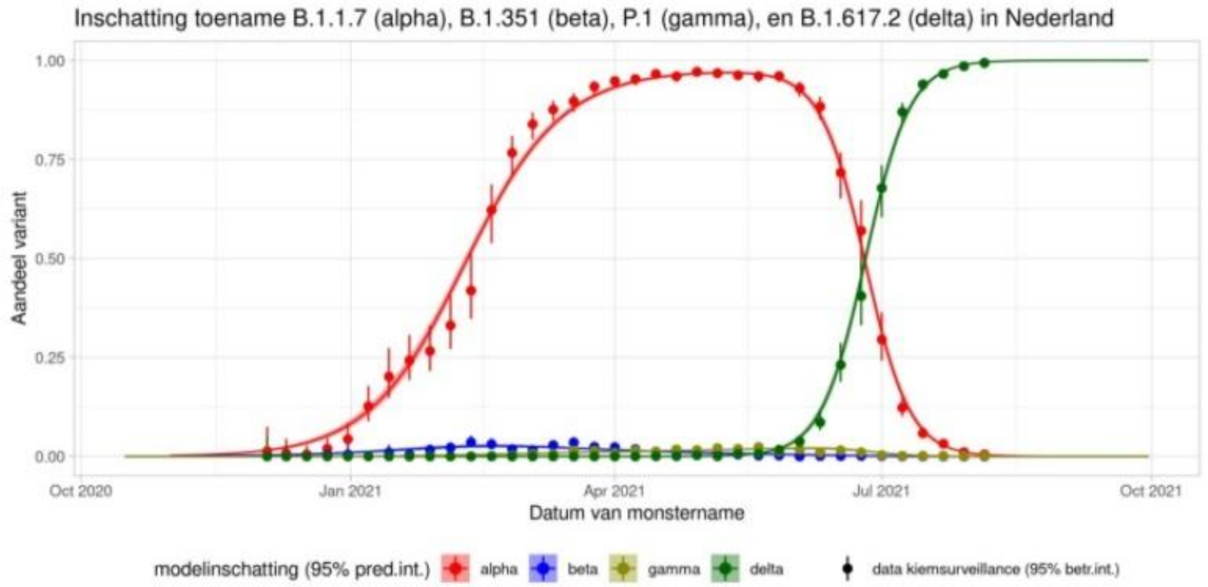


The first patient was included on 13-10-2020 and recruitment ended 13-07-2021. Available from: https://www.rivm.nl/en/coronavirus-covid-19/virus-sars-cov-2/variants

**Supplementary Figure 4.** Circulating variants for each week in 2021 in Spain during recruitment in the COnV-ert study.

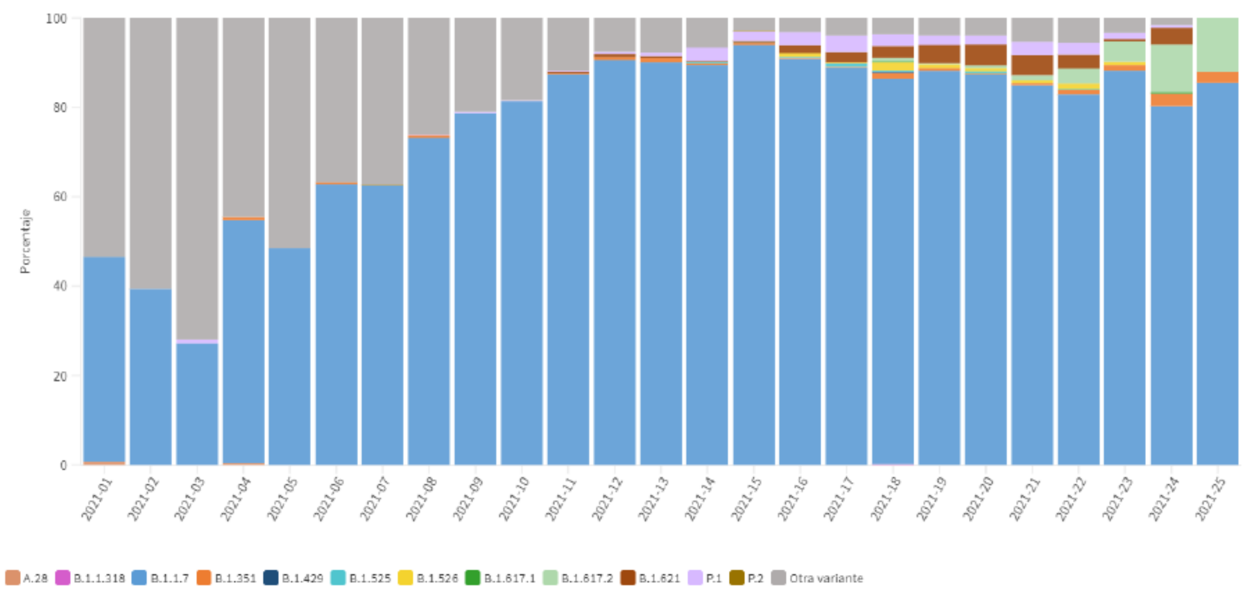


The first patient was recruited on November 10, 2020 and the last patient on May 28, 2021. Note that B.1.351 is the Beta variant (previously known as South-African variant), the B.1.617.2 is the delta variant (previously known as the Indian variant), B.1.1.7 is the alfa or UK variant, while the B.1.621 is also known as the Columbia variant. The grey era (other variants) is assumed to consist almost entirely of the original SARS-CoV-2 virus first isolated in Wuhan. Available from: https://www.mscbs.gob.es/profesionales/saludPublica/ccayes/alertasActual/nCov/documentos/COVID19_Actualizacion_variantes_20210705.pdf

**Supplementary Figure 5.** Odds ratios (ORs) of the primary analysis for the 5-point ordinal disease severity scale.

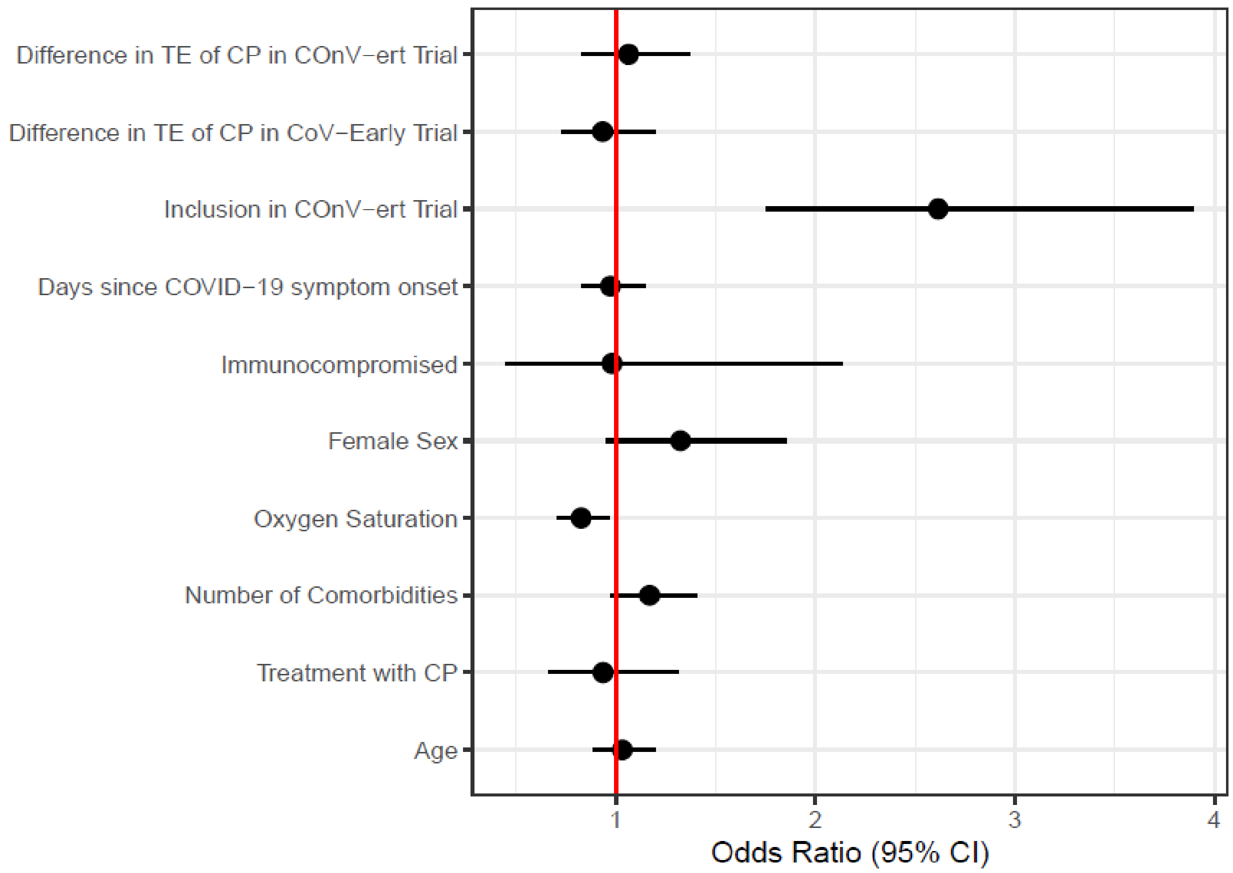


N = 779. Two-sided Wald-type tests, no adjustment for multiple testing. Note that an OR <1.0 denotes improved outcome with CP therapy. CP = Convalescent Plasma. TE = Treatment Effect. The OR (dot) with 95% credible intervals (line) were 0.936 (0.667-1.311) for treatment with convalescent plasma, 0.827 (0.706-0.970) for a higher oxygen saturation (per standard deviation unit), 0.973 (0.827-1.145) for more days (per standard deviation unit) since COVID-19 symptom onset, 1.170 (0.974-1.404) for each additional comorbidity, 2.615 (1.753-3.896) for inclusion in COnV-ert Trial versus CoV-Early, 1.325 (0.956-1.852) for female sex, 1.033 (0.890-1.198) for age (per year above 50), 0.982 (0.451-2.135) for being immunocompromised. The effect size of convalescent plasma was very comparable for both trials as illustrated by the ORs close to 1 for the difference in TE for both trials.

**Supplementary Figure 6.** Odds ratio (OR) for improved outcome on the binary endpoint of hospital admission or death.

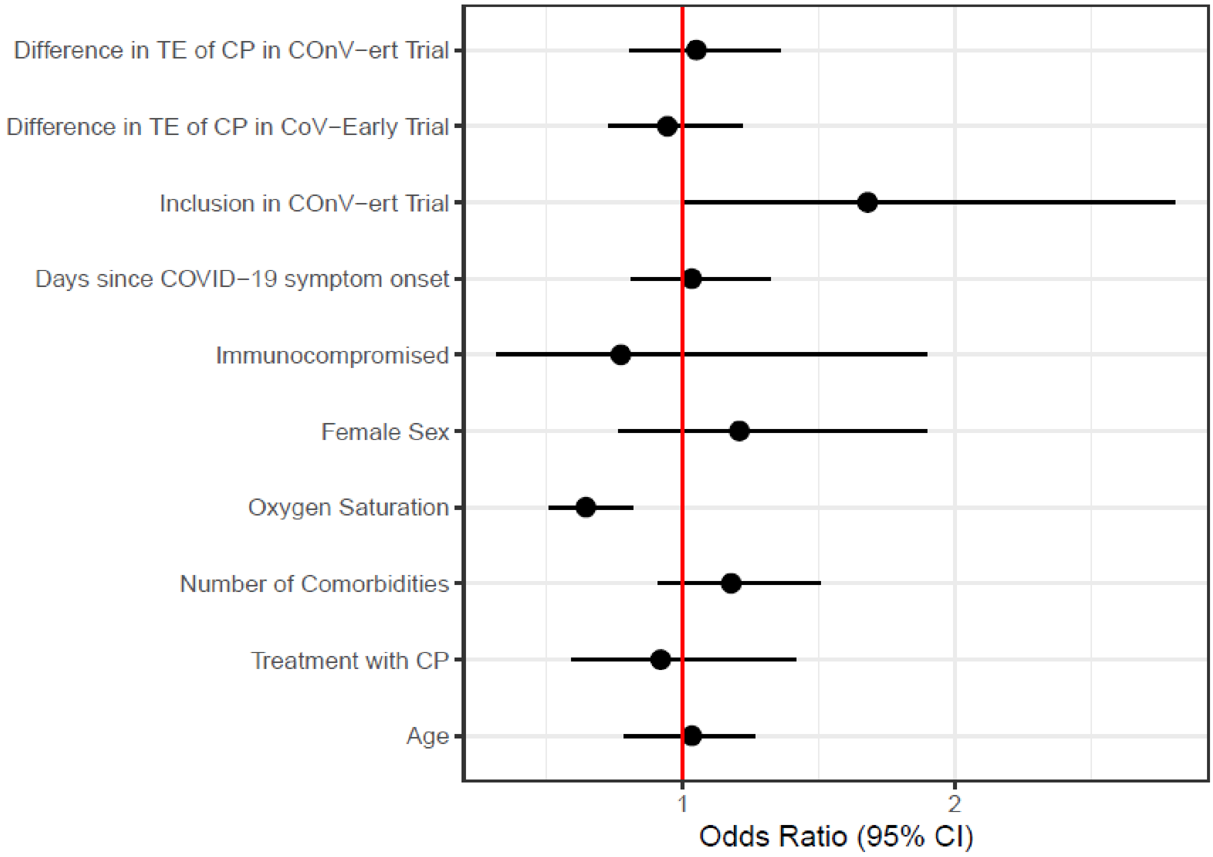


N = 779. Two-sided Wald-type tests, no adjustment for multiple testing. Note that an OR <1.0 denotes improved outcome. CP = Convalescent Plasma. TE = Treatment Effect. The OR (dot) with 95% credible intervals (line) were 0.919 (0.592-1.416) for treatment with convalescent plasma, 0.644 (0.508-0.815) for a higher oxygen saturation (per standard deviation unit), (range 91-99%), 1.033 (0.811-1.321) for more days (per standard deviation unit), since COVID-19 symptom onset, 1.178 (0.908-1.507) for each additional comorbidity, 1.679 (0.992-2.810) for inclusion in COnV-ert Trial versus CoV-Early, 1.208 (0.765-1.894) for female sex, 1.033 (0.765-1.266) for age (per year above 50) and 0.773 (0.318-1.896) for being immunocompromised. The effect size of convalescent plasma was very comparable for both trials as illustrated by the ORs close to 1 for the difference in TE for both trials.

**Supplementary Figure 7.** Antibody status and days since symptoms on hospitalization or death.

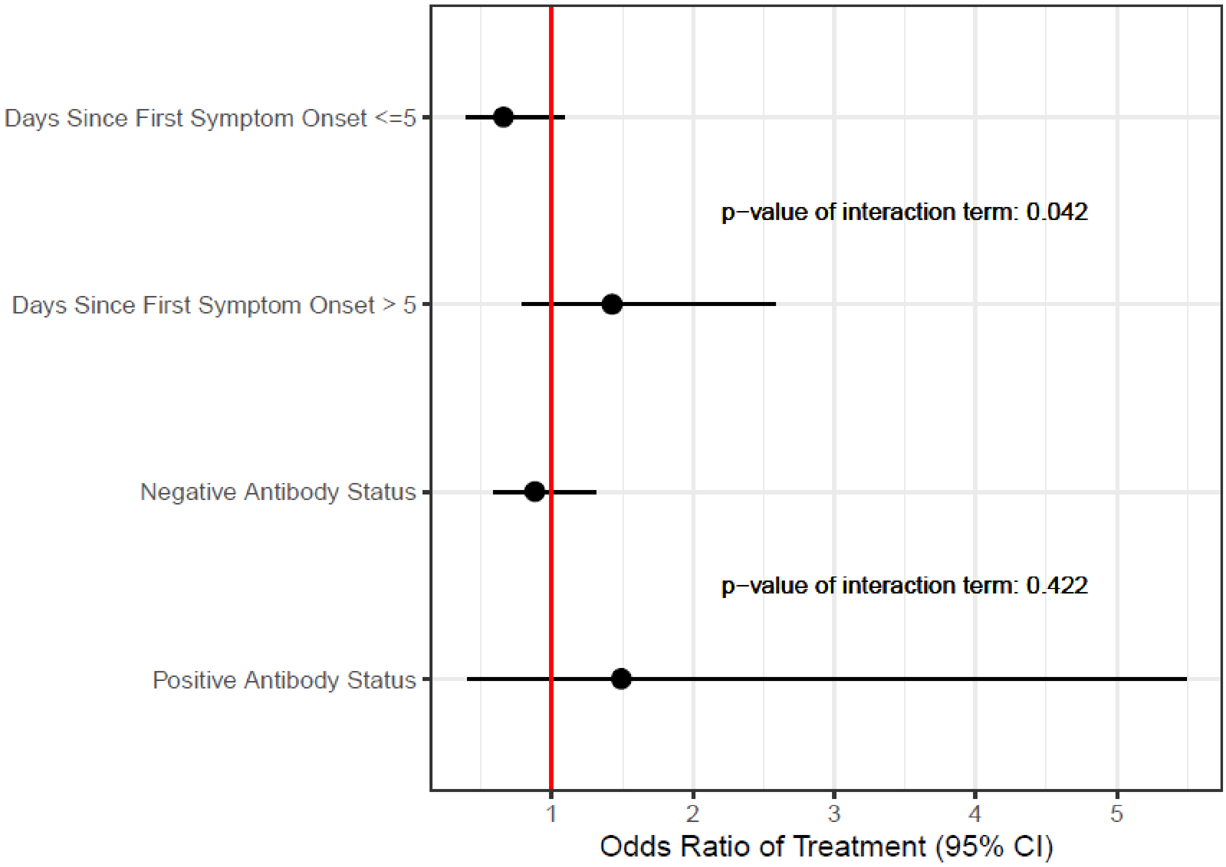


Results of subgroup analysis regarding days since symptom onset and impact of antibody status (positive or negative) of the patient at baseline for the risk of hospital admission or death, n = 779. Two-sided Wald-type tests, no adjustment for multiple testing.  OR (dot) with 95% confidence intervals (line) are given. Please note that an OR <1.0 denotes improved outcome with CP therapy.

**Supplementary Figure 8.** Antibody status and days since symptoms on 5-point scale.

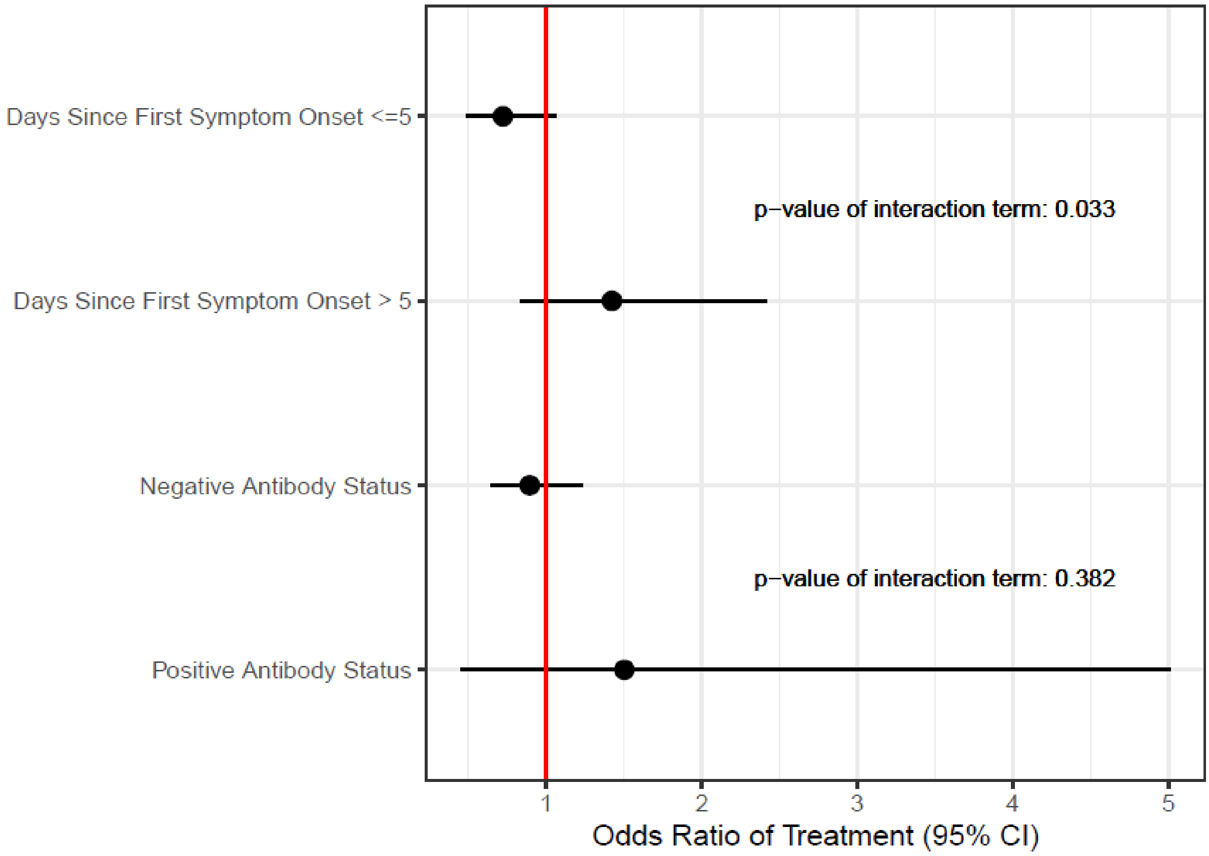


Results of subgroup analysis regarding days since symptom onset, antibody status level at baseline for the 5-point ordinal disease severity scale endpoint, n = 779. Two-sided Wald-type tests, no adjustment for multiple testing.  OR (dot) with 95% confidence intervals (line) are given. Please note that an OR <1.0 denotes improved outcome with CP therapy.

**Supplementary Figure 9.** Antibody level in plasma on hospitalization or death.

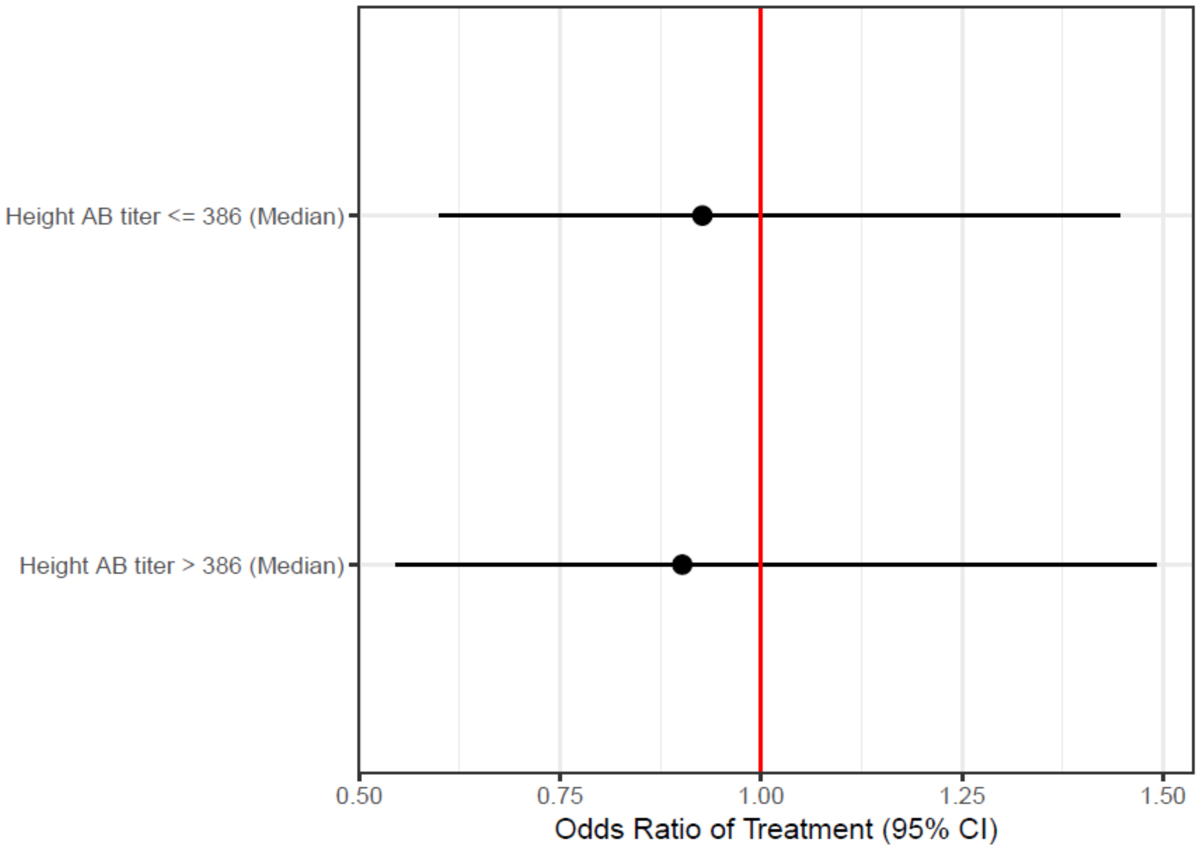


Results of subgroup analysis regarding neutralizing antibody titer level in the convalescent plasma that a patient received for hospital admission or death, n = 779. Two-sided Wald-type tests, no adjustment for multiple testing. OR (dot) with 95% confidence intervals (line) are given. Please note that an OR <1.0 denotes improved outcome with CP therapy. The analysis of this secondary endpoint could not be done according to the original statistical analysis plan. This was caused by differences in the way the height of antibody titer was measured in the two studies (continuous versus integer values) as well as the fact that one of both study labs did not dilute serum further once the titer was >1:640 local units (>1:908 IU/mL). To avoid numerical and interpretation issues caused by these differences as well as the low event rates we needed to change the analysis to a dichotomized analysis at using the median titer of 1:386 IU/mL.

# 2 Supplementary tables

**Supplementary Table 1.** Comorbidity criteria.

| | | Cov-Early | COnV-ert | Compile$_{home}$ |
|---|---|---|---|---|
| 1 | **Obesity** | BMI | BMI | BMI 35 or higher |
| 2 | **Cardiac disease** | AF, HF, CAD, … | HF, CAD | Atrial fibrillation, coronary heart disease, chronic heart Failure, ischemic heart disease, symptomatic atherosclerotic disease other than cardiac |
| 3 | **Lung disease** | COPD or Asthma | COPD and Asthma is registered separately | COPD, Asthma, other chronic lung disease |
| 4 | **Neurological disease** | Stroke or chronic debilitating disease | Cerebrovascular disease | Cerebrovascular disease or chronic debilitating other neurological disease |
| 5 | **Diabetes** | For which medical therapy is given | Any | Diabetes |
| 6 | **Chronic renal failure** | GFR <60 | Is registered but not further defined in CRF | GFR 60 or lower |
| 7 | **Cancer** | Not in complete remission for 1 yr excluding baso/spinocellular skin cancer | Is registered but not further defined in CRF | Cancer not in complete remission for 1 year but excluding baso/spinocellular skin cancer |
| 8 | **Liver disease** | Chronic liver disease leading to cirrhosis or liver dysfunction | Registered in list of other comorbidities | Chronic liver disease with cirrhosis or with liver dysfunction |
| **Other** | | Not registered | Other significant comorbidities are registered in the list of other comorbidities | Not applicable |

Immunodeficiency is not included as a comorbidity. However, it is one of the covariates in the analysis of the primary endpoint and therefore, it is taken into account there. Isolated hypertension without another underlying cardiovascular disease or cardiovascular risk facture is not included as a comorbidity. Abbreviations: AF= atrial fibrillation, BMI = body mass index, CAD= coronary artery disease, COPD = chronic obstructive pulmonary disease; GFR = glomerular filtration rate, HF= heart failure.

**Supplementary Table 2.** Comparison table with similarities and differences between CoV-Early and COnV-ert study protocols.

| | CoV-Early | COnV-ert |
|---|---|---|
| **Inclusion Criteria** | COVID-19, confirmed by PCR or CE-marked antigen test. | Confirmed SARS-CoV-2 infection as determined by PCR or validated antigen rapid diagnostic test from nasopharyngeal swabs ≤5 days prior to inclusion/baseline visit. |
| | Symptomatic (e.g but not limited to fatigue, fever, cough, dyspnoea, loss of taste or smell, diarrhoea, falls or confusion). | Symptomatic with mild or moderate COVID-19 with symptoms onset date ≤ 7 days prior to inclusion/baseline visit. |
| | 70 years or older OR 50-69 years and 1 or more risk factors OR 18-49 and severely immunocompromised. | Adult male or female individuals of ≥50 years old. |
| | | Willing to comply with the requirements of the protocol and available for follow-up for the planned duration of the study. |
| | | Has understood the information provided and capable of giving informed consent. |
| **Exclusion criteria** | Life expectancy <28 days in the opinion of the treating physician. | |
| | Patient or legal representative is unable to provide written informed consent | Inability to consent and/or comply with study protocol, in the opinion of the investigator. |
| | Symptomatic for 8 days or more at the time of screening. | (see Inclusion criteria) |
| | Being admitted to the hospital at the informed consent procedure | Current hospital admission for any cause.<br>Severe or critical COVID-19:<br>a.Severe COVID-19: respiratory frequency >30 breaths per minute, $SpO_2$ <94% on room air at sea level, ratio $PaO_2/FiO_2$ <300 mmHg, or lung infiltrates >50%.<br>b. Critical COVID-19: respiratory failure, septic shock, and/or multiple organ dysfunction. |
| | Known previous history of transfusion-related acute lung injury- | History of allergic reactions to blood or plasma products or methylene blue.<br>Medical conditions for which 200-300 mL of intravenous fluid is considered dangerous (i.e., decompensated heart failure or renal failure with fluid overload). |
| | Known IgA deficiency | Known IgA deficiency with anti-IgA antibodies. |
| | | If female, pregnant or breastfeeding, or planning a pregnancy during the study. |
| | | History of previous confirmed SARS-CoV-2 infection. |
| | | History of significantly abnormal liver function (Child Pugh C). |
| | | History of CKD ≥ stage 4, or need of dialysis treatment. |
| | | Any pre-existing condition that increases risk of thrombosis. |
| **Interventional product** | Single iv infusion of 300 mL of thawed convalescent plasma, preferably ABO-identical<br>+ Standard medical treatment | Single iv infusion of 200-300 mL of convalescent plasma, preferably ABO-identical<br>+ Standard medical treatment |
| **Control arm** | Single iv infusion of 300 mL of thawed non-convalescent plasma (fresh frozen plasma), preferably ABO-identical<br>+ Standard medical treatment | Single iv infusion of 200 to 300 mL of sterile saline solution 0.9%<br>+ Standard medical treatment |
| **Convalescent plasma donor criteria** | Donors had a history of PCR proven symptomatic COVID-19 and had recovered from COVID-19 for at least 14 days. | Donors had a history of PCR proven symptomatic or asymptomatic COVID-19 and had recovered from COVID-19 for at least 28 days. |
| | Plasma donors were selected based on a virus neutralization titer of at least 1:160. | Plasma donors were selected based on an Euroimmun test with OD of at least 6.0. |
| | Each donation 600 mL plasma was collected in 2 bags of 300 mL each. Plasma was stored at minus 25 degrees Celsius or colder and tested for pathogens during routine procedures. | Each donation was of a maximum of 600 mL plasma, and it was collected in 2 bags of 200-300 mL each. Plasma was treated with blue methylene and stored at minus 25 degrees Celsius or colder and tested for pathogens during routine procedures. |
| | Every plasma had a unique identification number by which the product can always be traced back to the donor.<br>When plasma was administered, this number was registered in the patient file. | Every plasma had a unique identification number by which the product can always be traced back to the donor.<br>When plasma was administered, this number was registered in the blinded tab of the eCRF for each study participant. |
| | Administration of conv plasma or fresh frozen plasma was blinded by masking the plasma bag with an opaque bag wrapped around the plasma bag. The transfusion lab personal received the allocation email and wrapped the concealment bag around the plasma bag. | Administration of conv plasma or saline solution was blinded by masking the plasma bag with an opaque bag wrapped around the plasma bag. The transfusion lab received the allocation email. An unblinded study nurse (since the IV perfusion system was not masked) was in charge of the infusion of the iv products. The rest of the investigators and study nurses remained blinded. |

Abbreviations: CKD = chronic kidney disease; eCRF = electronic case report form; IV = intravascular; OD = optical density; PCR = Polymerase Chain Reaction.

# 3 Supplementary methods

The complete CoV-Early and COnV-ert study protocols are available as online supplements. A short summary is given here.

## COnV-ert study (NCT04621123)

**Trial design**
The COnV-ert study was a multicenter, double-blinded, randomized, controlled trial to assess the efficacy of convalescent plasma in preventing severe COVID-19 in patients infected with SARS-CoV-2 with mild and moderate illness. The trial was conducted between November 2, 2020 and July 28, 2021 at four healthcare centers providing universal healthcare to a catchment population of 3,883,700 in Catalonia, Spain.

The study was conducted according to the Helsinki Declaration of the World Medical Association, and the study protocol was approved by the Ethics Committee at Hospital Germans Trias i Pujol (number PI 20-313) and the institutional review boards of the rest of participating centers. All patients provided written informed consent before enrolling the study, which was supervised by an independent data and safety monitoring board. The trial was registered in ClinicalTrials.gov (NCT04621123).

**Participants**
We included outpatients aged ≥50 years with mild-to-moderate COVID-19 confirmed by RT-PCR or antigen rapid test ≤5 days before randomization and symptoms onset ≤7 days. Mild and moderate COVID-19 were defined according to international guidelines as follows: patients with fever, cough, sore throat, malaise, headache, and muscle pain were considered mild COVID-19, whereas evidence of lower respiratory disease by clinical assessment or imaging and a saturation of oxygen ≥94% on room air was considerate moderate COVID-19. Patients were excluded if they had severe COVID-19 or required hospitalization for any cause, a previous SARS-CoV-2 infection, contraindications with the investigational product, increased thrombotic risk, a history of significantly abnormal liver function (e.g., Child Pugh C), chronic kidney disease stage ≥ 4. Female participants pregnant or breastfeeding or planning a pregnancy during the study were also excluded. Further details on the eligibility criteria are listed in the full protocol that is included as an online supplement.

**Trial Procedures**
Study candidates were identified from two sources: (1) we actively screened the healthcare records of study sites for individuals with evidence of SARS-CoV-2 infection and (2) individuals who tested positive for SARS-CoV-2 infection during epidemiological surveillance could voluntarily register to an institutional website launched by the sponsor and the Catalan Institute of Health. Investigators contacted candidates by phone or in person to inform them about the study, invite them to participate, and check their suitability. Suitable candidates were scheduled a baseline visit, performed either at the hospital or at home by the hospital domiciliary care unit, in which written informed consent was obtained, and the eligibility confirmed.

Eligible patients who provided written informed consent were randomly assigned (1:1) using a computer-generated random-number list to receive one intravenous (IV) infusion of either 200-300 mL of ABO-compatible high-titer convalescent plasma (experimental arm) or 250 mL of sterile saline solution 0.9% (control arm). The study convalescent plasma was selected after being screened for high anti-SARS-CoV-2 IgG titers with ELISA (EUROIMMUN ratio ≥6), according to guidelines, and supplied by the regional blood bank *(Banc de Sang i Teixits de Catalunya* – BST).[1] Further details on the preparation and characteristics of the plasma are provided in the in the full protocol that is included as an online supplement.

Trained BST staff masked the investigational product with opaque tubular bags that covered the entire infusion catheter to prevent product identification. The masked investigational product was infused over

30 minutes. Patients and all investigators who participated in the trial (including laboratory staff and the statistician) were blinded to treatment allocation, except the baseline study nurses and BST trained personnel, who were not involved in the participants' follow-up.

Follow-up visits were scheduled on days 7 and 28; additionally, we contacted study patients by phone on days 3, 14, and 60 for assessing their clinical status. During follow-up visits, we obtained blood samples (baseline and day 7) for assessing inflammatory markers and nasopharyngeal swabs (baseline and days 7 and 28) for quantification of SARS-CoV-2 viral load, analyzed by RT-qPCR in a centralized laboratory. Serologic status of all enrolled participants (serum antibody positive or serum antibody negative) were prospectively characterized from baseline samples. All collected data were recorded in an electronic case report form.

**Follow-up**

We defined two primary outcomes regarding treatment efficacy: the clinical outcome was the hospitalization rate on a time frame of 28 days after treatment, and the virological outcome was viral load reduction in nasopharyngeal swabs at day 7 and 28.

Prespecified secondary outcomes were time to complete symptom resolution, change in the 10-point WHO Clinical progression scale score within the 60 days following infusion and change in inflammatory parameters (ferritin, prealbumin, interleukin 6 (IL-6), D-dimer, C reactive protein (CRP)) from baseline to day 7 of follow-up.

Safety was assessed as the proportion of patients with adverse events that occurred or worsened during the follow-up period. Adverse events were assessed for seriousness and causality.

More details are available in the full protocol that is included as on online supplement

## CoV-Early study (NCT04589949)

**Trial design**

The CoV-Early study was a multicenter, double-blinded, randomized, controlled trial to assess the efficacy of convalescent plasma in preventing severe COVID-19 in patients infected with SARS-CoV-2 with mild and moderate illness at 11 hospitals across the Netherlands. The first patient was included on 13-10-2020 and recruitment ended 13-07-2021.

The study was conducted according to the Helsinki Declaration of the World Medical Association, and the study protocol was approved by the competent authority of the Netherlands (CCMO) and the institutional review board of the Erasmus MC University Medical Center in Rotterdam as well as the board of directors of each of the participating hospitals. All patients provided written informed consent before enrolling the study. The conduct was supervised by an independent data and safety monitoring board. The trial was registered in ClinicalTrials.gov with NCT04589949.

**Participants, recruitment and trial procedures**

Outpatients diagnosed with COVID-19 by PCR or antigen testing and symptomatic for <8 days could be screened. Unless they were severely immunocompromised, they had to be at least 50 years old and have at least one risk factor associated with a higher risk of severe COVID-19. Further details can be found in the full protocol available as an online supplement.

The study was communicated with the Dutch public using all kinds of media including newspapers, medical journals for general practitioners, public health free-of-charge COVID test centers as well as social media. Patients aged 50 or older that tested positive for SARS-CoV-2 at a public health SARS-CoV-2 test centers were contacted by telephone about the positive result of their test by the test center and informed about the possibility of study participation at a nearby hospital. When they showed interest and agreed to be

contacted by the study team, their telephone number was shared with the study team and the patient was contacted to get additional information. When the patient fulfilled the in- and exclusion criteria and wanted to participate, he/she received an appointment at the nearest study site the next day. Self-referral was possible as well via www.cov-early.nl or www.coronaplasmastudie.nl

**Screening at study site, baseline visit and follow-up**
All study sites had convalescent plasma for all ABO blood groups available on site. The official screening and baseline visit were done consecutively. Regarding the screening visit, the in- and exclusion criteria were checked again, oxygen saturation was measured to exclude patients with a saturation <93%, written informed consent was obtained, a nasopharyngeal swab was taken, ABO blood group was determined and serum was collected. Eligible patients were randomized using an online randomization tool incorporated in the eCRF (ALEA). The allocation code was mailed to the transfusion lab of the hospital. They provided the study team with one unit of convalescent or control plasma. Masking of investigators and the patient was done by the transfusion lab with the use of a non-transparent concealment bag around the plasma unit. After transfusion, the patient was observed for at least 30 minutes and then could leave the hospital.

**Follow-up**
Patients or when needed their representative or general practitioner were contacted on day 7, day 14 and 28 to evaluate their disease status and severity on a scale from 0 to 5. If a patient had recovered completely (no further symptoms attributable to COVID-19 except for loss of smell or taste), the date of full symptom resolution was registered. Patients that also agreed to participate in a virology and immunology substudy came back to the hospital on several occasions (see full protocol for more details). Patients that participated in the geriatric substudy (age 70 or older) were contacted for a more detailed evaluation (e.g. frailty score, see full protocol for more details).

More details are available in the full protocol that is included as on online supplement

## Neutralizing antibody testing and inter-laboratory comparison

As both study labs used a different SARS-CoV-2 neutralizing antibody test, a panel of 15 plasma samples was provided for comparison by the Support-E consortium.[2] This panel included a research reagent 20/130 obtained from the National Institute for Biological Standards and Control (NIBSC, United Kingdom), which had been assigned a unitage of 1,300 international units (IU)/mL of SARS-CoV-2-neutralising antibodies. A further dilution series of a high-titre convalescent plasma sample (initial neutralising antibody titre of 1:5120 provided as neat, and diluted in 1:10, 1:50 and 1:100) was calibrated in IU/mL against this research reagent, and used to assess the linearity of both assays. This allowed retrospective conversion of neutralizing antibody titers into international units (IU/mL) using linear regression formulae derived from assay calibration as shown below for each trials.

**Methods for the neutralizing antibody titer measurement**

**COnV-ert study:** a sample of each convalescent plasma bag was sent to a centralized laboratory (*IrsiCaixa laboratory*) for prospective characterization of neutralizing antibody titers. More than one participant could receive plasma from the same donor. Pseudovirus generation and neutralization assay: HIV reporter pseudoviruses expressing SARS-CoV-2 S protein and Luciferase were generated. pNL4-3.Luc.R-.E- was obtained from the NIH AIDS Reagent Program SARS-CoV-2.[3] SctΔ19 was generated (GeneArt) from the full protein sequence of SARS-CoV-2 spike with a deletion of the last 19 amino acids in C-terminal, human-codon optimized and inserted into pcDNA3.4-TOPO.[4] Expi293F cells were transfected using ExpiFectamine293 Reagent (Thermo Fisher Scientific) with pNL4-3.Luc.R-.E- and SARS-CoV-2.SctΔ19 (WH1, B.1.1.7 or B.1.351), at an 8:1 ratio, respectively. Control pseudoviruses were obtained by replacing the S protein expression plasmid with a VSV-G protein expression plasmid as previously reported.[5] Supernatants were harvested 48 hours after transfection, filtered at 0.45 µm, frozen, and titrated on HEK293T cells overexpressing WT human ACE-2 (Integral Molecular, USA). The neutralization assay has been previously

validated in a large subset of samples with a replicative viral inhibition assay.[6] Briefly, neutralization assays were performed in duplicate in Nunc 96-well cell culture plates (Thermo Fisher Scientific), 200 TCID50 of pseudovirus were preincubated with three-fold serial dilutions (1:60–1:14,580) of heat-inactivated plasma samples for 1 hour at 37ºC. Then, $2x10^4$ HEK293T/hACE2 cells treated with DEAE-Dextran (Sigma-Aldrich) were added. Results were read after 48 hours using the EnSight Multimode Plate Reader and BriteLite Plus Luciferase reagent (PerkinElmer, USA). Neutralization capacity of the plasma samples was calculated by comparing the experimental Relative Light Units (RLU) calculated from infected cells treated with each plasma to the max RLUs (maximal infectivity calculated from untreated infected cells) and min RLUs (minimal infectivity calculated from uninfected cells), and expressed as percent neutralization: %Neutralization = (RLUmax–RLUexperimental)/(RLUmax–RLUmin)*100. The ID50 (reciprocal dilution inhibiting 50% of the infection) was calculated by plotting and fitting the log of plasma dilution vs. normalized response to a 4-parameters equation in Prism 9.0.2 (GraphPad Software, USA).

To facilitate conversion to International Units (IU), a calibrated panel of plasma samples containing a dilution series of a high titer convalescent plasma calibrated in IU/mL using the standard 20/130 obtained from the National Institute for Biological Standards and Control (NIBSC, United Kingdom).[2] Experimental neutralization titers were converted to IU/mL using the following regression formula (IU/mL = $4160/(2^\wedge(Log_2^{(experimentalID50-13.962)/-0.9798})$ derived from assay calibration with the pre-quantified control.

**CoV-Early study:** A serum sample from each plasma donor taken on the day of plasma donation was sent to the RIVM lab for virus neutralization testing. Duplicates of two-fold serial dilutions (starting at 1:10) of heat-inactivated sera (30 m, 56°C) were incubated with 100 median tissue culture infectious dose of SARS-CoV-2 strains hCoV-19/Netherlands/ZuidHolland_10004/2020, D614G (WT) and hCoV-19/Netherlands/ NoordHolland_10159/2021 (B.1.351, EVAg, catalog no. 014 V-04058) at 35°C for 1 hour in 96-well plates. Vero E6 cells were added in a concentration of 20,000 cells per well and were incubated for 72 hours at 35°C. The serum virus neutralization titer was defined as the reciprocal value of the sample dilution that showed a 50% protection of virus growth. Samples with titers of ≥20 were defined as SARS-CoV-2 seropositive.

To facilitate conversion to International Units (IU), a calibrated panel of plasma samples containing a dilution series of a high titer convalescent plasma calibrated in IU/mL using the standard 20/130 obtained from the National Institute for Biological Standards and Control (NIBSC, United Kingdom).[2] Experimental neutralization titers were converted to IU/mL using the following regression formula (IU/mL = $4160/(2^\wedge(Log_2^{(experimentalID50-11.832)/-1.146})$ derived from assay calibration with the pre-quantified control.

**References**


1. Convalescent Plasma EUA Letter of Authorization 06032021. 2021.
2. Nguyen D, Simmonds P, Steenhuis M, et al. SARS-CoV-2 neutralising antibody testing in Europe: towards harmonisation of neutralising antibody titres for better use of convalescent plasma and comparability of trial data. Euro Surveill 2021;26.
3. Connor RI, Chen BK, Choe S, Landau NR. Vpr is required for efficient replication of human immunodeficiency virus type-1 in mononuclear phagocytes. Virology 1995;206:935-44.
4. Trinité B, Pradenas E, Marfil S, et al. Previous SARS-CoV-2 Infection Increases B.1.1.7 Cross-Neutralization by Vaccinated Individuals. Viruses 2021;13:1135.
5. Sánchez-Palomino S, Massanella M, Carrillo J, et al. A cell-to-cell HIV transfer assay identifies humoral responses with broad neutralization activity. Vaccine 2011;29:5250-9.
6. Trinité B, Tarrés-Freixas F, Rodon J, et al. SARS-CoV-2 infection elicits a rapid neutralizing antibody response that correlates with disease severity. Scientific Reports 2021;11:2608.

# Statistical analysis plan for COMPILE*home*

V4.1 September 1, 2021

**Authors:**
Greg Papageourgiou
Nan van Geloven
Bart Rijnders

## Table of content

Signature page ........ 2
1 Introduction and rationale ........ 3
Study Hypotheses ........ 3
2 Study Objectives and Endpoints ........ 3
Primary Objectives ........ 3
Secondary Objectives ........ 4
3 Study Methods ........ 4
General Study Design and Plan ........ 4
Inclusion and Exclusion Criteria and General Study Population ........ 5
Randomization and Blinding ........ 5
Minimal data set ........ 5
Data Sources ........ 7
Data Merger ........ 7
Ensuring Complete Data De-identification ........ 7
4 Statistical Considerations ........ 8
Sample Size ........ 8
Timing of Analysis ........ 8
Covariates and subgroups ........ 8
Missing Data ........ 9
Interim Analysis and Data Monitoring ........ 9
Scope of Interim Analysis ........ 9
Interim Analysis Results and the Collective Data Safety Monitoring Board ........ 10
Stopping Rules ........ 11
Primary Efficacy Analysis ........ 11
Worst disease severity score on a 5-point ordinal scale at day 28 after transfusion ........ 11
Hospitalization Rate ........ 12

# 1 Introduction and rationale

### Study Hypotheses

The compilation of a pooled dataset of de-identified individual patient data (IPD)from randomized clinical trial (RCTs) collaborating in the **COMPILE*home*** Consortium will result in a dataresource that will provide evidence with high degree of certainty regarding the efficacy (or harm) andsafety of convalescent plasma (CP) as a treatment for outpatients diagnosed with COVID-19. The COMPILE *home* Consortium RCTs are trials of CP against control treatment in the target population of outpatients with a confirmed COVID-19 diagnosis within 7 days after symptoms onset.

# 2 Study Objectives and Endpoints

### Primary Objectives

The purpose of this study is to evaluate the efficacy following the administration of convalescent plasma (ConvP) as a therapy for outpatients diagnosed with COVID-19 at an increased risk for an unfavorable clinical outcome and within 8 days after symptom onset. This will be achieved by poolingde-identified IPD from two independent but similar RCTs, and all outcomes will be continuously monitored using a pre-established stopping guideline for efficacy. A minimal data set (MDS) of IPD data will be identified and each independent RCT will submit an updated MDS every 4 weeks to updatethe pooled dataset with newly enrolled patients and/or new data from already enrolled patients.

### Primary Endpoint

The primary efficacy outcome will consist of two outcome measures:

1. Worst disease severity score within 28 days. The highest disease status on the 5-point ordinaldisease severity scale in the 28 days following transfusion:

   1 = Fully recovered (no symptoms) within 7 days after transfusion

   2 = Continued symptoms attributable to COVID – 19 on day 7 after transfusion

   3 = Admitted to hospital but no invasive ventilation needed

   4 = Admitted to hospital and invasive ventilation needed

   5 = Death

2. Hospitalization OR death rate: scoring 3 or higher on the 5-point ordinal disease severity scaleas described above up to 28 days after transfusion.

### Secondary Objectives

The secondary objective of this study is to generate a pooled dataset of de-identified IPD from participating RCTs, to study (timing of) individual components of the disease severity scale and to study differential efficacy in subgroups.

### Secondary Endpoints

1. Time to full symptom resolution.
2. Safety of CP in outpatients with COVID-19.

The secondary endpoints will not be analyzed on a 6-weekly basis. When deemed necessary, the cDSMB can request to perform these analyses within the extent possible as some of these outcome measures will not be available on short notice.

#### Subgroup Analyses

**The following endpoints will be evaluated in subgroups of patients:**

1. Efficacy according to 5-point ordinal scale in those with days from symptom onset (DFSO) of 1 to 5 days.
2. Efficacy according to binary outcome of hospital admission or death in those with DFSO of 1 to 5 days.
3. Efficacy according to the 5-point ordinal scale in SARS-CoV-2 in relation to height of neutralizing antibody titers in transfused plasma.
4. Efficacy according to the binary outcome of hospital admission or death in SARS-CoV-2 in relation to height of neutralizing antibody titers in transfused plasma.
5. Efficacy according to the 5-point ordinal scale in SARS-CoV-2 according to antibody positive vs negative patients.
6. Efficacy according to the binary outcome of hospital admission or death according to antibody positive vs negative patients .

## 3 Study Methods

### General Study Design and Plan

This is a Bayesian Adaptive IPD Meta-Analysis (IPD MA) of a collection of multi-center RCTs to evaluate whether CP is superior compared to non-CP treatment. We will report the posterior distribution of the treatment effect estimates. Control arm may vary by RCT; the control arms in the trials included in this IPD MA could be: a) Fresh Frozen Plasma, b) saline with or without coloring agent and c) standard of care.

Randomization schemes may vary across RCTs. That is in some cases, randomization can be stratified by site of care, while in other cases there will be no stratification. Blinding across studies may vary depending on the control treatment used. Endpoint evaluation for the first of both primary endpoints will be blinded for all participating studies. Data from studies thathave no blinded endpoint evaluation will only be used for the analysis of the second primary endpoint(hospital admission).

### Inclusion and Exclusion Criteria and General Study Population

The COMPILE$_{home}$ study will evaluate the efficacy following the administration of CP as a therapy for outpatients diagnosed with COVID-19 and within 8 days after symptom onset.

Inclusion and exclusion criteria may vary across RCTs. However, all the subjects to be included in the pooled analysis must fulfill the following criteria:

- Participant in a qualifying RCT for CP assigned to either CP or control treatment arm.
- Confirmed COVID-19 diagnosis by a CE of FDA approved diagnostic test.
- Not hospitalized at time of randomization
- Symptomatic with symptoms onset date <8 days at screening and up to and including 8 days on the day of the baseline visit/the intervention.
- The RCT has to collect minimally one of the efficacy outcomes as described on section 2.1.1 and the minimal required additional data as described by the definition of the minimal data set (MDS) in section 4.4.
- Aged 50 or older

Furthermore, only patients that received the intervention as assigned will be included in the analysis.So, the population will consist of a modified intent to treat population, where patients that are randomized but who did not receive the intervention for any reasons will not be included.

### Randomization and Blinding

Randomization and blinding schemes will vary by study. Only data from studies in which the endpoint evaluation was blinded for the evaluator will be used for the first of both primary endpoints. For the second primary endpoint of hospitalization or death, unblinded data can be used as well.

### Minimal data set

Table 1 below lists the minimally required variables that need to be collected by an individual RCT to participate in the pooling initiative. Table 2 lists additional data to be collected if available.

| **Table 1** Minimal data set | |
|---|---|
| Data | Type |
| **Date of randomization** | Date |

| | |
|---|---|
| **Demographics** | |
| Age | In years; NA = not available |
| Sex | 0 = male; 1 = female; NA = not available |
| **Status at Enrollment** | |
| Number of comorbidities<br>Immunocompromised status (to be specified) | #; NA = not available0 = no; 1 = yes |
| **Status at Transfusion** | |
| Duration of symptoms on day of transfusion | In days; NA = Not available |
| **Outcome data (at least one of both is required)Outcome data part 1** | |
| Worst disease severity score within 28 days since randomization | 1 = Fully recovered within 7 days after transfusion<br>2 = Continued symptoms on day 7 after transfusion<br>3 = Admitted to hospital but no invasive ventilation needed<br>4 = Admitted to hospital and invasive ventilation needed<br>5 = Death; NA = not available |
| **Outcome data part 2** | 1 = Not admitted and not death on or before day 282 = Admitted or death on or before day 28 |

| **Table 2** Additional Data | |
|---|---|
| Data | Type |
| **Status at Enrollment** | |
| BMI | In kg/$m^2$; NA = not available |
| O2 saturation | In %; NA = not available |
| *Result of baseline antibody test in patient* (*) | *1=Positive; 2=Negative; NA = not available* |
| *Height of the neutralizing antibody titer in theplasma that was transfused in PRNT50* (*) | *1-100.000; NA = not available* |
| **Outcomes** | |
| Date of complete resolution of symptoms | Date of symptom resolution; NA = not available |
| Date of death | Date of death; NA = not available |
| Time to hospital admission | Date of hospital admission; NA = not available |
| Time to ICU admission for invasive ventilation | Date of start of invasive V; NA = not available |
| *Quantitative PCR test results on day 1*(*) | *To be defined (Ct values, or copy numbers)* |
| *Quantitative PCR test results on day 3*(*) | *To be defined (Ct values, or copy numbers)* |
| *Quantitative PCR test results on day 7*(*) | *To be defined (Ct values, or copy numbers)* |
| *Quantitative PCR test results on day 14*(*) | *To be defined (Ct values, or copy numbers)* |

*(*)Data that will not be available real-time and that are not part of the primary endpoint analysis*

### Data Sources

Individual RCTs will transfer IPD to Erasmus MC by secure file transfer protocol (FTP) using the …

The first transfer will include data on qualifying patients currently enrolled in the RCTs and of which the minimum data set is available including the day-28 evaluation. Subsequent transfers will take placeevery 4 weeks and will contain new data from previously enrolled subjects, data from newly enrolledsubjects, and any corrections of data previously submitted. Each new dataset will supersede the previously submitted data from a given RCT in order to allow for the trials to correct newly discoveredimperfections in the data (e.g., missing values, incorrect entries etc.). If there are no updates to the previous transmission, the RCT teams will explicitly inform the pooled database manager.

Each RCT will be provided with a unique COMPILE$_{home}$ study ID, recruitment site IDs as needed, and patient IDs. Each trial will keep a log that will match the trial-specific IDs to the COMPILE$_{home}$ study andpatient IDs. The matching log will be kept locally and will never be provided to the COMPILE$_{home}$ Consortium, but will exist at the local site for quality control purposes. This will provide further securityof the RCT participants' protected information. The COMPILE$_{home}$ data dictionary and instructions for preparing and submitting the data sets can be found in the Data Dictionary and Instructions document.

### Data Merger

Erasmus MC will maintain the COMPILE$_{home}$ Consortium data in a secure environment. Files of RCTs received at Erasmus MC will be pooled into the COMPILE$_{home}$ Consortium dataset, which will be maintained by Erasmus MC as a common database within a secure drive.

### Ensuring Complete Data De-identification

To ensure complete data de-identification in compliance with HIPPA and GDPR, the following steps will be taken:

1. The RCTs will be provided with logs giving a list of specific COMPILE$_{home}$ RCT ID, site within RCT ID and patient within site within RCT ID. The data managers responsible for submitting data to the COMPILE$_{home}$ dataset will map the RCT-specific patients' IDs to the COMPILE$_{home}$ subject ID. This mapping log will only be available at the location of the RCT and will never be made available to the COMPILE$_{home}$ Consortium. An example of a log with COMPILE$_{home}$ subjects IDs is presented below. Suppose a collaborating RCT has 2 recruitment sites. The RCT will receivea log similar to this:

| RCT Name | Site | RCT Subject ID | COMPILE*home* Study Identifier | COMPILE*home* Site Identifier | COMPILE*home* Subject Identfier |
|---|---|---|---|---|---|
| | | | X | 1 | 001 |
| | | | X | 1 | 002 |
| | | | X | 1 | 003 |
| | | | . | | |
| | | | XX | 1 | 150 |
| | | | XX | 2 | 001 |
| | | | XX | 2 | 002 |

The shaded columns will be required in the minimal data set. The RCT will keep the log locally to allow mapping the RCT original patients' IDs to the COMPILE$_{home}$ IDs. The log will never be shared with the COMPILE$_{home}$ Consortium. More COMPILE$_{home}$ subject IDs will be added in an obvious fashion if a recruitment site has more than 100 patients enrolled. A similar extension will be made if an RCT adds additional sites during the trial's conduct. Dddd

The transferred data will only be identified by the COMPILE$_{home}$ identifiers, including the names of the data file.

# 4 Statistical Considerations

### Sample Size

The number of studies included in the IPD MA will not be restricted. There is no pre-determined maximum number of patients. The continuous monitoring will continue until the DSMBs have determined that there is sufficient evidence to recommend stopping the study. This may be when the stopping guideline has signaled efficacy or when all or most of the included studies have finished enrollment or recruitment is not expected to be substantial anymore and follow up and new studies on the topic are not expected.

### Timing of Analysis

Continuous monitoring and analysis will occur every 6 weeks. The final analysis will occur when the DSMBs have recommended stopping the study.

### Covariates

The primary outcome model will include as covariates: age, sex, number of comorbidities per category(0-9), oxygen saturation at baseline (in %), immunocompromised state (Y/N) and duration of time since COVID-19 symptom onset as covariates (only main effects).

## Missing Data

We do anticipate some missingness in the covariates and/or outcome data. While RCT investigators will be asked to provide the worst disease severity score on the 5-point ordinal disease severity scale and hospitalization rate for all trial participants, the actual outcomes at day 28 might e.g. not be available from patients that have been discharged from the hospital prior to those assessment dates. If a patient is discharged prior to 28 days RCT investigators are asked to make all possible efforts to obtain those patients' status on day 28. If despite all efforts, the worst disease status and/or hospitalization rate of discharged patients is unavailable at day 28, we will use the worst disease status and/or hospitalization rate at the last known time point for the 28-day measurement. Some covariates are expected to be missing on some patients or for the entire RCT, if the RCT was not collecting a specific variable from the minimal data set. The primary analysis will only control for age, sex, comorbidities and duration of COVID-19 symptoms. While we anticipate that those variables are collected in all RCTs, in case we receive data sets that have missing values on those covariates, we willquery the respective RCT teams to find and complete the missing records from the primary data sources. In cases when these data cannot be recovered the respective median or mode values will be used to replace the missing values on these covariates. Some of the secondary and exploratory analyses thatwould be conducted, might encounter significant missingness. Those secondary and exploratory investigations will develop separate plans for addressing the problem of missing data.

## Interim Analysis and Data

## MonitoringScope of Interim

## Analysis

Continuous monitoring, using Bayesian stopping rules allows for real-time decisions without the penalties for multiple data looks and alpha-spending associated with the classic RCT monitoring approach. At each interim analysis, the posterior distribution of the parameter describing the treatment effect will be reported (graphically and numerically) and the prespecified stopping criteria will guide the recommendations of the cDSMB. The Bayesian monitoring approach enables straight-forward, actionable rules for efficacy which can incorporate information accrued across all studies. The process involves estimation of the posterior probability of a favorable odds ratio for the two primary outcomes, and the stopping rules will be based on this posterior probability exceeding a pre-specified threshold.

## Interim Analysis Results and the Collective Data Safety Monitoring Board

All interim analyses will be conducted by an unblinded biostatistician from Erasmus MC. When one orboth of the stopping rules are reached the following actions are taken;

1. If not already done, the analysis will first be verified or confirmed by a second unblinded CoV-Early statistician. The results of the analysis will be shared with the unblinded statistician from the COn-Vert study team (as well as the unblinded statistician from any other study team thatmay have joined the COMPILEhome consortium at that time).

2. If the results of the interim analysis are confirmed by all unblinded statisticians, the

unblindedErasmus MC statistician will inform the DSMB of COMPILE$_{home}$ (cDSMB) and the cDSMB will be asked to formulate a recommendation regarding future enrollment in COMPILE$_{home}$. This recommendation is forwarded to the chair of the COMPILE$_{home}$ steering committee. This chairwill inform the other steering committee members about the advice that the cDSMB formulated.

3. The PI of each of the individual study teams that form the COMPILE$_{home}$ consortium will informtheir DSMB about the fact that a stopping rule was reached in the COMPILE$_{home}$ analysis and ask for their advice on continuing or stopping the Cov-Early, respectively COn-Vert study (andany other study team that may have joined the COMPILE$_{home}$ consortium at that time).

4. The PI of the COn-Vert and CoV-Early (and any other study team that may have joined the COMPILE$_{home}$ consortium at that time) will inform the COMPILE$_{home}$ steering committee members about the advice that each of the individual DSMBs formulated regarding stopping or continuing the individual trials. The PI of each of the individual study teams will inform theCOMPILE$_{home}$ steering committee if this DSMB advice will be followed or not.

Please note that, when on a certain date and time the decision is made to stop the pooling in the COMPILE$_{home}$ database, there may still be a substantial number of patients in the study of which the follow-up is not yet complete. The totality of the accumulating trial data of these patients will be included in the final study report. This means that the probabilities and or p-values as well as effect sizes in the final report may differ slightly from what was observed at the time of the interim analysis that led to the COMPILE$_{home}$ cDSMB advice to stop the COMPILE$_{home}$ study.

When, after discussion with their individual DSMB, one or more of the individual studies decides to continue enrollment, the COMPILE$_{home}$ team will, to the extent possible, include additional data that are generated in the continuing individual studies up until the COMPILE$_{home}$ study report and paper isbeing finalized.

## Stopping Rules

The analysis of the primary outcomes: worst disease severity score on a 5-point ordinal scale at day 28 after transfusion and hospitalization rate at day 28 (a ≥ 3 score on the 5-point ordinal scale) after transfusion will be based on the following models 1) a proportional odds (PO) model for the first component and 2) a logistic regression model for the second component. In each model , the estimated log odds will be modeled as a function of a CP treatment indicator, the covariates (see section 5.3), and two random effects for RCTs. We denote the parameters of interest (the overall treatment effects) in the PO model using $\Delta_{PO}$ and $\Delta_l$.Details about the analytic model and the initial priors are found in Section 5.6.

We propose considerations for stopping the study based on the following posterior probabilities for the odds ratios ($OR_{PO} = e^{\Delta PO}$ and $OR_l = e^{\Delta l}$)

**Stopping for efficacy:**

$P(OR_{\mathrm{PO}} < 1) \geq 0.985$ and $P(OR_l < 1) \geq 0.80$

OR

$P(OR_l < 1) \geq 0.975$

**Primary Efficacy Analysis**

**Worst disease severity score on a 5-point ordinal scale at day 28 after transfusion**

Let $i$ denote the i-th patient included in any of $K$ trials. Let $x_i$ be a vector of baseline variables, such as sex, age and BMI. Any numerical baseline variables will be standardized to have mean zero and standard deviation 1. We have the following additional predictors:

- $trt_i$ is a dummy with 0 if patient $i$ on control, and 1 if CP.
- $trial\_1_i, \ldots, trial\_K_i$ are dummies with $trial\_K_i = 1$ if patient $i$ is in trial $k$ ($k = 1, 2, \ldots, K$). $trt\_trial\_1_i, \ldots, trt\_trial\_K_i$ are dummies with $trt\_trial\_K_i = 1$ if patient $i$ is included in trial $k$ ($k = 1, 2, \ldots, K$) and treated with CP. Note that $trt\_trial\_k_i = trt_i \times trial\_k_i$.

Let $p_i = (p_{i1}, \ldots, p_{i5})$ denote the probabilities that patient $i$ experiences either of the 5 (ordinal) outcome categories. We will assume a priori that conditionally on $trt_i = 0$, $trial\_1_i = 1$ and all baseline variables at their reference values, the vector $p_i$ follows the Dirichlet distribution with concentration parameter $\alpha = (1;\ 1;\ 1;\ 1;\ 1)$.

The effects of all predictors (including the main treatment effect $trt$) appear as a log oddsratios in a proportional odds model. They have a priori normal distributions with mean zero and the following standard deviations:

- For the effects of baseline covariates (centered and standardized) we will assume standard deviations of 0.5.
- For the main treatment effect $trt$ we will assume a skeptical standard deviation of 0.4.
- For the $trial$ effects $trial\_2_i, \ldots, trial\_K_i$ we will assume standard deviations of 0.5
- For the trial-by-treatment interaction effects $trt\_trial\_1_i, \ldots, trt\_trial\_K_i$ we will assume standard deviations of 0.14

Note that the prior on the trial-specific treatment effects ($= trt + trt\_trial\_k$) is normal with mean zero and standard deviation $\sqrt{0.4^2 + 0.14^2} = 0.42$.

If we have $K = 2$ trials, the model stated above can be fitted with the function brm() in theR package **brms** as follows:

```
brm(y ~ trt + trial_2 + trt_trial_1 + trt_trial_2 + sex + age + bmi +
…,family = cumulative("logit"),
prior = c(
set_prior("normal(0,0.4)",class="b",coef="trt"),
set_prior("normal(0,0.14)",class="b",coef="trt_trial_1"),
set_prior("normal(0,0.14)",class="b",coef="trt_trial_2"), set_prior("normal(0,0.5)",class="b")
),
chains = 4,warmup=100,iter=5000,
include=FALSE,pars=c("disc"),refresh=0))
```

**Hospitalization Rate**

The analysis of the second primary outcome will be a Bayesian logistic regression model with similar prior specification as for the primary outcome.

**Secondary efficacy Analyses**

For the following secondary efficacy analyses we will work under the frequentist framework. When using regression models, we will adjust for the following covariates, when possible given the number of observed events and the number of observations per category in categorical covariates: age, sex, O2 saturation at inclusion, immunocompromised or not, number of comorbidities and time since symptoms onset (as a continuous variable). However, in case of very low event counts and/or low number of observations per category, some of these covariates may have to be removed from the model. Also, in each regression model trial effects ($trial\_1i, \dots, trial\_Ki$) will be added as a fixed-effect in the model as dummy variables which take the value 1 if patient $i$ is in trial $k$ and 0 otherwise.

1. Time to full symptom resolution in intervention versus control arm up to 28 days of follow-up will be assessed by presenting the respective Kaplan Meier curves, excluding subjects who died during follow-up. A log-rank test will be used to compare the Kaplan Meier curves complemented by a Cox proportional hazards regression model including the covariates listed above.

2. Safety of CP in outpatients with COVID-19 using descriptive statistics and tables. Serious adverse events will be presented according to 3 categories: death, life threatening transfusion reactions, (prolongation of) hospitalization.

**Subgroup Analyses**

**The following will be evaluated in subgroups of patients:**

1. Efficacy according to 5 point ordinal scale in those with days from symptom onset (DFSO) of 1 to 5 days. For this, a proportional odds model will be used, along with the covariates listed above, the main effect of treatment an interaction term between treatment and DFSO will be included to capture differences in the effect of treatment between subjects with 1 to 5 days since first symptoms onset and more than 5. From this model an estimate will be made of the interaction effect between treatment

and days since first symptoms onset (odds ratio). The odds ratio along with 95% confidence interval will be presented and the estimate will be assessed using the Wald test statistic at the alpha level of 5%. Absolute percentages will also be reported.

2. Efficacy according to binary outcome of hospital admission or death in those with DFSO of 1 to 5 days . For this, a logistic regression model will be used along the covariates listed above, the main effect of treatment, an interaction term between treatment and days since first symptoms onset will be included to capture differences in the effect of treatment between subjects with 1 to 5 days since first symptoms onset and more than 5. From this model an estimate will be made of the interaction effect between treatment and days since first symptoms onset (odds ratio). The odds ratio along with 95% confidence intervals will be presented and the estimate will be assessed using the Wald test statistic at the alpha level of 5%. Absolute percentages will also be reported.

3. Efficacy according to the 5-point ordinal scale in SARS-CoV-2 in relation to height of neutralizing antibody titers in transfused plasma. For this, a proportional odds model will be used, along with covariates listed above, the main effect of treatment, and an interaction term between treatment and the height of neutralizing antibody titers in transfused plasma will be included to capture differences in the effect of treatment per unit change in height of neutralizing antibody titers in transfused plasma. From this model an estimate will be made of the interaction effect between treatment and the height of neutralizing antibody titers in transfused plasma (odds ratio). The odds ratio along with 95% confidence intervals will be presented and the estimate will be assessed using the Wald test statistic at the alpha level of 5%. Absolute percentages for the first and third quantiles will also be calculated and reported, using model-based estimates .

4. Efficacy according to the binary outcome of hospital admission or death in SARS-CoV-2 in relation to height of neutralizing antibody titers in transfused plasma. For this, a logistic regression model will be used, along with the covariates mentioned above, a main effect of treatment and an interaction term between treatment and the height of neutralizing antibody titers in transfused plasma will be included to capture differences in the effect of treatment per unit change in height of neutralizing antibody titers in transfused plasma. From this model an estimate will be made of the interaction effect between treatment and the height of neutralizing antibody titers in transfused plasma (odds ratio). The odds ratio along with 95% confidence interval will be presented and the estimate will be assessed using the Wald test statistic at the alpha level of 5%. Absolute percentages of the first and third quantiles will also be calculated and reported, using model-based estimates.

5. Efficacy according to the 5-point ordinal scale in SARS-CoV-2 antibody negative patients. For this, a proportional odds model adjusted for the covariates listed above Along with the main effect of treatment, an interaction term between treatment and antibody negative vs positive will be included to capture differences between antibody negative patients and antibody positive patients. From this model an estimate will be made of the interaction effect between treatment and the antibody status (odds ratio). The odds ratio along with 95% confidence interval will be presented and the estimate will be assessed using the Wald test statistic at the alpha level of 5%. Absolute percentages will also be reported.

6. Efficacy according to the binary outcome of hospital admission or death in SARS-CoV-2

antibody negative patients a logistic regression adjusted for the covariates listed aboveAlong with the main effect of treatment, an interaction term between treatment and antibody negative vs positive will be included to capture differences between antibody negative patients and antibody positive patients. From this model an estimate will be made of the interaction effect between treatment and antibody status (odds ratio). The odds ratio along with 95% confidence interval will be presented and the estimate will be assessed using the Wald test statistic at the alpha level of 5%. Absolute percentages will also be reported.

This supplement on the **COnV-ert** protocol contains the following items:

1. Protocol: Original (version 4.0 28th October 2020) and last amendment (protocol version 5.0, 10th December 2020) with a summary of changes
- Page 2 COnV-ert Original protocol (version 4.0, 28th October 2020)
- Page 74 COnV-ert final protocol (version 5.0, 10th December 2020)
- Page 148 Summary of changes

2. Statistical analysis plan: The final SAP (version 1.0, 25th August 2020)
- P162 Summary of changes

# Convalescent Methylene Blue Treated (MBT) Plasma for Early Treatment in Non-hospitalised Mild or Moderate COVID-19 Patients: a Randomized Double Blind Study (COnV-ert)

**Version 4.0, 28th October 2020**

**Sponsors:**

*Fundació FLS de Lluita contra la Sida, les Malalties Infeccioses i la Promoció de la Salut i La Ciència*
Hospital Universitari Germans Trias i Pujol
Carretera de Canyet s/n
08916 Badalona (Barcelona)

*Hospital Germans Trias i Pujol, Institut Català de la Salut*
Carretera de Canyet s/n
08916 Badalona (Barcelona)

**Coordinating Investigator:**

Oriol Mitjà, MD, PhD
Hospital Universitari Germans Trias i Pujol

The information contained in this document is confidential and must not be revealed to third persons without prior authorization as contemplated by Law.

This study will be conducted under the conditions described in this protocol and in compliance with the International Council for Harmonisation of Technical Requirements for Pharmaceuticals for Human Use (ICH) Good Clinical Practice (GCP) and all applicable regulatory requirements.

**SIGNATURES**

The Coordinating Investigator and the Sponsors of the study:

"Convalescent Methylene Blue Treated (MBT) Plasma for Early Treatment in Non-hospitalised mild or moderate COVID-19 Patients: a Randomized Double Blind Study (COnV-ert)"

Declare that this study will be conducted in compliance with the protocol, Good Clinical Practices (GCP) published by the International Conference of Harmonization Guideline (ICH), and the applicable regulatory requirements.

Modifications to this protocol must be submitted prior agreement of the Principal Investigator and Sponsor.

**Coordinating Investigator:** Oriol Mitjà, MD, PhD

Signature and Date: ____________________________

**Sponsor:** Bonaventura Clotet, MD, PhD

President of the Fundació FLS de Lluita contra la Sida, les Malalties Infeccioses i la Promoció de la Salut i La Ciència

Signature and Date: ____________________________

**Sponsor:** Jordi Ara, MD, PhD

Managing Director of the Àrea Metropolitana Nord, Hospital Germans Trias i Pujol, Institut Català de la Salut

Signature and Date: ____________________________

# 1 GENERAL INFORMATION

## 1.1 Title

Convalescent Methylene Blue Treated (MBT) Plasma for Early Treatment in Non-hospitalised Mild or Moderate COVID-19 Patients: a Randomized Double Blind Study (COnV-ert)

## 1.2 Code

COnV-ert

## 1.3 Protocol Version and Date

Version 4.0, 28th October 2020

Any modification of the protocol must also bear the amendment number and date.

## 1.4 Sponsors

This study will be sponsored by two different institutions:

- Fundació FLS de Lluita contra la Sida, les Malalties Infeccioses i la Promoció de la Salut i La Ciència
  Hospital Universitari Germans Trias i Pujol
  Carretera de Canyet s/n
  08916 Badalona (Barcelona)

  Person authorized by the sponsor to sign the protocol and amendments: Bonaventura Clotet, President

- Hospital Germans Trias i Pujol, Institut Català de la Salut
  Carretera de Canyet s/n
  08916 Badalona (Barcelona)

  Person authorized by the sponsor to sign the protocol and amendments: Jordi Ara, Managing Director

## 1.5 Coordinating Investigator

Oriol Mitjà, MD, PhD
Fundació FLS de Lluita contra la Sida, les Malalties Infeccioses i la Promoció de la Salut i La Ciència
Hospital Universitari Germans Trias i Pujol
Carretera de Canyet s/n
08916 Badalona (Barcelona)
omitja@flsida.org

## 1.6 Clinical Research Organization

The study monitoring, regulatory submission to EC and other study tasks will be performed by the following Clinical Research Organization (CRO):

FLS-Research Support
Fundació FLS de Lluita contra la Sida, les Malalties Infeccioses i la Promoció de la Salut i La Ciència

Hospital Universitari Germans Trias i Pujol
Carretera de Canyet s/n
08916 Badalona
Phone +34 93 497 84 14
info@fls-rs.com

## 1.7 Sites and Investigators

This is a multiple site study. The list of investigators and participating sites may be found in **ANNEX 8**.

## 1.8 Technical Services and Institutions Involved

Blood inflammatory markers tests will be assessed at the central laboratories of Hospital Universitari Germans Trias i Pujol.

ABO compatibility test will be assessed at the Banc de Sang i Teixits (BST).

Quantitative measurement of SARS-CoV-2 viral load will be assessed at the Clinical genetics Service of the Hospital Universitari Germans Trias i Pujol (principal investigator: Ignasi Blanco).

Quantitative measurement of neutralizing antibodies against SARS-CoV-2 in convalescent MBT plasma and in participant's plasma will be assessed at the IrsiCaixa laboratory located in Hospital Universitari Germans Trias i Pujol, by the "Cell virology and immunology" group (principal investigator: Julià Blanco).

Inclusion/baseline hospital visits will be organized in partnership with the "Hospital at home care unit" (*Hospitalització domiciliària*).

Electronic case report form (eCRF), data management and statistics will be performed by BioClever 2005 S.L.U., (contact person: Mireia Bonet, T. 93 408 63 88).

Information regarding additional key personnel and organizations involved in the conduct of the study, including names and contact details of participating investigators, monitors, clinical laboratories, technical departments and/or institutions, as well as information on members of additional study committees, will be found in the study files of the sponsor and at the investigator sites.

Investigators and study staff will receive training in appropriate individual site training session(s).

## TABLE OF CONTENTS

**1 GENERAL INFORMATION** **3**
*1.1 TITLE* *3*
*1.2 CODE* *3*
*1.3 PROTOCOL VERSION AND DATE* *3*
*1.4 SPONSORS* *3*
*1.5 COORDINATING INVESTIGATOR* *3*
*1.6 CLINICAL RESEARCH ORGANIZATION* *3*
*1.7 SITES AND INVESTIGATORS* *4*
*1.8 TECHNICAL SERVICES AND INSTITUTIONS INVOLVED* *4*
**TABLE OF CONTENTS** **5**
**STUDY SUMMARY** **10**
**ABBREVIATIONS** **17**
**2 BACKGROUND INFORMATION** **19**
*2.1 CONVALESCENT PLASMA* *19*
2.1.1 The use of Convalescent Plasma as treatment in infectious diseases 19
2.1.2 Name and Description of the Investigational Plasma 19
2.1.3 Relevant Findings from Nonclinical and Clinical Trials 19
2.1.4 Safety 20
2.1.5 Justification for the Route of Administration, Dosage, Dosage Regimen, and Treatment Periods 20
*2.2 MONITORING EFFICACY OF TREATMENT* *21*
2.2.1 Virologic Outcomes 21
2.2.2 Clinical Outcomes 21
2.2.3 Inflammatory Markers 22
2.2.4 Quantification of Neutralizing Antibodies Against SARS-Cov-2 22
2.2.5 Sample Collection Method 23
*2.3 HYPOTHESES* *23*
**3 STUDY OBJECTIVES** **25**
*3.1 PRIMARY OBJECTIVES* *25*
*3.2 SECONDARY OBJECTIVES* *25*
**4 TRIAL DESIGN** **26**
*4.1 TYPE OF TRIAL* *26*
*4.2 DESCRIPTION OF THE DESIGN* *26*

*4.3 STUDY ENDPOINTS* *27*
4.3.1 Primary endpoint(s) 27
4.3.2 Secondary endpoints 28
*4.4 MEASURES TO AVOID BIAS* *28*
4.4.1 Randomization 28
4.4.2 Stratification 28
4.4.3 Blinding 28
4.4.4 Unblinding 29
4.4.5 Identification of Subjects 29
*4.5 END OF STUDY* *29*
*4.6 SOURCE DATA* *30*
**5 STUDY INVESTIGATIONAL PRODUCTS** **31**
*5.1 EXPERIMENTAL AND CONTROL INVESTIGATIONAL PRODUCTS* *31*
*5.2 ARM DESCRIPTION* *31*
*5.3 SUPPLY, PACKAGING, LABELLING AND STORAGE* *31*
5.3.1 Supply 31
5.3.2 Packaging and Labelling 31
*5.4 DOSE, INTERVAL, ROUTE AND METHOD OF ADMINISTRATION* *32*
5.4.1 Dose, Interval, and Route 32
5.4.2 Administration and Timing of Investigational Plasma for Each Subject 32
*5.5 MODIFICATION OF THE TREATMENT REGIMEN* *32*
*5.6 PRIOR AND CONCOMITANT TREATMENTS* *32*
*5.7 ACCOUNTABILITY PROCEDURES FOR INVESTIGATIONAL PRODUCT* *32*
*5.8 TREATMENT COMPLIANCE* *33*
**6 SELECTION AND WITHDRAWAL OF SUBJECTS** **34**
*6.1 STUDY POPULATION* *34*
*6.2 INCLUSION CRITERIA* *34*
*6.3 EXCLUSION CRITERIA* *34*
*6.4 SUBJECT WITHDRAWAL CRITERIA* *35*
6.4.1 Early Subject Withdrawal 35
6.4.2 Medical Approach to Withdrawal 35
6.4.3 Follow-up after Early Withdrawal 35
6.4.4 Replacement of Participants 35
6.4.5 Pre-randomization Loses 35

**7 STUDY CONDUCT AND ASSESSMENT OF RESPONSE 36**
*7.1 STUDY VISITS AND PROCEDURES 36*
7.1.1 Recruitment procedures 36
7.1.2 Screening 36
7.1.3 Inclusion/baseline 36
7.1.4 Day 3 38
7.1.5 Day 7 38
7.1.6 Day 14 38
7.1.7 Day 28 38
7.1.8 Day 60 (End-of-Study) 39
7.1.9 Extra home visit (unscheduled visit) 39
7.1.10 Participant's Diary 39
*7.2 CLINICAL PROGRESSION SCALES 39*
7.2.1 WHO Clinical Progression Scale 39
7.2.2 FLU-PRO® PLUS Questionnaire 39
7.2.3 Assessment of Safety 40
*7.3 DESCRIPTION OF LABORATORY TESTS AND PROCEDURES 41*
*7.4 STUDY VARIABLES 42*
*7.5 SCHEDULE OF PROCEDURES 45*
**8 ADVERSE EVENTS AND INCIDENTS 46**
*8.1 SAFETY PARAMETERS 46*
*8.2 DEFINITIONS 46*
*8.3 ADVERSE EVENTS ASSESSMENT 46*
8.3.1 Seriousness 46
8.3.2 Intensity 47
8.3.3 Causality 47
8.3.4 Expectedness 48
8.3.5 Duration 48
8.3.6 Action Taken 48
8.3.7 Outcome 48
*8.4 TIME FRAME FOR RECORDING ADVERSE EVENTS AND INCIDENTS 49*
*8.5 DOCUMENTATION RELATED TO ADVERSE EVENTS AND INCIDENTS 49*
*8.6 PREGNANCY 49*
*8.7 PROCEDURE FOR ADVERSE EVENT REPORTING 49*

8.7.1 Investigator 49
8.7.2 Sponsor 50
*8.8 DATA AND SAFETY MONITORING BOARD 50*
**9 STATISTICAL ANALYSIS 51**
*9.1 METHODS 51*
9.1.1 Descriptive Analysis 51
9.1.2 Bivariate Analysis 51
9.1.3 Multivariate Analysis 51
*9.2 ANALYSIS POPULATION 51*
*9.3 SAMPLE SIZE 52*
*9.4 INTERIM ANALYSES 52*
*9.5 DEVIATION OF STATISTICAL PLAN 52*
**10 DIRECT ACCESS TO SOURCE DATA/DOCUMENTS 53**
**11 QUALITY CONTROL AND QUALITY ASSURANCE 54**
*11.1 STUDY MONITORING 54*
*11.2 PROTOCOL DEVIATIONS 54*
*11.3 AUDITS AND INSPECTIONS 54*
*11.4 DATA COLLECTION (ELECTRONIC CASE REPORT FORM, ECRF) 55*
*11.5 DATA MANAGEMENT 55*
**12 ETHICS 57**
*12.1 GENERAL CONSIDERATIONS 57*
*12.2 PARTICIPANT INFORMATION SHEET AND INFORMED CONSENT 57*
**13 DATA HANDLING AND RECORD KEEPING 58**
*13.1 DATA HANDLING 58*
*13.2 RECORD KEEPING 58*
13.2.1 Investigator File and Document Retention 58
13.2.2 Source Documents and Basic Data 58
**14 FINANCING AND INSURANCE 59**
*14.1 SOURCE OF FINANCING 59*
*14.2 INSURANCE POLICY 59*
**15 PUBLICATION POLICY 60**
**16 REFERENCES 61**
**17 ANNEXES 63**
**ANNEX 1: DONOR SELECTION, COLLECTION AND PROCESSING PROCEDURES FOR CONVALESCENT MBT PLASMA PREPARATION FROM DONORS RECOVERED FROM COVID-19 63**

**ANNEX 1.1.: CRITERIA FOR ACCEPTING / EXCLUDING DONORS OF WHOLE BLOOD AND BLOOD COMPONENTS 63**

**ANNEX 1.2: UPDATE OF THE PROTOCOL FOR THE SELECTION OF COVID-19 CONVALESCENT PLASMA DONORS 63**

**ANNEX 1.3.: REGISTER OF THE ASSESSMENT OF COVID-19 CONVALESCENT PLASMA DONOR 63**

**ANNEX 1.4.: CONVALESCENT LABEL TEMPLATE E9744 63**

**ANNEX 1.5.: METHYLENE BLUE INACTIVATION PROCESS 63**

**ANNEX 2: WHO CLINICAL PROGRESSION SCALE 64**

**ANNEX 3: FLU-PRO© PLUS QUESTIONNAIRE 65**

**ANNEX 4: ASSESSMENT OF SAFETY 67**

**ANNEX 5: HEMOVIGILANCE: DETECTION, ASSESSEMENT AND REGISTER OF INCIDENTS RELATED TO TRANSFUSION 69**

**ANNEX 6: WEB CONTENT 70**

**ANNEX 7: COMPOSITION, RESPONSIBILITIES AND FLOW CHART FOR DSMB 71**

**ANNEX 8: LIST OF PARTICIPATING SITES 72**

**ANNEX 9: PARTICIPANT ID CARD 73**

## STUDY SUMMARY

| |
|---|
| **Title of Study:** Convalescent Methylene Blue Treated (MBT) Plasma for Early Treatment in Non-hospitalised Mild or Moderate COVID-19 Patients: a Randomized Double Blind Study (COnV-ert) |
| **Study code:** COnV-ert |
| **Study Objectives:**<br>Primary Objectives<br>1. Assess the therapeutic potential of early administration of convalescent MBT plasma in reducing the rate of hospitalization at day 28 in non-hospitalised mild or moderate COVID-19 patients.<br>2. Assess the therapeutic potential of early administration of convalescent MBT plasma in reducing SARS-CoV-2 viral load at day 7, measured by quantitative RT-PCR (RT-qPCR) in non-hospitalised mild or moderate COVID-19 patients.<br><br>Secondary Objectives<br>1. Assess the therapeutic potential of early administration of convalescent MBT plasma in reducing WHO Clinical progression scale score in non-hospitalised mild or moderate COVID-19 patients.<br>2. Assess the therapeutic potential of early administration of convalescent MBT plasma in reducing the severity of common COVID-19 symptoms, measured with the FLU-PRO© PLUS scale, in non-hospitalised mild or moderate COVID-19 patients.<br>3. Assess the therapeutic potential of early administration of convalescent MBT plasma in reducing the duration of symptoms in non-hospitalised mild or moderate COVID-19 patients.<br>4. Assess the therapeutic potential of early administration of convalescent MBT plasma in reducing the mortality at day 60 in non-hospitalised mild or moderate COVID-19 patients.<br>5. Evaluate the safety and tolerability of convalescent MBT plasma in non-hospitalised mild or moderate COVID-19 patients.<br>6. Assess the change from baseline to day 7 of ferritin, prealbumin, interleukin 6 (IL-6), D-dimer, C reactive protein (CRP), and leukocytes and lymphocytes counts in peripheral blood in non-hospitalised mild or moderate COVID-19 patients receiving convalescent MBT plasma.<br>7. Assess the impact of infused plasma on neutralizing activity by quantifying the change from baseline to day 7 in neutralizing antibodies against SARS-CoV-2 in peripheral blood in non-hospitalised mild or moderate COVID-19 patients receiving convalescent MBT plasma.<br>8. Assess the long-term impact of plasma infusion on humoral immune responses, by quantifying the change from baseline to day 60 in neutralizing antibodies against SARS-CoV-2 in peripheral blood in non-hospitalised mild or moderate COVID-19 patients receiving convalescent MBT plasma.<br>9. Compare agreement and SARS-CoV-2 viral load in self-collected middle turbinate (MT) swab and self-collected saliva samples with nasopharyngeal swab samples collected by a healthcare worker. |

10. Assess the therapeutic potential of early administration of convalescent MBT plasma in reducing SARS-CoV-2 viral load at day 28, measured by quantitative RT-PCR (RT-qPCR) in non-hospitalised mild or moderate COVID-19 patients.

**Overall Study Design and Description:**

This is a prospective, randomized (1:1), double blind study of Convalescent anti-SARS-CoV-2 MBT Plasma (also known as convalescent plasma) plus standard medical treatment (SMT) versus placebo plus SMT in mild or moderate COVID-19 patients who are non-hospitalised. Subjects with confirmed infection by SARS-CoV-2 will receive SMT plus a total of 200-250 mL of convalescent plasma that has been pathogen-inactivated using MBT or placebo.

Study candidates will voluntarily express their interest in participating in the study through the study website or will be offered to participate at the emergency (ER) and out-patient departments (OPD) of the participating hospitals. Candidates registered on the website will be contacted by study physicians by phone to inform them about the study and check their suitability for the study. Suitable candidates will be scheduled an inclusion/baseline visit in which informed consent will be obtained (i.e., the informed consent will be signed), and their eligibility will be confirmed. Candidates identified through ER and OPD departments will undergo an inclusion/baseline visit, where the informed consent will be obtained and eligibility will be checked. A subgroup of eligible candidates from selected study sites will be offered participation in the substudy to assess the immune response and the methods of sampling.

Blood and nasopharyngeal samples will be obtained from all eligible candidates.

Eligible candidates will be randomized and administered an intravenous (IV) infusion at baseline (convalescent plasma or placebo). Both the investigator and the participant will be blinded to the study treatment.

Specifically, subjects randomized to combination convalescent anti-SARS-CoV-2 MBT plasma plus SMT will undergo an ABO compatibility test and will receive a single infusion of 200 to 250 ml of ABO-compatible convalescent plasma. Subjects randomized to placebo plus SMT will receive a single infusion of 200 to 250 ml of sterile saline solution 0.9%. Infusion will be administered at baseline, using standard procedures for administration of fresh frozen plasma. Small adults weighing less than 45 kg will receive one infusion of 5 ml of convalescent plasma or placebo per kilogram of body weight.

Participants will be trained on the completion of symptoms diary card and safety diary card.

The participants of the substudy will be drawn an extra tube of blood sample and will be trained on self-collection of middle turbinate (MT) swabs and saliva, and self-collected samples will be obtained.

The symptoms and safety diary card will be filled by the participants daily from baseline to day 14. On follow-up visits on days 3, 7, 14, and 28, all participants will be assessed for clinical and safety outcomes. These visits will be all by telephone except for the day 7 and day 28 visits that will be at home and at hospital, respectively, where additionally blood samples (only on day 7) and nasopharyngeal swabs will be collected.

At day 60 visit, all participants will be assessed by telephone for health-status outcome.

For the participants of the substudy, on day 7, an extra tube of blood sample will be obtained and they will be asked to self-collect MT swabs and saliva. And on day 60, an extra tube of blood sample will be obtained during an additional home or hospital visit.

**Number of Subjects Planned:**

Approximately 474 individuals will be randomized (1:1) with an interim analysis after the first 60 subjects (30 in each arm).

The sample size will be re-assessed upon interim analysis.

Approximately 135 individuals from selected study sites will be included in the substudy to assess the immune response and the methods of sampling.

**Diagnosis and Main Criteria for Eligibility:**

Individuals with a SARS-CoV-2 confirmed infection from *Metropolitana Sud, Metropolitana Nord* and *Barcelona* areas.

Inclusion Criteria:

1. Adult male or female individuals of ≥50 years old.
2. In women of childbearing potential[1], negative pregnancy test at inclusion/baseline.
3. Has confirmed SARS-CoV-2 infection as determined by PCR or validated antigen rapid diagnostic test[2] from nasopharyngeal swabs ≤5 days prior to inclusion/baseline visit.
4. Symptomatic with mild or moderate COVID-19 with symptoms onset date ≤ 7 days prior to inclusion/baseline visit.
    a. Mild COVID-19: Individuals who have any of the common signs and/or symptoms of COVID-19 (i.e., fever, cough, sore throat, malaise, headache, muscle pain) without shortness of breath, dyspnoea, or abnormal chest imaging.
    b. Moderate COVID-19: Individuals who have evidence of lower respiratory disease by clinical assessment or imaging and a saturation of oxygen ($SpO_2$) ≥94% on room air at sea level.
5. Willing to comply with the requirements of the protocol and available for follow-up for the planned duration of the study.
6. Has understood the information provided and capable of giving informed consent.

*[1]A woman will be considered of childbearing potential if not permanently sterilized nor postmenopausal. Permanent sterilization methods include tubal ligation, hysterectomy and bilateral oophorectomy. Postmenopausal is defined as 12 months with no menses without an alternative medical cause.*

*[2]Panbio™ COVID-19 Ag Rapid Test (Abbott), STANDARD™ Q COVID-19 Ag Test (Roche) or any other CE marketed test for SARS-CoV-2 Ag detection.*

**Diagnosis and Main Criteria for Eligibility:**

Exclusion Criteria:

1. If female, pregnant, breastfeeding, or planning a pregnancy during the study.
2. Severe or critical COVID-19:
    a. Severe COVID-19: respiratory frequency >30 breaths per minute, $SpO_2$ <94% on room air at sea level, ratio of arterial partial pressure of oxygen to fraction of inspired oxygen ($PaO_2/FiO_2$) <300 mmHg, or lung infiltrates >50%.
    b. Critical COVID-19: respiratory failure, septic shock, and/or multiple organ dysfunction.

3. Current hospital admission for any cause.
4. History of previous confirmed SARS-CoV-2 infection.
5. History of significantly abnormal liver function (Child Pugh C).
6. History of chronic kidney disease (CKD) ≥ stage 4, or need of dialysis treatment.
7. Any pre-existing condition that increases risk of thrombosis.
8. History of allergic reactions to blood or plasma products or methylene blue.
9. Known IgA deficiency with anti-IgA antibodies.
10. Medical conditions for which 250 ml of intravenous fluid is considered dangerous (i.e., decompensated heart failure or renal failure with fluid overload).
11. Inability to consent and/or comply with study requirements, in the opinion of the investigator.
12. Currently participating or planning to participate in any interventional study for the treatment of COVID-19 or SARS-CoV-2 infection until day 60.

**Investigational Product, Dose and Mode of Administration:**

Randomized participants will receive one intravenous (IV) infusion at baseline (convalescent plasma or placebo) and will continue their SMT for COVID-19 disease, as prescribed by regular physicians.

- Experimental arm: Subjects randomized to convalescent anti-SARS-CoV-2 MBT plasma plus SMT will receive one infusion of 200 to 250 ml of ABO-compatible convalescent plasma obtained from a convalescent donor.
- Placebo arm: Subjects randomized to placebo plus SMT will receive one infusion of 200 to 250 ml of sterile saline solution 0.9%.

**Duration of Treatment:**

The investigational product will be administered by IV infusion at baseline.

Participants will continue their standard medical treatment (SMT) for SARS-CoV-2 infection as prescribed by their regular physician. If applicable, SMT may be modified during the study, depending on personal requirements, the severity and progression of the disease, and need for hospitalization.

Subjects' participation (from inclusion/baseline visit to the end-of-study visit) will be up to 60 days.

**Endpoints and Timepoints of Assessment:**

Primary Endpoints and Timepoints of Assessment:

1. Hospitalization rate (i.e., who reach a score ≥4 in the WHO scale for clinical progression) [Time Frame: Up to 28 days after reception of investigational product]
   Reduction of SARS-CoV-2 viral load in nasopharyngeal swabs at day 7 after start of treatment, as determined by RT-qPCR. [Time Frame: Up to 7 days after reception of investigational product]

Secondary Endpoints and Timepoints of Assessment:

1. Change in COVID-19 WHO Clinical progression scale score [Time Frame: Up to 60 days after reception of investigational product]

2. Change in COVID-19 symptoms severity score, assessed with the COVID-19 daily self-score tool (FLU-PRO© PLUS instrument) [Time Frame: Up to 14 days after reception of investigational product]
3. Time to complete resolution of symptoms [Time Frame: Up to 28 days after reception of investigational product]
4. Death rate [Time Frame: Up to 60 days after reception of investigational product]
5. Proportion of patients with adverse events (AE) and proportion of grade ≥4 AE, based on the *FDA Toxicity Grading Scale for Healthy Adult and Adolescent Volunteers scale* [Time Frame: Up to 28 days after reception of investigational product]
6. Change in inflammatory prognostic markers (ferritin, prealbumin, interleukin 6 (IL-6), D-dimer, C reactive protein (CRP) and leukocyte and lymphocyte counts) [Time Frame: Baseline and day 7 after reception of investigational product]
7. Intergroup comparison of absolute neutralization titers against SARS-CoV-2 in plasma of a subgroup of participants [Time Frame: Baseline and day 7 after reception of investigational product]
8. Change in titers of neutralizing antibodies against SARS-CoV-2 in plasma of a subgroup of participants [Time Frame: Baseline and day 60 after reception of investigational product]
9. Agreement and SARS-CoV-2 viral load of self-collected middle turbinate (MT) swab and saliva samples compared to nasopharyngeal swabs collected by a healthcare worker on a subgroup of participants [Time Frame: Baseline and day 7 after reception of the investigational product]
10. Reduction of SARS-CoV-2 viral load in nasopharyngeal swabs at day 28 after start of treatment, as determined by RT-qPCR. [Time Frame: Up to 28 days after reception of investigational product].

**Study Assessments and Procedures:**

COVID-19 patients of ≥50 years (1) will voluntarily express their interest in participating in the study through the study website or (2) will be informed of the study at the emergency (ER) or out-patient departments (OPD) from the participating sites.

1. Candidates who register on the study website will sign an authorization for the study investigators (physicians) to access their shared medical records (*Història Clínica Compartida de Catalunya HC3*). Study physicians will call the candidates who register on the website to perform a remote screening visit, where candidates will be informed about the study over the phone, and their suitability for the study will be checked. Suitable candidates will be scheduled a inclusion/baseline visit .
2. Candidates identified at the participating sites will be invited to participate by study physicians on-site, and those interested will undergo an inclusion/baseline visit.

In the inclusion/baseline visit, either at the hospital or at home by the Hospital at Home care unit (*Hospitalització domiciliària*), candidates will be informed in person of study details and the informed consent will be obtained (i.e., subjects will sign the informed consent form). A physical examination will be performed, and women of childbearing potential will undergo a urine pregnancy test. Eligibility will be confirmed on this visit.

Blood samples will be obtained from all eligible candidates. Participants who meet all the inclusion and none of the exclusion criteria will be randomized. Evaluation of ABO compatibility using standard guidelines for compatibility procedures in blood transfusion

laboratories will be performed in participants assigned to experimental (convalescent plasma) group.

Nasopharyngeal samples will be obtained from all participants. The participants of the substudy will be drawn an extra tube of blood sample and will be trained on self-collection of middle turbinate (MT) swabs and saliva, and self-collected samples will be obtained.

Participants will be also trained on completion of symptoms and safety diary card, which will be filled daily by the participant until the day 14 visit, through an electronic Case Report Form (eCRF). Paper forms will be available for those participants who are unable to submit data using the eCRF, in this scenario data will be collected at follow-up visits. Should any participant report any new symptom with grade 4 at the FLU-PRO© PLUS questionnaire, he or she will be contacted by telephone by the study staff to evaluate any possible AE and the need of an extra home visit. Participants will be also given a contact number to report AEs, to seek medical advice, and resolve any question related to the study.

All randomized participants will be administered an IV infusion at baseline (convalescent plasma or placebo). If an AE develops during infusion, the infusion will be slowed or stopped as per investigator's decision. Infusion will be halted if any manifestations of anaphylaxis develop, and it will not be restarted. Medicines for transfusion reactions (e.g. paracetamol, dexchlorpheniramine) may be given as a treatment.

On day 3, participants will be contacted by telephone by study staff for safety and clinical follow-up.

On day 7, participants will be visited at home by study staff, a blood sample will be drawn, and a nasopharyngeal swab will be collected by study staff. The participants of the substudy will be drawn an extra tube of blood sample and will self-collect middle turbinate (MT) swabs and saliva.

On day 14, participants will be contacted by telephone by study staff for safety and clinical follow-up.

On day 28, participants will be visited at hospital by study staff for safety and clinical follow-up, and a nasopharyngeal swab will be collected.

On day 60, participants will be contacted by telephone by study staff to evaluate health status. The participants of the substudy will be scheduled for a hospital or home visit to obtain a blood sample.

If the participant is unreachable at phone follow-up visits, clinical progression, as well as possible AEs will be assessed through medical record (*Història Clínica Compartida, HC3*), in order to assess all the study outcomes.

Study samples

Nasopharyngeal swab samples for quantitative measurement of SARS-CoV-2 viral load by RT-qPCR will be obtained prior to infusion at baseline, on day 7 and on day 28.

MT swab and saliva self-collected samples for quantitative measurement of SARS-CoV-2 viral load by RT-qPCR will be obtained prior to infusion at baseline and at day 7 in the participants of the substudy.

Blood samples will be collected prior to infusion at baseline and at day 7 for all participants. Inflammatory prognostic markers (ferritin, prealbumin, interleukin 6 (IL-6), D-dimer, C

| |
|---|
| reactive protein (CRP), and leukocytes and lymphocytes counts) will be assessed. Leftovers of plasma will be stored for future investigations.<br><br>In the participants of the substudy, an extra tube will be collected at baseline and day 7, and an extra blood draw will be performed at day 60 to assess neutralizing antibodies against SARS-CoV-2. Leftovers of plasma will be stored for future investigations.<br><br>Results of the sample analyses are not necessary for participants to remain in the study, and participants will only be informed of relevant results for their health.<br><br>Samples of the infused convalescent anti-SARS-CoV-2 MBT plasma will be obtained before infusion for quantitative measurement of neutralizing antibodies against SARS-CoV-2. |
| **Statistical Method:**<br><br>Descriptive statistics will include the number of non-missing observation, mean, standard deviation (SD), median, minimum, and maximum values for the continuous/quantitative data or absolute and relative frequency counts and percentages for categorical/qualitative data. All analyses will be done with the R statistical package, version 6.3 or higher under a significance level of 0.05.<br><br>Populations: The primary efficacy analysis will be performed on the intention-to-treat (ITT) population, which will include all randomized participants. If deemed necessary, sensitivity analyses will be performed with the per-protocol (PP) population. Safety will be assessed in the safety population, which will include all participants who receive the investigational product (convalescent plasma or placebo).<br><br>Determination of Sample Size:<br><br>A sample size of 237 cases per arm would provide the trial with 80% power to detect 50% reduction in hospitalization rate at day 28 after starting the treatment, assuming an expected rate of hospitalization of 15%, allowing a 5% of loss to follow-up.<br><br>Approximately 150 cases per arm are required to have 80% power to detect a difference of 0.5 $log_{10}$ in the mean reduction of SARS-CoV-2 viral load at a two-sided significance level of $\alpha = 0.05$, assuming an expected overall standard deviation of 1.5. A 0.5 $log_{10}$ copies/mL difference in reduction was chosen to represent the minimal threshold for a biologically relevant change for our analyses.<br><br>An interim analysis of efficacy and safety variables will be performed after the first 60 subjects achieve the primary endpoint (i.e., day 28) for the purpose of sample size recalculation. The interim analyses will be performed using blinded data, unless otherwise indicated by the DSMB. |

## ABBREVIATIONS

| | |
|---|---|
| ACE2 | angiotensin converting enzyme 2 |
| ADL | Activities daily life |
| AE | Adverse event |
| CI | Confidence interval |
| COVID-19 | Coronavirus disease 2019 |
| Ct | cycle threshold |
| CTCAE | Common Terminology Criteria for Adverse Events |
| ELISA | Enzyme, linked immunosorbent assay |
| ER | Emergency Department |
| EuroQol | Europe Quality of life |
| FDA | Food and Drug Administration |
| FFP | Fresh frozen plasma |
| FLU-PRO | FLU- patient-reported outcome measure |
| GCP | Good clinical practice |
| ICH | International Council for Harmonisation |
| IFA | Indirect Fluorescent Antibody |
| IgA/IgM/IgG | Immunoglobulin A / M / G |
| IL-6 | Interleukin 6 |
| ILI | influenza-like illness |
| ITT | Intention to treat |
| IV | intravenous |
| MBT | Methylene blue treated |
| MERS | Middle East respiratory syndrome |
| ml | millilitre |
| MT | middle turbinate |
| NAT | nucleic acid amplification technology |
| NIH | National Institutes of Health |
| OPD | Out-Patient Department |
| OR | Odds ratio |
| PaO2/FiO2 | arterial partial pressure of oxygen to fraction of inspired oxygen |
| PCR | Polymerase chain reaction |
| PP | Per protocol |

| | |
|---|---|
| qPCR | Quantitative PCR |
| CRP | C-reactive Protein |
| RSV | Respiratory Syncytial Virus |
| RT-PCR | Reverse transcriptase PCR |
| SAE | Serious Adverse Event |
| SARS | Severe acute respiratory syndrome |
| SARS-CoV-2 | Severe acute respiratory syndrome coronavirus 2 |
| SD | Standard Deviation |
| SMT | Standard Medical Treatment |
| Sp02 | Peripheral Oxygen Saturation |
| SUSAR | Serious Unexpected Adverse Reaction |
| TACO | Transfusion-Associated Circulatory Overload |
| TRALI | Transfusion-related acute lung injury |
| UAE | Unexpected adverse event |
| WHO | World Health Organization |

# 2 BACKGROUND INFORMATION

Coronavirus disease 2019 (COVID-19) is a respiratory tract infection caused by a newly emergent coronavirus, severe acute respiratory syndrome coronavirus 2 (SARS-CoV-2), that was first recognized in Wuhan, China, in December 2019. The emergence of COVID-19 has caused a large global outbreak and it is a major public health issue. As of 13 August 2020, data from the World Health Organization (WHO) have shown that more than 20 million confirmed cases have been identified in 216 countries, areas or territories (WHO Interim guidance 13 August 2020) (**1**).

## 2.1 Convalescent Plasma

### *2.1.1 The use of Convalescent Plasma as treatment in infectious diseases*

Currently, there are no FDA-approved drugs for the treatment of COVID-19. Definitive clinical trial data is needed to find safe and effective treatments for COVID-19. Vaccine development is progressing at a rapid pace, but widespread vaccine availability is estimated to be at least six months away. There is an urgent need for effective interventions presently. Administration of SARS-CoV-2 neutralizing antibodies is the only form of immunization available in the absence of vaccines or humanized monoclonal antibodies.

Convalescent plasma therapy has been used to treat patients with infections using plasma collected from recently recovered individuals to transfer passive antibody immunity to those who have recently been infected or have yet to be exposed to the virus. Use of convalescent plasma has been studied in outbreaks of other respiratory infections, including the 2003 SARS-CoV-1 epidemic, the 2009-2010 H1N1 influenza virus pandemic, and the 2012 MERS-CoV epidemic (FDA recommendations) (**2**). Convalescent plasma from donors who have recovered from COVID-19 may contain antibodies to SARS-CoV-2 that might help suppress the virus and modify the inflammatory response. It has been postulated that neutralizing antibodies would prevent SARS-CoV-2 spike protein from attaching to the ACE2 receptor, inhibiting viral entry into the cell (Nguyen et al., 2020) (**3**).

### *2.1.2 Name and Description of the Investigational Plasma*

The investigational plasma in this study is Convalescent anti-severe acute respiratory syndrome coronavirus 2 (SARS-CoV-2) MBT Plasma.

The product is being collected by plasma centres in Spain.

### *2.1.3 Relevant Findings from Nonclinical and Clinical Trials*

There is already clinical trial data supporting the efficacy of convalescent plasma in the treatment of COVID-19 patients. An open-label, multicentre, randomized clinical trial conducted in China that enrolled 103 participants with laboratory-confirmed COVID-19 severe or life-threatening patients showed higher rates of viral clearance at 72 hours in the convalescent plasma group compared to the control group (87.2% vs 37.5%, respectively) (OR 11.39 [95% CI, 3.91-33.18]; $P$<0.001), demonstrating association with antiviral activity of the treatment in patients with COVID-19 (Li et al., 2020) (**4**). A retrospective case-control study evaluating convalescent plasma conducted in Mount Sinai Hospital in New York City showed by day 14 a clinical worsening in 18% of the convalescent plasma patients vs 24% of the control patients ($P$=0.17), and a mortality of 13% of the plasma recipients and 24% of the control patients was observed ($P$=0.04) (Liu et al., 2020) (**5**). Preliminary data from a study from Mayo Clinic that aggregated patient outcome data from randomized clinical trials, matched control, and case-series studies showed that hospitalized COVID-19 patients transfused with convalescent plasma exhibited a 57% reduction in mortality rate (13%) compared to matched-patients receiving standard treatments (25%; OR: 0.43, $P$<0.001) (Joyner et al., 2020) (**6**).

The US FDA has approved the emergency use of convalescent plasma for patients with severe or life-threatening COVID-19 (Tanne, 2020) (**7**). And there are currently more than 100 ongoing studies evaluating convalescent plasma or hyperimmune immunoglobulin, of which approximately 50 are randomized (*https://clinicaltrials.gov/*).

Most clinical studies are investigating the use of convalescent plasma in hospitalized patients with severe or critical disease. However, this therapy may have clinical and virologic benefits in the treatment of mild or moderate disease in non-hospitalised patients, especially if given early in the disease course. Therefore, it is important to study the safety and efficacy of COVID-19 convalescent plasma in non-hospitalised patients in clinical trials.

#### *2.1.4 Safety*

Risks of passive administration of convalescent plasma fall into two categories, known and theoretical. Known risks are those associated with transfer of blood substances, which include inadvertent infection with another infectious disease agent and reactions to plasma constituents. With modern blood banking techniques that screen for blood-borne pathogens and match the blood type of donors and recipients, the risks of inadvertently transferring known infectious agents or triggering transfusion reactions are low. Additionally, MBT reduces risk of any pathogen undetected by donor screening and product testing. The most common adverse events are mild allergic or respiratory events (Piechotta et al., 2020) (**8**). Uncommon (i.e. in <1% of transfusions) but serious risks of convalescent plasma infusion may include transfusion-associated circulatory overload (TACO), transfusion-related acute lung injury (TRALI) and anaphylaxis (Joyner et al., 2020) (NIH Covid-19 treatment guidelines) (**9**, **10**). And this should be a consideration in the risk-benefit assessment.

The theoretical risk is potential for antibody-dependent enhancement (ADE) of infection. ADE can occur in several viral diseases and it is a harmful, exaggerated inflammatory response triggered by antibodies. Previously, ADE has been proposed as an underlying pathogenic mechanism in Dengue haemorrhagic fever (Nguyen et al., 2020) (**3**). For coronaviruses, several mechanisms for ADE have been described, and there is the theoretical concern that antibodies to one type of coronavirus could enhance infection to another viral strain (Wan et al., 2020) (**11**). Since the proposed use of convalescent sera in the COVID-19 epidemic would rely on preparations presumably with high titers of neutralizing antibody against the same virus, SARS2-CoV-2, ADE may be unlikely. Available evidence from the use of convalescent plasma in patients with SARS and MERS (Mair-Jenkins et al., 2015) (**12**) and for the treatment of Covid-19, in the Expanded Access Program (EAP), with data from 20.000 hospitalised patients with COVID-19 (Joyner et al., 2020) (**9**), suggest it is safe. Nevertheless, in convalescent plasma trials, close monitoring to identify any evidence of enhanced infection will be required.

Given that historical and current data on use of convalescent plasma suggest it is safe in coronavirus infection, and the high mortality of COVID-19, particularly in elderly and vulnerable persons, suggests that the benefits of its use in those at high risk for or with early disease outweigh the risks. However, for all cases where convalescent plasma administration is considered, a risk-benefit assessment must be conducted to assess individual variables (Casadevall et al., 2020) (**13**).

#### *2.1.5 Justification for the Route of Administration, Dosage, Dosage Regimen, and Treatment Periods*

Administration of Convalescent Plasma/Placebo

Subjects in the Convalescent anti-SARS-CoV-2 MBT plasma plus SMT arm, will receive intravenous (IV) Convalescent anti-SARS-CoV-2 MBT plasma according to standard hospital infusion practices for fresh frozen plasma (FFP). Subjects in the placebo plus SMT arm will receive sterile saline solution 0.9% by intravenous (IV) infusion.

Justification for Selection of Volume of Administration and time of Convalescent Plasma/Placebo

Administration of Convalescent anti-SARS-CoV-2 MBT plasma in this study is consistent with previous experience with the administration of convalescent plasma for other infectious indications (e.g. Ebola) (van Griensven et al., 2016) (**14**) (Joyner et al., 2020) (**9**).

Randomized subjects will receive SMT plus one infusion of 200 to 250 ml of ABO-compatible convalescent plasma or placebo. Small adults weighing less than 45 kg will receive one infusion of 5 ml of ABO-compatible convalescent plasma or placebo per kilogram of body weight (to a maximum of 250ml).

## 2.2 Monitoring Efficacy of Treatment

The WHO Working Group on the Clinical Characterisation and Management of COVID-19 Infection have developed a minimum set of common outcome measures for studies on COVID-19. The set includes three elements: a measure of viral burden, a measure of patient survival and a measure of patient progression. Study outcomes have been selected based on these recommendations (WHO Working Group on the Clinical Characterisation and Management of COVID-19 infection, 2020) (**15**).

### *2.2.1 Virologic Outcomes*

Viral burden is considered an appropriate core outcome for monitoring efficacy of treatment in COVID-19 patients. To measure viral burden, quantitative RT-PCR (RT-qPCR) to quantify viral copies is the best measure, with threshold cycle values (Ct) during PCR as an alternative. Quantification of viral burden has not proven yet to provide insight into the clinical status of the patient but does provide strong evidence of the presence of the pathogen, and it can be used to measured pathogen burden in response to treatment (WHO Working Group on the Clinical Characterisation and Management of COVID-19 infection, 2020) (**15**).

Viral load is associated with transmission risk and disease severity in other viral illnesses. And, recently, it is been reported SARS-CoV-2 viral load at diagnosis as an independent predictor of mortality in a large hospitalised cohort (n=1145). There was found a significant independent association between viral load and mortality (hazard ratio 1.07 [95% CI, 1.03-1.11], $P$=0.0014), with a 7% increase in hazard for each log transformed copy per ml (Pujadas et al., 2020) (**16**). Viral load in COVID-19 might correlate, not only with mortality, but also with infectivity, disease phenotype and morbidity.

### *2.2.2 Clinical Outcomes*

Clinical progression will be measured using different validated scales.

Firstly, the WHO Clinical Progression Scale will be used and will assess **hospitalization rate** (i.e. who reach a score ≥4), as recommended for non-mortal clinical outcomes (WHO Working Group on the Clinical Characterisation and Management of COVID-19 infection, 2020) (**15**). This is an ordinal scale to measure clinical progression and recovery based on location and supportive measures used within the healthcare system. It provides a measure of illness severity across a range from 0 (not infected) to 10 (death) with data elements that are rapidly obtainable from clinical records.

Considering that our target population is non-hospitalised, ambulatory participants, and the rate of hospitalisation is relatively low, even in high-risk patients, we selected some other clinical progression scales to help assess clinical outcomes.

**Symptom severity** will be measured using a validated scale. Severity over time is been considered a clinically meaningful endpoint, particularly in a disease that exhibits such heterogeneous symptomatology.

Symptom severity will be also assessed with the FLU-PRO© PLUS Questionnaire, a daily self-score tool. The COVID-19 version, adapted from FLU-PRO© instrument, consists of 34 items that are answered daily. The FLU-PRO© is a self-administered patient-reported outcome measure (PRO) to quantify symptom severity in influenza and influenza-like illness and it has been tested and used in studies of influenza, influenza-like illness (ILI), respiratory syncytial virus (RSV), rhinovirus, and enterovirus (Han A et al., 2018) (**17**) (Powers JH et al., 2016) (**18**).

### *2.2.3 Inflammatory Markers*

Biomarkers are quantitative measurements used clinically for many conditions reflecting pathological development. A recent systematic review summarized the following biomarkers with most evidence as predictors for a severe COVID-19 infection (Kermali et al., 2020) (**19**):

| | |
|---|---|
| C-Reactive Protein (CRP) | Increases |
| Serum amyloid A | Increases |
| Interleukin-6 | Increases |
| Lactate Dehydrogenase | Increases |
| Neutrophil-to lymphocyte ratio | Increases |
| Lymphocyte count | Decreases |
| Platelet count | Decreases |
| D-dimer | Increases |
| Cardiac troponin | Increases |
| Renal biomarkers Urea & creatinine | Increases |

### *2.2.4 Quantification of Neutralizing Antibodies Against SARS-Cov-2*

Treatment of infectious diseases with therapeutic antibodies is an emergent field in clinical research. Although the major objective of this strategy is to block viral replication owing the antiviral effect of antibodies, the wide range of immunological functions of antibodies enlarges their potential benefit. In this regard, it has been widely documented that therapeutic antibodies exert a booster effect on humoral responses (Pelegrin et al., 2015) (**20**). Interestingly, this effect extends beyond the treatment period and may last once the therapeutic antibody has been cleared, suggesting that new specific B cells are being generated during the intervention (Schoofs et al., 2016) (**21**).

Therefore, we hypothesize that individuals treated with hyperimmune plasma might develop a better humoral response against SARS-COV-2 and therefore might show higher titers of neutralization in the long-term. To test this hypothesis, we plan to analyse the neutralizing activity of plasma or serum samples obtained in a subgroup of control and treated participants at three different timepoints: day 0 (before plasma infusion), day 7 (to quantify the impact of infused plasma on neutralizing activity) and day 60 (to quantify the long-term impact of plasma infusion on humoral immune responses). The increase in neutralizing titer between day 0 and day 60 will be compared between control and intervention groups to assess the potential booster effect of treatment. Intergroup comparison of absolute neutralization titers at day 0 and 7 will confirm similarity of pre-treatment and on-treatment titers, respectively, in control and treated participants.

Quantification of neutralizing antibodies against SARS-CoV-2 will be performed in blood samples of a subgroup of individuals. Since no data exist on COVID-19 patients regarding the impact of convalescent plasma on long-term neutralizing activity, the sample size calculation of the subgroup of participants needed will be based on the available data for other infectious diseases. Treatment of HIV infected individuals with a single injection of the broadly neutralizing antibody 3BNC117 induces a significant increase in neutralization titers that is maintained once the therapeutic antibody reaches

undetectable plasma levels (Schoofs et al., 2016) (**21**). Assuming a similar impact on SARS-CoV-2 infected individuals (i.e., similar endpoint differences between groups and similar standard deviation of the measures reported for HIV) a total of 42 (enrolment ratio 1, 21+21) participants would be necessary to reach a statistical power of 80% to observe a difference between the treated and control groups at day 90 (Type I error rate 0.05). Different simulations assuming less favourable scenarios have been also performed. For instance, assuming a reduction of 50% in the difference of the means the sample size would increase to 122 (maintaining the other parameters). Thus 61 and 61 participants from the control and treated groups will be invited to participate in this substudy. This sample size enables to detect differences similar to those reported for HIV infection and is still powered (80%) to identify lower differences (50% reduction in the difference of the means between groups).

#### *2.2.5 Sample Collection Method*

Increase diagnostics are urgently needed to contain the spread of COVID-19. Home self-collecting of swabs may increase testing through improving accessibility outside the healthcare system while minimizing exposure risk and personal protective equipment use. This approach could be safe and scalable in the pandemic setting, allowing for early community detection of COVID-19.

Recent data support the validity of non-nasopharyngeal samples for detection of SARS-CoV-2 (To et al., 2020) (**22**). There is also some data supporting the use of self-collected respiratory swabs (Tu et al., 2020) (**23**), particularly in symptomatic patients with higher viral loads (McCulloch et al., 2020) (**24**). Self-collected mid-turbinate samples had a sensitivity of 96,2% (97,5% CI, 87-100%) when compared with nasopharyngeal samples collected by a health care worker (Tu et al., 2020) (**23**). And self-collected midnasal swabs had a sensitivity of 80% (95% CI, 63%-91%) and a specificity of 97,9% (95% CI, 94%-99,5%) when compared with clinician-collected nasopharyngeal swabs for detection of SARS-CoV-2 infection (McCulloch et al., 2020) (**24**).

In line with available data, nasopharyngeal swabs will be paired with mid-turbinate (MT) swabs at baseline visit to gather further evidence to support clinical performance of self-collected MT swabs.

### 2.3 Hypotheses

This is a prospective, randomized (1:1), double blind study of convalescent anti-SARS-CoV-2 MBT plasma plus standard medical treatment (SMT) versus placebo plus SMT in mild or moderate COVID-19 patients who are non-hospitalised. Convalescent plasma will be provided to patients to assess reduction of viral load and inflammation, clinical efficacy, safety, and tolerability. Patients will be randomly allocated in an experimental or control placebo arm.

The study data and interim results will be monitored by an independent Data Safety and Monitoring Board (DSMB), following the procedures described in **ANNEX 7**. The main task for the DSMB will be to assess whether the randomized comparisons provide evidence on the primary outcomes, sample size re-calculation, safety or futility with evidence strong enough to affect current treatment strategies. In such a circumstance, the DSMB will inform the coordinating investigator and sponsor , who will discuss whether to make the results available to the public. Regardless, follow-up will continue for all randomized individuals.

The hypotheses of the study are the following:

1. Non-hospitalised mild or moderate COVID-19 patients receiving convalescent MBT plasma infusion will have a greater reduction in the severity of disease than those receiving placebo, reflected in a reduction of hospitalization rates.

2. Non-hospitalised mild or moderate COVID-19 patients receiving convalescent MBT plasma infusion will have a lower virologic load on day 7 than those receiving placebo.
3. Non-hospitalised mild or moderate COVID-19 patients receiving convalescent MBT plasma infusion will have a greater reduction in the severity of COVID-19 symptoms than those receiving placebo.
4. Non-hospitalised mild or moderate COVID-19 patients receiving convalescent MBT plasma infusion will have a shorter time to resolution of COVID-19 symptoms than those receiving placebo.
5. Non-hospitalised mild or moderate COVID-19 patients receiving convalescent MBT plasma infusion will have a greater reduction in the severity of disease than those receiving placebo, reflected in a reduction of mortality rates.
6. Non-hospitalised mild or moderate COVID-19 patients receiving convalescent MBT plasma will have a similar number and grade of AEs over a 28-day period, compared to those receiving placebo.
7. Non-hospitalised mild or moderate COVID-19 patients receiving convalescent MBT plasma will have blood lower levels of ferritin, IL-6, D-dimer, CRP, and leucocyte counts, and higher levels of prealbumin and lymphocyte count on day 7, compared to those receiving placebo.
8. Non-hospitalised mild or moderate COVID-19 patients receiving convalescent MBT plasma and placebo will confirm similarity of pre-treatment and on-treatment titers of neutralizing antibodies to SARS-CoV-2 among them on day 7.
9. Non-hospitalised mild or moderate COVID-19 patients receiving convalescent MBT plasma will reach higher titers of neutralizing antibodies to SARS-CoV-2 on day 60 than those receiving placebo, showing a better humoral response.
10. Self-collected MT swab and self-collected saliva will be suitable alternative samples to nasopharyngeal swabs collected by a healthcare worker to evaluate SARS-CoV-2 viral load.

# 3 STUDY OBJECTIVES

## 3.1 Primary Objectives

1. Assess the therapeutic potential of early administration of convalescent MBT plasma in reducing the rate of hospitalization at day 28 in non-hospitalised mild or moderate COVID-19 patients.
2. Assess the therapeutic potential of early administration of convalescent MBT plasma in reducing SARS-CoV-2 viral load at day 7, measured by quantitative RT-PCR (RT-qPCR) in non-hospitalised mild or moderate COVID-19 patients.

## 3.2 Secondary Objectives

1. Assess the therapeutic potential of early administration of convalescent MBT plasma in reducing WHO Clinical progression scale score in non-hospitalised mild or moderate COVID-19 patients.
2. Assess the therapeutic potential of early administration of convalescent MBT plasma in reducing the severity of common COVID-19 symptoms, measured with the FLU-PRO© PLUS scale, in non-hospitalised mild or moderate COVID-19 patients.
3. Assess the therapeutic potential of early administration of convalescent MBT plasma in reducing the duration of symptoms in non-hospitalised mild or moderate COVID-19 patients.
4. Assess the therapeutic potential of early administration of convalescent MBT plasma in reducing the mortality at day 60 in non-hospitalised mild or moderate COVID-19 patients.
5. Evaluate the safety and tolerability of convalescent MBT plasma in non-hospitalised mild or moderate COVID-19 patients.
6. Assess the change from baseline to day 7 of ferritin, prealbumin, interleukin 6 (IL-6), D-dimer, C reactive protein (CRP), and leukocytes and lymphocytes counts in peripheral blood in non-hospitalised mild or moderate COVID-19 patients receiving convalescent MBT plasma.
7. Assess the impact of infused plasma on neutralizing activity by quantifying the change from baseline to day 7 in neutralizing antibodies against SARS-CoV-2 in peripheral blood in non-hospitalised mild or moderate COVID-19 patients receiving convalescent MBT plasma.
8. Assess the long-term impact of plasma infusion on humoral immune responses, by quantifying the change from baseline to day 60 in neutralizing antibodies against SARS-CoV-2 in peripheral blood in non-hospitalised mild or moderate COVID-19 patients receiving convalescent MBT plasma.
9. Compare agreement and SARS-CoV-2 viral load in self-collected middle turbinate (MT) swab and self-collected saliva samples with nasopharyngeal swab samples collected by a healthcare worker.
10. Assess the therapeutic potential of early administration of convalescent MBT plasma in reducing SARS-CoV-2 viral load at day 28, measured by quantitative RT-PCR (RT-qPCR) in non-hospitalised mild or moderate COVID-19 patients.

# 4 TRIAL DESIGN

## 4.1 Type of Trial

This is a multi-site, randomized, controlled with placebo, double blind, parallel study.

## 4.2 Description of the Design

This is a prospective, randomized (1:1), double blind, study of convalescent anti-SARS-CoV-2 MBT plasma plus standard medical treatment (SMT) versus placebo plus SMT in mild or moderate COVID-19 patients who are non-hospitalised. Subjects with a confirmed SARS-CoV-2 infection as determined by positive polymerase chain reaction (PCR) or validated antigen rapid diagnostic test from nasopharyngeal swabs will receive SMT plus one single infusion of 200-250 ml of convalescent plasma that has been pathogen-inactivated using methylene blue treatment (MBT) or 200-250 ml of placebo.

The study will be announced on social media and the press (see ANNEX 6 for dissemination). Primary care centres (CAPs) will inform SARS-CoV-2 positive candidates of the possibility to volunteer in the study. At the CAPs, this information will be given during the medical visit in which patients are informed of their PCR positive result.

Study candidates will voluntarily register at the study website "www.estudicovid19.org/" for SARS-CoV-2-positive individuals to volunteer in COVID-19 clinical trials (see **ANNEX 6** for web content), and will provide an authorization to access their shared medical record.

In order to minimize the transfers of COVID-19 patients, given the risk of contagion that this would entail, eligibility of candidates will be assessed by study physicians in a remote screening visit, based on the revision of regional shared medical records (*Història Clínica Compartida de Catalunya HC3*), during an interview with the candidate. Non-eligible candidates will be informed of the reason for non-eligibility. Eligible candidates will be scheduled an inclusion/baseline visit.

In the inclusion/baseline visit, candidates will be informed in person about the study, formally invited to participate, and the informed consent will be obtained. Once each candidate has signed the informed consent, he/she will undergo the required clinical procedures for final eligibility assessment (i.e., physical examination and a urine pregnancy test). Non-eligible candidates will be informed about the reason for their non-inclusion.

Candidates will also be identified at emergency room (ER) and/or outpatient departments (OPDs) of the participating sites. In this case, the remote screening visit will not be necessary; an inclusion/baseline visit will be the performed, where the candidate will be informed in person about the study, invited to participate and the informed consent will be obtained before the assessment of eligibility.

Eligible candidates will be randomized. To avoid unnecessary tests, ABO compatibility test will be performed only in those that will receive convalescent plasma. The results of the tests will be known only by the unblinded study nurse and the information will be kept blinded for the rest of the study team.

Randomized participants will be administered a single intravenous (IV) infusion at baseline (convalescent plasma or placebo). The administration will be performed by an unblinded study nurse, but both the investigator and the participant will be blinded to the investigational product

Specifically, subjects randomized to combination convalescent anti-SARS-CoV-2 MBT plasma plus SMT will receive one infusion of 200 to 250 ml of ABO-compatible convalescent plasma. Subjects randomized to combination placebo plus SMT will receive one infusion of 200 to 250 ml of sterile saline solution 0.9%. Infusion will be administered at baseline, using standard operating procedures

(SOPs) that will be in place before the study start. Small adults weighing less than 45 kg will receive one infusion of 5 ml of convalescent plasma or placebo per kilogram of body weight (to a maximum of 250ml of convalescent plasma or placebo).

Samples of the infused convalescent anti-SARS-CoV-2 MBT plasma will be obtained before infusion for quantitative measurement of neutralizing antibodies against SARS-CoV-2.

Nasopharyngeal and blood samples will be obtained from all participants at inclusion/baseline visit, on day 7 and on day 28. Nasopharyngeal samples will be used to assess SARS-CoV-2 viral load quantification. Blood samples will be used to assess viral load in nasopharyngeal swabs and inflammatory prognostic markers (ferritin, prealbumin, interleukin 6, D-dimer, C reactive protein leukocyte and lymphocyte counts). Leftovers of plasma will be stored for future investigations.

Participants will be trained on the completion of symptoms and safety diary card.

The symptoms and safety diary card will be filled by the participants daily from baseline up to day 14. Self-reported data will be collected using an electronic case report form (eCRF). Paper forms will be available for those participants who are unable to submit data using eCRF, in which scenario data will be collected at follow-up visits on day 7 and by a study courier on day 15. If the participant reports any new symptom with grade 4 at the FLU-PRO© PLUS questionnaire, he or she will be contacted by telephone by the study staff to evaluate the need of an extra home visit. Participants will be also given a contact number to report AEs, to seek medical advice, and resolve any question related to the study. The investigator team will review the symptoms and safety diary cards on days 3, 7, and 14, to assess compliance with self-reporting data. For participants who do not respond to follow-up surveys, investigators will use emails or telephone calls to ascertain outcomes from them.

On follow-up visits on days 3, 7, 14, and 28, all participants will be assessed for clinical and safety outcomes. These visits will be all by telephone except for the day 7 and the day 28 visits. Day 7 will be a visit at home, where additionally nasopharyngeal swabs and blood samples will be collected. Day 28 will be a hospital visit, where additionally nasopharyngeal swabs sample will be collected

At day 60, all participants will be assessed by telephone for health-status outcome.

If the participant is unreachable at phone follow-up visits, clinical progression, as well as possible AEs will be assessed through medical record (*Història Clínica Compartida, HC3*), in order to assess all the study outcomes.

Results of the sample analyses are not necessary for participants to remain in the study to continue in the study, and participants will only be informed of relevant results for their health.

Participation in the substudy will be offered at the inclusion/baseline visit to a maximum of 135 participants in selected sites and those who accepts will sign a substudy specific informed consent. At baseline, the participants of the substudy will be drawn an extra tube of blood sample and will be trained on self-collection of middle turbinate (MT) swabs and saliva, and self-collected samples will be obtained. On day 7, an extra tube of blood sample will be obtained, and they will be asked to self-collect MT swabs and saliva. And on day 60, an extra tube of blood sample will be obtained during an additional home or hospital visit. The extra tube in both days 7 and 60 will be stored for neutralizing antibodies assessment. MT swabs and saliva samples will be compared with nasopharyngeal swabs for SARS-CoV-2 viral load quantification. Leftovers of plasma will be stored for future investigations.

## 4.3 Study Endpoints

### *4.3.1 Primary endpoint(s)*

- Hospitalization rate (i.e., who reach a score ≥4 in the WHO scale for clinical progression) [Time Frame: Up to 28 days after reception of investigational product]

- Reduction of SARS-CoV-2 viral load in nasopharyngeal swabs at day 7 after start of treatment, as determined by RT-qPCR. [Time Frame: Up to 7 days after reception of investigational product]

#### *4.3.2 Secondary endpoints*

1. Change in COVID-19 WHO Clinical progression scale score [Time Frame: Up to 60 days after reception of investigational product]
2. Change in COVID-19 symptoms severity score, assessed with the COVID-19 daily self-score tool (FLU-PRO© PLUS instrument) [Time Frame: Up to 14 days after reception of investigational product]
3. Time to complete resolution of symptoms [Time Frame: Up to 28 days after reception of investigational product]
4. Death rate [Time Frame: Up to 60 days after reception of investigational product]
5. Proportion of patients with adverse events (AE) and proportion of grade ≥4 AE, based on the FDA Toxicity Grading Scale for Healthy Adult and Adolescent Volunteers scale [Time Frame: Up to 28 days after reception of investigational product]
6. Change in inflammatory prognostic markers (ferritin, prealbumin, interleukin 6 (IL-6), D-dimer, C reactive protein (CRP) and leukocyte and lymphocyte counts) [Time Frame: Baseline and day 7 after reception of investigational product]
7. Intergroup comparison of absolute neutralization titers against SARS-CoV-2 in plasma of a subgroup of participants [Time Frame: Baseline and day 7 after reception of investigational product]
8. Change in titers of neutralizing antibodies against SARS-CoV-2 in plasma of a subgroup of participants [Time Frame: Baseline and day 60 after reception of investigational product]
9. Agreement and SARS-CoV-2 viral loads of self-collected middle turbinate (MT) swab and self-collected saliva compared to nasopharyngeal swabs collected by a healthcare worker [Time Frame: Baseline and day 7 after reception of investigational product].
10. Reduction of SARS-CoV-2 viral load in nasopharyngeal swabs at day 28 after start of treatment, as determined by RT-qPCR. [Time Frame: Up to 28 days after reception of investigational product].

### 4.4 Measures to Avoid Bias

The following measures will be used to avoid bias: Randomization and blinding.

#### *4.4.1 Randomization*

Participants will be randomly allocated using the study eCRF in a ratio of 1:1 between the convalescent plasma arm (experimental) and the placebo arm (control).

Randomization will be centralized at the blood bank during the inclusion/baseline visit, after confirmation of eligibility. ABO compatibility test will be performed for the experimental arm.

#### *4.4.2 Stratification*

There will not be stratification.

#### *4.4.3 Blinding*

This will be a double blind, placebo-controlled study. Masking of investigational products will ensure that both the investigator and the participant are blinded to the product administered.

Opaque bags will be used for masking the investigational products. Convalescent plasma infusion is to be performed using a filter, that opaque infusion system does not include. The investigational product and the system will be covered with opaque bags and opaque tubular bags. Nevertheless, convalescent plasma and placebo have very different appearance and the masking might not be completely efficient for the study nurse manipulating the IV cannula.

To ensure blinding of the participant and the investigators assessing the study outcomes, the following operational measures will be used:

- Randomization and preparation of the investigational product will be performed in the blood bank by unblinded staff , who will cover the investigational product with opaque bags.
- Infusion will be performed by an unblinded study nurse, using an opaque tubular bag to cover the infusion system. Neither the study physician nor the participant will be informed about the investigational product received.
- Study outcomes will be assessed by blinded study physicians. Should the physician assess clinical progression and/or AEs through medical record, specific measures will be used to avoid being informed of the product infused.

#### *4.4.4 Unblinding*

Unblinding of an individual participant will be indicated in the event of a medical emergency where the clinical management of the participant would be altered by knowledge of the investigational product received. In any situation, every attempt will be made to minimize the number of participants unblinded. If the blind is broken for any reason, this will be recorded and the sponsor will be notified immediately. Instructions for unblinding will be provided to the study investigators. Participants will be given a "study participant ID card" including information regarding the study and the contact details of the investigators team (**ANNEX 9**).

Global unblinding for the study team will be performed after the statistical analyses are finalized, and participants will be informed of the treatment received.

#### *4.4.5 Identification of Subjects*

The participant ID will consist of a sequential number that will be assigned to each participant at the inclusion/baseline visit. This number will be used as a unique identifier for each participant throughout the study.

### 4.5 End of Study

The date of the end of the study will be the last visit of the last participant.

The study will be completed when any of these premises are met:

- Inclusion of the number of participants needed for the sample size and end of clinical study visits.
- The DSMB recommends completing the study prematurely for safety reasons.
- Sponsor or principal investigator decision.

If the study must be interrupted prematurely, all non-used materials should be returned to the sponsor. The principal investigator will keep the investigator's site file and a copy of the completed eCRF.

In case there were no participants included in the study, the sponsor will take care of all materials.

### 4.6 Source Data

Source documents will be the participants' electronic health records including laboratory results obtained from blood and swab tests.

The symptom and safety diary card, composed of different adapted questionnaires described in section 7, will be considered source document and will be collected directly on the eCRF (filled by the participant and reviewed by the investigator). Paper questionnaires may also be used by participants who are unable to use the eCRF.

Study data will be collected through an eCRF.

All investigational results obtained from study sample analyses will be recorded in separated electronic databases, which will be merged with the clinical study data for statistical analysis.

# 5 STUDY INVESTIGATIONAL PRODUCTS

## 5.1 Experimental and Control Investigational Products

The experimental investigational product will be ABO-compatible SARS-CoV-2 convalescent anti-SARS-CoV-2 MBT plasma and the control investigational product will be sterile saline solution 0.9%.

Investigational products will be administered in addition to the standard medical treatment (SMT) for COVID-19 disease prescribed by their regular physicians.

## 5.2 Arm Description

After confirmation of eligibility at the inclusion/baseline visit, participants will be randomly allocated to one of the following arms:

| Arm | Investigational Product / Treatment |
|---|---|
| **EXPERIMENTAL ARM** | The participant will receive one infusion of 200 to 250 ml of ABO-compatible convalescent MBT plasma with neutralizing SARS-CoV-2 antibodies plus SMT. |
| **CONTROL ARM** | The participant will receive one infusion of 200 to 250 ml of sterile saline solution 0.9% via IV plus SMT. |

## 5.3 Supply, Packaging, Labelling and Storage

### *5.3.1 Supply*

Convalescent MBT plasma will be supplied by the "Banc de Sang i Teixits, BST".

Commercial saline solution 0.9% will be supplied by the sponsor.

Details regarding donor selection, collection and processing procedures for convalescent MBT plasma preparation from donors recovered from COVID-19 can be found in **ANNEX 1.**

### *5.3.2 Packaging and Labelling*

Packaging and labelling of convalescent plasma and placebo will comply with local regulatory requirements.

### *5.3.3 Storage*

Investigational products will be stored at the blood bank facilities of each study site.

Convalescent anti-SARS-CoV-2 MBT plasma will be stored to the conditions of temperature, humidity and light as defined in the "Banc de Sang i Teixits" standard procedures (**ANNEX 1).**

Commercial saline solution 0.9% will be stored at room temperature.

### *5.3.4 Preparation and Blinding*

Standard operating procedures (SOPs) will be in place for the preparation and blinding of the investigational products.

Both convalescent anti-SARS-CoV-2 MBT plasma and saline solution 0.9% will be prepared for infusion by unblinded personnel. Convalescent anti-SARS-CoV-2 MBT plasma will be thawed before being covered by an opaque bag, while saline solution 0.9% will be directly covered by an opaque bag. To

avoid a blinding breach, the time of preparation and blinding will be approximately the same for both investigational products.

Convalescent anti-SARS-CoV-2 MBT plasma will be thawed following the "Banc de Sang i Teixits" standard procedures.

Opaque bags will be labelled in the same manner, including at least the participant ID and the permitted time frame of infusion. Opaque tubular bags will be used to cover the infusion system. Infusion will be performed by study personnel, adhering to the time frames and storage conditions defined in the "Banc de Sang i Teixits" standard procedures (**ANNEX 1**).

## 5.4 Dose, Interval, Route and Method of Administration

### *5.4.1 Dose, Interval, and Route*

Participants will receive one infusion of 200 to 250 ml ABO-compatible convalescent anti-SARS-CoV2 MBT plasma or saline solution 0.9% at baseline.

Small adults weighing less than 45 kg will receive one infusion of 5 ml of ABO-compatible convalescent plasma or saline solution per kilogram of body weight.

### *5.4.2 Administration and Timing of Investigational Plasma for Each Subject*

Investigational products will be infused using standard procedures for administration of fresh frozen plasma, which will be detailed in the study SOPs.

Start and end of infusion time will be recorded. Vital signs will be measured immediately before the start of infusion, and immediately after the completion of infusion. Participants will remain under observation for 20-30 minutes after the completion of the infusion to monitor AEs.

## 5.5 Modification of the Treatment Regimen

No changes in treatment regimens are expected. If an AE develops during infusion, the infusion will be slowed or stopped as per investigator's decision. Infusion of the investigational product will be halted if any manifestations of anaphylaxis develops, and it will not be restarted.

## 5.6 Prior and Concomitant Treatments

SMT and treatments administered at baseline, and during the study up to day 28 will be considered concomitant treatments, will be recorded in the medical clinical record and in the eCRF, including the trade and/or generic names of the medication, the dose (if known), the route of administration, and the duration of the medication (frequency).

Medicines for transfusion reactions (e.g. paracetamol, dexchlorpheniramine) may be given as a treatment, if necessary.

There are no prohibited medications prior to study participation or during the study.

There are no restricted concomitant medications.

## 5.7 Accountability Procedures for Investigational Product

The investigator, or designee such as the blood bank personnel or study nurse, will keep the investigational products accountability.

Investigational products will be used only for the study in accordance with the directions given in this protocol.

The investigator, or designee such as the blood bank personnel or study nurse, is responsible for handling of the investigational products in accordance with the directions given in the protocol and in the study SOPs. The investigator is responsible for maintaining accurate records of the investigational product for the site. Documentation verifying receipt, dispensing, destruction, or return of the investigational products will be maintained by the investigator or designee and will be made available to the study monitor/relevant authorities for review. Inventory dispensing logs must be verified by the monitor. At the end of the study, a copy of the inventory/dispensing log(s) will be retrieved by the monitor, and the original document will be stored in the trial master file.

## 5.8 Treatment Compliance

A study nurse will perform the investigational product administration, and he/she will register the incidences occurring during the procedure.

# 6 SELECTION AND WITHDRAWAL OF SUBJECTS

## 6.1 Study population

Individuals with confirmed infection by SARS-CoV-2 from *Metropolitana Sud, Metropolitana Nord* and *Barcelona* areas.

## 6.2 Inclusion Criteria

1. Adult male or female individuals of ≥50 years old.
2. In women of childbearing potential[1], negative pregnancy test at inclusion/baseline visit.
3. Has confirmed SARS-CoV-2 infection as determined by PCR or validated antigen rapid diagnostic test[2] from nasopharyngeal swabs ≤5 days prior to inclusion/baseline visit.
4. Symptomatic with mild or moderate COVID-19 with symptoms onset date ≤ 7 days prior to inclusion/baseline visit.
    a. Mild COVID-19: Individuals who have any of the common signs and/or symptoms of COVID-19 (i.e., fever, cough, sore throat, malaise, headache, muscle pain) without shortness of breath, dyspnoea, or abnormal chest imaging.
    b. Moderate COVID-19: Individuals who have evidence of lower respiratory disease by clinical assessment or imaging and a saturation of oxygen ($SpO_2$) ≥94% on room air at sea level.
5. Willing to comply with the requirements of the protocol and available for follow-up for the planned duration of the study.
6. Has understood the information provided and capable of giving informed consent.

*[1]A woman will be considered of childbearing potential if not permanently sterilized nor postmenopausal. Permanent sterilization methods include tubal ligation, hysterectomy and bilateral oophorectomy. Postmenopausal is defined as 12 months with no menses without an alternative medical cause.*

*[2]Panbio™ COVID-19 Ag Rapid Test (Abbott), STANDARD™ Q COVID-19 Ag Test (Roche) or any other CE marketed test for SARS-CoV-2 Ag detection.*

## 6.3 Exclusion Criteria

1. If female, pregnant or breastfeeding, or planning a pregnancy during the study.
2. Severe or critical COVID-19:
    a. Severe COVID-19: respiratory frequency >30 breaths per minute, $SpO_2$ <94% on room air at sea level, ratio of arterial partial pressure of oxygen to fraction of inspired oxygen ($PaO_2/FiO_2$) <300 mmHg, or lung infiltrates >50%.
    b. Critical COVID-19: respiratory failure, septic shock, and/or multiple organ dysfunction.
3. Current hospital admission for any cause.
4. History of previous confirmed SARS-CoV-2 infection.
5. History of significantly abnormal liver function (Child Pugh C).
6. History of chronic kidney disease (CKD) ≥ stage 4, or need of dialysis treatment.
7. Any pre-existing condition that increases risk of thrombosis.
8. History of allergic reactions to blood or plasma products or methylene blue.
9. Known IgA deficiency with anti-IgA antibodies.
10. Medical conditions for which 250 ml of intravenous fluid is considered dangerous (i.e., decompensated heart failure or renal failure with fluid overload).

11. Inability to consent and/or comply with study protocol, in the opinion of the investigator.
12. Currently participating or planning to participate in any interventional study for the treatment of COVID-19 or SARS-CoV-2 infection until day 60.

## 6.4 Subject Withdrawal Criteria

### *6.4.1 Early Subject Withdrawal*

Participants will prematurely end their participation in the study in the following circumstances:

- At their own request.
- If, in the investigator's opinion, continuation in the study would be detrimental to the subject's well-being.
- At the specific request of the sponsor.
- The participant is not able to adhere to the main protocol requirements, in the opinion of the investigator.
- In the exceptional case of non-availability of ABO-compatible convalescent plasma.
- Other (the reason will be specified).

As a general rule, a participant will remain in the study as long as the primary outcome measures are available.

### *6.4.2 Medical Approach to Withdrawal*

Withdrawals will be notified to the sponsor and the date and reason for the withdrawal will be collected. The investigator will provide adequate medical support to the participant if required if required.

### *6.4.3 Follow-up after Early Withdrawal*

Randomized participants who withdraw will be followed up to day 28 for clinical and safety outcomes and up to day 60 for health status outcome, except in case the reason of withdrawing is voluntary.

### *6.4.4 Replacement of Participants*

Replacement of participants will be allowed only in case of early withdrawal happening before the start of the infusion of investigational product.

Participants withdrawn after the start of the infusion will not be replaced.

### *6.4.5 Pre-randomization Loses*

All candidates considered for the study will be registered on the screening list, including the reasons for non-suitability or non-attendance to the inclusion/baseline visit.

Candidates who sign the informed consent will undergo an inclusion/baseline visit; those who are found not eligible will not be randomized, but data from the visit and the reason for non-inclusion will be recorded in the eCRF.

Subject re-screening will not be performed.

# 7 STUDY CONDUCT AND ASSESSMENT OF RESPONSE

## 7.1 Study Visits and Procedures

### *7.1.1 Recruitment procedures*

Candidates will be recruited from:

- Voluntary register through the study website ("www.estudicovid19.org/"), where candidates will be asked essential questions to preliminary assess their suitability (see **ANNEX 6** for web content). The registration on the website will entail an authorization to access the candidate shared medical records to permit a preliminary assessment of eligibility over the phone.
- Invitation through ER or OPD staff of the participating sites, where candidates be invited to participate by study physicians on-site.

### *7.1.2 Screening*

With the aim of minimizing the transfers of COVID-19 patients, given the risk of contagion that this would entail, candidates who voluntarily register on the study website will be assessed for eligibility for the study by study physicians based on the revision of regional shared medical records (*Història Clínica Compartida de Catalunya HC3*) during a phone interview. Non-eligible candidates will be informed of the reason for non-eligibility. Eligible candidates will be scheduled an inclusion/baseline visit.

Candidates identified through ER or OPD of the participating sites will be informed about the study and if interested, will be directed to the inclusion/baseline visit to obtain informed consent and assess eligibility on-site.

A record containing all evaluated candidates and reasons for non-inclusion will be in place for the study (i.e., screening log).

### *7.1.3 Inclusion/baseline*

The inclusion/baseline visit will be a hospital setting visit with a study physician and a study nurse, either at the hospital or, if safe requirements detailed on the study SOPs are met, at home by Hospital at home care unit staff (*Hospitalització domiciliària*).

The procedures will be the following:

1. In-person information of study details
2. Informed consent form signature
3. Urine pregnancy test in females of child-bearing potential
4. Interview of the participant:
    a. Record demographic data.
    b. Record of SARS-CoV-2 PCR or antigen RDT result (including date of analysis).
    c. Identification baseline medical conditions and concomitant treatment.
    d. Identification of date of onset of COVID-19 symptoms.
5. Physical examination (including measuring weight and height) and vital signs (Body temperature, Oxygen saturation, Respiratory rate, Heart rate, Blood pressure).
6. Verification of inclusion and exclusion criteria. In case of non-inclusion, the participant will be informed of the reason and the study staff will provide the necessary medical attention.

If the participant is found eligible, the study staff will perform the following procedures:

1. In the selected sites, offer participation in the substudy and sign the specific informed consent.

2. Classification of baseline severity of COVID-19 using WHO scale.
3. Blood draw:
    a. Blood sample collection for an ABO-compatibility test (which will be performed in case the participant is assigned to convalescent plasma group).
    b. Blood sample collection for inflammatory prognostic markers (ferritin, prealbumin, IL-6, D-dimer, CRP, and leukocyte and lymphocyte counts).
    c. Blood sample collection for storage at IrsiCaixa.
    d. In substudy participants, blood sample collection for quantification of neutralizing antibodies against SARS-CoV-2.
4. Nasopharyngeal swabs collection by study nurse.
5. In the substudy participants, training of the participant on the process of self-collection of MT swabs and saliva samples, and self-collection of MT swabs and saliva samples by the participant.
6. Training in the completion of the symptoms and safety diary card, which will be filled by the participant at baseline and daily until day 14, using the study an eCRF. Paper forms will be available for those participants who are unable to submit electronic data using eCRF, and in this scenario data will be collected in the eCRF at follow-up visits on day 7 and by a study courier on day 15.
    a. If the participant reports any new symptom with grade 4 at the FLU-PRO© PLUS questionnaire, he or she will be contacted by telephone by the study staff to evaluate the need of an extra home visit
    b. Participants will be also given a contact number to report AEs, to seek medical advice, and resolve any question related to the study.
7. Randomization to assign the participant to a study arm, centralized at the Blood Bank facilities by study staff.
8. In case of convalescent plasma is assigned, the blood bank staff will perform the ABO compatibility test using standard guidelines for blood transfusion laboratories.
9. Preparation and blinding of ABO-compatible convalescent plasma or placebo to be administered.
10. Transport of the investigational product from the Blood Bank to the place of infusion.
11. Administration of a single infusion of 200 to 250 ml, of investigational product (either convalescent plasma or placebo).
    a. Time at start and end of infusion will be recorded.
    b. Infusion will be done in accordance with the standard policies for administration of blood and routine administration of fresh frozen plasma, and study specific SOPs will be in place.
    c. If an AE develops during infusion, the infusion will be slowed or stopped as per investigator's decision. Infusion will be halted if any manifestation of anaphylaxis develops, and will not be restarted.
    d. Vital signs (Temperature, Oxygen saturation, Respiratory rate, Heart rate, Blood pressure) will be measured at immediately after the completion of infusion.
    e. Medicines for infusion reactions (e.g. paracetamol, dexchlorpheniramine) may be given as a treatment, if necessary.
12. Monitoring of AEs for a period of 20-30 minutes after the completion of the infusion.
13. Provide the participant with the "study participant ID card" (**ANNEX 9**), and instruct the participant to always carry the card with him/her.

#### *7.1.4 Day 3*

The participant will be contacted by telephone by study staff for safety and clinical follow-up. Specifically,

1. Revision of symptom and safety daily data, and discussion of inconsistencies with the participant.
2. Classification of severity of COVID-19 using WHO scale.
3. AEs will be assessed by the study staff.
4. If deemed necessary, AEs will be informed to and reviewed by the study physician, and medical attention will be given when necessary.

#### *7.1.5 Day 7*

The participant will be visited at home by study staff, and the following procedures will be performed:

1. Vital signs (Temperature, Oxygen saturation, Respiratory rate, Heart rate, Blood pressure).
2. Blood draw:
    a. Blood sample collection for inflammatory prognostic markers (ferritin, prealbumin, IL-6, D-dimer, CRP, and leukocyte and lymphocyte count). This sample will be sent to the laboratory for a routine processing.
    b. In the substudy participants, blood sample collection for quantification of neutralizing antibodies against SARS-CoV-2.
3. Nasopharyngeal swab sample collection.
4. In the substudy participants, self-collection of MT swabs and saliva samples by the participant.
5. Revision of symptom and safety daily data, discussion of inconsistencies with the participant.
6. Classification of severity of COVID-19 using WHO scale.
7. AEs will be assessed by the study staff.
8. If deemed necessary, AEs will be informed to and reviewed by the study physician, and medical attention will be given when necessary.

#### *7.1.6 Day 14*

The participant will be contacted by telephone by study staff for safety and clinical follow-up. Specifically,

1. Revision of symptom and safety daily data, and discussion of inconsistencies with the participant
2. Classification of severity of COVID-19 using WHO scale.
3. AEs will be assessed by the study staff.
4. If deemed necessary, AEs will be informed to and reviewed by the study physician, and medical attention will be given when necessary.

#### *7.1.7 Day 28*

The participant will be scheduled a visit with a study nurse at the hospital, where the following procedures will be performed:

1. Vital signs (Temperature, Oxygen saturation, Respiratory rate, Heart rate, Blood pressure).
2. Nasopharyngeal swab sample collection.
3. Classification of severity of COVID-19 using WHO scale.
4. Assessment of COVID-19 symptoms that were still persisting at day 14 (as collected in the symptom diary) will be assessed as “persisting” or “resolved”.
5. AEs will be assessed by the study staff.

6. AEs will be informed to and reviewed by the study physician, and medical attention will be given when necessary.

#### *7.1.8 Day 60 (End-of-Study)*

The participant will be contacted by telephone by study staff for safety and clinical follow-up. Specifically,

1. Classification of severity of COVID-19 using WHO scale.
2. In the substudy participants, a visit will be scheduled within 5 days to obtain a blood sample.

#### *7.1.9 Extra home visit (unscheduled visit)*

The need of an extra home visit will be assessed by phone in case of:

- Report of any new symptom with grade 4 at the FLU-PRO© PLUS questionnaire
- Report of any AE of grade 3 or more

If deemed necessary by the study staff, at any time during the study period, the participant will be visited at home to perform the necessary procedures to assess an AE or provide medical attention.

#### *7.1.10 Participant's Diary*

Additionally to the scheduled study visits, the participant will perform the following procedures daily from day 1 to day 14:

1. Measurement of axillary body temperature
2. Fill the symptom and safety diary card, which will include the FLU-PRO© PLUS Questionnaire and additional questions regarding safety, and medications taken by the participant.

Data will be collected using an electronic case report form (eCRF). Paper forms will be available for those participants who are unable to submit data using eCRF, and in this scenario data will be collected at follow-up visit on day 7 and by a study courier on day 15.

### 7.2 Clinical Progression Scales

The following scales will be used to assess clinical progression of the participants:

#### *7.2.1 WHO Clinical Progression Scale*

This scale will be assessed by the study staff at baseline, and days 3, 7, 14, 28, and 60.

The WHO Clinical Progression Scale is recommended for non-mortal clinical outcomes (WHO Working Group on the Clinical Characterisation and Management of COVID-19 infection, 2020) (**15**), and will assess hospitalization rate (i.e., who reach a score ≥4).

This is an ordinal scale to measure clinical progression and recovery based on location and supportive measures used within the health-care system. It provides a measure of illness severity across a range from 0 (not infected) to 10 (death) with data elements that are rapidly obtainable from clinical records.

The WHO Clinical Progression Scale is located in **ANNEX 2**.

#### *7.2.2 FLU-PRO® PLUS Questionnaire*

Participants will fill daily questionnaires from baseline to day 14. Data will be self-reported, collected using the study eCRF and reviewed by the investigator at baseline, and days 3, 7, and 14.

Symptom severity will be also assessed with the certified-Spanish translation of the FLU-PRO© PLUS Questionnaire. It is a COVID-19 daily self-score tool, to assess severity of symptoms across six body systems: nose, throat, eyes, chest/respiratory, gastrointestinal, and body/systemic.

The instrument also provides data on the presence/absence of symptoms, symptom profiles, and change over time.

Adapted from the FLU-PRO® instrument, the COVID version consists of 34 items that are answered daily. Items 1-27 are Likert scale questions (rated 0-4) where 0 = not at all and 4 = very much. These items are summed to score the severity of symptoms- where a total score of 112 would indicate the greatest severity of symptoms and a score of 0 would indicate no severity of symptoms. Items 28-32 are also Likert scale questions (rated 0-4) that measure the frequency of specific daily symptoms where 0 = never or 0 times and 4 = always or 4 times. These items are summed to score the frequency of symptoms, where the highest score for the frequency of symptoms (20) indicates the greatest burden of symptom frequency. Items 33 and 34 are yes/no questions that measure the presence/absence of COVID-19 specific symptoms, where 0 = no and 1 = yes. These items are summed, where the highest score of 2 indicates presence of both symptoms. The total maximum score of the FLU-PRO© PLUS questionnaire is 134.

The FLU-PRO®PLUS Questionnaire is located in **ANNEX 3.** For the present study.

#### *7.2.3 Assessment of Safety*

Safety will be assessed using the safety diary card filled daily by participants and through telephone contact by study staff on day 3, 7 and 14. Safety diary card and phone assessments are detailed in **ANNEX 4**.

Participants will fill daily from baseline to day 14 self-reported data related to symptoms and safety. These data will include medication-related effects with directed questioning on the most common adverse effects and an open-ended free-text field.

The investigator team will review surveys on days 3, 7, and 14, to assess compliance in self-reporting data and safety data will be also collected, with direct questioning on common AEs and indirect questioning on other possible AEs.

If the participant reports any new symptom with grade 4 at the FLU-PRO© PLUS questionnaire or any AE of grade 3 or more, he or she will be contacted by telephone by the study staff to evaluate the need of an extra home visit. The need of an extra home visit will be evaluated if an important AE is detected on the phone visits.

Participants will be also given a contact number to report AEs, to seek medical advice, and resolve any question related to the study.

For participants who would not respond to follow-up surveys, investigators will use e-mails or telephone calls to ascertain outcomes from them.

### 7.3 Description of Laboratory Tests and Procedures

| Test | Type of sample | Description | Location | Visit |
|---|---|---|---|---|
| Inflammatory prognostic markers | Blood sample | White cell blood count , ferritin, prealbumin, interleukin 6 (IL-6), D-dimer, C reactive protein (CRP) | HUGTiP Central Laboratory | Baseline* and day 7 |
| ABO compatibility test | Blood sample | Type of anti-A and anti-B in red cells and in serum only in participants assigned to convalescent plasma group | BST | Baseline* |
| Quantitative measurement of SARS-CoV-2 viral load | Nasopharyngeal swab | Quantitative RT-PCR of SARS-CoV-2 | IrsiCaixa | Baseline*, days 7 and 28 |
| Quantitative measurement of SARS-CoV-2 viral load | Self-collected MT swab and self-collected saliva | Quantitative RT-PCR of SARS-CoV-2 (substudy) | IrsiCaixa | Baseline* and day 7 |
| Quantitative measurement of neutralizing antibodies against SARS-CoV-2 | Convalescent anti-SARS-CoV-2 MBT plasma infused | Quantitative measurement of neutralizing antibodies against SARS-CoV-2 | IrsiCaixa | Baseline |
| Quantitative measurement of neutralizing antibodies against SARS-CoV-2 | Blood sample | Quantitative measurement of neutralizing antibodies against SARS-CoV-2 (substudy) | IrsiCaixa | Baseline*, day 7 and 60 |
| Storage | Plasma | Future investigations. | IrsiCaixa and ISGlobal | Baseline* |
| | Leftovers of SARS-CoV-2 positive nasopharyngeal swab | Future investigations. | HUGTiP | Baseline* and days 7, and 28 |
| | Leftovers of SARS-CoV-2 positive self-collected MT swab and self-collected saliva | Future investigations. | HUGTiP | Baseline* and day 7. |

**All baseline samples will be obtained before start of infusion of investigational product.*

### 7.4 Study Variables

Among others, the following study variables will be collected:

Demographic data:

1. Age
2. Sex (Male/Female)

Clinical data:

1. Comorbidities
    a. Chronic obstructive pulmonary disease
    b. Asthma
    c. Chronic hypertension
    d. Obesity
    e. Cardiovascular disease (Chronic congestive cardiac failure, ischemic heart disease)
    f. Chronic kidney disease
    g. Diabetes
    h. Cerebrovascular disease
    i. Cancer
    j. Immunosuppression
2. Concomitant treatments

COVID-19 clinical data:

1. Date of positive SARS-CoV-2 result
2. Type of test:
    a. RT-PCR
    b. Validated antigen rapid diagnostic test
3. Date of symptom onset
4. COVID-19 severity (mild, moderate, severe or critical)

Participant diary card, list of symptoms (extracted from the FLU-PRO© PLUS Questionnaire, **ANNEX 3**):

1. Fever
    a. Felt cold
    b. Felt hot
    c. Sweating
2. Cough
    a. Dry or hacking cough
    b. Wet or loose cough
    c. Frequency of coughing
    d. Frequency of coughing up mucus or phlegm
3. Sore throat
    a. Scratchy or itchy throat
    b. Sore or painful throat
    c. Difficulty swallowing
4. Malaise
    a. Lack of appetite
    b. Sleeping more than usual

    c. Weak or tired
    d. Chills or shivering
5. Headache
    a. Felt dizzy
    b. Head congestion
    c. Headache
6. Muscle pain
    a. Body aches or pains
7. Shortness of breath, dyspnoea
    a. Trouble breathing
    b. Chest congestion
    c. Chest tightness
8. Gastrointestinal symptoms
    a. Felt nauseous
    b. Stomach ache
    c. Frequency of vomiting
    d. Frequency of diarrhoea
9. Anosmia
10. Ageusia
11. Rhinitis
    a. Runny or dripping nose
    b. Congested or stuffy nose
    c. Sinus pressure
    d. Frequency of sneezing
12. Conjunctivitis
    a. Teary or watery eyes
    b. Sore or painful eyes
    c. Eyes sensitive to light

Participant diary card, safety:

1. Injection site pain
2. Injection site swelling
3. Rash
4. Injection site pruritus

Physical examination and vital signs:

1. Saturation of oxygen on room air at sea level
2. Blood pressure
3. Temperature
4. Respiratory rate
5. Heart rate
6. Weight measurement
7. Height measurement

Convalescent anti-SARS-CoV-2 MBT plasma analysis:

1. Titers of anti-SARS-CoV-2 antibodies in donor's MBT plasma

Blood analysis:

1. Complete Blood Count (lymphocytes, leucocyte count)
2. Ferritin
3. Prealbumin
4. Interleukin 6 (IL-6)
5. D-dimer
6. C-reactive Protein (CRP)
7. Titers of anti-SARS-CoV-2 antibodies

Respiratory swabs (nasopharyngeal and MT) and saliva analysis:

1. SARS-CoV-2 RT-qPCR

Hospitalization

1. Date of hospitalization
2. Cause
3. Notification and collection of data as defined in section 8

Death

1. Date
2. Cause

Radiological findings (if available)

1. Chest CT scan
2. Chest x-ray

Adverse events

1. As defined in section 8

Concomitant treatments

1. As defined in section 5.6

## 7.5 Schedule of Procedures

| | Screening (phone)[6] | Inclusion/Baseline (hospital setting visit)[7] | Day 3 (phone) | Day 7 (home) | Day 14 (phone) | Day 28 (hospital) | Day 60 (phone) |
|---|---|---|---|---|---|---|---|
| Allowable window (days) | N/A | 0 | + 2 | +/-2 | +/-2 | +/- 2 | +/- 2 |
| Authorization to access medical records[1] | X | | | | | | |
| Informed consent signature | | X | | | | | |
| Pregnancy test | | X | | | | | |
| Clinical examination | | X | | | | | |
| Vital signs | | X | | X | | X | |
| Demographic, clinical data, and PCR record | | X | | | | | |
| Inclusion / exclusion criteria | X | X | | | | | |
| Nasopharyngeal swab | | X[2] | | X | | X | |
| MT swabs (self-collection) | | X[2,3] | | X[3] | | | |
| Saliva sample (self-collection) | | X[2,3] | | X[3] | | | |
| Blood draw | | X[2] | | X | | | X[3] |
| *ABO compatibility test* | | X[2,4] | | | | | |
| *Inflammatory prognostic markers* | | X[2] | | X | | | |
| *Neutralizing antibodies* | | X[2,3] | | X[3] | | | X[3] |
| *Sample storage* | | X[2] | | X | | | X[3] |
| Investigational product infusion | | X | | | | | |
| Symptom and safety diary card training | | X | | | | | |
| Adverse events | | X ---------------------------------------------------- X | | | | | X[5] |
| Symptom and safety diary card | | X ------------------------------------------ X | | | | | |
| Symptom persistency or resolution | | | | | | X | |
| Follow up assessment | | X ---------------------------------------------------------- X | | | | | |
| Concomitant Treatment | | X ---------------------------------------------------------- X | | | | | |

*MT: mid-turbinate; N/A: Not applicable*

[1] *In order to minimize the transfers of COVID-19 patients, given the risk of contagion that this would entail, eligibility will be assessed based on the revision of regional shared medical records (*Història Clínica Compartida de Catalunya HC3*), and clinical history taken by phone.*

[2] *Samples at baseline will be obtained prior to the infusion of the investigational product.* [3] *Substudy in a subgroup of 135 participants. Day 60 blood draw allowable window will be +5 days from the actual day of the day 60 visit.*

[4] *Blood for the ABO compatibility test will be collected from all study participants, but the test will only be performed in experimental (convalescent plasma) group.*

[5] *The time frame for new AE collection will end at day 28 visit. At day 60 visit, the investigator must collect follow-up data of AEs that were not resolved on day 28, and SAEs due to hospitalization or death.*

[6] *The screening remote visit will only be performed in participants registered on the study website.*

[7] *A visit at home by the Hospital at home care unit staff will be allowed if safe requirements detailed on the study SOPs are met.*

# 8 ADVERSE EVENTS AND INCIDENTS

## 8.1 Safety parameters

The safety endpoints will include:

1. Cumulative incidence of adverse events (AEs) through day 28
2. Cumulative incidence of serious adverse events (SAEs) through day 28
3. Cumulative incidence of severe AEs through day 28

In this study, disease progression is defined as the worsening of a subject's condition attributable to the disease for which the investigational product is being studied. It may be an increase in the severity of the targeted disease and/or increases in the symptoms of the targeted disease. Anticipated symptoms of COVID-19 include fever, cough, hypoxia, dyspnoea, haemoptysis, myalgia, fatigue, pharyngitis, diarrhoea which may develop at any time during the course of the disease.

## 8.2 Definitions

**Adverse event (AE):** Medical event presented by a participant administered an investigational product, and which does not necessarily have a causal relation to the treatment.

**Serious adverse event (SAE):** Medical event classified as such and which, regardless of the dose involved:

1. Causes participant death.
2. Produces a life-threatening situation for the participant.
3. Requires or prolongs in hospital admission.
4. Produces important or persistent incapacitation/handicap or constitutes a congenital defect or anomaly.
5. Needs action to prevent any of above situations.
6. Is considered medically significant (examples of such events are intensive care in an Emergency Service or at home in a patient with allergic bronchospasm; blood dyscrasias or seizures not giving rise to hospital admission, or the development of drug dependency or abuse).

**Unexpected adverse event (UAE):** AE related to the investigational product the nature or intensity of which does not coincide with the information available on the product.

**Suspected Unexpected Serious Adverse Reaction (SUSAR)**: SAE related to the investigational product the nature or intensity of which does not coincide with the information available on the product.

**Incident related to the infusion:**

- **Mistake in component infusion:** event in which a participant is administered an investigational product that is non-compliant or was intended for another participant.
- **Incident without effect or almost-incident:** event in which a mistake in component infusion is detected before happening.

## 8.3 Adverse Events Assessment

### *8.3.1 Seriousness*

A serious AE is any medical event that meets the criteria of SAE.

Events not considered to be SAEs are hospitalizations for:

1. A standard procedure for protocol therapy administration. However, hospitalization or prolonged hospitalization for a complication of therapy administration will be reported as an SAE.
2. Routine treatment or monitoring of the studied indication not associated with any deterioration in condition.
3. A procedure for protocol/disease-related investigations (e.g., surgery, scans, endoscopy, sampling for laboratory tests, bone marrow sampling). However, hospitalization or prolonged hospitalization for a complication of such procedures remains a reportable SAE.
4. Hospitalization or prolongation of hospitalization for technical, practical, or social reasons, in absence of an AE.
5. A procedure that is planned (i.e., planned prior to starting of treatment on study); must be documented in the source document and the CRF. Hospitalization or prolonged hospitalization for a complication remains a reportable SAE.
6. An elective treatment of a pre-existing condition unrelated to the studied indication.
7. Emergency out participant treatment or observation that does not result in admission, unless fulfilling other seriousness criteria above.

### *8.3.2 Intensity*

The FDA *Toxicity Grading Scale for Healthy Adult and Adolescent Volunteers scale* will be used to grade the intensity of the AE. Events not listed in the grading scale will be graded as follows:

| | |
|---|---|
| Grade 1 | Mild. No interference with activity. |
| Grade 2 | Moderate. Some interference with activity not requiring medical intervention. |
| Grade 3 | Severe. Prevents daily activity and requires medical intervention. |
| Grade 4 | Potentially Life Threatening. Emergency Room visit or hospitalization. |
| Grade 5 | Death. |

### *8.3.3 Causality*

All AEs must have their relationship to the investigational product assessed by the physician who examines/contacts and evaluates the participant based on temporal relationship and his/her clinical judgment. Causal relationship to the investigational product will be established according to medical judgment on whether there is a reasonable possibility of a causal relationship between the AE and the investigational product administration.

The investigator must determine and classify the AE causality according to the following categories:

- Unrelated/Not related: There is not a reasonable possibility that the administration of the investigational product caused the AE, there is no temporal relationship between the investigational product administration and AE onset, or an alternate aetiology has been established.

- Related: The AE is known to occur with the investigational product, there is a reasonable possibility that the investigational product caused the AE, or there is a temporal relationship between the investigational product administration and the AE. Reasonable possibility means that there is evidence to suggest a causal relationship between the investigational product administration and the AE.

- Possibly related: There is evidence to suggest a causal relationship between the investigational product and the AE.
- Definitely related: There is a reason to conclude that the investigational product caused the AE.

Criteria to assess the causal relationship should take in account of the following conditions.

1. A plausible temporal sequence from the investigational product administration to the AE onset.
2. Whether the AE follows a known response pattern to the suspected treatment.
3. Whether the AE could be reasonable explained by the participant's clinical state, comorbidities, or concomitant medications.
4. The occurrence of improvement on stopping/reducing the treatment (positive dechallenge) and/or reappearance of the AE on repeated exposure (positive rechallenge).

#### *8.3.4 Expectedness*

An AE related to the investigational product will be considered "unexpected" if the nature, seriousness, severity, or outcome of the reaction(s) is not consistent with the reference information or expected effects of fresh frozen plasma infusion.

The assessment of the expectedness between an AE and the administration of treatment is a decision to be made by the principal investigator or co-investigator, who are qualified physicians.

A suspected unexpected serious adverse reaction (SUSAR) is a serious adverse event related to the investigational product whose nature, severity or outcome is not consistent with the reference information or expected effects of the fresh frozen plasma infusion.

All unexpected serious ARs will be notified to the study sponsor.

#### *8.3.5 Duration*

For both AEs and SAEs, the investigator will provide a record of start and stop dates of the event (expressed in the shortest time unit possible). Changes in the severity of an AE or SAE will be documented in the clinical record.

For AEs that occur during the infusion, the infusion rate in effect at the time of onset of the AE, the time of onset of the AE and the time of AE change materially in intensity and/or resolve will be captured.

#### *8.3.6 Action Taken*

The investigator will report the action taken with study intervention as a result of an AE or SAE, as applicable (i.e., discontinuation or reduction of volume of infusion, as appropriate) and report whether concomitant and/or additional treatments were given for the event.

#### *8.3.7 Outcome*

Any AE or SAE will be followed preferably until:

- Resolution of the event.
- Stabilization of the event
- Resetting the baseline situation of the event, in case baseline situation is available.

Otherwise, they will continue until:

- The event can be attributed to products other than the investigational product or factors unrelated to the study; or

- It is unlikely to obtain further information.

If the subject dies from a SAE, the rest of AE or SAE that are active will be recorded as “not recovered”.

## 8.4 Time Frame for Recording Adverse Events and Incidents

The investigator must collect all the AE, SAE and incidences that occur from the moment the subject signs the informed consent until day 28. On day 60, the investigator must collect follow-up data of AEs that were not resolved on day 28, and SAEs due to hospitalization or death.

## 8.5 Documentation Related to Adverse Events and Incidents

Each AE, SAE or incident to take place during the study will be documented in the medical records of the participant in accordance with standard clinical practice of the investigator. For each SAE, an independent set of SAE form will be used. Only if there are multiple SAE at the time of the initial report and these are temporary and / or clinically interrelated can be registered on the same set of SAE form.

The investigator should try to make a diagnosis of the event based on the signs, symptoms and / or other clinical information. An AE diagnosis must be recorded per line, or a sign/symptom if the diagnosis is not available. If a diagnosis subsequently becomes available, this then should be entered, and the sign/symptom crossed out.

SAE pages found in the investigator’s file shall be completed as precisely as possible and shall be signed by the investigator before being sent to sponsor. In the initial page of the SAE form, the investigator must provide his/her opinion regarding the relationship of the event to the study intervention.

## 8.6 Pregnancy

Cases of pregnancy will be recorded as AE and should only be considered as SAE only if they meet any seriousness criteria. Pregnancy will also be a protocol deviation. The investigator will provide the necessary medical advice to the pregnant participant.

No special measures are required in relation to the pregnancy of a partner of a male participant.

## 8.7 Procedure for Adverse Event Reporting

### *8.7.1 Investigator*

The recording of AEs, SAEs and incidences is the responsibility of the study investigator team, which should indicate the time of appearance of the event (expressed in the shortest time unit possible), its serious / not serious status, and in case it is considered related to investigational products, whether it was expected or unexpected. The intensity of the event (grade 1 to 5) is to be specified, along with the measures adopted (none, treatment, temporal or permanent discontinuation of investigational product), course (complete remission, partial remission, persistence) and causality based on the criteria indicated in section 8.3.3.

All AEs, SAEs and incidences will be recorded, regardless of the causality or outcome, in the corresponding form of the eCRF of each participant. Depending on the nature of the event, each AE will be classified as:

- Serious / non-serious
- Related expected / related unexpected

The investigator will immediately notify the study sponsor of any SAE, any related AE, or any incidence. The notification will be performed within 24 hours of first knowledge by the investigator.

**Contact Details for Sponsor:**

safety@fls-rs.com

#### *8.7.2 Sponsor*

The sponsor will inform the relevant Ethics Committee about any important information of security of the investigational product.

The sponsor will inform the relevant Ethics Committee of any SUSAR which may be related to the investigational product.

The deadlines to notify a SUSAR is, from the first knowledge by the sponsor:

1. 15 days
2. 7 days if the SUSAR has resolved in death or has been life-threatening. Relevant follow-up information for these cases will be subsequently submitted within an additional 8 days.

The sponsor will inform the hemovigilance service of any AE, or any incidence related to the infusion.

The sponsor will keep a detailed register of all the AEs and SAEs notified by the investigators and all AEs, SAEs and incidents will be listed in table form in the final report of the study.

### 8.8 Data and Safety Monitoring Board

An independent Data and Safety Monitoring Board (DSMB) has been convened to assess the progress of this clinical study, the safety data, and critical efficacy endpoints and provide recommendations to the study PI. The members of the DSMB serve in an individual capacity and provide their expertise and recommendations. The DSMB will review cumulative study data to evaluate safety, efficacy, study conduct, and scientific validity and data integrity of the study.

The composition, responsibilities and independence of the DSMB, as well as the planned meetings and communication flow, will be detailed in **ANNEX 7**.

# 9 STATISTICAL ANALYSIS

## 9.1 Methods

### *9.1.1 Descriptive Analysis*

The characteristics of the study population will be described using frequencies for categorical variables and using mean and standard deviation for quantitative variables.

### *9.1.2 Bivariate Analysis*

We will use the chi-square test for categorical variables and the t-test and analysis of variance for continuous variables.

### *9.1.3 Multivariate Analysis*

Event rate comparison between groups

Hospitalization and/or death rate between groups will be analysed using the odds-ratio (OR) obtained by fitting a logistic regression model.

Longitudinal VL decay

Efficacy will be determined by comparing the mean reduction of the viral load from baseline to days 7 and 28, with the use of a linear/non-linear mixed effect model taking into account the randomization group and repeated measures within each individual. The viral load will be provided in logarithmic scale; if less than 15% of specimens presents undetectable viral load at a given follow-up assessment a value of 3 $log_{10}$ copies per mL (i.e., lower limit of detection) will be assigned for the purpose of statistical analysis. Otherwise, we will consider undetectable viral loads as left-censored values.

Mean change differences between groups

The secondary clinical outcome regarding between-group differences in symptoms severity score will be assessed by means of linear regression.

Time to event analysis

The time to complete resolution of symptoms will be analysed using Kaplan-Meier survival functions and hazard ratios (HRs), calculated using a Cox proportional hazards regression model based on the assumptions of proportional risks. Kaplan-Meier estimates will be compared using the log-rank test.

Variable selection

All models will be adjusted with basal covariates chosen according to their clinical relevance or from the observed correlation with the study outcome, and variable selection procedure will be done using the AIC-based stepwise algorithm.

Missing data and outliers

No missing data imputation method will be used in this study and we will not analyse the presence of potential outliers.

All analyses will be done with the R statistical package, version 6.3 or higher under a significance level of 0.05.

## 9.2 Analysis Population

The primary efficacy analysis will be performed on the intention-to-treat (ITT) population, which will include all randomized participants. If deemed necessary, sensitivity analyses will be performed with

the per-protocol (PP) population. Safety will be assessed in the safety population, which will include all participants who received investigational product (convalescent plasma or placebo).

### 9.3 Sample Size

A sample size of 237 cases per arm would provide the trial with 80% power to detect 50% reduction in hospitalization rate at day 28 after starting the treatment, assuming an expected rate of hospitalization of 15%, allowing a 5% of loss to follow-up.

Approximately 157 cases per arm are required to have 80% power to detect a minimal expected difference of 5 points in a scale of 156 points with 39 items, to assess symptoms' severity, at a significance level of $\alpha = 0.05$, assuming a standard deviation of 15.

Approximately 150 cases per arm are required to have 80% power to detect a difference of 0.5 log10 in the mean reduction of SARS-CoV-2 viral load at a two-sided significance level of $\alpha = 0.05$, assuming an expected overall standard deviation of 1.5. A 0.5 $\log_{10}$ copies/mL difference in reduction was chosen to represent the minimal threshold for a biologically relevant change for our analyses.

For the substudy for quantification of neutralizing antibodies against SARS-CoV-2 a total sample size of 135 was estimated considering parameters detailed in the rationale.

### 9.4 Interim Analyses

An interim analysis of efficacy and safety variables will be performed after the first 60 participants achieve the primary endpoint (i.e., day 28) for the purpose of sample size recalculation. The interim analyses will be performed using blinded data, unless otherwise indicated by the DSMB.

### 9.5 Deviation of Statistical Plan

Any deviation from that presented statistical plan will be described and justified in the final study report.

## 10 DIRECT ACCESS TO SOURCE DATA/DOCUMENTS

Investigators and institutions will allow the monitoring, and audits by the sponsor giving direct access to data and original source documents.

Access to personal participant information will be restricted to the study investigator and staff. To allow monitoring, audits and inspections, access to data to the Ethics Committee and personnel authorized by the sponsor, is guaranteed while maintaining the confidentiality thereof according to current legislation, in accordance with the Data Protection Law (LOPD, the organic Law 3/2018 of 5 December on the Protection of Personal Data and the Guarantee of Digital Rights complementary to the Regulation (EU) 2016/679 of the European Parliament and of the Council of 27 April 2016, on the protection of natural persons with regard to the processing of personal data and on the free movement of such data).

The principal investigator will have overall control of, and will act as the custodian for all data for the full duration of the study.

# 11 QUALITY CONTROL AND QUALITY ASSURANCE

## 11.1 Study Monitoring

In accordance with applicable regulations and Good Clinical Practice (GCP), monitoring visits will be performed during the study.

Risk-adjusted monitoring will be carried out since the study is performed in a clinical care practice setting, with follow-up of the subjects treated in the community or primary care setting.

The goal of the monitoring activity is to verify that:

- the rights and welfare of the participants are respected;
- data collected are accurate, complete and verifiable with the help of original documents;
- the study is performed according to the protocol and any amendment adopted, GCPs and regulations.

The investigator must agree to:

- grant to monitor direct access to all relevant documentation;
- devote part of his/her time and staff time to the monitor in order to discuss the results of the monitoring, as well as any other possible aspect.

Data monitoring tasks will be set up in the Monitoring Plan, in general:

1. Verification of the study master file (approvals, protocol, investigational product information and other essential documents, pursuant to section 8 of ICH Guide E6).
2. Verification of signature of informed consent.
3. Check of dates of visit and source data verification.
4. Detection of inconsistencies in the eCRF by review of data in the medical records.

## 11.2 Protocol Deviations

Major or critical deviations (i.e., that may have an impact on participant safety and data integrity) will be notified immediately to the sponsor. The sponsor will review all deviations from the protocol and assess whether any represents a serious breach of GCP and requires that the Ethics Committee be informed about it. The sponsor should report, when necessary, to the Ethics Committee any serious breach of current legislation or of the version of the protocol approved at the time of the breach that has occurred in Spain without delay and not later than 7 calendar days from becoming aware of the breach.

## 11.3 Audits and Inspections

The sponsor can carry out an audit of quality control at its sole discretion. In this case, the investigator should agree to grant the auditor direct access to all relevant documentation and devote part of his/her time and staff time to the auditor in order to discuss the results of the monitoring, as well as any other possible aspect.

Regulatory authorities may also inspect the study. In this case, the investigator should agree to give the inspector direct access to all relevant documentation and devote part of his/her time and staff time to the inspector in order to discuss the results of the supervision, as well as any other possible aspect.

### 11.4 Data Collection (electronic Case Report Form, eCRF)

Collection of data will be performed using an electronic Case Report Form (eCRF), which will include track changes monitoring (i.e., recording the changes made and details of the user that made these changes). The eCRF will be filled by blinded investigators with a personal system of access by username and password. Participants will also be given access to the eCRF to directly fill the study questionnaires, which will be reviewed by the study investigators.

The eCRF will be developed by BioClever 2005 S.L.U., after proper agreement with the sponsor.

Electronic devices will be used to collect the study data during home visits and telephone visits.

Paper questionnaires will be made available to participants without access to electronic devices or difficulties to use them, and they will also be available in case of connection problems. Data of paper questionnaires will be filled by participants and study staff will introduce it to the eCRF. Data collected will be reviewed by the investigator in the follow-up visits and collection of paper forms will be performed at day 7 by study staff and at day 15 by study staff or courier. The paper source will be verified by the study monitor.

### 11.5 Data Management

**A** data management system will be set up and procedures will be implemented to warrant homogenization, traceability, and data quality. Data will be entered in a study-specific eCRF. Quality control procedures will be put in place for data checking. Rigorous consistency checks will be created in order to reduce errors during data entry.

Data management will be performed by BioClever 2005 S.L.U., after proper agreement with the sponsor.

Different identification sources will be involved in the study, and candidates will volunteer in a study website that will collect candidate name, surname, address and contact information. These data will be centralized in order to avoid informal personal data transfers between the different services involved, and will only be accessible to the investigators and study staff for the purposes of identification and contact. These data will be separated from the study eCRF.

In the study eCRF data will be collected and stored in a dissociated manner, without including any personal data. This database will be accessible to the sponsor, the data management team, the investigators, the study staff with data entry privileges and the participants. Participants will only be granted access to their own symptoms and safety diary card. The tools used to identify individuals may have individual identifiers, but this information will only be associated with an identification number (i.e., participant ID). This information will uniquely identify participants and will be associated with the rest of the captured data for the study.

For data safety and audit trail purposes each person using any of the defined study databases will be required to define clear data access. Individual user/password codes will be available for each person with access privileges and different roles will be established for data entry and/or revision.

Data collected through the eCRF will be stored in a study database, that will be hosted at a secure data center with appropriate series of protocols to test and maintain network security, and to provide access management policies for network drives, databases and remote access.

Data management team and investigators will be the only ones to access the database. The backup of the data will be done on a timely basis. The final data for the analysis will be placed on the FLS server and will be anonymous; If information that could enable to identify individuals has to be stored, used

or shared, it will be encrypted. Consequently, those receiving the final data for analysis will not have access to any information that might help to physically identify individuals.

# 12 ETHICS

## 12.1 General Considerations

The clinical trial will be conducted according to the principles of the Declaration of Helsinki, Fortaleza, Brazil, October 2013.

This study will be conducted according to Spanish regulations regarding clinical studies without medicines (Orden SAS/3470/2009) and biomedical investigations (Law 14/2007 of biomedical investigation and the Royal Decree 1716/2011). The required documentation prior to the start will be:

- Protocol acceptance by the sponsor and the coordinating investigator
- Protocol approval by the Ethics Committee.

All participants will be guaranteed continued medical and nursing supervision throughout the duration of the study.

This study will conform to the standards of GCP published by ICH (E6 R2). Confidentiality requirements will follow the required Data Protection legislation (see section 13).

## 12.2 Participant Information Sheet and Informed Consent

The investigator will inform the candidates of the nature, duration, and purpose of this study and, in addition, of all the inconveniences and obstacles that, if any, can be expected. In addition, information will be provided to the participant. Subjects must have the legal capacity to give their consent and exercise their freedom of decision.

In order to minimize the transfers of COVID-19 patients, given the risk of contagion that this would entail, eligibility will be checked initially by phone before scheduling a hospital visit. An authorization from the candidates will be obtained over the phone to access their shared medical records (*Història Clínica Compartida de Catalunya HC3*) and check their suitability for the study. Candidates identified through the website will have authorized to obtain personal data and access to medical records when registering at the website. A confirmation e-mail including the study privacy policy will be sent.

During the inclusion/baseline visit, candidates will be informed in person of study details and the informed consent will be obtained (i.e., subjects will sign the informed consent form). The investigator will keep a call record of the informed consent process.

In the substudy participants, substudy informed consent will be obtained.

# 13 DATA HANDLING AND RECORD KEEPING

## 13.1 Data Handling

The processing of the data will be subject to current legislation as regards data protection (LOPD, The Organic Law 3/2018 of 5 December on the Protection of Personal Data and the Guarantee of Digital Rights complementary to the Regulation (EU) 2016/679 of the European Parliament and of the Council of 27 April 2016, on the protection of natural persons with regard to the processing of personal data and on the free movement of such data).

The eCRF and data management will be performed by BioClever 2005 S.L.U., after proper agreement with the sponsor, where the sponsor (FLS and HUGTiP) will be the data controller and BioClever 2005 S.L.U. will be data processor. Data servers will be located in Europe.

Data transmitted to third countries and other countries will in no case contain personal data. In the event that such transfer occurs, it will be for the same purposes of the study described and ensuring confidentiality at least to the level of protection of the law in Spain.

The participant will be identified in the records by the corresponding unique participant ID. The participant is to be guaranteed anonymity and is to be informed that all communication will take place between him/her and the investigator and not the sponsor of the study.

## 13.2 Record Keeping

### *13.2.1 Investigator File and Document Retention*

The investigator must keep the investigator file with the proper and accurate records to enable the study to be fully documented and data subsequently verified.

The investigator's study file will contain the protocol and its amendments, CRFs, questionnaires' forms, EC approval samples of the patient information sheet and informed consent, staff curriculum, signatures' delegation log and listing of subjects, as well as other appropriate documents and correspondence.

Clinical source documents from subjects (usually predefined by the project to record key efficacy and safety parameters or documents that are not in the clinical record of the hospital) will be filed when necessary indicating the participant ID without personal data.

The investigator should retain these documents at least five years, provided that the sponsor does not express another period.

### *13.2.2 Source Documents and Basic Data*

Participation in the study will be included on the participant medical records, including assigned participant ID and identification of the different study visits that will take place throughout the study. At the end of the study, a copy of the eCRF will be placed on the site.

# 14 FINANCING AND INSURANCE

## 14.1 Source of Financing

The funding source is the Fundació FLS de Lluita contra la Sida, les Malalties Infeccioses i la Promoció de la Salut i La Ciència, Hospital Germans Trias i Pujol and Grifols.

## 14.2 Insurance Policy

The study sponsor has a policy of liability insurance. The sponsor shall extend this policy or another with equivalent coverage until the end of the trial. This policy also covers the responsibilities of the sponsor, the principal and his/her collaborators, as well as the hospital or site where they carry out the study.

## 15 PUBLICATION POLICY

The sponsor and the principal investigator aim to publish the results of this study in international peer reviewed journals.

## 16 REFERENCES


1. WHO Interim guidance 13 August 2020. https://www.who.int/emergencies/diseases/novel-coronavirus-2019?gclid=CjwKCAjwydP5BRBREiwA-qrCGrDAeovgOiCboUh2dKJhptzKhJd9Tkha3iH1ZOiKAfe9nOkDggkXjxoC9rsQAvD_BwE
2. Recommendations for Investigational COVID-19 Convalescent Plasma. US FDA. Published May 1, 2020. Accessed July 27th, 2020. https://www.fda.gov/vaccines-blood-biologics/investigational-newdrug-ind-or-deviceexemption-ide-process-cber/recommendations-investigational-covid-19-convalescent-plasma
3. Nguyen AA, Habiballah SB, Platt CD, Geha RS, Chou JS, McDonald DR. Immunoglobulins in the treatment of COVID-19 infection: Proceed with caution!. *Clin Immunol*. 2020;216:108459. doi:10.1016/j.clim.2020.108459
4. Li L, Zhang W, Hu Y, et al. Effect of Convalescent Plasma Therapy on Time to Clinical Improvement in Patients With Severe and Life-threatening COVID-19: A Randomized Clinical Trial [published online ahead of print, 2020 Jun 3]. *JAMA*. 2020;e2010044. doi:10.1001/jama.2020.10044
5. Liu, S. T.H., Lin, H.-M. et al. Convalescent plasma treatment of severe COVID-19: A marched control study. *MedRxiv,* 2020.05.20.20102236. https://doi.org/10.1101/2020.05.20.20102236
6. Joyner, M. J., Klassen, S. A., Senefeld, J., et al. Evidence favouring the efficacy of convalescent plasma for COVID-19 therapy. *MedRxiv*, 2020.07.29.20162917. https://doi.org/10.1101/2020.07.29.20162917
7. Tanne JH. Covid-19: FDA approves use of convalescent plasma to treat critically ill patients. *BMJ*. 2020;368:m1256. Published 2020 Mar 26. doi:10.1136/bmj.m1256.
8. Valk SJ, Piechotta V, Chai KL, et al. Convalescent plasma or hyperimmune immunoglobulin for people with COVID-19: a rapid review. *Cochrane Database Syst Rev*. 2020;5(5):CD013600. Published 2020 May 14. doi:10.1002/14651858.CD013600
9. Joyner MJ, Bruno KA, Klassen SA, et al. Safety Update: COVID-19 Convalescent Plasma in 20,000 Hospitalized Patients [published online ahead of print, 2020 Jul 19]. *Mayo Clin Proc*. 2020;doi:10.1016/j.mayocp.2020.06.028
10. COVID-19 treatment guidelines. NIH. https://www.covid19treatmentguidelines.nih.gov/immune-based-therapy/
11. Wan Y, Shang J, Sun S, et al. Molecular Mechanism for Antibody-Dependent Enhancement of Coronavirus Entry. *J Virol*. 2020;94(5):e02015-19. Published 2020 Feb 14. doi:10.1128/JVI.02015-19
12. Mair-Jenkins J, Saavedra-Campos M, Baillie JK, et al. The effectiveness of convalescent plasma and hyperimmune immunoglobulin for the treatment of severe acute respiratory infections of viral etiology: a systematic review and exploratory meta-analysis. *J Infect Dis*. 2015;211(1):80-90. doi:10.1093/infdis/jiu396
13. Casadevall A, Pirofski LA. The convalescent sera option for containing COVID-19. *J Clin Invest*. 2020;130(4):1545-1548. doi:10.1172/JCI138003
14. van Griensven J, Edwards T, Gallian P; Ebola-Tx Consortium. Convalescent Plasma for Ebola Virus Disease. *N Engl J Med*. 2016;374(25):2500. doi:10.1056/NEJMc1602284
15. WHO Working Group on the Clinical Characterisation and Management of COVID-19 infection. A minimal common outcome measure set for COVID-19 clinical research. *Lancet Infect Dis*. 2020;20(8):e192-e197. doi:10.1016/S1473-3099(20)30483-7
16. Pujadas E, Chaudhry F, McBride R, et al. SARS-CoV-2 Viral Load Predicts COVID-19 Mortality. *medRxiv* 2020.06.11.20128934; doi: https://doi.org/10.1101/2020.06.11.20128934

17. Han A, Poon JL, Powers JH 3rd, Leidy NK, Yu R, Memoli MJ. Using the Influenza Patient-reported Outcome (FLU-PRO) diary to evaluate symptoms of influenza viral infection in a healthy human challenge model. *BMC Infect Dis*. 2018;18(1):353. Published 2018 Jul 28. doi:10.1186/s12879-018-3220-8
18. Powers JH, Guerrero ML, Leidy NK, et al. Development of the Flu-PRO: a patient-reported outcome (PRO) instrument to evaluate symptoms of influenza. *BMC Infect Dis*. 2016;16:1. Published 2016 Jan 5. doi:10.1186/s12879-015-1330-0
19. Kermali, M., Khalsa, R. K., Pillai, K., Ismail, Z., & Harky, A. (2020). The role of biomarkers in diagnosis of COVID-19 – A systematic review. In Life Sciences (Vol. 254, Issue January, p. 117788). https://doi.org/10.1016/j.lfs.2020.117788
20. Pelegrin M, Naranjo-Gomez M, Piechaczyk M. Antiviral Monoclonal Antibodies: Can They Be More Than Simple Neutralizing Agents? *Trends Microbiol* 2015; 23:653–665.
21. Schoofs T, Klein F, Braunschweig M, Kreider EF, Feldmann A, Nogueira L, *et al.* HIV-1 therapy with monoclonal antibody 3BNC117 elicits host immune responses against HIV-1. *Science (80- )* 2016; 352:997–1001.
22. To KK, Tsang OT, Yip CC, et al. Consistent Detection of 2019 Novel Coronavirus in Saliva. *Clin Infect Dis*. 2020;71(15):841-843. doi:10.1093/cid/ciaa149
23. Tu YP, Jennings R, Hart B, et al. Swabs Collected by Patients or Health Care Workers for SARS-CoV-2 Testing. *N Engl J Med*. 2020;383(5):494-496. doi:10.1056/NEJMc2016321
24. McCulloch DJ, Kim AE, Wilcox NC, et al. Comparison of Unsupervised Home Self-collected Midnasal Swabs With Clinician-Collected Nasopharyngeal Swabs for Detection of SARS-CoV-2 Infection. *JAMA Netw Open*. 2020;3(7):e2016382. Published 2020 Jul 1. doi:10.1001/jamanetworkopen.2020.16382

# 17 ANNEXES

## ANNEX 1: DONOR SELECTION, COLLECTION AND PROCESSING PROCEDURES FOR CONVALESCENT MBT PLASMA PREPARATION FROM DONORS RECOVERED FROM COVID-19

### ANNEX 1.1.: CRITERIA FOR ACCEPTING / EXCLUDING DONORS OF WHOLE BLOOD AND BLOOD COMPONENTS

Attached separately, corresponding to the document T-DI-EM-001 of the “Manual de Tècniques” of the BST.

### ANNEX 1.2: UPDATE OF THE PROTOCOL FOR THE SELECTION OF COVID-19 CONVALESCENT PLASMA DONORS

Attached separately, corresponding to the document NT-DI-EM-011 of the “Comissió Transversal d’Hemodonació BST”.

### ANNEX 1.3.: REGISTER OF THE ASSESSMENT OF COVID-19 CONVALESCENT PLASMA DONOR

Attached separately, corresponding to the BST register form.

### ANNEX 1.4.: CONVALESCENT LABEL TEMPLATE E9744

Attached separately, corresponding to the E9744 BST label for MBT convalescent plasma.

### ANNEX 1.5.: METHYLENE BLUE INACTIVATION PROCESS

Attached separately, corresponding to the document T-FR-015 of the “Manual de Tècniques” of the BST.

## ANNEX 2: WHO CLINICAL PROGRESSION SCALE

| Patient State | Descriptor | Score |
|---|---|---|
| Uninfected | Uninfected; no viral RNA detected | 0 |
| Ambulatory mild disease | Asymptomatic; viral RNA detected | 1 |
| | Symptomatic; independent | 2 |
| | Symptomatic; assistance needed | 3 |
| Hospitalised: moderate disease | Hospitalised; no oxygen therapy* | 4 |
| | Hospitalised; oxygen by mask or nasal prongs | 5 |
| Hospitalised: severe diseases | Hospitalised; oxygen by NIV or high flow | 6 |
| | Intubation and mechanical ventilation, $pO_2/FiO_2 \geq 150$ or $SpO_2/FiO_2 \geq 200$ | 7 |
| | Mechanical ventilation $pO_2/FIO_2 < 150$ ($SpO_2/FiO_2 < 200$) or vasopressors | 8 |
| | Mechanical ventilation $pO_2/FiO_2 < 150$ and vasopressors, dialysis, or ECMO | 9 |
| Dead | Dead | 10 |

## ANNEX 3: FLU-PRO© PLUS QUESTIONNAIRE

### Marcar los síntomas actuales

Nos gustaría que nos informara sobre los síntomas que le han afectado en las últimas 24 horas.

Para cada síntoma, marque la casilla situada debajo de la respuesta que mejor describa su experiencia. Marque la casilla que dice "En absoluto", si no ha tenido ese síntoma en las últimas 24 horas

**¿Qué hora es? ______ □ Mañana □ Tarde □ Noche**

**Indique en qué medida ha tenido cada síntoma en las últimas 24 horas.**

| | | En absoluto | Un poco | Algo | Bastante | Muchísimo |
|---|---|---|---|---|---|---|
| | | | | | | |
| 1 | Moqueo o goteo nasal | ☐ | ☐ | ☐ | ☐ | ☐ |
| 2 | Congestión nasal o nariz taponada | ☐ | ☐ | ☐ | ☐ | ☐ |
| 3 | Presión sinusal | ☐ | ☐ | ☐ | ☐ | ☐ |
| | | | | | | |
| 4 | Picor de garganta | ☐ | ☐ | ☐ | ☐ | ☐ |
| 5 | Molestias o dolor de garganta | ☐ | ☐ | ☐ | ☐ | ☐ |
| 6 | Dificultad para tragar | ☐ | ☐ | ☐ | ☐ | ☐ |
| | | | | | | |
| 7 | Ojos llorosos o acuosos | ☐ | ☐ | ☐ | ☐ | ☐ |
| 8 | Molestias o dolor de ojos | ☐ | ☐ | ☐ | ☐ | ☐ |
| 9 | Ojos sensibles a la luz | ☐ | ☐ | ☐ | ☐ | ☐ |
| | | | | | | |
| 10 | Dificultad para respirar | ☐ | ☐ | ☐ | ☐ | ☐ |
| 11 | Congestión en el pecho | ☐ | ☐ | ☐ | ☐ | ☐ |
| 12 | Opresión en el pecho | ☐ | ☐ | ☐ | ☐ | ☐ |
| 13 | Tos seca | ☐ | ☐ | ☐ | ☐ | ☐ |
| 14 | Tos productiva o con flemas | ☐ | ☐ | ☐ | ☐ | ☐ |
| | | | | | | |
| 15 | Sintió nauseas (con la sensación de que quería devolver) | ☐ | ☐ | ☐ | ☐ | ☐ |
| 16 | Dolor de estómago | ☐ | ☐ | ☐ | ☐ | ☐ |
| | | | | | | |
| 17 | Se sintió mareado | ☐ | ☐ | ☐ | ☐ | ☐ |

| | | | | | | |
|---|---|---|---|---|---|---|
| 18 | Congestión de cabeza | ☐ | ☐ | ☐ | ☐ | ☐ |
| 19 | Dolor de cabeza | ☐ | ☐ | ☐ | ☐ | ☐ |
| 20 | Falta de apetito | ☐ | ☐ | ☐ | ☐ | ☐ |
| 21 | Dormir más de lo habitual | ☐ | ☐ | ☐ | ☐ | ☐ |
| 22 | Dolores o molestias en el cuerpo | ☐ | ☐ | ☐ | ☐ | ☐ |
| 23 | Débil o cansado | ☐ | ☐ | ☐ | ☐ | ☐ |
| 24 | Escalofríos o tiritona | ☐ | ☐ | ☐ | ☐ | ☐ |
| 25 | Sintió frío | ☐ | ☐ | ☐ | ☐ | ☐ |
| 26 | Sintió calor | ☐ | ☐ | ☐ | ☐ | ☐ |
| 27 | Sudores | ☐ | ☐ | ☐ | ☐ | ☐ |

*** En absoluto (0), Un poco (1), Algo (2), Bastante (3), Muchísimo (4). Si alguno de los síntomas es igual a Muchísimo (4) registrado por primera vez, COORDINACIÓN recibirá una alerta (por email) para evaluar mediante una visita telefónica la programación de una visita presencial (VISITA DOMICILIARIA EXTRA)**

**En las últimas <u>24 horas</u>, ¿<u>con qué frecuencia</u> ha tenido alguno de los síntomas siguientes?**

| | | Nunca | Rara vez | Algunas veces | A menudo | Siempre |
|---|---|---|---|---|---|---|
| 28 | Estornudos | ☐ | ☐ | ☐ | ☐ | ☐ |
| 29 | Tos | ☐ | ☐ | ☐ | ☐ | ☐ |
| 30 | Tosió mocos o flemas | ☐ | ☐ | ☐ | ☐ | ☐ |

***Nunca (0), Rara vez (1), Algunas veces (2), A menudo (3), Siempre (4). Si alguno de los síntomas es igual a Siempre (4) registrado por primera vez, COORDINACIÓN recibirá una alerta (por email) para evaluar mediante una visita telefónica la programación de una visita presencial (VISITA DOMICILIARIA EXTRA)**

| | | 0 veces | 1 vez | 2 veces | 3 veces | 4 o más veces |
|---|---|---|---|---|---|---|
| 31 | ¿Cuántas veces ha vomitado? | ☐ | ☐ | ☐ | ☐ | ☐ |
| 32 | ¿Cuántas veces ha tenido diarrea? | ☐ | ☐ | ☐ | ☐ | ☐ |

***En las últimas 24h, ¿ha tenido alguno de los síntomas siguientes?***

| | | No | Si |
|---|---|---|---|
| 33 | Pérdida del olfato | ☐ | ☐ |
| 34 | Pérdida del gusto | ☐ | ☐ |

***No (0), Si (1)**

## ANNEX 4: ASSESSMENT OF SAFETY

### Safety Diary Card

OTROS SÍNTOMAS:

**¿Ha notado alguno de los siguientes síntomas en las últimas 24 horas?**

| | **En absoluto** | **Un poco** | **Algo** | **Bastante** | **Muchísimo** |
|---|---|---|---|---|---|
| Dolor local en el lugar de infusión | | | | | |
| Inflamación lugar de infusión | | | | | |
| Erupción cutánea | | | | | |
| Prurito o Picor | | | | | |

*** En absoluto (0), Un poco (1), Algo (2), Bastante (3), Muchísimo (4). Si se registra alguno de estos síntomas por primera vez, COORDINACIÓN recibirá una alerta (por email) para evaluar mediante una visita telefónica dicha sintomatología y su gravedad.**

**Aparte de los síntomas preguntados anteriormente, ¿ha notado algún otro síntoma?:** □ NO□ SI

**En caso afirmativo, ¿qué otro/s síntomas ha notado?**

| **SÍNTOMA** | **Fecha y hora de inicio** | **Fecha y hora de fin** | **Severidad** | | | |
|---|---|---|---|---|---|---|
| | | | **Un poco** | **Algo** | **Bastante** | **Muchísimo** |
| | | | | | | |
| | | | | | | |
| | | | | | | |

### Telephone Safety Assessment

EVENTOS ADVERSOS:

En caso de ser un evento grave o inesperado, llamar a COORDINACIÓN y completar el apartado I del documento de SAE en papel, fotografiarlo y enviarlo per baliza y/o correo electrónico a safety@fls-rs.com

| **Tipo de EVENTO ADVERSO** | (0) NO (1) SI | **Fecha y hora de inicio** | **Fecha y hora de fin** | **Grado 1-5 (*)** | **Relacionado (SI/NO)** | **Decisión investigador** |
|---|---|---|---|---|---|---|
| Erupción cutánea | | | | | | |
| Prurito o Picor | | | | | | |
| Dolor local en el lugar de infusión | | | | | | |
| Inflamación lugar de infusión | | | | | | |
| Fiebre | | | | | | |
| Cefalea | | | | | | |
| Náusea | | | | | | |
| Vómito | | | | | | |

| | | | | | | |
|---|---|---|---|---|---|---|
| Diarrea | | | | | | |
| Dolor abdominal | | | | | | |
| Dolor muscular o articular | | | | | | |
| Tos | | | | | | |
| Disnea | | | | | | |
| Fatiga | | | | | | |
| Mareo | | | | | | |
| Somnolencia | | | | | | |
| Otro: | | | | | | |

*** Grado 1 = Leve, 2=Moderado, 3=Severo, 4=potencialmente mortal, 5= Muerte. Guía de soporte para puntuar grados estará disponible para todo el personal médico del estudio**

## ANNEX 5: HEMOVIGILANCE: DETECTION, ASSESSEMENT AND REGISTER OF INCIDENTS RELATED TO TRANSFUSION

Attached separately.

**ANNEX 6: WEB CONTENT**

Attached separately.

## ANNEX 7: COMPOSITION, RESPONSIBILITIES AND FLOW CHART FOR DSMB

Attached separately.

## ANNEX 8: LIST OF PARTICIPATING SITES

Attached separately.

Version 1.0 18th September 2020

| Site | Principal Investigator | Status |
|---|---|---|
| Hospital Germans Trias i Pujol | Oriol Mitjà | Submitted to EC. |
| Hospital Universitari de Bellvitge | Pending | Agreement in process. |
| Hospital de Sant Joan Despí Moisès Broggi | Pending | Agreement in process. |
| Hospital Clínic de Barcelona | Pending | Agreement in process. |

## ANNEX 9: PARTICIPANT ID CARD

| | |
|---|---|
| **Información para contacto en caso de emergencia:**<br><br>Médico del estudio: ……………………………………………<br><br>Hospital: ……………………………………………<br><br>Teléfono de contacto: ………………………….. | **TARJETA DE IDENTIFICACIÓN**<br><br>**Código: COnV-ert**<br><br>Sr/Sra.<br><br>……………………………………………………<br><br>ID Participante<br><br>…………………………………………………… |

| | |
|---|---|
| Apreciado participante:<br><br>Por favor, lleve consigo esta tarjeta e informe a todos los médicos que le visiten durante y tras su participación en este estudio.<br><br>Informe también al equipo investigador si tuviese alguna visita planificada o inesperada en otro hospital o consulta durante el estudio.<br><br>Muchas gracias. | **Información para contacto con el equipo investigador:**<br><br>Si, en una emergencia, fuera necesario saber si el participante ha recibido plasma convaleciente o placebo; o si tuviera cualquier tipo de pregunta, por favor, contacte con:<br><br>Investigadores: Andrea Alemany, Marc Corbacho.<br><br>Dirección: Hospital Germans Trias i Pujol, Badalona.<br><br>T: 611 69 66 79 |

# Convalescent Methylene Blue Treated (MBT) Plasma for Early Treatment in Non-hospitalised Mild or Moderate COVID-19 Patients: a Randomized Double Blind Study (COnV-ert)

Version 5.0, 10th December 2020

Sponsors:

Fundació FLS de Lluita contra la Sida, les Malalties Infeccioses
i la Promoció de la Salut i la Ciència
Hospital Universitari Germans Trias i Pujol
Carretera de Canyet s/n
08916 Badalona (Barcelona)

Hospital Germans Trias i Pujol, Institut Català de la Salut
Carretera de Canyet s/n
08916 Badalona (Barcelona)


Coordinating Investigator:

Oriol Mitjà, MD, PhD
Hospital Universitari Germans Trias i Pujol



The information contained in this document is confidential and must not be revealed to third persons without prior authorization as contemplated by Law.


This study will be conducted under the conditions described in this protocol and in compliance with the International Council for Harmonisation of Technical Requirements for Pharmaceuticals for Human Use (ICH) Good Clinical Practice (GCP) and all applicable regulatory requirements.

SIGNATURES

The Coordinating Investigator and the Sponsors of the study:

Non-hospitalised mild or moderate COVID-19 Patients: a Randomized Double Blind Study (COnV

Declare that this study will be conducted in compliance with the protocol, Good Clinical Practices (GCP) published by the International Conference of Harmonization Guideline (ICH), and the applicable regulatory requirements.

Modifications to this protocol must be submitted prior agreement of the Principal Investigator and Sponsor.

Coordinating Investigator: Oriol Mitjà, MD, PhD

Signature and Date: ______________________________

Sponsor: Bonaventura Clotet, MD, PhD

President of the Fundació FLS de Lluita contra la Sida, les Malalties Infeccioses i la Promoció de la Salut i La Ciència

Signature and Date: ______________________________

Sponsor: Jordi Ara, MD, PhD

Managing Director of the Àrea Metropolitana Nord, Hospital Germans Trias i Pujol, Institut Català de la Salut

Signature and Date: ______________________________

# 1 GENERAL INFORMATION

## 1.1 Title

Convalescent Methylene Blue Treated (MBT) Plasma for Early Treatment in Non-hospitalised Mild or Moderate COVID-19 Patients: a Randomized Double Blind Study (COnV-ert)

## 1.2 Code

COnV-ert

## 1.3 Protocol Version and Date

Version 5.0, 10th December 2020

Any modification of the protocol must also bear the amendment number and date.

## 1.4 Sponsors

This study will be sponsored by two different institutions:

Fundació FLS de Lluita contra la Sida, les Malalties Infeccioses i la Promoció de la Salut i La Ciència
Hospital Universitari Germans Trias i Pujol
Carretera de Canyet s/n
08916 Badalona (Barcelona)

Person authorized by the sponsor to sign the protocol and amendments: Bonaventura Clotet, President

Hospital Germans Trias i Pujol, Institut Català de la Salut
Carretera de Canyet s/n
08916 Badalona (Barcelona)

Person authorized by the sponsor to sign the protocol and amendments: Jordi Ara, Managing Director

## 1.5 Coordinating Investigator

Oriol Mitjà, MD, PhD
Fundació FLS de Lluita contra la Sida, les Malalties Infeccioses i la Promoció de la Salut i La Ciència
Hospital Universitari Germans Trias i Pujol
Carretera de Canyet s/n
08916 Badalona (Barcelona)
omitja@flsida.org

## 1.6 Clinical Research Organization

The study monitoring, regulatory submission to EC and other study tasks will be performed by the following Clinical Research Organization (CRO):

FLS-Research Support

Fundació FLS de Lluita contra la Sida, les Malalties Infeccioses i la Promoció de la Salut i La Ciència
Hospital Universitari Germans Trias i Pujol
Carretera de Canyet s/n
08916 Badalona
Phone +34 93 497 84 14
info@fls-rs.com

## 1.7 Sites and Investigators

This is a multiple site study. The list of investigators and participating sites may be found in ANNEX 8

## 1.8 Technical Services and Institutions Involved

Blood inflammatory markers tests will be assessed at the central laboratories of Hospital Universitari Germans Trias i Pujol, Hospital Universitari de Bellvitge, Hospital Moisès Broggi de Sant Joan Despí, Hospital Sant Bernabé de Berga and Hospital Sant Joan de Déu de Manresa

ABO compatibility test will be assessed at the Banc de Sang i Teixits (BST) Hematology Department

Quantitative measurement of SARS-CoV-2 viral load will be assessed at the Clinical genetics Service of the Hospital Universitari Germans Trias i Pujol (principal investigator: Ignasi Blanco).

Quantitative measurement of neutralizing antibodies against SARS-CoV-2 in convalescent MBT plasma

Blanco).

Inclusion/baseline hospital visits will
(Hospitalització domiciliària)

Electronic case report form (eCRF), data management and statistics will be performed by BioClever 2005 S.L.U., (contact person: Mireia Bonet, T. 93 408 63 88)

Information regarding additional key personnel and organizations involved in the conduct of the study, including names and contact details of participating investigators, monitors, clinical laboratories, technical departments and/or institutions, as well as information on members of additional study committees, will be found in the study files of the sponsor and at the investigator sites.

Investigators and study staff will receive training in appropriate individual site training session(s).

# 1. TABLE OF CONTENTS

1 GENERAL INFORMATION 3
1.1 TITLE 3
1.2 CODE 3
1.3 PROTOCOL VERSION AND DATE 3
1.4 SPONSORS 3
1.5 COORDINATING INVESTIGATOR 3
1.6 CLINICAL RESEARCH ORGANIZATION 3
1.7 SITES AND INVESTIGATORS 4
1.8 TECHNICAL SERVICES AND INSTITUTIONS INVOLVED 4
1. TABLE OF CONTENTS 5
2. STUDY SUMMARY 10
3. ABBREVIATIONS 17
2 BACKGROUND INFORMATION 19
2.1 CONVALESCENT PLASMA 19
2.1.1 The use of Convalescent Plasma as treatment in infectious diseases 19
2.1.2 Name and Description of the Investigational Plasma 19
2.1.3 Relevant Findings from Nonclinical and Clinical Trials 19
2.1.4 Safety 20
2.1.5 Justification for the Route of Administration, Dosage, Dosage Regimen, and Treatment Periods 20
2.2 MONITORING EFFICACY OF TREATMENT 21
2.2.1 Virologic Outcomes 21
2.2.2 Clinical Outcomes 21
2.2.3 Inflammatory Markers 22
2.2.4 Quantification of Neutralizing Antibodies Against SARS-CoV-2 22
2.2.5 Sample Collection Method 23
2.3 HYPOTHESES 23
3 STUDY OBJECTIVES 25
3.1 PRIMARY OBJECTIVES 25
3.2 SECONDARY OBJECTIVES 25
4 TRIAL DESIGN 26
4.1 TYPE OF TRIAL 26
4.2 DESCRIPTION OF THE DESIGN 26

4.3 STUDY ENDPOINTS 28
4.3.1 Primary endpoint(s) 28
4.3.2 Secondary endpoints 28
4.4 MEASURES TO AVOID BIAS 28
4.4.1 Randomization 28
4.4.2 Stratification 29
4.4.3 Blinding 29
4.4.4 Unblinding 29
4.4.5 Identification of Subjects 29
4.5 END OF STUDY 29
4.6 SOURCE DATA 30
5 STUDY INVESTIGATIONAL PRODUCTS 31
5.1 EXPERIMENTAL AND CONTROL INVESTIGATIONAL PRODUCTS 31
5.2 ARM DESCRIPTION 31
5.3 SUPPLY, PACKAGING, LABELLING AND STORAGE 31
5.3.1 Supply 31
5.3.2 Packaging and Labelling 31
5.4 DOSE, INTERVAL, ROUTE AND METHOD OF ADMINISTRATION 32
5.4.1 Dose, Interval, and Route 32
5.4.2 Administration and Timing of Investigational Plasma for Each Subject 32
5.5 MODIFICATION OF THE TREATMENT REGIMEN 32
5.6 PRIOR AND CONCOMITANT TREATMENTS 32
5.7 ACCOUNTABILITY PROCEDURES FOR INVESTIGATIONAL PRODUCT 32
5.8 TREATMENT COMPLIANCE 33
6 SELECTION AND WITHDRAWAL OF SUBJECTS 34
6.1 STUDY POPULATION 34
6.2 INCLUSION CRITERIA 34
6.3 EXCLUSION CRITERIA 34
6.4 SUBJECT WITHDRAWAL CRITERIA 35
6.4.1 Early Subject Withdrawal 35
6.4.2 Medical Approach to Withdrawal 35
6.4.3 Follow-up after Early Withdrawal 35
6.4.4 Replacement of Participants 35
6.4.5 Pre-randomization Loses 35

| | | |
|---|---|---|
| 7 | STUDY CONDUCT AND ASSESSMENT OF RESPONSE | 36 |
| 7.1 | STUDY VISITS AND PROCEDURES | 36 |
| 7.1.1 | Recruitment procedures | 36 |
| 7.1.2 | Screening | 36 |
| 7.1.3 | Inclusion/baseline | 36 |
| 7.1.4 | Day 3 | 38 |
| 7.1.5 | Day 7 | 38 |
| 7.1.6 | Day 14 | 38 |
| 7.1.7 | Day 28 | 39 |
| 7.1.8 | Day 60 (End-of-Study) | 39 |
| 7.1.9 | Extra home visit (unscheduled visit) | 39 |
| 7.1.10 | | 39 |
| 7.2 | CLINICAL PROGRESSION SCALES | 39 |
| 7.2.1 | WHO Clinical Progression Scale | 39 |
| 7.2.2 | FLU-PRO® PLUS Questionnaire | 40 |
| 7.2.3 | Assessment of Safety | 40 |
| 7.3 | DESCRIPTION OF LABORATORY TESTS AND PROCEDURES | 41 |
| 7.4 | STUDY VARIABLES | 42 |
| 7.5 | SCHEDULE OF PROCEDURES | 45 |
| 8 | ADVERSE EVENTS AND INCIDENTS | 46 |
| 8.1 | SAFETY PARAMETERS | 46 |
| 8.2 | DEFINITIONS | 46 |
| 8.3 | ADVERSE EVENTS ASSESSMENT | 47 |
| 8.3.1 | Seriousness | 47 |
| 8.3.2 | Intensity | 47 |
| 8.3.3 | Causality | 47 |
| 8.3.4 | Expectedness | 48 |
| 8.3.5 | Duration | 48 |
| 8.3.6 | Action Taken | 48 |
| 8.3.7 | Outcome | 48 |
| 8.4 | TIME FRAME FOR RECORDING ADVERSE EVENTS AND INCIDENTS | 49 |
| 8.5 | DOCUMENTATION RELATED TO ADVERSE EVENTS AND INCIDENTS | 49 |
| 8.6 | PREGNANCY | 49 |
| 8.7 | PROCEDURE FOR ADVERSE EVENT REPORTING | 49 |

8.7.1 Investigator 49
8.7.2 Sponsor 50
8.8 DATA AND SAFETY MONITORING BOARD 50
9 STATISTICAL ANALYSIS 51
9.1 METHODS 51
9.1.1 Descriptive Analysis 51
9.1.2 Bivariate Analysis 51
9.1.3 Multivariate Analysis 51
9.2 ANALYSIS POPULATION 51
9.3 SAMPLE SIZE 52
9.4 INTERIM ANALYSES 52
9.5 DEVIATION OF STATISTICAL PLAN 52
10 DIRECT ACCESS TO SOURCE DATA/DOCUMENTS 53
11 QUALITY CONTROL AND QUALITY ASSURANCE 54
11.1 STUDY MONITORING 54
11.2 PROTOCOL DEVIATIONS 54
11.3 AUDITS AND INSPECTIONS 54
11.4 DATA COLLECTION (ELECTRONIC CASE REPORT FORM, ECRF) 55
11.5 DATA MANAGEMENT 55
12 ETHICS 57
12.1 GENERAL CONSIDERATIONS 57
12.2 PARTICIPANT INFORMATION SHEET AND INFORMED CONSENT 57
13 DATA HANDLING AND RECORD KEEPING 58
13.1 DATA HANDLING 58
13.2 RECORD KEEPING 58
13.2.1 Investigator File and Document Retention 58
13.2.2 Source Documents and Basic Data 58
14 FINANCING AND INSURANCE 59
14.1 SOURCE OF FINANCING 59
14.2 INSURANCE POLICY 59
15 PUBLICATION POLICY 60
16 REFERENCES 61
17 ANNEXES 63

8. ANNEX 1: DONOR SELECTION, COLLECTION AND PROCESSING PROCEDURES FOR CONVALESCENT MBT PLASMA PREPARATION FROM DONORS RECOVERED FROM COVID-19 63

9. ANNEX 1.1.: CRITERIA FOR ACCEPTING / EXCLUDING DONORS OF WHOLE BLOOD AND BLOOD COMPONENTS 63

10. ANNEX 1.2: UPDATE OF THE PROTOCOL FOR THE SELECTION OF COVID-19 CONVALESCENT PLASMA DONORS 63

11. ANNEX 1.3.: REGISTER OF THE ASSESSMENT OF COVID-19 CONVALESCENT PLASMA DONOR 63

12. ANNEX 1.4.: CONVALESCENT LABEL TEMPLATE E9744 63

13. ANNEX 1.5.: METHYLENE BLUE INACTIVATION PROCESS 63

14. ANNEX 2: WHO CLINICAL PROGRESSION SCALE 64

**15.** ANNEX 3: FLU-PRO© PLUS QUESTIONNAIRE 65

16. ANNEX 4: ASSESSMENT OF SAFETY 67

17. ANNEX 5: HEMOVIGILANCE: DETECTION, ASSESSEMENT AND REGISTER OF INCIDENTS RELATED TO TRANSFUSION 69

18. ANNEX 6: WEB COENT 70

19. ANNEX 7: COMPOSITION, RESPONSIBILITIES AND FLOW CHART FOR DSMB 71

20. ANNEX 8: LIST OF PARTICIPATING SITES 72

21. ANNEX 9: PARTICIPANT ID CARD 73

## 2. STUDY SUMMARY

| |
|---|
| Title of Study: Convalescent Methylene Blue Treated (MBT) Plasma for Early Treatment i Non-hospitalised Mild or Moderate COVID-19 Patients: a Randomized Double Blind St (COnVert) |
| Study code: COnVert |
| Study Objectives:<br><br>Primary Objectives<br><br>1. Assess the therapeutic potential of early administration of convalescent MBT pl in reducing the rate of hospitalization at day 28 in non-hospitalised mild or moderate COVID-19 patients.<br>2. Assess the therapeutic potential of early administration of convalescent MBT plasm in reducing SARS-CoV-2 viral load at day 7, measured by quantitative RT-PCR (RT qPCR) in non-hospitalised mild or moderate COVID-19 patients.<br><br>Secondary Objectives<br><br>1. Assess the therapeutic potential of early administration of convalescent MBT pl in reducing WHO Clinical progression scale score in non-hospitalised mild or moderate COVID-19 patients.<br>2. Assess the therapeutic potential of early administration of convalescent MBT plasm in reducing the severity of common COVID-19 symptoms, measured with the FL PRO® PLUS scale, in non-hospitalised mild or moderate COVID-19 patients.<br>3. Assess the therapeutic potential of early administration of convalescent MBT pl in reducing the duration of symptoms in non-hospitalised mild or moderate COVI 19 patients.<br>4. Assess the therapeutic potential of early administration of convalescent MBT pl in reducing the mortality at day 60 in non-hospitalised mild or moderate COVID-19 patients.<br>5. Evaluate the safety and tolerability of convalescent MBT plasma in non-hospitalised mild or moderate COVID-19 patients.<br>6. Assess the change from baseline to day 7 of ferritin, prealbumin, interleukin-6, ( D-dimer, C reactive protein (CRP), and leukocytes and lymphocytes counts peripheral blood in non-hospitalised mild or moderate COVID-19 patients receiving convalescent MBT plasma.<br>7. Assess the impact of infused plasma on neutralizing activity by quantifying the c from baseline to day 7 in neutralizing antibodies against SARS-CoV-2 in peripheral blood in non-hospitalised mild or moderate COVID-19 patients receiving convalesce MBT plasma.<br>8. Assess the long-term impact of plasma infusion on humoral immune responses quantifying the change from baseline to day 60 in neutralizing antibodies aga SARS-CoV-2 in peripheral blood in non-hospitalised mild or moderate COVID-19 patients receiving convalescent MBT plasma.<br>9. Compare agreement and SARS-CoV-2 viral load in self-collected middle turbinate (MT) swab and self-collected saliva samples with nasopharyngeal swab sam collected by a healthcare worker |

| |
|---|
| 10. Assess the therapeutic potential of early administration of convalescent MBT pl in reducing SARS-CoV-2 viral load at day 28, measured by quantitative PCR (RT-qPCR) in non-hospitalised mild or moderate COVID-19 patients. |
| Overall Study Design and Description:<br><br>This is a prospective, randomized (1:1), double blind study of Convalescent Anti-SARS-CoV-2 MBT Plasma (also known as convalescent plasma) plus standard medical treatmen versus placebo plus SMT in mild or moderate COVID-19 patients who are non-hospitalised. Subjects with confirmed infection by SARS-CoV-2 will receive SMT plus a total of 200-300 mL of convalescent plasma that has been pathogen-inactivated using MBT or placebo.<br><br>Study candidates will voluntarily express their interest in participating in the study throug the study website or will be offered to participate at the emergency (ER) and outpatient departments (OPD) of the participating hospitals. Candidates registered on the websi be contacted by study physicians by phone to inform them about the study and check th suitability for the study. Suitable candidates will be scheduled an inclusion/baseline v which informed consent will be obtained (i.e., the informed consent will be signed), and eligibility will be confirmed. Candidates identified through ER and OPD department undergo an inclusion/baseline visit, where the informed consent will be obtained eligibility will be checked. A subgroup of eligible candidates from selected study sites will be offered participation in the substudy to assess the immune response and the metho sampling.<br><br>Blood and nasopharyngeal samples will be obtained from all eligible candidates.<br><br>Eligible candidates will be randomized and administered an intravenous (IV) infusion at baseline (convalescent plasma or placebo). Both the investigator and the participant blinded to the study treatment.<br><br>Specifically, subjects randomized to combination convalescent anti-SARS-CoV-2 MBT plasma plus SMT will undergo an ABO compatibility test and will receive a single infusion of 20 300 ml of ABO-compatible convalescent plasma. Subjects randomized to placebo plus will receive a single infusion of 200 to 300 ml of sterile saline solution 0.9%. Infusion will be administered at baseline, using standard procedures for administration of fresh f plasma. Small adults weighing less than 45 kg will receive one infusion of 5 ml of conva plasma or placebo per kilogram of body weight.<br><br>Participants will be trained on the completion of symptoms diary card and safety diary c<br><br>The participants of the substudy will be drawn an extra tube of blood sample and will trained on self-collection of middle turbinate (MT) swabs and saliva, and self-collected samples will be obtained.<br><br>The symptoms and safety diary card will be filled by the participants daily from basel day 14. On follow-up visits on days 3, 7, 14, and 28, all participants will be assessed for and safety outcomes. These visits will be all by telephone except for the day 7 and day visits that will be at home or at hospital, where additional blood samples (only on da 7) and nasopharyngeal swabs will be collected.<br><br>At day 60 visit, all participants will be assessed by telephone for health status outcome. |

| |
|---|
| For the participants of the substudy on day 7, an extra tube of blood sample will be obtai and they will be asked to self-collect MT swabs and saliva. And on day 60, an extra tu blood sample will be obtained during an additional home or hospital visit. |
| Number of Subjects Planned:<br><br>Approximately 474 individuals will be randomized (1:1) with an interim analysis after th 60 subjects (30 in each arm).<br><br>The sample size will be re-assessed upon interim analysis.<br><br>Approximately 135 individuals from selected study sites will be included in the substu assess the immune response and the methods of sampling. |
| Diagnosis and Main Criteria for Eligibility:<br><br>Individuals with a SARS-CoV-2 confirmed infection from different regions of Catalonia. .<br><br><u>Inclusion Criteria</u>:<br><br>1. Adult male or female individuals of<br>2. In women of childbearing potential[1], negative pregnancy test at inclusion/baseline.<br>3. Has confirmed SARS-CoV-2 infection as determined by PCR or validated antigen r diagnostic test[2]<br>4. Symptomatic with mild or moderate COVID-19 with symptoms prior to inclusion/baseline visit.<br>a. Mild COVID-19: Individuals who have any of the common signs an symptoms of COVID-19 (i.e., fever, cough, sore throat, malaise, heada muscle pain) without shortness of breath, dyspnoea, or abnormal chest imaging.<br>b. Moderate COVID-19: Individuals who have evidence of lower respirat disease by clinical assessment or imaging and a saturation of oxyge[illegible]2)<br>5. Willing to comply with the requirements of the protocol and available for follow-up for the planned duration of the study.<br>6. Has understood the information provided and capable of giving informed conse<br><br>[1]A woman will be considered of childbearing potential if not permanently sterilize postmenopausal. Permanent sterilization methods include tubal ligation, hysterectom bilateral oophorectomy. Postmenopausal is defined as 12 months with no menses wit alternative medical cause.<br><br>[2]Panbio™ COVID-19 Ag Rapid Test (Abbott), STANDARD™ Q COVID-19 Ag Test (Roche) or a other CE marketed test for SARS-CoV-2 Ag detection. |
| Diagnosis and Main Criteria for Eligibility:<br><br><u>Exclusion Criteria</u>:<br><br>1. If female, pregnant, breastfeeding, or planning a pregnancy during the study.<br>2. Severe or critical COVID-19: |

a. Severe COVID-19: respiratory frequency >30 breaths per minute, $SpO_2$ <94% on room air at sea level, ratio of arterial partial pressure of oxygen to frac of inspired oxygen ($PaO_2/FiO_2$) <300 mmHg, or lung infiltrates >50%.

b. Critical COVID-19: respiratory failure, septic shock, and/or multiple org dysfunction.

3. Current hospital admission for any cause.
4. History of previous confirmed SARS-CoV-2 infection.
5. History of significantly abnormal liver function (Child Pugh C).
6. History of chronic kidney disease (CKD) stage 4, or need of dialysis treatment.
7. Any preexisting condition that increases risk of thrombosis.
8. History of allergic reactions to blood or plasma products or methylene blue.
9. Known IgA deficiency with anti-IgA antibodies.
10. Medical conditions for which 200-300 ml of intravenous fluid is considere dangerous (i.e., decompensated heart failure or renal failure with fluid overload
11. Inability to consent and/or comply with study requirements, in the opinion of the investigator.
12. Currently participating or planning to participate in any interventional study for treatment of COVID-19 or SARS-CoV-2 infection until day 60.

Investigational Product, Dose and Mode of Administration:

Randomized participants will receive one intravenous (IV) infusion at baseline (conval plasma or placebo) and will continue their SMT for COVID-19 disease, as prescribed by regu physicians.

Experimental arm: Subjects randomized to convalescent anti-SARS-CoV-2 MBT plasma plus SMT will receive one infusion of 200 to 300 ml of ABO-compatible convalescent plasma obtained from a convalescent donor.

Placebo arm: Subjects randomized to placebo plus SMT will receive one infus 200 to 300 ml of sterile saline solution 0.9%.

Duration of Treatment:

The investigational product will be administered by IV infusion at baseline.

Participants will continue their standard medical treatment (SMT) for SARS-CoV-2 infection as prescribed by their regular physician. If applicable, SMT may be modified during the st depending on personal requirements, the severity and progression of the disease, and for hospitalization.

-of-study visit) will be up to 60 days.

Endpoints and Timepoints of Assessment:

<u>Primary Endpoints and Timepoints of Assess</u>ment:

1.
progression) [Time Frame: Up to 28 days after reception of investigational prod

Reduction of SARS-CoV-2 viral load in nasopharyngeal swabs at day 7 after sta treatment, as determined by RT-qPCR. [Time Frame: Up to 7 days after receptio investigational product]

<u>Secondary Endpoints and Timepoints of Assessment</u>:

1. Change in COVID-19 WHO Clinical progression scale score [Time Frame: Up to 6 after reception of investigational product]
2. Change in COVID-19 symptoms severity score, assessed with the COVID-19 daily self score tool (FLU-PRO© PLUS instrument) [Time Frame: Up to 14 days after rece of investigational product]
3. Time to complete resolution of symptoms [Time Frame: Up to 28 days after receptio of investigational product]
4. Death rate [Time Frame: Up to 60 days after reception of investigational produc
5. on the FDA Toxicity Grading Scale for Healthy Adult and Adolescent Volunteer [Time Frame: Up to 28 days after reception of investigational product]
6. Change in inflammatory prognostic markers (ferritin, prealbumin, interleukin 6 (IL-6), D-dimer, C reactive protein (CRP) and leukocyte and lymphocyte counts) [Time Frame: Baseline and day 7 after reception of investigational product]
7. Intergroup comparison of absolute neutralization titers against SARS-CoV-2 in plasma of a subgroup of participants [Time Frame: Baseline and day 7 after recepti investigational product]
8. Change in titers of neutralizing antibodies against SARS-CoV-2 in plasma of a subgroup of participants [Time Frame: Baseline and day 60 after receptio investigational product]
9. Agreement and SARS-CoV-2 viral load of self-collected middle turbinate (MT) swa and saliva samples compared to nasopharyngeal swabs collected by a hea worker on a subgroup of participants [Time Frame: Baseline and day 7 after rece of the investigational product]
10. Reduction of SARS-CoV-2 viral load in nasopharyngeal swabs at day 28 after sta treatment, as determined by RT-qPCR. [Time Frame: Up to 28 days after reception of investigational product].

Study Assessments and Procedures:

COVID-

study through the study website or (2) will be informed of the study at the emergency (ER) or out-patient departments (OPD) from the participating sites.

1. Candidates who register on the study website will sign an authorization for the investigators (physicians) to access their shared medical records (Història Clínica Compartida de Catalunya HC3). Study physicians will call the candidates who registe the website to perform a remote <u>screening</u> visit, where candidates will be informe about the study over the phone, and their suitability for the study will be chec Suitable candidates will be scheduled an <u>inclusion/baseline</u> visit .
2. Candidates identified at the participating sites will be invited to participate by study physicians on-site, and those interested will undergo an <u>inclusion/baseline</u> visit.

In the inclusion/baseline visit, either at the hospital or at home by the Hospital at Home c unit (Hospitalització domiciliària), candidates will be informed in person of study details a the informed consent will be obtained (i.e., subjects will sign the informed consent forr physical examination will be performed, and women of childbearing potential will undei urine pregnancy test. Eligibility will be confirmed on this visit.

Blood samples will be obtained from all eligible candidates. Participants who meet a inclusion and none of the exclusion criteria will be randomized. Evaluation of compatibility using standard guidelines for compatibility procedures in blood transfusi laboratories will be performed in participants assigned to experimental (convalescent pl group.

Nasopharyngeal samples will be obtained from all participants. The participants c substudy will be drawn an extra tube of blood sample and will be trained on self-collection of middle turbinate (MT) swabs and saliva, and self-collected samples will be obtained.

Participants will be also trained on completion of symptoms and safety diary card, which will be filled daily by the participant until the day 14 visit, through an electronic Case Report (eCRF). Paper forms will be available for those participants who are unable to subm using the eCRF, in this scenario data will be collected at follow-up visits. Should any participar report any new symptom with grade 4 at the FLU-PRO® PLUS questionnaire, he or she will contacted by telephone by the study staff to evaluate any possible AE and the need extra home visit. Participants will be also given a contact number to report AEs, to se medical advice, and resolve any question related to the study.

All randomized participants will be administered an IV infusion at baseline (convale plasma or placebo). If an AE develops during infusion, the infusion will be slowed or stoppe

develop, and it will not be restarted. Medicines for transfusion reactions (e.g. paraceta dexchlorpheniramine) may be given as a treatment.

On day 3, participants will be contacted by telephone by study staff for safety and cli follow-up.

On day 7, participants will be visited by study staff either at home or at the hospital, a blood sample will be drawn, and a nasopharyngeal swab will be collected by study staff participants of the substudy will be drawn an extra tube of blood sample and will self-collect middle turbinate (MT) swabs and saliva.

On day 14, participants will be contacted by telephone by study staff for safety and clinic follow-up.

On day 28, participants will be visited at hospital by study staff for safety and clinical fol up, and a nasopharyngeal swab will be collected.

On day 60, participants will be contacted by telephone by study staff to evaluate health statu The participants of the substudy will be scheduled for a hospital or home visit to obtair blood sample.

If the participant is unreachable at phone follow-up visits, clinical progression, as well possible AEs will be assessed through medical record (Història Clínica Compartida, HC3) in order to assess all the study outcomes.

<u>Study samples</u>

Nasopharyngeal swab samples for quantitative measurement of SARS-CoV-2 viral load by RT-qPCR will be obtained prior to infusion at baseline, on day 7 and on day 28.

MT swab and saliva self-collected samples for quantitative measurement of SARS-CoV-2 viral load by RT-qPCR will be obtained prior to infusion at baseline and at day 7 in the partici of the substudy.

Blood samples will be collected prior to infusion at baseline and at day 7 for all partici Inflammatory prognostic markers (ferritin, prealbumin, interleukin 6 (IL-6), D-dimer, C reactive protein (CRP), and leukocytes and lymphocytes counts) will be assessed. Left plasma will be stored for future investigations.

In the participants of the <u>substudy</u> an extra tube will be collected at baseline and day 7, an an extra blood draw will be performed at day 60 to assess neutralizing antibodies a SARS-CoV-2. Leftovers of plasma will be stored for future investigations.

Results of the sample analyses are not necessary for participants to remain in the study, an participants will only be informed of relevant results for their health.

Samples of the infused convalescent anti-SARS-CoV-2 MBT plasma will be obtained befo infusion for quantitative measurement of neutralizing antibodies against SARS-CoV-2.

Statistical Method:

Descriptive statistics will include the number of non-missing observation, mean, standa deviation (SD), median, minimum, and maximum values for the continuous/quantitative or absolute and relative frequency counts and percentages for categorical/qualitative da All analyses will be done with the R statistical package, version 6.3 or higher u significance level of 0.05.

<u>Populations</u>: The primary efficacy analysis will be performed on the intent-to-treat (ITT) population, which will include all randomized participants. If deemed necessary, sens analyses will be performed with the per-protocol (PP) population. Safety will be assesse the safety population, which will include all participants who receive the investigationa product (convalescent plasma or placebo).

<u>Determination of Sample Size</u>:

A sample size of 237 cases per arm would provide the trial with 80% power to detec reduction in hospitalization rate at day 28 after starting the treatment, assuming an exp rate of hospitalization of 15%, allowing a 5% of loss to follow-up

Approximately 150 cases per arm are required to have 80% power to detect a differen 0.5 $\log_{10}$ in the mean reduction of SARS-CoV-2 viral load at a two-
= 0.05, assuming an expected overall standard deviation of 1.5. A 0.5 $\log_{10}$ copies/mL difference in reduction was chosen to represent the minimal threshold for a biologi relevant change for our analyses.

An interim analysis of efficacy and safety variables will be performed after the first 60 su achieve the primary endpoint (i.e., day 28) for the purpose of sample size recalculation. interim analyses will be performed using blinded data, unless otherwise indicated b DSMB.

## 3. ABBREVIATIONS

| | |
|---|---|
| ACE2 | angiotensin converting enzyme 2 |
| ADL | Activities daily life |
| AE | Adverse event |
| CI | Confidence interval |
| COVID-19 | Coronavirus disease 2019 |
| Ct | cycle threshold |
| CTCAE | Common Terminology Criteria for Adverse Events |
| ELISA | Enzyme, linked immunosorbent assay |
| ER | Emergency Department |
| EuroQol | Europe Quality of life |
| FDA | Food and Drug Administration |
| FFP | Fresh frozen plasma |
| FLU-PRO | FLU patient-reported outcome measure |
| GCP | Good clinical practice |
| ICH | International Council for Harmonisation |
| IFA | Indirect Fluorescent Antibody |
| IgA/IgM/IgG | Immunoglobulin A / M / G |
| IL-6 | Interleukin 6 |
| ILI | influenza-like illness |
| ITT | Intention to treat |
| IV | intravenous |
| MBT | Methylene blue treated |
| MERS | Middle East respiratory syndrome |
| ml | millilitre |
| MT | middle turbinate |
| NAT | nucleic acid amplification technology |
| NIH | National Institutes of Health |
| OPD | Out-Patient Department |
| OR | Odds ratio |
| PaO2/FiO2 | arterial partial pressure of oxygen to fraction of inspired oxygen |
| PCR | Polymerase chain reaction |

| | |
|---|---|
| PP | Per protocol |
| qPCR | Quantitative PCR |
| CRP | C-reactive Protein |
| RSV | Respiratory Syncytial Virus |
| RT-PCR | Reverse transcriptase PCR |
| SAE | Serious Adverse Event |
| SARS | Severe acute respiratory syndrome |
| SARS-CoV-2 | Severe acute respiratory syndrome coronavirus 2 |
| SD | Standard Deviation |
| SMT | Standard Medical Treatment |
| Sp02 | Peripheral Oxygen Saturation |
| SUSAR | Serious Unexpected Adverse Reaction |
| TACO | Transfusion-Associated Circulatory Overload |
| TRALI | Transfusion-related acute lung injury |
| UAE | Unexpected adverse event |
| WHO | World Health Organization |

## 2 BACKGROUND INFORMATION

Coronavirus disease 2019 (COVID-19) is a respiratory tract infection caused by a newly emergent coronavirus, severe acute respiratory syndrome coronavirus 2 (SARS-CoV-2), that was first recognized in Wuhan, China, in December 2019. The emergence of COVID-19 has caused a large global outbreak and it is a major public health issue. As of 13 August 2020, data from the World Health Organization (WHO) have shown that more than 20 million confirmed cases have been identified in 216 countries, areas or territories (WHO Interim guidance 13 August 2020) (1).

### 2.1 Convalescent Plasma

#### *2.1.1 The use of Convalescent Plasma as treatment in infectious diseases*

Currently, there are no FDA-approved drugs for the treatment of COVID-19. Definitive clinical trial data is needed to find safe and effective treatments for COVID-19. Vaccine development is progressing at a rapid pace, but widespread vaccine availability is estimated to be at least six months away. There is an urgent need for effective interventions presently. Administration of SARS-CoV-2 neutralizing antibodies is the only form of immunization available in the absence of vaccines or humanized monoclonal antibodies.

Convalescent plasma therapy has been used to treat patients with infections using plasma collected from recently recovered individuals to transfer passive antibody immunity to those who have recently been infected or have yet to be exposed to the virus. Use of convalescent plasma has been studied in outbreaks of other respiratory infections, including the 2003 SARS-CoV-1 epidemic, the 2009-2010 H1N1 influenza virus pandemic, and the 2012 MERS-CoV epidemic (FDA recommendations) (2). Convalescent plasma from donors who have recovered from COVID-19 may contain antibodies to SARS-CoV-2 that might help suppress the virus and modify the inflammatory response. It has been postulated that neutralizing antibodies would prevent SARS-CoV-2 spike protein from attaching to the ACE2 receptor, inhibiting viral entry into the cell (Nguyen et al., 2020) (3).

#### *2.1.2 Name and Description of the Investigational Plasma*

The investigational plasma in this study is Convalescent-severe acute respiratory syndrome coronavirus 2 (SARS-CoV-2) MBT Plasma.

The product is being collected by plasma centres in Spain.

#### *2.1.3 Relevant Findings from Nonclinical and Clinical Trials*

There is already clinical trial data supporting the efficacy of convalescent plasma in the treatment of COVID-19 patients. An open-label, multicentre, randomized clinical trial conducted in China that enrolled 103 participants with laboratory-confirmed COVID-19 severe or life-threatening patients showed higher rates of viral clearance at 72 hours in the convalescent plasma group compared to the control group (87.2% vs 37.5%, respectively) (OR 11.39 [95% CI, 3.91-33.18]; P<0.001), demonstrating association with anti-viral activity of the treatment in patients with COVID-19 (Li et al., 2020) (4). A retrospective case-control study evaluating convalescent plasma conducted in Mount Sinai Hospital in New York City showed by day 14 a clinical worsening in 18% of the convalescent plasma patients vs 24% of the control patients (P=0.17), and a mortality of 13% of the plasma recipients and 24% of the control patients was observed (P=0.04) (Liu et al., 2020) (5). Preliminary data from a study from Mayo Clinic that aggregated patient outcome data from randomized clinical trials, matched control, and case-series studies showed that hospitalized COVID-19 patients transfused with convalescent plasma exhibited a 57% reduction in mortality rate (13%) compared to matched-patients receiving standard treatments (25%; OR: 0.43; P<0.001) (Joyner et al., 2020) (6).

The US FDA has approved the emergency use of convalescent plasma for patients with severe or life-threatening COVID-19 (Tanne, 2020) (7). And there are currently more than 100 ongoing studies evaluating convalescent plasma or hyperimmune immunoglobulin, of which approximately 50 are randomized (https://clinicaltrials.gov/).

Most clinical studies are investigating the use of convalescent plasma in hospitalized patients with severe or critical disease. However, this therapy may have clinical and virologic benefits in the treatment of mild or moderate disease in non-hospitalised patients, especially if given early in the disease course. Therefore, it is important to study the safety and efficacy of COVID-19 convalescent plasma in non-hospitalised patients in clinical trials.

#### *2.1.4 Safety*

Risks of passive administration of convalescent plasma fall into two categories, known and theoretical. Known risks are those associated with transfer of blood substances, which include inadvertent infection with another infectious disease agent and reactions to plasma constituents. With modern blood banking techniques that screen for blood-borne pathogens and match the blood type of donors and recipients, the risks of inadvertently transferring known infectious agents or triggering transfusion reactions are low. Additionally, MBT reduces risk of any pathogen undetected by donor screening and product testing. The most common adverse events are mild allergic or respiratory events (Piechotta et al., 2020) (8). Uncommon (i.e. in <1% of transfusions) but serious risks of convalescent plasma infusion may include transfusion-associated circulatory overload (TACO), transfusion-related acute lung injury (TRALI) and anaphylaxis (Joyner et al., 2020) (NIH Covid-19 treatment guidelines) (9, 10). And this should be a consideration in the risk-benefit assessment.

The theoretical risk is potential for antibody-dependent enhancement (ADE) of infection. ADE can occur in several viral diseases and it is a harmful, exaggerated inflammatory response triggered by antibodies. Previously, ADE has been proposed as an underlying pathogenic mechanism in Dengue haemorrhagic fever (Nguyen et al., 2020) (3). For coronaviruses, several mechanisms for ADE have been described, and there is the theoretical concern that antibodies to one type of coronavirus could enhance infection to another viral strain (Wan et al., 2020) (11). Since the proposed use of convalescent sera in the COVID-19 epidemic would rely on preparations presumably with high titers of neutralizing antibody against the same virus, SARS-CoV-2, ADE may be unlikely. Available evidence from the use of convalescent plasma in patients with SARS and MERS (Mair-Jenkins et al., 2015) (12) and for the treatment of Covid-19, in the Expanded Access Program (EAP), with data from 20.000 hospitalised patients with COVID-19 (Joyner et al., 2020) (9) suggest it is safe. Nevertheless, in convalescent plasma trials, close monitoring to identify any evidence of enhanced infection will be required.

Given that historical and current data on use of convalescent plasma suggest it is safe in coronavirus infection, and the high mortality of COVID-19, particularly in elderly and vulnerable persons, suggests that the benefits of its use in those at high risk for or with early disease outweigh the risks. However, for all cases where convalescent plasma administration is considered, a risk-benefit assessment must be conducted to assess individual variables (Casadevall et al., 2020) (13).

#### *2.1.5 Justification for the Route of Administration, Dosage, Dosage Regimen, and Treatment Periods*

Administration of Convalescent Plasma/Placebo

Subjects in the Convalescent anti-SARS-CoV-2 MBT plasma plus SMT arm, will receive intravenous (IV) Convalescent anti-SARS-CoV-2 MBT plasma according to standard hospital infusion practices for fresh

frozen plasma (FFP). Subjects in the placebo plus SMT arm will receive sterile saline solution 0.9% by intravenous (IV) infusion.

Justification for Selection of Volume of Administration and time of Convalescent Plasma/Placebo

Administration of Convalescent anti-SARS-CoV-2 MBT plasma in this study is consistent with previous experience with the administration of convalescent plasma for other infectious indications (e.g. Ebola) (van Griensven et al., 2016) (14) (Joyner et al., 2020) (9).

Randomized subjects will receive SMT plus one infusion of 200 to 300 ml of ABO-compatible convalescent plasma or placebo. Small adults weighing less than 45 kg will receive one infusion of 5 ml of ABO-compatible convalescent plasma or placebo per kilogram of body weight (to a maximum of 250ml).

## 2.2 Monitoring Efficacy of Treatment

The WHO Working Group on the Clinical Characterisation and Management of COVID-19 Infection have developed a minimum set of common outcome measures for studies on COVID-19. The set includes three elements: a measure of viral burden, a measure of patient survival and a measure of patient progression. Study outcomes have been selected based on these recommendations (WHO Working Group on the Clinical Characterisation and Management of COVID-19 infection, 2020) (15).

### *2.2.1 Virologic Outcomes*

Viral burden is considered an appropriate outcome for monitoring efficacy of treatment in COVID-19 patients. To measure viral burden, quantitative PCR (RT-qPCR) to quantify viral copies is the best measure, with threshold cycle values (Ct) during PCR as an alternative. Quantification of viral b has not proven yet to provide insight into the clinical status of the patient but does provide strong evidence of the presence of the pathogen, and it can be used to measured pathogen burden in response to treatment (WHO Working Group on the Clinical Characterisation and Management of COVID-19 infection, 2020) (15).

Viral load is associated with transmission risk and disease severity in other viral illnesses. And, recently, it is been reported SARS-CoV-2 viral load at diagnosis as an independent predictor of mortality in a large hospitalised cohort (n=1145). There was found a significant independent association between viral load and mortality (hazard ratio 1.07 [95% CI 1.03-1.11], P=0.0014), with a 7% increase in hazard for each log transformed copy per ml (Pujadas et al., 2020) (16). Viral load in COVID-19 might correlate, not only with mortality, but also with infectivity, disease phenotype and morbidity.

### *2.2.2 Clinical Outcomes*

Clinical progression will be measured using different validated scales.

Firstly, the WHO Clinical Progression Scale will be used and will assess hospitalization rate (i.e. who -mortal clinical outcomes (WHO Working Group on the Clinical Characterisation and Management of COVID-19 infection, 2020) (15). This is an ordinal scale to measure clinical progression and recovery based on location and supportive measures used within the healthcare system. It provides a measure of illness severity across a range from 0 (not infected) to 10 (death) with data elements that are rapidly obtainable from clinical records.

Considering that our target population is non-hospitalised, ambulatory participants, and the rate of hospitalisation is relatively low, even in high-risk patients, we selected some other clinical progression scales to help assess clinical outcomes.

Symptom severity will be measured using a validated scale. Severity over time is been considered a clinically meaningful endpoint, particularly in a disease that exhibits such heterogeneous symptomatology.

Symptom severity will be also assessed with the FLU-PRO© PLUS Questionnaire, a daily self-score tool. The COVID-19 version, adapted from FLU-PRO© instrument, consists of 34 items that are answered daily. The FLU-PRO© is a self-administered patient-reported outcome measure (PRO) to quantify symptom severity in influenza and influenza-like illness and it has been tested and used in studies of influenza, influenza-like illness (ILI), respiratory syncytial virus (RSV), rhinovirus, and enterovirus (Han A et al., 2018)(17) (Powers JH et al., 2016)(18).

### 2.2.3 Inflammatory Markers

Biomarkers are quantitative measurements used clinically for many conditions reflecting pathological development. A recent systematic review summarized the following biomarkers with most evidence as predictors for a severe COVID-19 infection (Kermali et al., 2020)(19):

| | |
|---|---|
| C-Reactive Protein (CRP) | Increases |
| Serum amyloid A | Increases |
| Interleukin-6 | Increases |
| Lactate Dehydrogenase | Increases |
| Neutrophil-to lymphocyte ratio | Increases |
| Lymphocyte count | Decreases |
| Platelet count | Decreases |
| D-dimer | Increases |
| Cardiac troponin | Increases |
| Renal biomarkers Urea & creatinine | Increases |

### 2.2.4 Quantification of Neutralizing Antibodies Against SARS-Cov-2

Treatment of infectious diseases with therapeutic antibodies is an emergent field in clinical research. Although the major objective of this strategy is to block viral replication owing the antiviral effect of antibodies, the wide range of immunological functions of antibodies enlarges their potential benefit. In this regard, it has been widely documented that therapeutic antibodies exert a booster effect on humoral responses (Pelegrin et al., 2015)(20). Interestingly, this effect extends beyond the treatment period and may last once the therapeutic antibody has been cleared, suggesting that new specific B cells are being generated during the intervention (Schoofs et al., 2016) (21).

Therefore, we hypothesize that individuals treated with hyperimmune plasma might develop a better humoral response against SARS-CoV-2 and therefore might show higher titers of neutralization in the long-term. To test this hypothesis, we plan to analyse the neutralizing activity of plasma or serum samples obtained in a subgroup of control and treated participants at three different timepoints: day 0 (before plasma infusion), day 7 (to quantify the impact of infused plasma on neutralizing activity) and day 60 (to quantify the long-term impact of plasma infusion on humoral immune responses). The increase in neutralizing titer between day 0 and day 60 will be compared between control and intervention groups to assess the potential booster effect of treatment. Intergroup comparison of absolute neutralization titers at day 0 and 7 will confirm similarity of pre-treatment and on-treatment titers, respectively, in control and treated participants.

Quantification of neutralizing antibodies against SARS-CoV-2 will be performed in blood samples of a subgroup of individuals. Since no data exist on COVID-19 patients regarding the impact of

convalescent plasma on long-term neutralizing activity, the sample size calculation of the subgroup of participants needed will be based on the available data for other infectious diseases. Treatment of HIV infected individuals with a single injection of the broadly neutralizing antibody 3BNC117 induces a significant increase in neutralization titers that is maintained once the therapeutic antibody reaches undetectable plasma levels (Schoofs et al., 2016) (21). Assuming a similar impact on SARS-CoV-2 infected individuals (i.e., similar endpoint differences between groups and similar standard deviation of the measures reported for HIV) a total of 42 (enrolment ratio 1, 21+21) participants would be necessary to reach a statistical power of 80% to observe a difference between the treated and control groups at day 90 (Type I error rate 0.05). Different simulations assuming less favourable scenarios have been also performed. For instance, assuming a reduction of 50% in the difference of the means the sample size would increase to 122 (maintaining the other parameters). Thus 61 and 61 participants from the control and treated groups will be invited to participate in this substudy. This sample size enables to detect differences similar to those reported for HIV infection and is still powered (80%) to identify lower differences (50% reduction in the difference of the means between groups).

#### 2.2.5 Sample Collection Method

Increase diagnostics are urgently needed to contain the spread of COVID-19. Home self-collecting of swabs may increase testing through improving accessibility outside the healthcare system while minimizing exposure risk and personal protective equipment use. This approach could be safe and scalable in the pandemic setting, allowing for early community detection of COVID-19.

Recent data support the validity of non-nasopharyngeal samples for detection of SARS-CoV-2 (To et al., 2020) (22). There is also some data supporting the use of self-collected respiratory swabs (Tu et al., 2020) (23), particularly in symptomatic patients with higher viral loads (McCulloch et al., 2020) (24). Self-collected mid-turbinate samples had a sensitivity of 96,2% (97,5% CI, 87-100%) when compared with nasopharyngeal samples collected by a health care worker (Tu et al., 2020) (23). And self-collected midnasal swabs had a sensitivity of 80% (95% CI, 63-91%) and a specificity of 97,9% (95% CI, 94%-99,5%) when compared with clinician-collected nasopharyngeal swabs for detection of SARS-CoV-2 infection (McCulloch et al., 2020) (24).

In line with available data, nasopharyngeal swabs will be paired with mid-turbinate (MT) swabs at baseline visit to gather further evidence to support clinical performance of self-collected MT swabs.

### 2.3 Hypotheses

This is a prospective, randomized (1:1), double blind study of convalescent-anti-SARS-CoV-2 MBT plasma plus standard medical treatment (SMT) versus placebo plus SMT in mild or moderate COVID-19 patients who are non-hospitalised. Convalescent plasma will be provided to patients to assess reduction of viral load and inflammation, clinical efficacy, safety, and tolerability. Patients will be randomly allocated in an experimental or control placebo arm.

The study data and interim results will be monitored by an independent Data Safety and Monitoring Board (DSMB), following the procedures described in ANNEX 7. The main task for the DSMB will be to assess whether the randomized comparisons provide evidence on the primary outcomes, sample size re-calculation, safety or futility with evidence strong enough to affect current treatment strategies. In such a circumstance, the DSMB will inform the coordinating investigator and sponsor , who will discuss whether to make the results available to the public. Regardless, follow-up will continue for all randomized individuals.

The hypotheses of the study are the following:

1. Non-hospitalised mild or moderate COVID-19 patients receiving convalescent MBT plasma infusion wll have a greater reduction in the severity of disease than those receiving placebo, reflected in a reduction of hospitalization rates.
2. Non-hospitalised mild or moderate COVID-19 patients receiving convalescent MBT plasma infusion will have a lower virologic load on day 7 than those receiving placebo.
3. Non-hospitalised mild or moderate COVID-19 patients receiving convalescent MBT plasma infusion will have a greater reduction in the severity of COVID-19 symptoms than those receiving placebo.
4. Non-hospitalised mild or moderate COVID-19 patients receiving convalescent MBT plasma infusion will have a shorter time to resolution of COVID-19 symptoms than those receiving placebo.
5. Non-hospitalised mild or moderate COVID-19 patients receiving convalescent MBT plasma infusion will have a greater reduction in the severity of disease than those receiving placebo, reflected in a reduction of mortality rates.
6. Non-hospitalised mild or moderate COVID-19 patients receiving convalescent MBT plasma will have a similar number and grade of AEs over a 28-day period, compared to those receiving placebo.
7. Non-hospitalised mild or moderate COVID-19 patients receiving convalescent MBT plasma will have blood lower levels of ferritin, IL-6, D-dimer, CRP, and leucocyte counts, and higher levels of prealbumin and lymphocyte count on day 7, compared to those receiving placebo.
8. Non-hospitalised mild or moderate COVID-19 patients receiving convalescent MBT plasma and placebo will confirm similarity of pre-treatment and on-treatment titers of neutralizing antibodies to SARS-CoV-2 among them on day 7.
9. Non-hospitalised mild or moderate COVID-19 patients receiving convalescent MBT plasma will reach higher titers of neutralizing antibodies to SARS-CoV-2 on day 60 than those receiving placebo, showing a better humoral response.
10. Self-collected MT swab and self-collected saliva will be suitable alternative samples to nasopharyngeal swabs collected by a healthcare worker to evaluate SARS-CoV-2 viral load

# 3 STUDY OBJECTIVES

## 3.1 Primary Objectives

1. Assess the therapeutic potential of early administration of convalescent MBT plasma in reducing the rate of hospitalization at day 28 in non-hospitalised mild or moderate COVID-19 patients.
2. Assess the therapeutic potential of early administration of convalescent MBT plasma in reducing SARS-CoV-2 viral load at day 7, measured by quantitative PCR (RT-qPCR) in non-hospitalised mild or moderate COVID-19 patients.

## 3.2 Secondary Objectives

1. Assess the therapeutic potential of early administration of convalescent MBT plasma in reducing WHO Clinical progression scale score in non-hospitalised mild or moderate COVID-19 patients.
2. Assess the therapeutic potential of early administration of convalescent MBT plasma in reducing the severity of common COVID-19 symptoms, measured with the FLU-PRO© PLUS scale, in non-hospitalised mild or moderate COVID-19 patients.
3. Assess the therapeutic potential of early administration of convalescent MBT plasma in reducing the duration of symptoms in non-hospitalised mild or moderate COVID-19 patients.
4. Assess the therapeutic potential of early administration of convalescent MBT plasma in reducing the mortality at day 60 in non-hospitalised mild or moderate COVID-19 patients.
5. Evaluate the safety and tolerability of convalescent MBT plasma in non-hospitalised mild or moderate COVID-19 patients.
6. Assess the change from baseline to day 7 of ferritin, prealbumin, interleukin-6 (IL-6), D-dimer, C reactive protein (CRP), and leukocytes and lymphocytes count in peripheral blood in non-hospitalised mild or moderate COVID-19 patients receiving convalescent MBT plasma.
7. Assess the impact of infused plasma on neutralizing activity by quantifying the change from baseline to day 7 in neutralizing antibodies against SARS-CoV-2 in peripheral blood in non-hospitalised mild or moderate COVID-19 patients receiving convalescent MBT plasma.
8. Assess the long-term impact of plasma infusion on humoral immune responses, by quantifying the change from baseline to day 60 in neutralizing antibodies against SARS-CoV-2 in peripheral blood in non-hospitalised mild or moderate COVID-19 patients receiving convalescent MBT plasma.
9. Compare agreement and SARS-CoV-2 viral load in self-collected middle turbinate (MT) swab and self-collected saliva samples with nasopharyngeal swab samples collected by a healthcare worker.
10. Assess the therapeutic potential of early administration of convalescent MBT plasma in reducing SARS-CoV-2 viral load at day 28, measured by quantitative PCR (RT-qPCR) in non-hospitalised mild or moderate COVID-19 patients.

# 4 TRIAL DESIGN

## 4.1 Type of Trial

This is a multi-site, randomized, controlled with placebo, double blind, parallel study.

## 4.2 Description of the Design

This is a prospective, randomized (1:1), double blind, study of convalescent anti-SARS-CoV-2 MBT plasma plus standard medical treatment (SMT) versus placebo plus SMT in mild or moderate COVID-19 patients who are non-hospitalised. Subjects with a confirmed SARS-CoV-2 infection as determined by positive polymerase chain reaction (PCR) or validated antigen rapid diagnostic test from nasopharyngeal swabs will receive SMT plus one single infusion of 200-300 ml of convalescent plasma that has been pathogen-inactivated using methylene blue treatment (MBT) or 200-300 ml of placebo.

The study will be announced on social media and the press (see ANNEX 6 for dissemination). Primary care centres (CAPs) will inform SARS-CoV-2 positive candidates of the possibility to volunteer in the study. At the CAPs, this information will be given during the medical visit in which patients are informed of their PCR positive result.

Study candidates will voluntarily register at the study w www.estudicovid19.org/ - CoV-2-positive individuals to volunteer in COVID-19 clinical trials (see ANNEX 6 for web content), and will provide an authorization to access their shared medical record.

In order to minimize the transfers of COVID-19 patients, given the risk of contagion that this would entail, eligibility of candidates will be assessed by study physicians in a remote screening visit, based on the revision of regional shared medical records (Història Clínica Compartida de Catalunya; HC3) during an interview with the candidate. Non-eligible candidates will be informed of the reason for non-eligibility. Eligible candidates will be scheduled an inclusion/baseline visit.

In the inclusion/baseline visit, candidates will be informed in person about the study, formally invited to participate, and the informed consent will be obtained. Once each candidate has signed the informed consent, he/she will undergo the required clinical procedures for final eligibility assessment (i.e., physical examination and a urine pregnancy test). Non-eligible candidates will be informed about the reason for their non-inclusion.

Candidates will also be identified at emergency room (ER) and/or outpatient departments (OPDs) of the participating sites. In this case, the remote screening visit will not be necessary; an inclusion/baseline visit will be the performed, where the candidate will be informed in person about the study, invited to participate and the informed consent will be obtained before the assessment of eligibility.

Eligible candidates will be randomized. To avoid unnecessary tests, ABO compatibility test will be performed only in those that will receive convalescent plasma. The results of the tests will be known only by the unblinded study nurse and the information will be kept blinded for the rest of the study team.

Randomized participants will be administered a single intravenous (IV) infusion at baseline (convalescent plasma or placebo). The administration will be performed by an unblinded study nurse, but both the investigator and the participant will be blinded to the investigational product

Specifically, subjects randomized to combination convalescent anti-SARS-CoV-2 MBT plasma plus SMT will receive one infusion of 200 to 300 ml of ABO-compatible convalescent plasma. Subjects

randomized to combination placebo plus SMT will receive one infusion of 200 to 300 ml of sterile saline solution 0.9%. Infusion will be administered at baseline, using standard operating procedures (SOPs) that will be in place before the study start. Small adults weighing less than 45 kg will receive one infusion of 5 ml of convalescent plasma or placebo per kilogram of body weight (to a maximum of 250ml of convalescent plasma or placebo).

Samples of the infused convalescent anti-SARS-CoV-2 MBT plasma will be obtained before infusion for quantitative measurement of neutralizing antibodies against SARS-CoV-2.

Nasopharyngeal and blood samples will be obtained from all participants at inclusion/baseline visit, on day 7 and on day 28. Nasopharyngeal samples will be used to assess SARS-CoV-2 viral load quantification. Blood samples will be used to assess viral load in nasopharyngeal swabs and inflammatory prognostic markers (ferritin, prealbumin, interleukin 6, d-Dimer, C reactive protein leukocyte and lymphocyte counts). Leftovers of plasma will be stored for future investigations.

Participants will be trained on the completion of symptoms and safety diary card.

The symptoms and safety diary card will be filled by the participants daily from baseline up to day 14. Self-reported data will be collected using an electronic case report form (eCRF). Paper forms will be available for those participants who are unable to submit data using eCRF, in which scenario data will be collected at follow-up visits on day 7 and by a study courier on day 15. If the participant reports any new symptom with grade 4 at the FLU-PRO® PLUS questionnaire, he or she will be contacted by telephone by the study staff to evaluate the need of an extra home visit. Participants will be also given a contact number to report AEs, to seek medical advice, and resolve any question related to the study. The investigator team will review the symptoms and safety diary cards on days 3, 7, and 14, to assess compliance with self-reporting data. For participants who do not respond to follow-up surveys, investigators will use emails or telephone calls to ascertain outcomes from them.

On follow-up visits on days 3, 7, 14, and 28, all participants will be assessed for clinical and safety outcomes. These visits will be by telephone except for the day 7 and the day 28 visits. Day 7 will be a visit at home or at the hospital, where additionally nasopharyngeal swabs and blood samples will be collected. Day 28 will be a hospital visit, where additionally nasopharyngeal swabs sample will be collected

At day 60, all participants will be assessed by telephone for health status outcome.

If the participant is unreachable at phone follow-up visits, clinical progression, as well as possible AEs will be assessed through medical records (Història Clínica Compartida, HC3) in order to assess all the study outcomes.

Results of the sample analyses are not necessary for participants to remain in the study to continue in the study, and participants will only be informed of relevant results for their health.

Participation in the substudy will be offered at the inclusion/baseline visit to a maximum of 135 participants in selected sites and those who accepts will sign a substudy specific informed consent. At baseline, the participants of the substudy will be drawn an extra tube of blood sample and will be trained on self-collection of middle turbinate (MT) swabs and saliva, and self-collected samples will be obtained. On day 7, an extra tube of blood sample will be obtained, and they will be asked to self-collect MT swabs and saliva. And on day 60, an extra tube of blood sample will be obtained during an additional home or hospital visit. The extra tube in both days 7 and 60 will be stored for neutralizing antibodies assessment. MT swabs and saliva samples will be compared with nasopharyngeal swabs for SARS-CoV-2 viral load quantification. Leftovers of plasma will be stored for future investigations.

## 4.3 Study Endpoints

### *4.3.1 Primary endpoint(s)*

Hospitalization rate (i.e., who r
Frame: Up to 28 days after reception of investigational product]
Reduction of SARS-CoV-2 viral load in nasopharyngeal swabs at day 7 after start of treatment, as determined by RT-qPCR. [Time Frame: Up to 7 days after reception of investigational product]

### *4.3.2 Secondary endpoints*

1. Change in COVID-19 WHO Clinical progression scale score [Time Frame: Up to 60 days after reception of investigational product]
2. Change in COVID-19 symptoms severity score, assessed with the COVID-19 daily self-score tool (FLU-PRO© PLUS instrument) [Time Frame: Up to 14 days after reception of investigational product]
3. Time to complete resolution of symptoms [Time Frame: Up to 28 days after reception of investigational product]
4. Death rate [Time Frame: Up to 60 days after reception of investigational product]
5. FDA Toxicity Grading Scale for Healthy Adult and Adolescent Volunteers [Time Frame: Up to 28 days after reception of investigational product]
6. Change in inflammatory prognostic markers (ferritin, prealbumin, interleukin-6 (IL-6), D-dimer, C reactive protein (CRP) and leukocyte and lymphocyte counts) [Time Frame: Baseline and day 7 after reception of investigational product]
7. Intergroup comparison of absolute neutralization titers against SARS-CoV-2 in plasma of a subgroup of participants [Time Frame: Baseline and day 7 after reception of investigational product]
8. Change in titers of neutralizing antibodies against SARS-CoV-2 in plasma of a subgroup of participants [Time Frame: Baseline and day 60 after reception of investigational product]
9. Agreement and SARS-CoV-2 viral loads of self-collected middle turbinate (MT) swab and self-collected saliva compared to nasopharyngeal swabs collected by a health care worker [Time Frame: Baseline and day 7 after reception of investigational product].
10. Reduction of SARS-CoV-2 viral load in nasopharyngeal swabs at day 28 after start of treatment, as determined by RT-qPCR. [Time Frame: Up to 28 days after reception of investigational product].

## 4.4 Measures to Avoid Bias

The following measures will be used to avoid bias: Randomization and blinding.

### *4.4.1 Randomization*

Participants will be randomly allocated using the study eCRF in a ratio of 1:1 between the convalescent plasma arm (experimental) and the placebo arm (control).

Randomization will be centralized at the blood bank during the inclusion/baseline visit, after confirmation of eligibility. ABO compatibility test will be performed for the experimental arm.

### 4.4.2 Stratification

There will not be stratification.

### 4.4.3 Blinding

This will be a double blind, placebo-controlled study. Masking of investigational products will ensure that both the investigator and the participant are blinded to the product administered.

Opaque bags will be used for masking the investigational products. Convalescent plasma infusion is to be performed using a filter, that opaque infusion system does not include. The investigational product and the system will be covered with opaque bags and opaque tubular bags. Nevertheless, convalescent plasma and placebo have very different appearance and the masking might not be completely efficient for the study nurse manipulating the IV cannula.

To ensure blinding of the participant and the investigators assessing the study outcomes, the following operational measures will be used:

- Randomization and preparation of the investigational product will be performed in the blood bank by unblinded staff , who will cover the investigational product with opaque bags.
- Infusion will be performed by an unblinded study nurse, using an opaque tubular bag to cover the infusion system. Neither the study physician nor the participant will be informed about the investigational product received.
- Study outcomes will be assessed by blinded study physicians. Should the physician assess clinical progression and/or AEs through medical record, specific measures will be used to avoid being informed of the product infused.

### 4.4.4 Unblinding

Unblinding of an individual participant will be indicated in the event of a medical emergency where the clinical management of the participant would be altered by knowledge of the investigational product received. In any situation, every attempt will be made to minimize the number of participants unblinded. If the blind is broken for any reason, this will be recorded and the sponsor will be notified immediately. Instructions for unblinding will be provided to the study investigators. Participants will rmation regarding the study and the contact details of the investigators team (ANNEX 9).

Global unblinding for the study team will be performed after the statistical analyses are finalized, and participants will be informed of the treatment received.

### 4.4.5 Identification of Subjects

The participant ID will consist of a sequential number that will be assigned to each participant at the inclusion/baseline visit. This number will be used as a unique identifier for each participant throughout the study.

## 4.5 End of Study

The date of the end of the study will be the last visit of the last participant.

The study will be completed when any of these premises are met:

- Inclusion of the number of participants needed for the sample size and end of clinical study visits.
- The DSMB recommends completing the study prematurely for safety reasons.
- Sponsor or principal investigator decision.

If the study must be interrupted prematurely, all non-used materials should be returned to the sponsor The principal investigator
eCRF.

In case there were no participants included in the study, the sponsor will take care of all materials.

## 4.6 Source Data

Source documents will be the participants' electronic health records including laboratory results obtained from blood and swab tests.

The symptom and safety diary card, composed of different adapted questionnaires described in section 7, will be considered source document and will be collected directly on the eCRF (filled by the participant and reviewed by the investigator). Paper questionnaires may also be used by participants who are unable to use the eCRF.

Study data will be collected through an eCRF.

All investigational results obtained from study sample analyses will be recorded in separated electronic databases, which will be merged with the clinical study data for statistical analysis.

# 5 STUDY INVESTIGATIONAL PRODUCTS

## 5.1 Experimental and Control Investigational Products

The experimental investigational product will be ABO-compatible SARS-CoV-2 convalescent anti-SARS-CoV-2 MBT plasma and the control investigational product will be sterile saline solution 0.9%.

Investigational products will be administered in addition to the standard medical treatment (SMT) for COVID-19 disease prescribed by their regular physicians.

## 5.2 Arm Description

After confirmation of eligibility at the inclusion/baseline visit, participants will be randomly allocated to one of the following arms:

| Arm | Investigational Product / Treatment |
| --- | --- |
| EXPERIMENTAL ARM | The participant will receive one infusion of 200 to 300 ml of ABO-compatible convalescent MBT plasma with neutralizing SARS-CoV-2 antibodies plus SMT. |
| CONTROL ARM | The participant will receive one infusion of 200 to 300 ml of sterile saline solution 0.9% via IV plus SMT. |

## 5.3 Supply, Packaging, Labelling and Storage

### *5.3.1 Supply*

Commercial saline solution 0.9% will be supplied by the sponsor.

Details regarding donor selection, collection and processing procedures for convalescent MBT plasma preparation from donors recovered from COVID-19 can be found in ANNEX 1.

### *5.3.2 Packaging and Labelling*

Packaging and labelling of convalescent plasma and placebo will comply with local regulatory requirements.

### *5.3.3 Storage*

Investigational products will be stored at the blood bank facilities of each study site.

Convalescent anti-SARS-CoV-2 MBT plasma will be stored to the conditions of temperature, humidity
ANNEX 1).

Commercial saline solution 0.9% will be stored at room temperature.

### *5.3.4 Preparation and Blinding*

Standard operating procedures (SOPs) will be in place for the preparation and blinding of the investigational products.

Both convalescent anti-SARS-CoV-2 MBT plasma and saline solution 0.9% will be prepared for infusion by unblinded personnel. Convalescent anti-SARS-CoV-2 MBT plasma will be thawed before being

covered by an opaque bag, while saline solution 0.9% will be directly covered by an opaque bag. To avoid a blinding breach, the time of preparation and blinding will be approximately the same for both investigational products.

Convalescent anti-SARS-CoV-
standard procedures.

Opaque bags will be labelled in the same manner, including at least the participant ID and the permitted time frame of infusion. Opaque tubular bags will be used to cover the infusion system. Infusion will be performed by study personnel, adhering to the time frames and storage conditions
ANNEX 1).

## 5.4 Dose, Interval, Route and Method of Administration

### *5.4.1 Dose, Interval, and Route*

Participants will receive one infusion of 200 to 300 ml ABO-compatible convalescent anti-SARS-CoV2 MBT plasma or saline solution 0.9% at baseline.

Small adults weighing less than 45 kg will receive one infusion of 5 ml of ABO-compatible convalescent plasma or saline solution per kilogram of body weight.

### *5.4.2 Administration and Timing of Investigational Plasma for Each Subject*

Investigational products will be infused using standard procedures for administration of fresh frozen plasma, which will be detailed in the study SOPs.

Start and end of infusion time will be recorded. Vital signs will be measured immediately before the start of infusion, and immediately after the completion of infusion. Participants will remain under observation for 20-30 minutes after the completion of the infusion to monitor AEs.

## 5.5 Modification of the Treatment Regimen

No changes in treatment regimens are expected. If an AE develops during infusion, the infusion will
uct will be
halted if any manifestations of anaphylaxis develops, and it will not be restarted.

## 5.6 Prior and Concomitant Treatments

SMT and treatments administered at baseline, and during the study up to day 28 will be considered concomitant treatments, will be recorded in the medical clinical record and in the eCRF, including the trade and/or generic names of the medication, the dose (if known), the route of administration, and the duration of the medication (frequency).

Medicines for transfusion reactions (e.g. paracetamol, dexchlorpheniramine) may be given as a treatment, if necessary.

There are no prohibited medications prior to study participation or during the study.

There are no restricted concomitant medications.

## 5.7 Accountability Procedures for Investigational Product

The investigator, or designee such as the blood bank personnel or study nurse, will keep the investigational products accountability.

Investigational products will be used only for the study in accordance with the directions given in this protocol.

The investigator, or designee such as the blood bank personnel or study nurse, is responsible for handling of the investigational products in accordance with the directions given in the protocol and in the study SOPs. The investigator is responsible for maintaining accurate records of the investigational product for the site. Documentation verifying receipt, dispensing, destruction, or return of the investigational products will be maintained by the investigator or designee and will be made available to the study monitor/relevant authorities for review. Inventory dispensing logs must be verified by the monitor. At the end of the study, a copy of the inventory/dispensing log(s) will be retrieved by the monitor, and the original document will be stored in the trial master file.

## 5.8 Treatment Compliance

A study nurse will perform the investigational product administration, and he/she will register the incidences occurring during the procedure.

# 6 SELECTION AND WITHDRAWAL OF SUBJECTS

## 6.1 Study population

Individuals with confirmed infection by SARS-CoV-2 from different regions of Catalonia.

## 6.2 Inclusion Criteria

1. Adult male or female individuals
2. In women of childbearing potential[1], negative pregnancy test at inclusion/baseline visit.
3. Has confirmed SARS-CoV-2 infection as determined by PCR or validated antigen rapid diagnostic test[2] .
4. Symptomatic with mild or moderate COVID inclusion/baseline visit.
    a. Mild COVID-19: Individuals who have any of the common signs and/or symptoms of COVID-19 (i.e., fever, cough, sore throat, malaise, headache, muscle pain) without shortness of breath, dyspnoea, or abnormal chest imaging.
    b. Moderate COVID-19: Individuals who have evidence of lower respiratory disease by clinical assessment or imaging and a saturation of oxygen ($SpO_2$ sea level.
5. Willing to comply with the requirements of the protocol and available for follow-up for the planned duration of the study.
6. Has understood the information provided and capable of giving informed consent.

[1]A woman will be considered of childbearing potential if not permanently sterilized nor postmenopausal. Permanent sterilization methods include tubal ligation, hysterectomy and bilateral oophorectomy. Postmenopausal is defined as 12 months with no menses without an alternative medical cause.

[2]Panbio™ COVID-19 Ag Rapid Test (Abbott), STANDARD™ Q COVID-19 Ag Test (Roche) or any other CE marketed test for SARS-CoV-2 Ag detection.

## 6.3 Exclusion Criteria

1. If female, pregnant or breastfeeding, or planning a pregnancy during the study.
2. Severe or critical COVID-19:
    a. Severe COVID-19: respiratory frequency >30 breaths per minute, $SpO_2$ <94% on room air at sea level, ratio of arterial partial pressure of oxygen to fraction of inspired oxygen ($PaO_2/FiO_2$) <300 mmHg, or lung infiltrates >50%.
    b. Critical COVID-19: respiratory failure, septic shock, and/or multiple organ dysfunction.
3. Current hospital admission for any cause.
4. History of previous confirmed SARS-CoV-2 infection.
5. History of significantly abnormal liver function (Child Pugh C).
6. History of chronic kidney disease (CKD) stage 4, or need of dialysis treatment.
7. Any pre-existing condition that increases risk of thrombosis.
8. History of allergic reactions to blood or plasma products or methylene blue.
9. Known IgA deficiency with anti-IgA antibodies.
10. Medical conditions for which 02 0-300 ml of intravenous fluid is considered dangerous (i.e., decompensated heart failure or renal failure with fluid overload).
11. Inability to consent and/or comply with study protocol, in the opinion of the investigator.

12. Currently participating or planning to participate in any interventional study for the treatment of COVID-19 or SARS-CoV-2 infection until day 60.

## 6.4 Subject Withdrawal Criteria

### *6.4.1 Early Subject Withdrawal*

Participants will prematurely end their participation in the study in the following circumstances:

At their own request.

-being.

At the specific request of the sponsor.

The participant is not able to adhere to the main protocol requirements, in the opinion of the investigator.

In the exceptional case of non-availability of ABO-compatible convalescent plasma.

Other (the reason will be specified).

As a general rule, a participant will remain in the study as long as the primary outcome measures are available.

### *6.4.2 Medical Approach to Withdrawal*

Withdrawals will be notified to the sponsor and the date and reason for the withdrawal will be collected. The investigator will provide adequate medical support to the participant if required if required.

### *6.4.3 Follow-up after Early Withdrawal*

Randomized participants who withdraw will be followed up to day 28 for clinical and safety outcomes and up to day 60 for health status outcome, except in case the reason of withdrawing is voluntary.

### *6.4.4 Replacement of Participants*

Replacement of participants will be allowed only in case of early withdrawal happening before the start of the infusion of investigational product.

Participants withdrawn after the start of the infusion will not be replaced.

### *6.4.5 Pre-randomization Loses*

All candidates considered for the study will be registered on the screening list, including the reasons for non-suitability or non-attendance to the inclusion/baseline visit.

Candidates who sign the informed consent will undergo an inclusion/baseline visit; those who are found not eligible will not be randomized, but data from the visit and the reason for non-inclusion will be recorded in the eCRF.

Subject re-screening will not be performed.

# 7 STUDY CONDUCT AND ASSESSMENT OF RESPONSE

## 7.1 Study Visits and Procedures

### *7.1.1 Recruitment procedures*

Candidates will be recruited from:

www.estudicovid19.org/
will be asked essential questions to preliminary assess their suitability (see ANNEX 6 for web content). The registration on the website will entail an authorization to access the candidate shared medical records to permit a preliminary assessment of eligibility over the phone.
Invitation through ER or OPD staff of the participating sites, where candidates be invited to participate by study physicians on-site.

Invitation by telephone call in a follow-up visit to patients with confirmed SARS-CoV-2 infection who appear in a list of positive PCR or rapid antigen test from the laboratory of the participating sites.

### *7.1.2 Screening*

With the aim of minimizing the transfers of COVID-19 patients, given the risk of contagion that this would entail, candidates who voluntarily register on the study website will be assessed for eligibility for the study by study physicians based on the revision of regional shared medical records (Història Clínica Compartida de Catalunya, HC3) during a phone interview. Non-eligible candidates will be informed of the reason for non-eligibility. Eligible candidates will be scheduled an inclusion/baseline visit.

Candidates identified through ER or OPD of the participating sites will be informed about the study and if interested, will be directed to the inclusion/baseline visit to obtain informed consent and assess eligibility on-site.

A record containing all evaluated candidates and reasons for non-inclusion will be in place for the study (i.e., screening log).

### *7.1.3 Inclusion/baseline*

The inclusion/baseline visit will be a hospital setting visit with a study physician and a study nurse, either at the hospital or, if safe requirements detailed on the study SOPs are met, at home by Hospital at home care unit staff (Hospitalització domiciliària).

The procedures will be the following:

1. In-person information of study details
2. Informed consent form signature
3. Urine pregnancy test in females of child-bearing potential
4. Interview of the participant:
    a. Record demographic data.
    b. Record of SARS-CoV-2 PCR or antigen RDT result (including date of analysis).
    c. Identification baseline medical conditions and concomitant treatment.
    d. Identification of date of onset of COVID-19 symptoms.
5. Physical examination (including measuring weight and height) and vital signs (Body temperature, Oxygen saturation, Respiratory rate, Heart rate, Blood pressure).

6. Verification of inclusion and exclusion criteria. In case of non-inclusion, the participant will be informed of the reason and the study staff will provide the necessary medical attention.

If the participant is found eligible, the study staff will perform the following procedures:

1. In the selected sites, offer participation in the substudy and sign the specific informed consent.
2. Classification of baseline severity of COVID-19 using WHO scale.
3. Blood draw:
   a. Blood sample collection for an ABO compatibility test (which will be performed in case the participant is assigned to convalescent plasma group).
   b. Blood sample collection for inflammatory prognostic markers (ferritin, prealbumin, IL-6, D-dimer, CRP, and leukocyte and lymphocyte counts)
   c. Blood sample collection for storage at IrsiCaixa.
   d. In substudy participants, blood sample collection for quantification of neutralizing antibodies against SARS-CoV-2.
4. Nasopharyngeal swabs collection by study nurse.
5. In the substudy participants, training of the participant on the process of self-collection of MT swabs and saliva samples, and self-collection of MT swabs and saliva samples by the participant.
6. Training in the completion of the symptoms and safety diary card, which will be filled by the participant at baseline and daily until day 14, using the study an eCRF. Paper forms will be available for those participants who are unable to submit electronic data using eCRF, and in this scenario data will be collected in the eCRF at follow-up visits on day 7 and by a study courier on day 15.
   a. If the participant reports any new symptom with grade 4 at the FLU-PRO© PLUS questionnaire, he or she will be contacted by telephone by the study staff to evaluate the need of an extra home visit
   b. Participants will be also given a contact number to report AEs, to seek medical advice, and resolve any question related to the study.
7. Randomization to assign the participant to a study arm, centralized at the Blood Bank facilities by study staff.
8. In case of convalescent plasma is assigned, the blood bank staff will perform the ABO compatibility test using standard guidelines for blood transfusion laboratories.
9. Preparation and blinding of ABO-compatible convalescent plasma or placebo to be administered.
10. Transport of the investigational product from the Blood Bank to the place of infusion.
11. Administration of a single infusion of 200 to 300 ml, of investigational product (either convalescent plasma or placebo).
    a. Time at start and end of infusion will be recorded.
    b. Infusion will be done in accordance with the standard policies for administration of blood and routine administration of fresh frozen plasma, and study specific SOPs will be in place.
    c. If an AE develops during infusion, the infusion will be slowed or stopped as per
    ation of anaphylaxis
    develops, and will not be restarted.

   d. Vital signs (Temperature, Oxygen saturation, Respiratory rate, Heart rate, Blood pressure) will be measured at immediately after the completion of infusion.
   e. Medicines for infusion reactions (e.g. paracetamol, dexchlorpheniramine) may be given as a treatment, if necessary.
12. Monitoring of AEs for a period of 20-30 minutes after the completion of the infusion.
13. ANNEX 9), and instruct the participant to always carry the card with him/her.

#### *7.1.4 Day 3*

The participant will be contacted by telephone by study staff for safety and clinical follow-up. Specifically,

1. Revision of symptom and safety daily data, and discussion of inconsistencies with the participant.
2. Classification of severity of COVID-19 using WHO scale.
3. AEs will be assessed by the study staff.
4. If deemed necessary, AEs will be informed to and reviewed by the study physician, and medical attention will be given when necessary.

#### *7.1.5 Day 7*

The participant will be visited at home or at the hospital by study staff, and the following procedures will be performed:

1. Vital signs (Temperature, Oxygen saturation, Respiratory rate, Heart rate, Blood pressure).
2. Blood draw:
   a. Blood sample collection for inflammatory prognostic markers (ferritin, prealbumin, IL-6, D-dimer, CRP, and leukocyte and lymphocyte count). This sample will be sent to the laboratory for a routine processing.
   b. In the <u>substudy</u> participants, blood sample collection for quantification of neutralizing antibodies against SARS-CoV-2.
3. Nasopharyngeal swab sample collection.
4. In the <u>substudy</u> participants, self-collection of MT swabs and saliva samples by the participant.
5. Revision of symptom and safety daily data, discussion of inconsistencies with the participant.
6. Classification of severity of COVID-19 using WHO scale.
7. AEs will be assessed by the study staff.
8. If deemed necessary, AEs will be informed to and reviewed by the study physician, and medical attention will be given when necessary.

#### *7.1.6 Day 14*

The participant will be contacted by telephone by study staff for safety and clinical follow-up. Specifically,

1. Revision of symptom and safety daily data, and discussion of inconsistencies with the participant
2. Classification of severity of COVID-19 using WHO scale.
3. AEs will be assessed by the study staff.
4. If deemed necessary, AEs will be informed to and reviewed by the study physician, and medical attention will be given when necessary.

### 7.1.7 Day 28

The participant will be scheduled a visit with a study nurse at the hospital, where the following procedures will be performed:

1. Vital signs (Temperature, Oxygen saturation, Respiratory rate, Heart rate, Blood pressure).
2. Nasopharyngeal swab sample collection.
3. Classification of severity of COVID-19 using WHO scale.
4. Assessment of COVID-19 symptoms that were still persisting at day 14 (as collected in the symptom diary
5. AEs will be assessed by the study staff.
6. AEs will be informed to and reviewed by the study physician, and medical attention will be given when necessary.

### 7.1.8 Day 60 (End-of-Study)

The participant will be contacted by telephone by study staff for safety and clinical follow-up. Specifically,

1. Classification of severity of COVID-19 using WHO scale.
2. In the <u>substudy</u> participants, a visit will be scheduled within 5 days to obtain a blood sample.

### 7.1.9 Extra home visit (unscheduled visit)

The need of an extra home visit will be assessed by phone in case of:

Report of any new symptom with grade 4 at the FLU-PRO© PLUS questionnaire
Report of any AE of grade 3 or more

If deemed necessary by the study staff, at any time during the study period, the participant will be visited at home to perform the necessary procedures to assess an AE or provide medical attention.

### 7.1.10 Participant's Diary

Additionally to the scheduled study visits, the participant will perform the following procedures daily from day 1 to day 14:

1. Measurement of axillary body temperature
2. Fill the symptom and safety diary card, which will include the FLU-PRO© PLUS Questionnaire and additional questions regarding safety, and medications taken by the participant.

Data will be collected using an electronic case report form (eCRF). Paper forms will be available for those participants who are unable to submit data using eCRF, and in this scenario data will be collected at follow-up visit on day 7 and by a study courier on day 15.

## 7.2 Clinical Progression Scales

The following scales will be used to assess clinical progression of the participants:

### 7.2.1 WHO Clinical Progression Scale

This scale will be assessed by the study staff at baseline, and days 3, 7, 14, 28, and 60.

The WHO Clinical Progression Scale is recommended for core clinical outcomes (WHO Working Group on the Clinical Characterisation and Management of COVID-19 infection, 2020) (15), and will

This is an ordinal scale to measure clinical progression and recovery based on location and supportive measures used within the health-care system. It provides a measure of illness severity across a range from 0 (not infected) to 10 (death) with data elements that are rapidly obtainable from clinical records.

The WHO Clinical Progression Scale is located in ANNEX 2

### *7.2.2 FLU-PRO® PLUS Questionnaire*

Participants will fill daily questionnaires from baseline to day 14. Data will be self-reported, collected using the study eCRF and reviewed by the investigator at baseline, and days 3, 7, and 14.

Symptom severity will be also assessed with the certified Spanish translation of the FLU-PRO® PLUS Questionnaire. It is a COVID-19 daily self-score tool, to assess severity of symptoms across six body systems: nose, throat, eyes, chest/respiratory, gastrointestinal, and body/systemic

The instrument also provides data on the presence/absence of symptoms, symptom profiles, and change over time.

Adapted from the FLU-PRO® instrument, the COVID version consists of 34 items that are answered daily. Items 1-27 are Likert scale questions (rated 0-4) where 0 = not at all and 4 = very much. These items are summed to score the severity of symptoms where a total score of 112 would indicate the greatest severity of symptoms and a score of 0 would indicate no severity of symptoms. Items 28-32 are also Likert scale questions (rated 0-4) that measure the frequency of specific daily symptoms where 0 = never or 0 times and 4 = always or 4 times. These items are summed to score the frequency of symptoms, where the highest score for the frequency of symptoms (20) indicates the greatest burden of symptom frequency. Items 33 and 34 are yes/no questions that measure the presence/absence of COVID-19 specific symptoms, where 0 = no and 1 = yes. These items are summed, where the highest score of 2 indicates presence of both symptoms. The total maximum score of the FLU-PRO® PLUS questionnaire is 134.

The FLU-PRO®PLUS Questionnaire is located in ANNEX 3 For the present study.

### *7.2.3 Assessment of Safety*

Safety will be assessed using the safety diary card filled daily by participants and through telephone contact by study staff on day 3, 7 and 14. Safety diary card and phone assessments are detailed in ANNEX 4

Participants will fill daily from baseline to day 14 self-reported data related to symptoms and safety. These data will include medication-related effects with directed questioning on the most common adverse effects and an open-ended free-text field.

The investigator team will review surveys on days 3, 7, and 14, to assess compliance in self-reporting data and safety data will be also collected, with direct questioning on common AEs and indirect questioning on other possible AEs.

If the participant reports any new symptom with grade 4 at the FLU-PRO® PLUS questionnaire or any AE of grade 3 or more, he or she will be contacted by telephone by the study staff to evaluate the need of an extra home visit. The need of an extra home visit will be evaluated if an important AE is detected on the phone visits.

Participants will be also given a contact number to report AEs, to seek medical advice, and resolve any question related to the study

For participants who would not respond to follow-up surveys, investigators will use e-mails or telephone calls to ascertain outcomes from them.

## 7.3 Description of Laboratory Tests and Procedures

| Test | Type of sample | Description | Location | Visit |
|---|---|---|---|---|
| Inflammatory prognostic markers | Blood sample | White cell blood count ferritin, prealbumin, interleukin 6 (IL-6), D-dimer, C reactive protein (CRP) | Central Laboratory of each study site | Baseline* and day 7 |
| ABO compatibility test | Blood sample | Type of anti-A and anti-B in red cells and in serum only i[illegible] participants assigned to convalescent plasma group | BST or Haematology Department of each study site | Baseline* |
| Quantitative measurement of SARS-CoV-2 viral load | Nasopharyngeal swab | Quantitative RT-PCR of SARS-CoV-2 | Clinical genetics Service of the HUGTiP | Baseline*, days 7 and 28 |
| Quantitative measurement of SARS-CoV-2 viral load | Self-collected MT swab and self-collected saliva | Quantitative RT-PCR of SARS-CoV-2 (substudy) | Clinical genetics Service of the HUGTiP | Baseline* and day 7 |
| Quantitative measurement of neutralizing antibodies against SARS-CoV-2 | Convalescent anti-SARS-CoV-2 MBT plasma infused | Quantitative measurement of neutralizing antibodies against SARS-CoV-2 | IrsiCaixa | Baseline |
| Quantitative measurement of neutralizing antibodies against SARS-CoV-2 | Blood sample | Quantitative measurement of neutralizing antibodies against SARS-CoV-2 (substudy) | IrsiCaixa | Baseline*, day 7 and 60 |
| Storage | Plasma | Future investigations. | IrsiCaixa and ISGlobal | Baseline* |
| | Leftovers of SARS-CoV-2 positive nasopharyngeal swab | Future investigations. | Clinical genetics Service of the HUGTiP | Baseline* and days 7, and 28 |
| | Leftovers of SARS-CoV-2 positive self-collected MT swab and self-collected saliva | Future investigations. | Clinical genetics Service of the HUGTiP | Baseline* and day 7. |

*All baseline samples will be obtained before start of infusion of investigational product.

## 7.4 Study Variables

Among others, the following study variables will be collected:

Demographic data:

1. Age
2. Sex (Male/Female)

Clinical data:

1. Comorbidities
    a. Chronic obstructive pulmonary disease
    b. Asthma
    c. Chronic hypertension
    d. Obesity
    e. Cardiovascular disease (Chronic congestive cardiac failure, ischemic heart disease)
    f. Chronic kidney disease
    g. Diabetes
    h. Cerebrovascular disease
    i. Cancer
    j. Immunosuppression
2. Concomitant treatments

COVID-19 clinical data:

4. Date of positive SARS-CoV-2 result
5. Type of test:
    a. RT-PCR
    b. Validated antigen rapid diagnostic test
6. Date of symptom onset
7. COVID-19 severity (mild, moderate, severe or critical)

Participant diary card, list of symptoms (extracted from the FLU-PRO® PLUS Questionnaire, ANNEX 3)

1. Fever
    a. Felt cold
    b. Felt hot
    c. Sweating
2. Cough
    a. Dry or hacking cough
    b. Wet or loose cough
    c. Frequency of coughing
    d. Frequency of coughing up mucus or phlegm
3. Sore throat
    a. Scratchy or itchy throat
    b. Sore or painful throat

        c. Difficulty swallowing
4. Malaise
    a. Lack of appetite
    b. Sleeping more than usual
    c. Weak or tired
    d. Chills or shivering
5. Headache
    a. Felt dizzy
    b. Head congestion
    c. Headache
6. Muscle pain
    a. Body aches or pains
7. Shortness of breath, dyspnoea
    a. Trouble breathing
    b. Chest congestion
    c. Chest tightness
8. Gastrointestinal symptoms
    a. Felt nauseous
    b. Stomach ache
    c. Frequency of vomiting
    d. Frequency of diarrhoea
9. Anosmia
10. Ageusia
11. Rhinitis
    a. Runny or dripping nose
    b. Congested or stuffy nose
    c. Sinus pressure
    d. Frequency of sneezing
12. Conjunctivitis
    a. Teary or watery eyes
    b. Sore orpainful eyes
    c. Eyes sensitive to light

Participant diary card, safety:

1. Injection site pain
2. Injection site swelling
3. Rash
4. Injection site pruritus

Physical examination and vital signs:

1. Saturation of oxygen on room air at sea level
2. Blood pressure
3. Temperature
4. Respiratory rate
5. Heart rate

6. Weight measurement
7. Height measurement

Convalescent anti-SARS-CoV-2 MBT plasma analysis:

1. Titers of anti-SARS-CoV-

Blood analysis:

1. Complete Blood Count (lymphocytes, leucocyte count)
2. Ferritin
3. Prealbumin
4. Interleukin 6 (IL-6)
5. D-dimer
6. C-reactive Protein (CRP)
7. Titers of anti-SARS-CoV-2 antibodies

Respiratory swabs (nasopharyngeal and MT) and saliva analysis:

1. SARS-CoV-2 RT-qPCR

Hospitalization

1. Date of hospitalization
2. Cause
3. Notification and collection of data as defined in section 8

Death

1. Date
2. Cause

Radiological findings (if available)

1. Chest CT scan
2. Chest x-ray

Adverse events

1. As defined in section 8

Concomitant treatments

1. As defined in section 5.6

## 7.5 Schedule of Procedures

| | Screening (phone)[6] | Inclusion/Baseline (hospital setting visit)[7] | Day 3 (phone) | Day 7 (home/ hospital ) | Day 14 (phone) | Day 28 (hospital) | Day 60 (phone) |
|---|---|---|---|---|---|---|---|
| Allowable window (days) | N/A | 0 | + 2 | +/-2 | +/-2 | +/- 2 | +/- 2 |
| Authorization to access medical records[1] | X | | | | | | |
| Informed consent signature | | X | | | | | |
| Pregnancy test | | X | | | | | |
| Clinical examination | | X | | | | | |
| Vital signs | | X | | X | | X | |
| Demographic, clinical data, and PCR record | | X | | | | | |
| Inclusion / exclusion criteria | X | X | | | | | |
| Nasopharyngeal swab | | X[2] | | X | | X | |
| MT swabs (self collection) | | X[2,3] | | X[3] | | | |
| Saliva sample (self collection) | | X[2,3] | | X[3] | | | |
| Blood draw | | X[2] | | X | | | X[3] |
| ABO compatibility test | | X[2,4] | | | | | |
| Inflammatory prognostic markers | | X[2] | | X | | | |
| Neutralizing antibodies | | X[2,3] | | X[3] | | | X[3] |
| Sample storage | | X[2] | | X | | | X[3] |
| Investigational product infusion | | X | | | | | |
| Symptom and safety diary card training | | X | | | | | |
| Adverse events | | X-----------------------------------------------------------------X--- | | | | | X[5] |
| Symptom and safety diary card | | X-----------------------------------------------X-- | | | | | |
| Symptom persistency or resolution | | | | | | X | |
| Follow up assessment | | X--------------------------------------------------------------------X--- | | | | | |
| Concomitant Treatment | | X--------------------------------------------------------------------X--- | | | | | |

MT: mid-turbinate; N/A: Not applicable

[1] In order to minimize the transfers of COVID-19 patients, given the risk of contagion that this would entail, eligibility will be assessed based on the revision of regional shared medical records (Història Clínica Compartida de Catalunya HC3) and clinical history taken by phone.

[2] Samples at baseline will be obtained prior to the infusion of the investigational product. [3] Substudy in a subgroup of 135 participants. Day 60 blood draw allowable window will be +5 days from the actual day of the day 60 visit.

[4] Blood for the ABO compatibility test will be collected from all study participants, but the test will only be performed in experimental (convalescent plasma) group.

[5] The time frame for new AE collection will end at day 28 visit. At day 60 visit, the investigator must collect follow up data of AEs that were not resolved on day 28, and SAEs due to hospitalization or death

[6] The screening remote visit will only be performed in participants registered on the study website.
[7] A visit at home by the Hospital at home care unit staff will be allowed if safe requirements detailed on the study SOPs are met.

# 8 ADVERSE EVENTS AND INCIDENTS

## 8.1 Safety parameters

The safety endpoints will include:

1. Cumulative incidence of adverse events (AEs) through day 28
2. Cumulative incidence of serious adverse events (SAEs) through day 28
3. Cumulative incidence of severe AEs through day 28

In this study,
the disease for which the investigational product is being studied. It may be an increase in the severity of the targeted disease and/or increases in the symptoms of the targeted disease. Anticipated symptoms of COVID-19 include fever, cough, hypoxia, dyspnoea, haemoptysis, myalgia, fatigue, pharyngitis, diarrhoea which may develop at any time during the course of the disease.

## 8.2 Definitions

Adverse event (AE): Medical event presented by a participant administered an investigational product, and which does not necessarily have a causal relation to the treatment.

Serious adverse event (SAE): Medical event classified as such and which, regardless of the dose involved:

1. Causes participant death.
2. Produces a life-threatening situation for the participant.
3. Requires or prolongs in hospital admission.
4. Produces important or persistent incapacitation/handicap or constitutes a congenital defect or anomaly.
5. Needs action to prevent any of above situations.
6. Is considered medically significant (examples of such events are intensive care in an Emergency Service or at home in a patient with allergic bronchospasm; blood dyscrasias or seizures not giving rise to hospital admission, or the development of drug dependency or abuse).

Unexpected adverse event (UAE): AE related to the investigational product the nature or intensity of which does not coincide with the information available on the product.

Suspected Unexpected Serious Adverse Reaction (SUSAR): SAE related to the investigational product the nature or intensity of which does not coincide with the information available on the product.

Incident related to the infusion:

Mistake in component infusion: event in which a participant is administered an investigational product that is non-compliant or was intended for another participant.
Incident without effect or almost-incident: event in which a mistake in component infusion is detected before happening.

## 8.3 Adverse Events Assessment

### *8.3.1 Seriousness*

A serious AE is any medical event that meets the criteria of SAE.

Events not considered to be SAEs are hospitalizations for:

1. A standard procedure for protocol therapy administration. However, hospitalization or prolonged hospitalization for a complication of therapy administration will be reported as an SAE.
2. Routine treatment or monitoring of the studied indication not associated with any deterioration in condition.
3. A procedure for protocol/disease-related investigations (e.g., surgery, scans, endoscopy, sampling for laboratory tests, bone marrow sampling). However, hospitalization or prolonged hospitalization for a complication of such procedures remains a reportable SAE.
4. Hospitalization or prolongation of hospitalization for technical, practical, or social reasons, in absence of an AE.
5. A procedure that is planned (i.e., planned prior to starting of treatment on study); must be documented in the source document and the CRF. Hospitalization or prolonged hospitalization for a complication remains a reportable SAE.
6. An elective treatment of a pre-existing condition unrelated to the studied indication.
7. Emergency out participant treatment or observation that does not result in admission, unless fulfilling other seriousness criteria above.

### *8.3.2 Intensity*

The FDA Toxicity Grading Scale for Healthy Adult and Adolescent Volunteers scale will be used to grade the intensity of the AE. Events not listed in the grading scale will be graded as follows:

| | |
|---|---|
| Grade 1 | Mild. No interference with activity. |
| Grade 2 | Moderate. Some interference with activity not requiring medical intervention. |
| Grade 3 | Severe. Prevents daily activity and requires medical intervention. |
| Grade 4 | Potentially Life Threatening. Emergency Room visit or hospitalization. |
| Grade 5 | Death. |

### *8.3.3 Causality*

All AEs must have their relationship to the investigational product assessed by the physician who examines/contacts and evaluates the participant based on temporal relationship and his/her clinical judgment. Causal relationship to the investigational product will be established according to medical judgment on whether there is a reasonable possibility of a causal relationship between the AE and the investigational product administration.

The investigator must determine and classify the AE causality according to the following categories:

Unrelated/Not related: There is not a reasonable possibility that the administration of the investigational product caused the AE, there is no temporal relationship between the investigational product administration and AE onset, or an alternate aetiology has been established.

Related: The AE is known to occur with the investigational product, there is a reasonable possibility that the investigational product caused the AE, or there is a temporal relationship between the investigational product administration and the AE. Reasonable possibility means that there is evidence to suggest a causal relationship between the investigational product administration and the AE.

- Possibly related: There is evidence to suggest a causal relationship between the investigational product and the AE.
- Definitely related: There is a reason to conclude that the investigational product caused the AE.

Criteria to assess the causal relationship should take in account of the following conditions.

1. A plausible temporal sequence from the investigational product administration to the AE onset.
2. Whether the AE follows a known response pattern to the suspected treatment.
3. comorbidities, or concomitant medications.
4. The occurrence of improvement on stopping/reducing the treatment (positive dechallenge) and/or reappearance of the AE on repeated exposure (positive rechallenge).

### 8.3.4 Expectedness

the nature, seriousness, severity, or outcome of the reaction(s) is not consistent with the reference information or expected effects of fresh frozen plasma infusion.

The assessment of the expectedness between an AE and the administration of treatment is a decision to be made by the principal investigator or co-investigator, who are qualified physicians.

A suspected unexpected serious adverse reaction (SUSAR) is a serious adverse event related to the investigational product whose nature, severity or outcome is not consistent with the reference information or expected effects of the fresh frozen plasma infusion.

All unexpected serious ARs will be notified to the study sponsor.

### 8.3.5 Duration

For both AEs and SAEs, the investigator will provide a record of start and stop dates of the event (expressed in the shortest time unit possible). Changes in the severity of an AE or SAE will be documented in the clinical record.

For AEs that occur during the infusion, the infusion rate in effect at the time of onset of the AE, the time of onset of the AE and the time of AE change materially in intensity and/or resolve will be captured.

### 8.3.6 Action Taken

The investigator will report the action taken with study intervention as a result of an AE or SAE, as applicable (i.e., discontinuation or reduction of volume of infusion, as appropriate) and report whether concomitant and/or additional treatments were given for the event.

### 8.3.7 Outcome

Any AE or SAE will be followed preferably until:

Resolution of the event.

Stabilization of the event

Resetting the baseline situation of the event, in case baseline situation is available.

Otherwise, they will continue until:

The event can be attributed to products other than the investigational product or factors unrelated to the study; or

It is unlkely to obtain further information.

## 8.4 Time Frame for Recording Adverse Events and Incidents

The investigator must collect all the AE, SAE and incidents that occur from the moment the subject signs the informed consent until day 28. On day 60, the investigator must collect follow-up data of AEs that were not resolved on day 28, and SAEs due to hospitalization or death.

## 8.5 Documentation Related to Adverse Events and Incidents

Each AE, SAE or incident to take place during the study will be documented in the medical records of the participant in accordance with standard clinical practice of the investigator. For each SAE, an independent set of SAE form will be used. Only if there are multiple SAE at the time of the initial report and these are temporary and / or clinically interrelated can be registered on the same set of SAE form.

The investigator should try to make a diagnosis of the event based on the signs, symptoms and / or other clinical information. An AE diagnosis must be recorded per line, or a sign/symptom if the diagnosis is not available. If a diagnosis subsequently becomes available, this then should be entered, and the sign/symptom crossed out.

SAE

by the investigator before being sent to sponsor. In the initial page of the SAE form, the investigator must provide his/her opinion regarding the relationship of the event to the study intervention.

## 8.6 Pregnancy

Cases of pregnancy will be recorded as AE and should only be considered as SAE only if they meet any seriousness criteria. Pregnancy will also be a protocol deviation. The investigator will provide the necessary medical advice to the pregnant participant.

No special measures are required in relation to the pregnancy of a partner of a male participant.

## 8.7 Procedure for Adverse Event Reporting

### *8.7.1 Investigator*

The recording of AEs, SAEs and incidences is the responsibility of the study investigator team, which should indicate the time of appearance of the event (expressed in the shortest time unit possible), its serious / not serious status, and in case it is considered related to investigational products, whether it was expected or unexpected. The intensity of the event (grade 1 to 5) is to be specified, along with the measures adopted (none, treatment, temporal or permanent discontinuation of investigational product), course (complete remission, partial remission, persistence) and causality based on the criteria indicated in section 8.3.3.

All AEs, SAEs and incidences will be recorded, regardless of the causality or outcome, in the corresponding form of the eCRF of each participant. Depending on the nature of the event, each AE will be classified as:

- Serious / non-serious
- Related expected / related unexpected

The investigator will immediately notify the study sponsor of any SAE, any related AE, or any incidence. The notification will be performed within 24 hours of first knowledge by the investigator.

Contact Details for Sponsor:

safety@flsrs.com

#### *8.7.2 Sponsor*

The sponsor will inform the relevant Ethics Committee about any important information of security of the investigational product.

The sponsor will inform the relevant Ethics Committee of any SUSAR which may be related to the investigational product.

The deadlines to notify a SUSAR is, from the first knowledge by the sponsor:

1. 15 days
2. 7 days if the SUSAR has resolved in death or has been life-threatening. Relevant follow-up information for these cases will be subsequently submitted within an additional 8 days.

The sponsor will inform the hemovigilance service of any AE, or any incidence related to the infusion.

The sponsor will keep a detailed register of all the AEs and SAEs notified by the investigators and all AEs, SAEs and incidents will be listed in table form in the final report of the study.

### 8.8 Data and Safety Monitoring Board

An independent Data and Safety Monitoring Board (DSMB) has been convened to assess the progress of this clinical study, the safety data, and critical efficacy endpoints and provide recommendations to the study PI. The members of the DSMB serve in an individual capacity and provide their expertise and recommendations. The DSMB will review cumulative study data to evaluate safety, efficacy, study conduct, and scientific validity and data integrity of the study.

The composition, responsibilities and independence of the DSMB, as well as the planned meetings and communication flow, will be detailed in ANNEX 7.

# 9 STATISTICAL ANALYSIS

## 9.1 Methods

### 9.1.1 Descriptive Analysis

The characteristics of the study population will be described using frequencies for categorical variables and using mean and standard deviation for quantitative variables.

### 9.1.2 Bivariate Analysis

We will use the chi-square test for categorical variables and the t-test and analysis of variance for continuous variables.

### 9.1.3 Multivariate Analysis

Event rate comparison between groups

Hospitalization and/or death rate between groups will be analysed using the odds ratio (OR) obtained by fitting a logistic regression model.

Longitudinal VL decay

Efficacy will be determined by comparing the mean reduction of the viral load from baseline to days 7 and 28, with the use of a linear/non-linear mixed effect model taking into account the randomization group and repeated measures within each individual. The viral load will be provided in logarithmic scale; if less than 15% of specimens presents undetectable viral load at a given follow-up assessment a value of 3 $\log_{10}$ copies per mL (i.e., lower limit of detection) will be assigned for the purpose of statistical analysis. Otherwise, we will consider undetectable viral loads as censored values.

Mean change differences between groups

The secondary clinical outcome regarding between-group differences in symptoms severity score will be assessed by means of linear regression.

Time to event analysis

The time to complete resolution of symptoms will be analysed using Kaplan-Meier survival functions and hazard ratios (HRs), calculated using a Cox proportional hazards regression model based on the assumptions of proportional risks. Kaplan-Meier estimates will be compared using the log-rank test.

Variable selection

All models will be adjusted with basal covariates chosen according to their clinical relevance or f the observed correlation with the study outcome, and variable selection procedure will be done using the AIC-based stepwise algorithm.

Missing data and outliers

No missing data imputation method will be used in this study and we will not analyse the presence of potential outliers.

All analyses will be done with the R statistical package, version 6.3 or higher under a significance level of 0.05.

## 9.2 Analysis Population

The primary efficacy analysis will be performed on the intention-to-treat (ITT) population, which will include all randomized participants. If deemed necessary, sensitivity analyses will be performed with

the per-protocol (PP) population. Safety will be assessed in the safety population, which will include all participants who received investigational product (convalescent plasma or placebo).

## 9.3 Sample Size

A sample size of 237 cases per arm would provide the trial with 80% power to detect 50% reduction in hospitalization rate at day 28 after starting the treatment, assuming an expected rate of hospitalization of 15%, allowing a 5% of loss to follow-up.

Approximately 157 cases per arm are required to have 80% power to detect a minimal expected difference of 5

Approximately 150 cases per arm are required to have 80% power to detect a difference of 0.5 log10 in the mean reduction of SARS-CoV-2 viral load at a two-
an expected overall standard deviation of 1.5. A 0.5 $\log_{10}$ copies/mL difference in reduction was chosen to represent the minimal threshold for a biologically relevant change for our analyses.

For the substudy for quantification of neutralizing antibodies against SARS-CoV-2 a total sample size of 135 was estimated considering parameters detailed in the rationale.

## 9.4 Interim Analyses

An interim analysis of efficacy and safety variables will be performed after the first 60 participants achieve the primary endpoint (i.e., day 28) for the purpose of sample size recalculation. The interim analyses will be performed using blinded data, unless otherwise indicated by the DSMB.

## 9.5 Deviation of Statistical Plan

Any deviation from that presented statistical plan will be described and justified in the final study report.

## 10 DIRECT ACCESS TO SOURCE DATA/DOCUMENTS

Investigators and institutions will allow the monitoring, audits by the sponsor giving direct access to data and original source documents.

Access to personal participant information will be restricted to the study investigator and staff. To allow monitoring, audits and inspections, access to data to the Ethics Committee and personnel authorized by the sponsor, is guaranteed while maintaining the confidentiality thereof according to current legislation, in accordance with the Data Protection Law (LOPD, the organic Law 3/2018 of 5 December on the Protection of Personal Data and the Guarantee of Digital Rights complementary to the Regulation (EU) 2016/679 of the European Parliament and of the Council of 27 April 2016, on the protection of natural persons with regard to the processing of personal data and on the free movement of such data).

The principal investigator will have overall control of, and will act as the custodian for all data for the full duration of the study.

# 11 QUALITY CONTROL AND QUALITY ASSURANCE

## 11.1 Study Monitoring

In accordance with applicable regulations and Good Clinical Practice (GCP), monitoring visits will be performed during the study.

Risk-adjusted monitoring will be carried out since the study is performed in a clinical care practice setting, with follow-up of the subjects treated in the community or primary care setting.

The goal of the monitoring activity is to verify that:

- the rights and welfare of the participants are respected;
- data collected are accurate, complete and verifiable with the help of original documents;
- the study is performed according to the protocol and any amendment adopted, GCPs and regulations.

The investigator must agree to:

- grant to monitor direct access to all relevant documentation;
- devote part of his/her time and staff time to the monitor in order to discuss the results of the monitoring, as well as any other possible aspect.

Data monitoring tasks will be set up in the Monitoring Plan, in general:

1. Verification of the study master file (approvals, protocol, investigational product information and other essential documents, pursuant to section 8 of ICH Guide E6).
2. Verification of signature of informed consent.
3. Check of dates of visit and source data verification
4. Detection of inconsistencies in the eCRF by review of data in the medical records.

## 11.2 Protocol Deviations

Major or critical deviations (i.e., that may have an impact on participant safety and data integrity) will be notified immediately to the sponsor. The sponsor will review all deviations from the protocol and assess whether any represents a serious breach of GCP and requires that the Ethics Committee be informed about it. The sponsor should report, when necessary, to the Ethics Committee any serious breach of current legislation or of the version of the protocol approved at the time of the breach that has occurred in Spain without delay and not later than 7 calendar days from becoming aware of the breach.

## 11.3 Audits and Inspections

The sponsor can carry out an audit of quality control at its sole discretion. In this case, the investigator should agree to grant the auditor direct access to all relevant documentation and devote part of his/her time and staff time to the auditor in order to discuss the results of the monitoring, as well as any other possible aspect.

Regulatory authorities may also inspect the study. In this case, the investigator should agree to give the inspector direct access to all relevant documentation and devote part of his/her time and staff time to the inspector in order to discuss the results of the supervision, as well as any other possible aspect.

## 11.4 Data Collection (electronic Case Report Form, eCRF)

Collection of data will be performed using an electronic Case Report Form (eCRF), which will include track changes monitoring (i.e., recording the changes made and details of the user that made these changes). The eCRF will be filled by blinded investigators with a personal system of access by username and password. Participants will also be given access to the eCRF to directly fill the study questionnaires, which will be reviewed by the study investigators.

The eCRF will be developed by BioClever 2005 S.L.U., after proper agreement with the sponsor.

Electronic devices will be used to collect the study data during home visits and telephone visits.

Paper questionnaires will be made available to participants without access to electronic devices or difficulties to use them, and they will also be available in case of connection problems. Data of paper questionnaires will be filled by participants and study staff will introduce it to the eCRF. Data collected will be reviewed by the investigator in the follow-up visits and collection of paper forms will be performed at day 7 by study staff and at day 15 by study staff or courier. The paper source will be verified by the study monitor.

## 11.5 Data Management

A data management system will be set up and procedures will be implemented to warrant homogenization, traceability, and data quality. Data will be entered in a study specific eCRF. Quality control procedures will be put in place for data checking. Rigorous consistency checks will be created in order to reduce errors during data entry.

Data management will be performed by BioClever 2005 S.L.U., after proper agreement with the sponsor.

Different identification sources will be involved in the study, and candidates will volunteer in a study website that will collect candidate name, surname, address and contact information. These data will be centralized in order to avoid informal personal data transfers between the different services involved, and will only be accessible to the investigators and study staff for the purposes of identification and contact. These data will be separated from the study eCRF.

In the study eCRF data will be collected and stored in a dissociated manner, without including any personal data. This database will be accessible to the sponsor, the data management team, the investigators, the study staff with data entry privileges and the participants. Participants will only be granted access to their own symptoms and safety diary card. The tools used to identify individuals may have individual identifiers, but this information will only be associated with an identification number (i.e., participant ID). This information will uniquely identify participants and will be associated with the rest of the captured data for the study.

For data safety and audit trail purposes each person using any of the defined study databases will be required to define clear data access. Individual user/password codes will be available for each person with access privileges and different roles will be established for data entry and/or revision.

Data collected through the eCRF will be stored in a study database, that will be hosted at a secure data center with appropriate series of protocols to test and maintain network security, and to provide access management policies for network drives, databases and remote access.

Data management team and investigators will be the only ones to access the database. The backup of the data will be done on a timely basis. The final data for the analysis will be placed on the FLS server and will be anonymous; If information that could enable to identify individuals has to be stored, used

or shared, it will be encrypted. Consequently, those receiving the final data for analysis will not have access to any information that might help to physically identify individuals.

# 12 ETHICS

## 12.1 General Considerations

The clinical trial will be conducted according to the principles of the Declaration of Helsinki, Fortaleza, Brazil, October 2013.

This study will be conducted according to Spanish regulations regarding clinical studies without medicines (Orden SAS/3470/2009) and biomedical investigations (Law 14/2007 of biomedical investigation and the Royal Decree 1716/2011). The required documentation prior to the start will be:

- Protocol acceptance by the sponsor and the coordinating investigator
- Protocol approval by the Ethics Committee.

All participants will be guaranteed continued medical and nursing supervision throughout the duration of the study.

This study will conform to the standards of GCP published by ICH (E6 R2). Confidentiality requirements will follow the required Data Protection legislation (see section 13).

## 12.2 Participant Information Sheet and Informed Consent

The investigator will inform the candidates of the nature, duration, and purpose of this study and, in addition, of all the inconveniences and obstacles that, if any, can be expected. In addition, information will be provided to the participant. Subjects must have the legal capacity to give their consent and exercise their freedom of decision.

In order to minimize the transfers of COVID-19 patients, given the risk of contagion that this would entail, eligibility will be checked initially by phone before scheduling a hospital visit. An authorization from the candidates will be obtained over the phone to access their shared medical records (Historia Clínica Compartida de Catalunya, HC3) and check their suitability for the study. Candidates identified through the website will have authorized to obtain personal data and access to medical records when registering at the website. A confirmation e-mail including the study privacy policy will be sent.

During the inclusion/baseline visit, candidates will be informed in person of study details and the informed consent will be obtained (i.e., subjects will sign the informed consent form). The investigator will keep a call record of the informed consent process.

In the substudy participants, substudy informed consent will be obtained.

## 13 DATA HANDLING AND RECORD KEEPING

### 13.1 Data Handling

The processing of the data will be subject to current legislation as regards data protection (LOPD, The Organic Law 3/2018 of 5 December on the Protection of Personal Data and the Guarantee of Digital Rights complementary to the Regulation (EU) 2016/679 of the European Parliament and of the Council of 27 April 2016, on the protection of natural persons with regard to the processing of personal data and on the free movement of such data).

The responsible for the personal data registered will be the Delegado de Protección de Datos (DPD) in the Departamento de Salud (dpd@ticsalutsocial.cat). The responsible of the codified study data will be each of the centres involved in the study.

The eCRF and data management will be performed by BioClever 2005 S.L.U., after proper agreement with the sponsor, where the sponsor (FLS and HUGTiP) will be data controller and BioClever 2005 S.L.U. will be data processor. Data servers will be located in Europe.

Data transmitted to third countries and other countries will in no case contain personal data. In the event that such transfer occurs, it will be for the same purposes of the study described and ensuring confidentiality at least to the level of protection of the law in Spain.

The participant will be identified in the records by the corresponding unique participant ID. The participant is to be guaranteed anonymity and is to be informed that all communication will take place between him/her and the investigator and not the sponsor of the study.

### 13.2 Record Keeping

#### *13.2.1 Investigator File and Document Retention*

The investigator must keep the investigator file with the proper and accurate records to enable the study to be fully documented and data subsequently verified.

forms, EC approval samples of the patient information sheet and informed consent, staff curriculum,

correspondence.

Clinical source documents from subjects (usually predefined by the project to record efficacy and safety parameters or documents that are not in the clinical record of the hospital) will be filed when necessary indicating the participant ID without personal data.

The investigator should retain these documents at least five years, provided that the sponsor does not express another period.

#### *13.2.2 Source Documents and Basic Data*

Participation in the study will be included on the participant medical records, including assigned participant ID and identification of the different study visits that will take place throughout the study. At the end of the study, a copy of the eCRF will be placed on the site.

# 14 FINANCING AND INSURANCE

## 14.1 Source of Financing

The funding source is the Fundació FLS de Lluita contra la Sida, les Malalties Infeccioses i la Promoció de la Salut i La Ciència, Hospital Germans Trias i Pujol and Grifols.

## 14.2 Insurance Policy

The study sponsor has a policy of liability insurance. The sponsor shall extend this policy or another with equivalent coverage until the end of the trial. This policy also covers the responsibilities of the sponsor, the principal and his/her collaborators, as well as the hospital or site where they carry out the study.

## 15 PUBLICATION POLICY

The sponsor and the principal investigator aim to publish the results of this study in international peer reviewed journals.

## 16 REFERENCES


1. WHO Interim guidance 13 August 2020. https://www.who.int/emergencies/diseases/novel-coronavirus-2019?gclid=CjwKCAjwydP5BRBREiwA-qrCGrDAeovgOiCboUh2dKJhptzKhJd9Tkha3iH1ZOiKAfe9nOkDggkXjxoC9rsQAvD_BwE
2. Recommendations for Investigational COVID-19 Convalescent Plasma. US FDA. Published May 1, 2020. Accessed July 27th, 2020. https://www.fda.gov/vaccines-blood-biologics/investigational-new-drug-ind-or-device-exemption-ide-process-cber/recommendations-investigational-covid-19-convalescent-plasma
3. Nguyen AA, Habiballah SB, Platt CD, Geha RS, Chou JS, McDonald DR. Immunoglobulins in the treatment of COVID-19 infection: Proceed with caution!. Clin Immunol. 2020;216:108459. doi:10.1016/j.clim.2020.108459
4. Li L, Zhang W, Hu Y, et al. Effect of Convalescent Plasma Therapy on Time to Clinical Improvement in Patients With Severe and Life-threatening COVID-19: A Randomized Clinical Trial [published online ahead of print, 2020 Jun 3]. JAMA. 2020;e2010044. doi:10.1001/jama.2020.10044
5. Liu, S. T.H., Lin, H.-M. et al. Convalescent plasma treatment of severe COVID-19: A marched control study. MedRxiv, 2020.05.20.20102236. https://doi.org/10.1101/2020.05.20.20102236
6. Joyner, M. J., Klassen, S. A., Senefeld, J., et al. Evidence favouring the efficacy of convalescent plasma for COVID-19 therapy. MedRxiv 2020.07.29.20162917. https://doi.org/10.1101/2020.07.29.20162917
7. Tanne JH. Covid-19: FDA approves use of convalescent plasma to treat critically ill patients. BMJ. 2020;368:m1256. Published 2020 Mar 26. doi:10.1136/bmj.m1256.
8. Valk SJ, Piechotta V, Chai KL, et al. Convalescent plasma or hyperimmune immunoglobulin for people with COVID-19: a rapid review. Cochrane Database Syst Rev. 2020;5(5):CD013600. Published 2020 May 14. doi:10.1002/14651858.CD013600
9. Joyner MJ, Bruno KA, Klassen SA, et al. Safety Update: COVID-19 Convalescent Plasma in 20,000 Hospitalized Patients [published online ahead of print, 2020 Jul 19]. Mayo Clin Proc. 2020;doi:10.1016/j.mayocp.2020.06.028
10. COVID-19 treatment guidelines. NIH. https://www.covid19treatmentguidelines.nih.gov/immune-based-therapy/
11. Wan Y, Shang J, Sun S, et al. Molecular Mechanism for Antibody-Dependent Enhancement of Coronavirus Entry. J Virol. 2020;94(5):e02015-19. Published 2020 Feb 14. doi:10.1128/JVI.02015-19
12. Mair-Jenkins J, Saavedra-Campos M, Baillie JK, et al. The effectiveness of convalescent plasma and hyperimmune immunoglobulin for the treatment of severe acute respiratory infections of viral etiology: a systematic review and exploratory meta-analysis. J Infect Dis. 2015;211(1):80-90. doi:10.1093/infdis/jiu396
13. Casadevall A, Pirofski LA. The convalescent sera option for containing COVID-19. J Clin Invest. 2020;130(4):1545-1548. doi:10.1172/JCI138003
14. van Griensven J, Edwards T, Gallian P; Ebola-Tx Consortium. Convalescent Plasma for Ebola Virus Disease. N Engl J Med. 2016;374(25):2500. doi:10.1056/NEJMc1602284
15. WHO Working Group on the Clinical Characterisation and Management of COVID-19 infection. A minimal common outcome measure set for COVID-19 clinical research. Lancet Infect Dis. 2020;20(8):e192-e197. doi:10.1016/S1473-3099(20)30483-7

16. Pujadas E, Chaudhry F, McBride R, et al. SARS-CoV-2 Viral Load Predicts COVID-19 Mortality. medRxiv 2020.06.11.20128934; doi: https://doi.org/10.1101/2020.06.11.20128934
17. Han A, Poon JL, Powers JH 3rd, Leidy NK, Yu R, Memoli MJ. Using the Influenza Patient-reported Outcome (FLU-PRO) diary to evaluate symptoms of influenza viral infection in a healthy human challenge model. BMC Infect Dis. 2018;18(1):353. Published 2018 Jul 28. doi:10.1186/s12879-018-3220-8
18. Powers JH, Guerrero ML, Leidy NK, et al. Development of the Flu-PRO: a patient-reported outcome (PRO) instrument to evaluate symptoms of influenza. BMC Infect Dis. 2016;16:1. Published 2016 Jan 5. doi:10.1186/s12879-015-1330-0
19. Kermali, M., Khalsa, R. K., Pillai, K., Ismail, Z., & Harky, A. (2020). The role of biomarkers in diagnosis of COVID-19 – A systematic review. In Life Sciences (Vol. 254, Issue January, p. 117788). https://doi.org/10.1016/j.lfs.2020.117788
20. Pelegrin M, Naranjo-Gomez M, Piechaczyk M. Antiviral Monoclonal Antibodies: Can They Be More Than Simple Neutralizing Agents? Trends Microbiol. 2015; 23:653665.
21. Schoofs T, Klein F, Braunschweig M, Kreider EF, Feldmann A, Nogueira L, et al. HIV-1 therapy with monoclonal antibody 3BNC117 elicits host immune responses against HIV-1. Science (80-) 2016; 352:9971001.
22. To KK, Tsang OT, Yip CC, et al. Consistent Detection of 2019 Novel Coronavirus in Saliva. Clin Infect Dis. 2020;71(15):841-843. doi:10.1093/cid/ciaa149
23. Tu YP, Jennings R, Hart B, et al. Swabs Collected by Patients or Health Care Workers for SARS-CoV-2 Testing. N Engl J Med. 2020;383(5):494-496. doi:10.1056/NEJMc2016321
24. McCulloch DJ, Kim AE, Wilcox NC, et al. Comparison of Unsupervised Home Self-collected Midnasal Swabs With Clinician-Collected Nasopharyngeal Swabs for Detection of SARS-CoV-2 Infection. JAMA Netw Open. 2020;3(7):e2016382. Published 2020 Jul 1. doi:10.1001/jamanetworkopen.2020.16382

# 17 ANNEXES

8. ANNEX 1: DONOR SELECTION, COLLECTION AND PROCESSING PROCEDURES FO CONVALESCENT MBT PLASMA PREPARATION FROM DONORS RECOVERED FROM COVID-19

9. ANNEX 1.1.: CRITERIA FOR ACCEPTING / EXCLUDING DONORS OF WHOLE BLOOD AND BLOOD COMPONENTS

Attached separately, corresponding to the document DI-EM-
the BST.

10. ANNEX 1.2: UPDATE OF THE PROTOCOL FOR THE SELECTION OF COVID-19 CONVALESCENT PLASMA DONORS

Attached separately, corresponding to the document NT-DI-EM-

11. ANNEX 1.3.: REGISTER OF THE ASSESSMENT OF COVID-19 CONVALESCENT PLASMA DONOR

Attached separately, corresponding to the BST register form.

12. ANNEX 1.4.: CONVALESCENT LABEL TEMPLATE E9744

Attached separately, corresponding to the E9744 BST label for MBT convalescent plasma.

13. ANNEX 1.5.: METHYLENE BLUE INACTIVATION PROCESS

Attached separately, corresponding to the document FR-T
BST.

## 14. ANNEX 2: WHO CLINICAL PROGRESSION SCALE

| Patient State | Descriptor | Score |
|---|---|---|
| Uninfected | Uninfected; no viral RNA detected | 0 |
| Ambulatory mild disease | Asymptomatic; viral RNA detected | 1 |
| | Symptomatic; independent | 2 |
| | Symptomatic; assistance needed | 3 |
| Hospitalised: moderate disease | Hospitalised; no oxygen therapy* | 4 |
| | Hospitalised; oxygen by mask or nasal prongs | 5 |
| Hospitalised: severe diseases | Hospitalised; oxygen by NIV or high flow | 6 |
| | Intubation and mechanical ventilation, $pO_2/FiO_2 \geq 150$ or $SpO_2/FiO_2 \geq 200$ | 7 |
| | Mechanical ventilation $pO_2/FIO_2 < 150$ ($SpO_2/FiO_2 < 200$) or vasopressors | 8 |
| | Mechanical ventilation $pO_2/FiO_2 < 150$ and vasopressors, dialysis, or ECMO | 9 |
| Dead | Dead | 10 |

## 15. ANNEX 3: FLU-PRO© PLUS QUESTIONNAIRE

**Marcar los síntomas actuales**

Nos gustaría que nos informara sobre los síntomas que le han afectado en las últimas 24 horas.

Para cada síntoma, marque la casilla situada debajo de la respuesta que mejor describa su experiencia. Marque la casilla que dice "En absoluto", si no ha tenido ese síntoma en las últimas 24 horas

**¿Qué hora es? ______ □ Mañana □ Tarde □ Noche**

**Indique en qué medida ha tenido cada síntoma en las últimas 24 horas.**

| | | **En absoluto** | **Un poco** | **Algo** | **Bastante** | **Muchísimo** |
|---|---|---|---|---|---|---|
| | | | | | | |
| 1 | Moqueo o goteo nasal | ☐ | ☐ | ☐ | ☐ | ☐ |
| 2 | Congestión nasal o nariz taponada | ☐ | ☐ | ☐ | ☐ | ☐ |
| 3 | Presión sinusal | ☐ | ☐ | ☐ | ☐ | ☐ |
| | | | | | | |
| 4 | Picor de garganta | ☐ | ☐ | ☐ | ☐ | ☐ |
| 5 | Molestias o dolor de garganta | ☐ | ☐ | ☐ | ☐ | ☐ |
| 6 | Dificultad para tragar | ☐ | ☐ | ☐ | ☐ | ☐ |
| | | | | | | |
| 7 | Ojos llorosos o acuosos | ☐ | ☐ | ☐ | ☐ | ☐ |
| 8 | Molestias o dolor de ojos | ☐ | ☐ | ☐ | ☐ | ☐ |
| 9 | Ojos sensibles a la luz | ☐ | ☐ | ☐ | ☐ | ☐ |
| | | | | | | |
| 10 | Dificultad para respirar | ☐ | ☐ | ☐ | ☐ | ☐ |
| 11 | Congestión en el pecho | ☐ | ☐ | ☐ | ☐ | ☐ |
| 12 | Opresión en el pecho | ☐ | ☐ | ☐ | ☐ | ☐ |
| 13 | Tos seca | ☐ | ☐ | ☐ | ☐ | ☐ |
| 14 | Tos productiva o con flemas | ☐ | ☐ | ☐ | ☐ | ☐ |
| | | | | | | |
| 15 | Sintió nauseas (con la sensación de que quería devolver) | ☐ | ☐ | ☐ | ☐ | ☐ |
| 16 | Dolor de estómago | ☐ | ☐ | ☐ | ☐ | ☐ |
| | | | | | | |
| 17 | Se sintió mareado | ☐ | ☐ | ☐ | ☐ | ☐ |

| | | | | | | |
|---|---|---|---|---|---|---|
| 18 | Congestión de cabeza | ☐ | ☐ | ☐ | ☐ | ☐ |
| 19 | Dolor de cabeza | ☐ | ☐ | ☐ | ☐ | ☐ |
| 20 | Falta de apetito | ☐ | ☐ | ☐ | ☐ | ☐ |
| 21 | Dormir más de lo habitual | ☐ | ☐ | ☐ | ☐ | ☐ |
| 22 | Dolores o molestias en el cuerpo | ☐ | ☐ | ☐ | ☐ | ☐ |
| 23 | Débil o cansado | ☐ | ☐ | ☐ | ☐ | ☐ |
| 24 | Escalofríos o tiritona | ☐ | ☐ | ☐ | ☐ | ☐ |
| 25 | Sintió frío | ☐ | ☐ | ☐ | ☐ | ☐ |
| 26 | Sintió calor | ☐ | ☐ | ☐ | ☐ | ☐ |
| 27 | Sudores | ☐ | ☐ | ☐ | ☐ | ☐ |

*** En absoluto (0), Un poco (1), Algo (2), Bastante (3), Muchísimo (4). Si alguno de los síntomas es igual a Muchísimo (4) registrado por primera vez, COORDINACIÓN recibirá una alerta (por email) para evaluar mediante una visita telefónica la programación de una visita presencial (VISITA DOMICILIARIA EXTRA)**

**En las últimas <u>24 horas</u>, ¿<u>con qué frecuencia</u> ha tenido alguno de los síntomas siguientes?**

| | | Nunca | Rara vez | Algunas veces | A menudo | Siempre |
|---|---|---|---|---|---|---|
| 28 | Estornudos | ☐ | ☐ | ☐ | ☐ | ☐ |
| 29 | Tos | ☐ | ☐ | ☐ | ☐ | ☐ |
| 30 | Tosió mocos o flemas | ☐ | ☐ | ☐ | ☐ | ☐ |

***Nunca (0), Rara vez (1), Algunas veces (2), A menudo (3), Siempre (4). Si alguno de los síntomas es igual a Siempre (4) registrado por primera vez, COORDINACIÓN recibirá una alerta (por email) para evaluar mediante una visita telefónica la programación de una visita presencial (VISITA DOMICILIARIA EXTRA)**

| | | 0 veces | 1 vez | 2 veces | 3 veces | 4 o más veces |
|---|---|---|---|---|---|---|
| 31 | ¿Cuántas veces ha vomitado? | ☐ | ☐ | ☐ | ☐ | ☐ |
| 32 | ¿Cuántas veces ha tenido diarrea? | ☐ | ☐ | ☐ | ☐ | ☐ |

***En las últimas 24h, ¿ha tenido alguno de los síntomas siguientes?***

| | | No | Si |
|---|---|---|---|
| 33 | Pérdida del olfato | ☐ | ☐ |
| 34 | Pérdida del gusto | ☐ | ☐ |

***No (0), Si (1)**

# 16. ANNEX 4: ASSESSMENT OF SAFETY

## Safety Diary Card

OTROS SÍNTOMAS:

**¿Ha notado alguno de los siguientes síntomas en las últimas 24 horas?**

| | En absoluto | Un poco | Algo | Bastante | Muchísimo |
|---|---|---|---|---|---|
| Dolor local en el lugar de infusión | | | | | |
| Inflamación lugar de infusión | | | | | |
| Erupción cutánea | | | | | |
| Prurito o Picor | | | | | |

*** En absoluto (0), Un poco (1), Algo (2), Bastante (3), Muchísimo (4). Si se registra alguno de estos síntomas por primera vez, COORDINACIÓN recibirá una alerta (por email) para evaluar mediante una visita telefónica dicha sintomatología y su gravedad.**

**Aparte de los síntomas preguntados anteriormente, ¿ha notado algún otro síntoma?:** □ NO□ SI

**En caso afirmativo, ¿qué otro/s síntomas ha notado?**

| SÍNTOMA | Fecha y hora de inicio | Fecha y hora de fin | Severidad | | | |
|---|---|---|---|---|---|---|
| | | | Un poco | Algo | Bastante | Muchísimo |
| | | | | | | |
| | | | | | | |
| | | | | | | |

## Telephone Safety Assessment

EVENTOS ADVERSOS:

En caso de ser un evento grave o inesperado, llamar a COORDINACIÓN y completar el apartado I del documento de SAE en papel, fotografiarlo y enviarlo per baliza y/o correo electró[illegible] safety@flsrs.com

| Tipo de EVENTO ADVERSO | (0) NO (1) SI | Fecha y hora de inicio | Fecha y hora de fin | Grado 1-5 (*) | Relacionado (SI/NO) | Decisión investigador |
|---|---|---|---|---|---|---|
| Erupción cutánea | | | | | | |
| Prurito o Picor | | | | | | |
| Dolor local en el lugar de infusión | | | | | | |
| Inflamación lugar de infusión | | | | | | |
| Fiebre | | | | | | |
| Cefalea | | | | | | |
| Náusea | | | | | | |

| | | | | | | |
|---|---|---|---|---|---|---|
| Vómito | | | | | | |
| Diarrea | | | | | | |
| Dolor abdominal | | | | | | |
| Dolor muscular o articular | | | | | | |
| Tos | | | | | | |
| Disnea | | | | | | |
| Fatiga | | | | | | |
| Mareo | | | | | | |
| Somnolencia | | | | | | |
| Otro: | | | | | | |

*** Grado 1 = Leve, 2=Moderado, 3=Severo, 4=potencialmente mortal, 5= Muerte. Guía de soporte para puntuar grados estará disponible para todo el personal médico del estudio**

## 17. ANNEX 5: HEMOVIGILANCE: DETECTION, ASSESSEMENT AND REGISTER OF INCIDENTS RELATED TO TRANSFUSION

Attached separately.

## 18. ANNEX 6: WEB CONTENT

Attached separately.

## 19. ANNEX 7: COMPOSITION, RESPONSIBILITIES AND FLOW CHART FOR DSMB

Attached separately.

## 20. ANNEX 8: LIST OF PARTICIPATING SITES

Attached separately.

Version 2.0 10th December 2020

| Site | Principal Investigator | Status |
|---|---|---|
| Hospital Germans Trias i Pujol | Oriol Mitjà | Submitted to EC. |
| Hospital Universitari de Bellvitge | Carlota Gudiol/ Pierre Malchair | Submitted to EC . |
| Hospital de Sant Joan Despí Moisès Broggi | Ana Coloma | Submitted to EC . |
| CUAP Badalona<br>CUAP Santa Coloma<br>Atenció Primària Àrea Metropolitana Nord de Barcelona | Thatiana Vertiz<br>Núria Prat (investigadora Metropolitana Nord) | Submitted to EC |
| CUAP Manresa<br>Atenció Primària Àrea Catalunya Central | Dra. Anna Maria Ramírez Morros<br>Dra.Anna Ruiz Comellas<br>Anna Forcada (investigadora Central) | Submitted to EC |
| Hospital Sant Bernabé de Berga | Dra. Rosa Amado Simón<br>Joana Rodríguez Codina | Agreement in process. |

## 21. ANNEX 9PARTICIPANT ID CARD

| | |
|---|---|
| **Información para contacto en caso de emergencia:**<br><br>Médico del estudio: .....................................................<br><br>Hospital: .....................................................<br><br>Teléfono de contacto: ................................ | **TARJETA DE IDENTIFICACIÓN**<br><br>**Código: COnV-ert**<br><br>Sr/Sra.<br><br>..............................................................<br><br>ID Participante<br><br>.............................................................. |

| | |
|---|---|
| Apreciado participante:<br><br>Por favor, lleve consigo esta tarjeta e informe a todos los médicos que le visiten durante y tras su participación en este estudio.<br><br>Informe también al equipo investigador si tuviese alguna visita planificada o inesperada en otro hospital o consulta durante el estudio.<br><br>Muchas gracias. | **Información para contacto con el equipo investigador:**<br><br>Si, en una emergencia, fuera necesario saber si el participante ha recibido plasma convaleciente o placebo; o si tuviera cualquier tipo de pregunta, por favor, contacte con:<br><br>Investigadores: Andrea Alemany, Marc Corbacho.<br><br>Dirección: Hospital Germans Trias i Pujol, Badalona.<br><br>T: 611 69 66 79 |

## Summary of changes in Protocol approved versions

| **Section** | **Version 4.0** (28th Oct 2020) | **Version 5.0** (10th Dec 2020) |
|---|---|---|
| 1.8 Technical services and Institutions involved<br><br>+ Study Summary section | | **New laboratory sites are included:** Hospital Universitari de Bellvitge, Hospital Moisès Broggi de Sant Joan Despí, Hospital Sant Bernabé de Berga and Hospital Sant Joan de Déu de Manresa.<br><br>**New blood banks are included:** ABO compatibility test will be assessed at the Banc de Sang i Teixits (BST) or Hematology Department or Laboratory in each of the sites involved, depending on each center's transfusion protocols. |
| 4.2 Trial Design<br><br>5.2 Arm description<br><br>5.4 Dose, interval, route and method of administration<br><br>7.1 Study visits and procedures<br><br>+ Study Summary section | Interventional product volume 200-250 mL | Interventional product **volume 200-300 mL** |
| 4.2 Trial Design<br><br>7.1 Study visits and procedures<br><br>7.5 Schedule of procedures<br><br>+ Study Summary section | Day 7 visit is performed at home | **Day 7 visit is performed either at home or at study site (hospital).** |
| 6.1 Study population<br><br>+ Study Summary section | Individuals from Metropolitana Nord Area of Catalonia | **Individuals from different regions of Catalonia.** |
| 6.3 Exclusion criteria<br><br>+ Study Summary section | Medical conditions for which 200-250 mL of intravenous fluid is considered dangerous. | Medical conditions for which **200-300 mL** of intravenous fluid is considered dangerous. |
| 7.1. Study visits and procedures<br><br>+ Study Summary section | | **New recruitment procedure is added:** Invitation by telephone call in a follow-up visit to patients with confirmed SARS-CoV-2 infection who appear in a list of positive PCR or rapid antigen test from the laboratory of the participating sites. |

| | | |
|---|---|---|
| 7.3 Description of Laboratory tests and procedures<br><br>+ Study Summary Section | | **new study sites.** |
| 13.1 Data handling and record keeping<br><br>+ Study Summary Section | | **Sentence is added:** The responsible for the personal data registered will be the Delegado de Protección de Datos (DPD) in the Departamento de Salud (dpd@ticsalutsocial.cat). The responsible of the codified study data will be each of the centres involved in the study. |
| Annex 8: list of participating sites | | **New study sites with respective site principal investigators are added.** |
| PATIENT INFORMATION SHEET AND INFORMED CONSENT | | New study sites are added.<br><br>A sentence is added to clarify that the trip expenses to and from the study sites are not covered.<br><br>A sentence is added to clarify that the participants should attend their reference health centre in case of emergency and they should show the study identification card.<br><br>A new laboratory name where leftover samples could be kept is added.<br><br>A sentence is added to clarify that the responsible for the personal data registered will be the Delegado de Protección de Datos (DPD) in the Departamento de Salud (dpd@ticsalutsocial.cat).<br><br>Name and phone number for study team and principal investigator of each of the sites are added. |
| SUBESTUDY PATIENT INFORMATION SHEET AND INFORMED CONSENT | | A sentence is added to clarify that the trip expenses to and from the study sites are not covered.<br><br>A sentence is added to clarify that the participants should attend their reference health centre in case of emergency and they should show the study identification card.<br><br>A sentence is added to clarify that the responsible for the personal data registered will be the Delegado de Protección de Datos (DPD) in the Departamento de Salud (dpd@ticsalutsocial.cat). |

# STATISTICAL ANALYSIS PLAN

| | |
|---|---|
| Trial Name: | COnV-ert |
| Trial Title: | Convalescent Methylene Blue Treated (MBT) Plasma for Early Treatment in Non-hospitalised Mild or Moderate COVID-19 Patients: a Randomized Double Blind Study. |
| Protocol No.: | -- |
| EudraCT No.: | -- |

| *Reference to version and date of protocol and SAP on which report is based* | |
|---|---|
| Protocol version: | 4.0 |
| Protocol date: | 28th, October 2020 |

| | |
|---|---|
| Date: | 25th, August 2021 |
| Version: | 1.0 |

| Performed by: | Performed for: |
|---|---|
| BIOCLEVER | FUNDACIÓ LLUITA CONTRA LA SIDA |

All rights reserved. No part of this material may be reproduced or copied in any form by any means (written, electronic, photocopying, recording or otherwise) without the prior consent of BioClever.

-82:1547 .7;36893 0-) ,#%"//*$ ,%%" $',' ($! &,*./,&+'%+'



## Index


**1. Approvals (signatures) .......... 12**

**2. Distribution List .......... 13**

**3. Document Version History .......... 13**

3.1 Changes to the protocol .......... 13

3.2 Documentation history .......... 13

**4. Introduction .......... 14**

4.1 Background and rationale .......... 14

4.2 Objectives .......... 14

4.2.1 Primary objective .......... 14

4.2.2 Secondary objectives .......... 15

**5. Clinical trial methodology .......... 15**

5.1 Study design .......... 15

5.2 Randomisation .......... 16

5.3 Sample size .......... 16

**6. General statistical considerations .......... 17**

6.1 Study population .......... 17

6.2 General issues .......... 17

6.3 Presentation/Format of results .......... 17

6.4 Level of significance .......... 18

6.5 Handling of dropouts and missing data .......... 18

**7. Statistical analysis .......... 18**

7.1 Definitions of variables .......... 18

7.2 Hypothesis and statistical methods .......... 21

7.2.1 Univariate analysis. Descriptive statistics .......... 21

7.2.2 Bivariate analysis .......... 21

7.2.3 Multivariate analysis .......... 21

7.3 Demographic and baseline characteristics .......... 22

7.4 Efficacy analysis .......... 23


All rights reserved. No part of this material may be reproduced or copied in any form by any means (written, electronic, photocopying, recording or otherwise) without the prior consent of BioClever.

7.4.1 Primary endpoints ... 23
7.4.2 Secondary endpoints ... 24
7.5 Safety analysis ... 27
7.6 Other analysis ... 27
**8. Refences ... 29**
**9. List of tables that will be presented in the report ... 30**
9.1 Analysis sets ... 30
9.2 Descriptive analysis ... 34
9.2.1 Descriptive analysis of participant’s baseline characteristics - ITT ... 34
9.2.2 Convalescent plasma infused volume ... 46
9.2.3 Symptoms before treatment infusion ... 47
9.3 Efficacy primary objective ... 52
9.3.1 Intention-to-treat set (ITT) ... 52
9.3.1.1 Hospitalization rate ... 52
9.3.1.2 Viral load ... 55
9.3.2 Per protocol (PP) ... 59
9.3.2.1 Hospitalisation rate ... 59
9.3.2.2 Viral load ... 59
9.4 Efficacy secondary objectives – ITT ... 59
9.4.1 COVID-19 WHO scale score progression ... 59
9.4.2 Time to COVID-19 symptoms resolution ... 71
9.4.3 Death rate ... 72
9.4.4 Inflammatory prognostic markers ... 74
9.5 Safety primary objective - SAF ... 77
9.6 Others analysis ... 81
9.6.1 Worst COVID-19 WHO scale score ... 81
9.6.2 Hospitalisation ... 83


All rights reserved. No part of this material may be reproduced or copied in any form by any means (written, electronic, photocopying, recording or otherwise) without the prior consent of BioClever.

**Table index**

**Tabla 1.1.1 Analysis sets - Patients recruited** ..... 30

**Tabla 1.1.2 Visits - ITT** ..... 31

**Tabla 1.1.3 Extra visits - ITT** ..... 33

**Tabla 2.1.1 Demographic data** ..... 34

**Tabla 2.1.2 Physical characteristics** ..... 35

**Tabla 2.1.3 Vital signs** ..... 37

**Tabla 2.1.4 Risk factors** ..... 38

**Tabla 2.1.5 Women characteristics regarding sterilization, menstruation and pregnancy test result** ..... 44

**Tabla 2.1.6 Covid-19 severity at baseline** ..... 45

**Tabla 2.1.7 Symptoms onset at baseline** ..... 46

**Tabla 2.2.1 Treatment infused volume received** ..... 46

**Tabla 2.3.1 Frequency of Covid-19 symptoms registered by the medical staff** ..... 47

**Tabla 2.3.2 Covid-19 symptoms registered by the medical staff – I – (A)** ..... 48

**Tabla 2.3.3 Covid-19 symptoms registered by the medical staff – II – (A)** ..... 49

**Tabla 2.3.4 Covid-19 symptoms registered by the medical staff – III – (A)** ..... 50

**Tabla 2.3.5 Covid-19 symptoms registered by the medical staff – I – (B)** ..... 51

**Tabla 2.3.6 Covid-19 symptoms registered by the medical staff – II – (B)** ..... 51

**Tabla 2.3.7 Covid-19 symptoms registered by the medical staff – III – (B)** ..... 51

**Tabla 3.1.1 Hospitalization through day 28 per group** ..... 52

**Tabla 3.1.2 Log-binomial regression model 1 – basic model** ..... 52

**Tabla 3.1.3 Log-binomial regression model 2 – one-covariate model** ..... 52

**Tabla 3.1.4 Log-binomial regression model 3 – final selected model** ..... 53

**Tabla 3.1.5 Log-binomial regression model 4 – final model** ..... 53

**Tabla 3.1.6 Hospitalization through day 28 per group - Seropositives** ..... 53

**Tabla 3.1.7 Log-binomial regression model 1 – basic model** ..... 53

**Tabla 3.1.8 Log-binomial regression model 2 – one-covariate model** ..... 53

**Tabla 3.1.9 Log-binomial regression model 3 – final selected model** ..... 53

**Tabla 3.1.10 Log-binomial regression model 4 – final model** ..... 53

**Tabla 3.1.11 Hospitalization through day 28 per group - Seronegatives** ..... 53

All rights reserved. No part of this material may be reproduced or copied in any form by any means (written, electronic, photocopying, recording or otherwise) without the prior consent of BioClever.

**Tabla 3.1.12 Log-binomial regression model 1 – basic model ........................................ 53**

**Tabla 3.1.13 Log-binomial regression model 2 – one-covariate model .......................... 53**

**Tabla 3.1.14 Log-binomial regression model 3 – final selected model........................... 53**

**Tabla 3.1.15 Log-binomial regression model 4 – final model .......................................... 53**

**Tabla 3.1.16 Hospitalization through day 28 per group - ≤ 3 days.......................... 54**

**Tabla 3.1.17 Log-binomial regression model 1 – basic model ........................................ 54**

**Tabla 3.1.18 Log-binomial regression model 2 – one-covariate model .......................... 54**

**Tabla 3.1.19 Log-binomial regression model 3 – final selected model........................... 54**

**Tabla 3.1.20 Log-binomial regression model 4 – final model .......................................... 54**

**Tabla 3.1.21 Hospitalization through day 28 per group - >3 days .................................. 54**

**Tabla 3.1.22 Log-binomial regression model 1 – basic model ........................................ 54**

**Tabla 3.1.23 Log-binomial regression model 2 – one-covariate model .......................... 54**

**Tabla 3.1.24 Log-binomial regression model 3 – final selected model........................... 54**

**Tabla 3.1.25 Log-binomial regression model 4 – final model .......................................... 54**

**Tabla 3.1.26 Hospitalization through day 28 per group – ID50>250................................ 54**

**Tabla 3.1.27 Log-binomial regression model 1 – basic model ........................................ 54**

**Tabla 3.1.28 Log-binomial regression model 2 – one-covariate model .......................... 54**

**Tabla 3.1.29 Log-binomial regression model 3 – final selected model........................... 54**

**Tabla 3.1.30 Log-binomial regression model 4 – final model .......................................... 54**

**Tabla 3.1.31 Hospitalization through day 28 per group – ID50 ≤250................. 54**

**Tabla 3.1.32 Log-binomial regression model 1 – basic model ........................................ 54**

**Tabla 3.1.33 Log-binomial regression model 2 – one-covariate model .......................... 54**

**Tabla 3.1.34 Log-binomial regression model 3 – final selected model........................... 54**

**Tabla 3.1.35 Log-binomial regression model 4 – final model .......................................... 54**

**Tabla 3.1.36 Positives, negatives and undetectables at each time point ........................ 55**

**Tabla 3.1.37 Viral load* reduction by group in logarithm$_{10}$ scale .................................... 56**

**Tabla 3.1.38 Viral load reduction – Model 1 – basic model ............................................. 56**

**Tabla 3.1.39 Viral load reduction – Model 2 – one covariate............................................ 56**

**Tabla 3.1.40 Viral load reduction – Model 3 – final selected model ................................ 57**

**Tabla 3.1.41 Viral load reduction – Model 4 – final model............................................... 57**

**Tabla 3.1.42 Viral load* reduction by group in logarithm$_{10}$ scale - Seropositives .......... 57**


All rights reserved. No part of this material may be reproduced or copied in any form by any means (written, electronic, photocopying, recording or otherwise) without the prior consent of BioClever.

-82:1547 .7;3893 0-) ,#%"//*$ ,%%" $',' ($! &,*./,&+'%+'



**Tabla 3.1.43** **Viral load reduction – Model 1 – basic model ..... 57**
**Tabla 3.1.44** **Viral load reduction – Model 2 – one covariate ..... 57**
**Tabla 3.1.45** **Viral load reduction – Model 3 – final selected model ..... 57**
**Tabla 3.1.46** **Viral load reduction – Model 4 – final model ..... 58**
**Tabla 3.1.47** **Viral load* reduction by group in logarithm$_{10}$ scale - Seronegatives ..... 58**
**Tabla 3.1.48** **Viral load reduction – Model 1 – basic model ..... 58**
**Tabla 3.1.49** **Viral load reduction – Model 2 – one covariate ..... 58**
**Tabla 3.1.50** **Viral load reduction – Model 3 – final selected model ..... 58**
**Tabla 3.1.51** **Viral load reduction – Model 4 – final model ..... 58**
**Tabla 3.1.52** **Viral load* reduction by group in logarithm$_{10}$ scale - ≤ 3 days ..... 58**
**Tabla 3.1.53** **Viral load reduction – Model 1 – basic model ..... 58**
**Tabla 3.1.54** **Viral load reduction – Model 2 – one covariate ..... 58**
**Tabla 3.1.55** **Viral load reduction – Model 3 – final selected model ..... 58**
**Tabla 3.1.56** **Viral load reduction – Model 4 – final model ..... 58**
**Tabla 3.1.57** **Viral load* reduction by group in logarithm$_{10}$ scale - >3 days ..... 58**
**Tabla 3.1.58** **Viral load reduction – Model 1 – basic model ..... 58**
**Tabla 3.1.59** **Viral load reduction – Model 2 – one covariate ..... 58**
**Tabla 3.1.60** **Viral load reduction – Model 3 – final selected model ..... 58**
**Tabla 3.1.61** **Viral load reduction – Model 4 – final model ..... 58**
**Tabla 3.1.62** **Viral load* reduction by group in logarithm$_{10}$ scale – ID50>250 ..... 58**
**Tabla 3.1.63** **Viral load reduction – Model 1 – basic model ..... 58**
**Tabla 3.1.64** **Viral load reduction – Model 2 – one covariate ..... 58**
**Tabla 3.1.65** **Viral load reduction – Model 3 – final selected model ..... 58**
**Tabla 3.1.66** **Viral load reduction – Model 4 – final model ..... 58**
**Tabla 3.1.67** **Viral load* reduction by group in logarithm$_{10}$ scale - ID50 ≤250 ..... 58**
**Tabla 3.1.68** **Viral load reduction – Model 1 – basic model ..... 58**
**Tabla 3.1.69** **Viral load reduction – Model 2 – one covariate ..... 58**
**Tabla 3.1.70** **Viral load reduction – Model 3 – final selected model ..... 58**
**Tabla 3.1.71** **Viral load reduction – Model 4 – final model ..... 58**
**Tabla 3.2.1** **Hospitalization per group ..... 59**
**Tabla 3.2.2** **Log-binomial regression model 1 – basic model ..... 59**

All rights reserved. No part of this material may be reproduced or copied in any form by any means (written, electronic, photocopying, recording or otherwise) without the prior consent of BioClever.

**Tabla 3.2.3 Log-binomial regression model 2 – one-covariate model............................ 59**

**Tabla 3.2.4 Log-binomial regression model 3 – final selected model ............................ 59**

**Tabla 3.2.5 Log-binomial regression model 4 – final model........................................... 59**

**Tabla 3.2.6 Positives, negatives and undetectable according to original values of CT 59**

**Tabla 3.2.7 Viral load* reduction by group in logarithm scale ....................................... 59**

**Tabla 3.2.8 Viral load reduction – Model 1 – basic model ............................................. 59**

**Tabla 3.2.9 Viral load* reduction – Model 2 – one covariate........................................... 59**

**Tabla 3.2.10 Viral load reduction – Model 3 – final selected model ................................ 59**

**Tabla 3.2.11 Viral load reduction – Model 4 – final model................................................ 59**

**Tabla 4.1.1 Covid-19 WHO scale score progression I ...................................................... 59**

**Tabla 4.1.2 Covid-19 WHO scale progression I – extra visits.......................................... 64**

**Tabla 4.1.3 Covid-19 WHO scale score progression II ..................................................... 66**

**Tabla 4.1.4 Covid-19 WHO scale progression by group – extra visits ............................ 68**

**Tabla 4.1.5 WHO scale score by mixed model - Model 1 ................................................ 69**

**Tabla 4.1.6 WHO scale score by mixed model – Model 2 ................................................ 70**

**Tabla 4.1.7 WHO scale score by mixed model – Model 3 ................................................ 70**

**Tabla 4.1.8 WHO scale score by mixed model – Model 4 ................................................ 70**

**Tabla 4.2.1 Time to symptoms complete resolution ........................................................ 71**

**Tabla 4.2.2 Symptoms complete resolution: Hazard Ratio (HR) by Cox proportional hazard model 72**

**Tabla 4.2.3 Symptoms completely resolved within the first 28 days after infusion ...... 72**

**Tabla 4.3.1 Death per group.............................................................................................. 72**

**Tabla 4.3.2 Log-binomial regression model 1 – basic model.......................................... 72**

**Tabla 4.3.3 Log-binomial regression model 2 – one-covariate model............................ 73**

**Tabla 4.3.4 Log-binomial regression model 3 – final selected model ............................ 73**

**Tabla 4.3.5 Log-binomial regression model 4 – final model........................................... 73**

**Tabla 4.4.1 Change in D-dimer.......................................................................................... 74**

**Tabla 4.4.2 Change in D-dimer – Model 1 – basic model ................................................ 74**

**Tabla 4.4.3 Change in D-dimer – Model 2 – one covariate.............................................. 74**

**Tabla 4.4.4 Change in D-dimer – Model 3 – final selected model .................................. 75**

**Tabla 4.4.5 Change in D-dimer – Model 4 – final model.................................................. 75**


All rights reserved. No part of this material may be reproduced or copied in any form by any means (written, electronic, photocopying, recording or otherwise) without the prior consent of BioClever.

Tabla 4.4.6 Change in Ferritin by group ... 75

Tabla 4.4.7 Change in Ferritin – Model 1 – basic model ... 75

Tabla 4.4.8 Change in Ferritin – Model 2 – one covariate ... 75

Tabla 4.4.9 Change in Ferritin – Model 3 – final selected model ... 75

Tabla 4.4.10 Change in Ferritin – Model 4 – final model ... 75

Tabla 4.4.11 Change in IL-6 by group ... 76

Tabla 4.4.12 Change in IL-6 – Model 1 – basic model ... 76

Tabla 4.4.13 Change in IL-6 – Model 2 – one covariate ... 76

Tabla 4.4.14 Change in IL-6 – Model 3 – final selected model ... 76

Tabla 4.4.15 Change in IL-6 – Model 4 – final model ... 76

Tabla 4.4.16 Change in Lynphocites by group ... 76

Tabla 4.4.17 Change in Lynphocites – Model 1 – basic model ... 76

Tabla 4.4.18 Change in Lynphocites – Model 2 – one covariate ... 76

Tabla 4.4.19 Change in Lynphocites – Model 3 – final selected model ... 76

Tabla 4.4.20 Change in Lynphocites – Model 4 – final model ... 76

Tabla 4.4.21 Change in PCR by group ... 76

Tabla 4.4.22 Change in PCR – Model 1 – basic model ... 76

Tabla 4.4.23 Change in PCR – Model 2 – one covariate ... 76

Tabla 4.4.24 Change in PCR – Model 3 – final selected model ... 76

Tabla 4.4.25 Change in PCR – Model 4 – final model ... 76

Tabla 4.4.26 Change in Pre-albumin by group ... 76

Tabla 4.4.27 Change in Pre-albumin – Model 1 – basic model ... 76

Tabla 4.4.28 Change in Pre-albumin – Model 2 – one covariate ... 76

Tabla 4.4.29 Change in Pre-albumin – Model 3 – final selected model ... 76

Tabla 4.4.30 Change in Pre-albumin – Model 4 – final model ... 76

Tabla 5.1.1 General summary of adverse events – SAF ... 77

Tabla 5.1.2 Adverse events I (A) - SAF ... 78

Tabla 5.1.3 Adverse events II (A) - SAF ... 79

Tabla 5.1.4 Adverse events III (A) - SAF ... 80

Tabla 5.1.5 Adverse events I (B) - SAF ... 80

Tabla 5.1.6 Advere events II (B) - SAF ... 80


All rights reserved. No part of this material may be reproduced or copied in any form by any means (written, electronic, photocopying, recording or otherwise) without the prior consent of BioClever.

-82:1547 .7;3893 0-) ,#%"//*$ ,%%" $',' ($!  &,*./,&+'%+'



**Tabla 5.1.7 Advere events III (B) - SAF ........ 80**

**Tabla 6.1.1 Worst COVID-19 WHO scale score by group ........ 81**

**Tabla 6.1.2 Worst by proportional-odds - model 1 – basic model ........ 82**

**Tabla 6.1.3 Worst by proportional-odds - model 2 – one-covariate model ........ 82**

**Tabla 6.1.4 Worst by proportional-odds - model 3 – final selected model ........ 82**

**Tabla 6.1.5 Worst by proportional-odds - model 4 – final model ........ 83**

**Tabla 6.2.1 Distribution of gender, age and days of symptom onset by hospitalisation whitin groups83**

**Figure Index**

**Figure 1 Reduction of logarithm of Viral load by group ........ 56**

**Figure 2 Reduction of logarithm of Viral load by group ........ 59**

**Figure 3 Longitudinal display of patients COVID-19 WHO score progression by group .. 69**

**Figure 4 Time to symptoms complete resolution (Kaplan Meyer) ........ 71**

**Figure 5 Change in D-dimer by group ........ 74**

**Figure 6 Change in Ferritin by group ........ 75**

**Figure 7 Change in IL6 by group ........ 76**

**Figure 8 Change in Lynphocites by group ........ 76**

**Figure 9 Change in PCR by group ........ 76**

**Figure 10 Change in Pre-albumin by group ........ 76**

All rights reserved. No part of this material may be reproduced or copied in any form by any means (written, electronic, photocopying, recording or otherwise) without the prior consent of BioClever.

-82:1547 .7;3893 0-) ,#%"//*$ ,%%" $',' ($! &,*./,&+'%+'



## ABBREVATIONS AND DEFINITIONS

| | |
|---|---|
| AE | Adverse Event |
| AR | Adverse reaction |
| CI | Confidence interval |
| COVID-19 | Coronavirus disease 2019 |
| CRF | Case Report Form |
| FDA | Food and Drug Administration |
| IgA/IgM/IgG | Immunoglobulin A/ M/ G |
| ITT | Intetion-to-treat |
| MedDRA | Medical Dictionary for Regulatory Activities |
| MBT | Methylene blue treated |
| ml | milliliter |
| MT | Milddle turbinate |
| OR | Odds ratio |
| PCR | Polymerase chain reaction |
| PP | Per Protocol (set) |
| PT | Preferred Term |
| qPCR | Quantitative PCR |
| Q1, Q3 | Quartiles 1 and 3 |
| RR | Risk ratio |
| RT-PCR | Reverse transcriptase PCR |
| SAE | Serious Adverse Event |
| SAF | Safety analysis set |
| SAP | Statistical Analysis Plan |
| SAS® | Statistical Analysis System |
| SASR-CoV-2 | Severe acute respiratory syndrome coronavirus 2 |
| SD | Standard Deviation |

All rights reserved. No part of this material may be reproduced or copied in any form by any means (written, electronic, photocopying, recording or otherwise) without the prior consent of BioClever.

| | |
|---|---|
| SMT | Standard medical treatment |
| SOC | System Organ Class |
| SUSAR | Suspected Unexpected Serious Adverse Reaction |
| UAE | Unexpected adverse event |

All rights reserved. No part of this material may be reproduced or copied in any form by any means (written, electronic, photocopying, recording or otherwise) without the prior consent of BioClever.

# 1. Approvals (signatures)

The signatures on this page indicate review and approval of the statistical analysis plan for the final analysis and referenced Sections.

BioClever: Francini Ferreira

DocuSigned by:
Francini Ferreira
B4F3FCB55905453...

(Sig.) ______________________ Date: 25/8/2021

Principal investigator: Oriol Mitjá

DocuSigned by:
A1B4890500774E8...

(Sig.) ______________________ Date: 25/8/2021

Investigator: Andrea Alemany

DocuSigned by:
3EE9D9F3B95F48E...

(Sig.) ______________________ Date: 25/8/2021

All rights reserved. No part of this material may be reproduced or copied in any form by any means (written, electronic, photocopying, recording or otherwise) without the prior consent of BioClever.

# 2. Distribution List

| Name | Function / Role | Office/Sponsor |
|---|---|---|
| Oriol Mitjà | Principal Investigator | FLS |
| Andrea Alemany | Co-investigator | FLS |
| Marc Corbacho | Co-investigator | FLS |
| Bàrbara Baro | Co-investigator | ISGlobal |
| Pere Millat | Co-investigator | ISGlobal |
| Dan Ouchi | Statistician | IRSICaixa |

# 3. Document Version History

## 3.1 Changes to the protocol

According to the protocol, hospitalisation between groups, which is the primary endpoint, would be compared by the OR estimated by fitting a logistic regression. However, we have decided to analyse this outcome by the relative risk (RR) fitted by the log-binomial regression model.

Additionally, the analyses of 4 endpoints will be completed and published separatedly from this statistical analysis plan according to sponsor's decision.

## 3.2 Documentation history

| Version | Effective Date | Significant Changes |
|---|---|---|
| 0.2 | 25th, August 2021 | Changes accepted and section 9 completed. |
| | | |

All rights reserved. No part of this material may be reproduced or copied in any form by any means (written, electronic, photocopying, recording or otherwise) without the prior consent of BioClever.

-82:1547 .7;3893 0-) ,#%"//*$ ,%%" $',' ($! &,*./,&+'%+'



# 4. Introduction

## 4.1 Background and rationale

Coronavirus disease 2019 (COVID-19) is a respiratory tract infection caused by a newly emergent coronavirus, severe acute respiratory syndrome coronavirus 2 (SARS-CoV-2), that was first recognized in Wuhan, China, in December 2019. The emergence of COVID-19 has caused a large global outbreak and it is a major public health issue. As of 13 August 2020, data from the World Health Organization (WHO) have shown that more than 20 million confirmed cases have been identified in 216 countries, areas or territories (WHO Interim guidance 13 August 2020) (1).

Currently, there are no FDA-approved drugs for the treatment of COVID-19. Definitive clinical trial data is needed to find safe and effective treatments for COVID-19. Vaccine development is progressing at a rapid pace, but widespread vaccine availability is estimated to be at least six months away. There is an urgent need for effective interventions presently. Administration of SARS-CoV-2 neutralizing antibodies is the only form of immunization available in the absence of vaccines or humanized monoclonal antibodies.

Convalescent plasma therapy has been used to treat patients with infections using plasma collected from recently recovered individuals to transfer passive antibody immunity to those who have recently been infected or have yet to be exposed to the virus. Use of convalescent plasma has been studied in outbreaks of other respiratory infections, including the 2003 SARS-CoV-1 epidemic, the 2009-2010 H1N1 influenza virus pandemic, and the 2012 MERS-CoV epidemic (FDA recommendations) (2). Convalescent plasma from donors who have recovered from COVID-19 may contain antibodies to SARS-CoV-2 that might help suppress the virus and modify the inflammatory response. It has been postulated that neutralizing antibodies would prevent SARS-CoV-2 spike protein from attaching to the ACE2 receptor, inhibiting viral entry into the cell (Nguyen et al., 2020) (3).

## 4.2 Objectives

### 4.2.1 Primary objective

- To assess the therapeutic potential of early administration of convalescent MBT plasma in reducing the rate of hospitalization at day 28 in non-hospitalized mild or moderate COVID-19 patients.
- Assess the therapeutic potential of early administration of convalescent MBT plasma in reducing SARS-CoV-2 viral load at day 7, measured by quantitative RT-PCR (RT-qPCR) in non-hospitalised mild or moderate COVID-19 patients.

All rights reserved. No part of this material may be reproduced or copied in any form by any means (written, electronic, photocopying, recording or otherwise) without the prior consent of BioClever.

### 4.2.2 Secondary objectives

- Assess the therapeutic potential of early administration of convalescent MBT plasma in reducing WHO Clinical progression scale score in non-hospitalised mild or moderate COVID-19 patients.
- Assess the therapeutic potential of early administration of convalescent MBT plasma in reducing the severity of common COVID-19 symptoms, measured with the FLU-PRO© PLUS scale, in non-hospitalised mild or moderate COVID-19 patients.
- Assess the therapeutic potential of early administration of convalescent MBT plasma in reducing the duration of symptoms in non-hospitalised mild or moderate COVID-19 patients.
- Assess the therapeutic potential of early administration of convalescent MBT plasma in reducing the mortality at day 60 in non-hospitalised mild or moderate COVID-19 patients.
- Evaluate the safety and tolerability of convalescent MBT plasma in non-hospitalised mild or moderate COVID-19 patients.
- Assess the change from baseline to day 7 of ferritin, prealbumin, interleukin 6 (IL-6), D-dimer, C reactive protein (CRP), and leukocytes and lymphocytes counts in peripheral blood in non-hospitalised mild or moderate COVID-19 patients receiving convalescent MBT plasma.
- Assess the impact of infused plasma on neutralizing activity by quantifying the change from baseline to day 7 in neutralizing antibodies against SARS-CoV-2 in peripheral blood in non-hospitalised mild or moderate COVID-19 patients receiving convalescent MBT plasma.
- Assess the long-term impact of plasma infusion on humoral immune responses, by quantifying the change from baseline to day 60 in neutralizing antibodies against SARS-CoV-2 in peripheral blood in non-hospitalised mild or moderate COVID-19 patients receiving convalescent MBT plasma.
- Compare agreement and SARS-CoV-2 viral load in self-collected middle turbinate (MT) swab and self-collected saliva samples with nasopharyngeal swab samples collected by a healthcare worker.
- Assess the therapeutic potential of early administration of convalescent MBT plasma in reducing SARS-CoV-2 viral load at day 28, measured by quantitative RT-PCR (RT-qPCR) in non-hospitalised mild or moderate COVID-19 patients.

# 5. Clinical trial methodology

## 5.1 Study design

**Type of the trial:** This is a multi-site, randomized, controlled with placebo, double blind, parallel study.

This is a prospective, randomized (1:1), double blind, study of convalescent anti-SARS-CoV-2 MBT plasma plus standard medical treatment (SMT) versus placebo plus SMT in mild or moderate COVID-19 patients who are non-hospitalised. Subjects with a confirmed SARS-CoV-2 infection as determined by positive polymerase chain reaction (PCR) or validated antigen rapid diagnostic test from nasopharyngeal swabs will receive SMT plus one single infusion of 200-250 ml of convalescent plasma that has been pathogen-inactivated using methylene blue treatment (MBT) or 200-250 ml of placebo.

Study candidates voluntarily expressed their interest in participating in the study through the study website or will be offered to participate at the emergency (ER) and out-patient

All rights reserved. No part of this material may be reproduced or copied in any form by any means (written, electronic, photocopying, recording or otherwise) without the prior consent of BioClever.

departments (OPD) of the participating hospitals. Candidates registered on the website will be contacted by study physicians by phone to inform them about the study and check their suitability for the study. Suitable candidates will be scheduled an inclusion/baseline visit in which informed consent will be obtained (i.e., the informed consent will be signed), and their eligibility will be confirmed. Candidates identified through ER and OPD departments will undergo an inclusion/baseline visit, where the informed consent will be obtained and eligibility will be checked. A subgroup of eligible candidates from selected study sites will be offered participation in the substudy to assess the immune response and the methods of sampling.

Blood and nasopharyngeal samples will be obtained from all eligible candidates.

Eligible candidates will be randomized and administered an intravenous (IV) infusion at baseline (convalescent plasma or placebo). Both the investigator and the participant will be blinded to the study treatment.

Specifically, subjects randomized to combination convalescent anti-SARS-CoV-2 MBT plasma plus SMT will undergo an ABO compatibility test and will receive a single infusion of 200 to 250 ml of ABO-compatible convalescent plasma. Subjects randomized to placebo plus SMT will receive a single infusion of 200 to 250 ml of sterile saline solution 0.9%. Infusion will be administered at baseline, using standard procedures for administration of fresh frozen plasma. Small adults weighing less than 45 kg will receive one infusion of 5 ml of convalescent plasma or placebo per kilogram of body weight.

Participants will be trained on the completion of symptoms diary card and safety diary card.

The participants of the substudy will be drawn an extra tube of blood sample and will be trained on self-collection of middle turbinate (MT) swabs and saliva, and self-collected samples will be obtained.

The symptoms and safety diary card will be filled by the participants daily from baseline to day 14. On follow-up visits on days 3, 7, 14, and 28, all participants will be assessed for clinical and safety outcomes. These visits will be all by telephone except for the day 7 and day 28 visits that will be at home and at hospital, respectively, where additionally blood samples (only on day 7) and nasopharyngeal swabs will be collected.

At day 60 visit, all participants will be assessed by telephone for health-status outcome.

For the participants of the substudy, on day 7, an extra tube of blood sample will be obtained and they will be asked to self-collect MT swabs and saliva. And on day 60, an extra tube of blood sample will be obtained during an additional home or hospital visit.

## 5.2 Randomisation

Participants will be randomly allocated using the study eCRF in a ratio of 1:1 between the convalescent plasma arm (experimental) and the placebo arm (control).

Randomization will be centralized at the blood bank during the inclusion/baseline visit, after confirmation of eligibility. ABO compatibility test will be performed for the experimental arm.

## 5.3 Sample size

A sample size of 237 cases per arm would provide the trial with 80% power to detect 50% reduction in hospitalization rate at day 28 after starting the treatment, assuming an expected rate of hospitalization of 15%, allowing a 5% of loss to follow-up.

All rights reserved. No part of this material may be reproduced or copied in any form by any means (written, electronic, photocopying, recording or otherwise) without the prior consent of BioClever.

-82:1547 .7;3893 0-) ,#%"//*$ ,%%" $',' ($! &,*./,&+'%+'



Approximately 157 cases per arm are required to have 80% power to detect a minimal expected difference of 5 points in a scale of 156 points with 39 items, to assess symptoms' severity, at a significance level of α = 0.05, assuming a standard deviation of 15.

Approximately 150 cases per arm are required to have 80% power to detect a difference of 0.5 log10 in the mean reduction of SARS-CoV-2 viral load at a two-sided significance level of α = 0.05, assuming an expected overall standard deviation of 1.5. A 0.5 log10 copies/mL difference in reduction was chosen to represent the minimal threshold for a biologically relevant change for our analyses.

For the substudy for quantification of neutralizing antibodies against SARS-CoV-2 a total sample size of 135 was estimated considering parameters detailed in the rationale.

EARLY TRIAL TERMINATION: The trial stopped enrolment on the 31/05/2021 with enrollment of 76% of the target population. During April and May 2021, the incidence of COVID-19 in population over 50 years or older decreased drastically, partly due to the high vaccination rate of that population. The DSMB recommended the early trial termination, and the sponsor accepted the measure, considering it would be logistically impossible and ethically questionable to continue the trial.

# 6. General statistical considerations

## 6.1 Study population

- Intention-to-treat (ITT): This set will include all randomized patients. The treatment groups for this population will be defined according to the treatment assignment at randomisation.
- Per protocol (PP): It will include all the patients who satisfy:
  - Patients who fulfil all inclusion / exclusion criteria.
  - Correctly randomized patients
  - Without major or critical deviations
- Safety population (SAF): This set will include all patients who receive the investigational product (convalescent plasma or placebo). Analyses will be performed using the treatment received.

## 6.2 General issues

All data processing and analysis will be performed using SAS® version 9.4 or posterior, and the R statistical package, version 4.0.2 under a significance level of 0.05.

## 6.3 Presentation/Format of results

Mean, median and standard deviation will be printed out to one more decimal place than the recorded data and rounded appropriately. Minimum and maximum values will be presented using the same number of decimal places as the recorded data. The number of patients will be presented as a whole number.

All rights reserved. No part of this material may be reproduced or copied in any form by any means (written, electronic, photocopying, recording or otherwise) without the prior consent of BioClever.

Percentage values will be presented with one digit to the right of decimal point.

p-values will be presented to 4 decimal places (or as <0.0001 where appropriate).

## 6.4 Level of significance

Statistical comparisons will be made using two sided tests at the α=0.05 significance level unless specifically stated otherwise.

## 6.5 Handling of dropouts and missing data

No missing data imputation method will be used in this study and we will not analyse the presence of potential outliers.

# 7. Statistical analysis

## 7.1 Definitions of variables

<u>Baseline/ Day 3 - telephone/ Day 7 - home/ Day 14 - telephone/ Day 28 - hospital/ Day 60 – telephone/ Extra visits (at any time within follow-up period)</u>

- Yes: if the patient attended to the visit or if the family was contacted
- No: if the patient and his family did not attend to the visit

<u>Hospitalisation:</u>

Yes: if in any visit through day 28, the severity of Covid-19 according to WHO scale was ≥ 4

No: if in all visits through day 28, the severity of Covid-19 according to WHO scale was always < 4

<u>Death:</u>

Yes: if in any visit, the severity of Covid-19 according to WHO scale is 10 and death date was registered in the eCRD.

<u>Viral load difference:</u> Viral load at day 7 – baseline viral load. Viral load at day 28 – baseline viral load.

<u>Covid-19 severity based on WHO scale:</u>

Original scale:

0: Uninfected; no viral RNA detected

1: Asymptomatic; viral RNA detected

All rights reserved. No part of this material may be reproduced or copied in any form by any means (written, electronic, photocopying, recording or otherwise) without the prior consent of BioClever.

2: Symptomatic; independent

3: Symptomatic; assistance needed

4: Hospitalized; no oxygen therapy

5: Hospitalized; oxygen by mask or nasal prongs

6: Hospitalized; oxygen by NIV or high flow

7: Intubation and mechanical ventilation, $pO_2/FIO_2 \geq 150$ or $SpO_2/FI0_2 \geq 200$

8: Mechanical ventilation $pO_2/FIO_2 < 150$ ($SpO_2/FI0_2 < 200$) or vasopressors

9: Mechanical ventilation $pO_2/FIO_2 < 150$ and vasopressors, dialysis, or ECMO

10: Death

<u>New recategorization:</u>

No infection: when WHO scale is 0 or 1

Mild: when WHO scale is 2 or 3

Moderate: when WHO scale is 4 or 5

Severe: when WHO scale is between 6 and 9

Death: when WHO scale is 10

<u>Worst Covid-19 severity based on WHO scale:</u>

Each patient will receive their worst Covid-19 scale score registered considering the entire follow-up time.

<u>Days of symptom onset at baseline:</u>

Days= (baseline date – first symptom date)

<u>Days since positive test at baseline:</u>

Days= (baseline date – positive test date)

<u>Risk factors will be reorganized according to the following criteria defined by the searchers</u>

1) Smoker (current or former tobacco use): registered in eCRD
2) Obesity: variable registered in eCRD or if BMI ≥30
3) Cardiac disease: variable registered in eCRD or in other risk factors
4) Lung disease: if asthma, EPOC or any other lung disease was registered in other risk factors
5) Diabetes: variable registered in eCRD or in other risk factors
6) Chonic renal insufficiency: variable registered in eCRD or in other risk factors

All rights reserved. No part of this material may be reproduced or copied in any form by any means (written, electronic, photocopying, recording or otherwise) without the prior consent of BioClever.

7) Cancer (history of neoplastic disease): variable registered in eCRD or in other risk factors

8) Immune-compromised: variable registered in eCRD or in other risk factors

Number of comorbities: sum of the above comorbities, which can vary from 0 to 9.

Number of participants with no comorbidies.

Number of participants with 1, 2 or ≥3 comorbidities.

Presence of COVID-19 Symptom:

Symptoms will be cathegorized by coordinator-investigators from database (after recording of the symptoms by all subinvestigators during the trial in eCRD) in the following cathegories: (1) congestion or runny nose; (2) cough; (3) diarrhea; (4) fatigue; (5) fever or chills; (6) headache; (7) muscle or body aches; (8) nausea or vomiting; (9) new loss of taste or smell; (10) shortness of breath or difficulty breathing; (11) sore throat; (12) COVID-19 pneumonia; (13) COVID-19 other complications; (14) Other COVID-19 related; (15) AE.

*Cathegory 14 (other COVID-19 related) will not be included in symptoms analysis (time to resolution of symptoms).

*Cathegory 15 (AE) will be considered AE and analyzed with the rest of AE.

Time to resolution of symptoms*:

Duration (days) = (End date –baseline visit date)

*Only for symptoms of cathegories 1-13 (detailed above).

If the patient presents any symptom with no end date registered, the duration will be treated as censored at the last visit registered (most likely the date of day 60 or death date).

Symptoms classified also as Adverse Events:

Yes: if symptom I, registered as an adverse event, started before the infusion of the treatment (covalescent plasma or placebo), lasted until after the infusion day, and presented a grade change to worst.

Duration of AE and SAE:

Duration (days) = (End date – initial date)

AE related to treatment:

Those adverse events that were registered in eCRD as related to treatment by subinvestigators during the trial.

All rights reserved. No part of this material may be reproduced or copied in any form by any means (written, electronic, photocopying, recording or otherwise) without the prior consent of BioClever.

-82:1547 .7;36893 0-) ,#%"//*$ ,%%" $',' ($! &,*./,&+'%+'



AE related to treatment will be cathegorized by coordinator-investigators from database (after recording of the AE by all subinvestigators during the trial) in the following cathegories: (1) local reactions; (2) vasovagal syndrome; (3) fever or chills; (4) gastrointestinal symptoms; (5) mild allergic reactions; (6) severe allergic reactions; (7) thromboembolic events; (8) volume overload; (9) acute haemolytic transfusion reaction; (10) transfusion-related acute lung injury (TRALI); (11) other.

Unexpected AE: those AEs related to the treatment and not expected

Suspected unexpected serious adverse event reaction (SUSAR): those AE related to the treatment, not expected and severe for gravity.

## 7.2 Hypothesis and statistical methods

### 7.2.1 Univariate analysis. Descriptive statistics

Unless otherwise noted, all variables will be described according to their character as follows:

- **Categorical variables** will be summarized using frequencies and percentages.
- **Continuous variables** will be summarized using measures of central tendency and dispersion: mean, standard deviation, median, 25% and 75% percentiles (Q1 and Q3) and extreme values (minimum and maximum).

### 7.2.2 Bivariate analysis

When it is of interest to answer the study objectives, the relationship between variables will be evaluated:

- For two categorical variables, contingency tables with the frequency in each category and the percentage by columns will be presented. To evaluate the possible association Chi-square tests or Fisher's exact test will be performed and the resulting p-value will be presented.
- For a numerical variable with a categorical, descriptive statistics will be presented by groups. To evaluate the possible association, T-Test or Wilcoxon nonparametric tests will be performed, as well as their resulting p-value.
- Time to event data will be analysed and compared by the Kaplan-Meier estimator, and the median and the 25$^{th}$ and 75$^{th}$ percentiles will be presented along with their 95% CI, comparing the curves using Log Rank test.

### 7.2.3 Multivariate analysis

Event rate comparison between groups

All rights reserved. No part of this material may be reproduced or copied in any form by any means (written, electronic, photocopying, recording or otherwise) without the prior consent of BioClever.

-82:1547 .7;3893 0-) ,#%"//*$ ,%%" $',' ($! &,*./,&+'%+'



Hospitalisation and/or death rate between groups will be analysed using the risk ratio (RR) obtained by fitting a log-binomial regression model.

Longitudinal VL decay

Efficacy will be determined by comparing the mean reduction of the viral load from baseline to days 7 and 28, with the use of linear mixed-effects regression model. The mean reduction of viral load (in logarithmic$_{10}$ scale) will be compared by fitting linear mixed-effect models with the individual as a random effect in the intercept to adjutst for intra individual correlation. The viral load will be provided in logarithmic scale; if less than 15% of specimens presents undetectable viral load at a given follow-up assessment a value of 3 log10 copies per mL (i.e., lower limit of detection) will be assigned for the purpose of statistical analysis.

Prespecified analyses of the primary outcomes:

According to current available evidence on factors influencing the successful treatment of COVID-19, preespecified analyses of the primary outcomes will be performed in subgroups defined by baseline participant’s antibody serum status (IgG or IgM anti-SARS-CoV-2 positive and negative), duration of illness (≤3 days and >3 days), and according to the neutralization activity of the plasma received (ID50>250 and ID50≤250).

Mean change differences between groups

The secondary outcome regarding between-group differences in symptoms severity score will be assessed by means of linear regression. The mean reduction of WHO Clinical progression scale score will be compared by fitting proportional-odds mixed-effect models with the individual as random effect in the intercept to adjust for intra individual correlation.

The secondary outcome regarding change in inflammatory markers will be assessed by linear mixed-effects models.

Time to event analysis

The time to complete resolution of symptoms will be analysed using Kaplan-Meier survival functions and hazard ratios (HRs), calculated using a Cox proportional hazards regression model based on the assumptions of proportional risks. Kaplan-Meier estimates will be compared using the log-rank test.

Variable selection

All models will be adjusted with basal covariates chosen according to their clinical relevance or from the observed correlation with the study outcome, and variable selection procedure will be done using the AIC-based stepwise algorithm.

## 7.3 Demographic and baseline characteristics

Demographic data and baseline characteristics will be described following the methods that appear in section 7.2.

All rights reserved. No part of this material may be reproduced or copied in any form by any means (written, electronic, photocopying, recording or otherwise) without the prior consent of BioClever.

## 7.4 Efficacy analysis

### 7.4.1 Primary endpoints

a) *Hospitalization rate (i.e., who reach a score ≥4 in the WHO scale for clinical progression) [Time Frame: Up to 28 days after reception of investigational product]*

The frequency and percentage of hospitalization in each group will be described and hospitalization rate between groups will be assessed using the risk ratio (RR) obtained by fitting a log-binomial regression model. Three models will be adjusted:

1) Model 1: basic model, with only group of treatment (convalescent plasma/placebo) as independent variable.
2) Model 2: one-covariate model, adjusted by group of treatment plus one independent covariate: age, gender, BMI, days of symptom onset at baseline and risk factors (including inflammatory markers).
3) Model 3: full selected model, with only the statistically significant variables (pvalue<0.1) of models 2.
4) Model 4: final model, with only statistically significant variables of model 3. In case of all independent variables result statistically no significant, this model is equivalent to the model 1 and will not be presented once more.

*SUBGROUP ANALYSES: Subgroup analyses of the pimary endpoint of hospitalization rate will be performed according to:

a) Patients' baseline serum antibody status: Baseline serum antibody status: negative vs positive

b) Duracion of illness: ≤3 days vs >3 days from symptoms onset (to baseline visit)

c) Neutralization activity of the convalescent plasma received: ID50>250 vs ID50≤250.

b) *Reduction of SARS-CoV-2 viral load in nasopharyngeal swabs at day 7 and day 28 after start of treatment, as determined by RT-qPCR. [Time Frame: Up to 7 days and 28 days after reception of investigational product]*

Firstly, a description of mean viral load at baseline, day 7, day 28 and the differences of mean values (Day 7 - baseline) and (Day 28 - baseline) separated by group will be presented. Afterwards, the mean reduction of viral load within groups will be compared by mixed effects regression model:

- Dependent variable: the viral load differences (Day 7 - baseline) and (Day 28 - baseline), provided in $logarithmic_{10}$ scale
- Independent variables:
  - Group (Experimental (convalescent plasma) and control (placebo)) as fixed effect;
  - Individual as random effect.

All rights reserved. No part of this material may be reproduced or copied in any form by any means (written, electronic, photocopying, recording or otherwise) without the prior consent of BioClever.

- Covariates: age, gender, BMI, days of symptom onset at baseline and risk factors (including inflammatory markers).

The analysis will be also performed as describe above for the first endpoint: Model 1, adjusted for group of treatment only; Model 2 adjusted by group of treatment, and every covariate separately; Model 3 including all statistically significant variables of models 2; and Model 4 with the selected variables.

*SUBGROUP ANALYSES: Subgroup analyses of the primary endpoint of reduction of SARS-CoV-2 viral load in nasopharungeal swabs at day 7 and day 28 will be performed according to:

a) Patients' baseline serum antibody status: Baseline serum antibody status: negative vs positive

b) Duration of illness: ≤3 days vs >3 days from symptoms onset (to baseline visit)

c) Neutralization activity of the convalescent plasma: ID50>250 vs ID50≤250

### 7.4.2 Secondary endpoints

- *Change in COVID-19 WHO Clinical progression scale score [Time Frame: Up to 60 days after reception of investigational product]*

Firstly, we will present a description of the Covid-19 WHO score at baseline, day 3, 14 and 60 by telephone, day 7 at home, day 28 at the hospital and at the any extra day when contemplated. Afterwards, the progression within groups will be compared by cumulative logit proportional odds-model using PROC GLIMMIX in multinomial model with random effects, which will basically carry:

- Dependent variable: Covid-19 WHO scale score; an ordinal variable.
- Independent variables:
  - Group (Experimental (convalescent plasma) and control (placebo)) as fixed effect;
  - Day (baseline, day 3, 7, 14, 28, 60 and extra days) as random effect.

The analysis will be similarly performed as presented to assess the primary objectives: model 1 with only the basic variables (group and day in this respect); model 2 containing the basic variables plus age, gender, BMI, days of symptoms onset and every risk factor separately; model 3 with the basic variables and the statistically significant variables of Model 2 all together; and model 4 with the selected variables, which will include the basic variables and the statistically significant variables of model 3. It is worth noting in the case of no significant variables are found in models 2 (pvalue>0.1), model 3 and 4 will be dispensable seeing that model 1 would be the final model.

- *Change in COVID-19 symptoms severity score, assessed with the COVID-19 daily self-score tool (FLU-PRO© PLUS instrument) [Time Frame: Up to 14 days after reception of investigational product]*

All rights reserved. No part of this material may be reproduced or copied in any form by any means (written, electronic, photocopying, recording or otherwise) without the prior consent of BioClever.

-82:1547 .7;3893 0-) ,#%"//*$ ,%%"$',' ($! &,*./,&+'%+'



These analyses will not be included in this statistical analysis, according to sponsor's decision. Data regarding symptoms was collected during study follow-up until day 60 by subinvestigators and by daily self-registered symptom inventory until day 14 of follow-up using the FLU-PRO PLUS instrument. Coordinating investigators considered that the data recorded by subinvestigators was more reliable and consequently it was the chosen one for the statistical analysis. Only time to complete resolution of symptoms will be analyzed, not severity of symptoms.

- *Time to complete resolution of symptoms [Time Frame: Up to 28 days after reception of investigational product]*

Time to complete resolution of symptoms will be analysed as proposed in 7.2.3. Kaplan-Meier estimatior, the median, 25$^{th}$ ad 75$^{th}$ percentiles will be presented along with their confidence interval (95%), comparing the groups of treatment (covalescent plasma and placebo) by log-rank test. Moreover, hazard ratios (HRs) estimation will be also presented as well as its confidence interval (95%), adjusted by Cox proportional hazards regression models.

- *Death rate [Time Frame: Up to 60 days after reception of investigational product]*

Death rate will be analyzed similarly to hospitalization rate: by fitting log-binomial models to assess the death rate between groups by the risk ratio. Firstly, the frequency and percentage of death in each arm will be presented. Afterwards, the models will be adjusted as described in the primary objectives analysis.

1) Model 1: basic model, with only group of treatment (convalescent plasma/placebo) as independent variable.
2) Model 2: one-covariate model, adjusted by group of treatment plus one independent covariate: age, gender, days of symptom onset at baseline and risk factors.
3) Model 3: full selected model, with only the statistically significant variables (pvalue<0.1) of models 2.
4) Model 4: final model, with only statistically significant variables of model 3. In case of all independent variables result statistically no significant, this model is equivalent to the model 1 and will not be presented once more.

- *Proportion of patients with adverse events (AE) and proportion of grade ≥4 AE, based on the FDA Toxicity Grading Scale for Healthy Adult and Adolescent Volunteers scale [Time Frame: Up to 28 days after reception of investigational product]*

The frequency and percentage of general adverse events and adverse events with grade ≥4 in each arm will be described as detailed in section 7.5 Safety analysis.

All rights reserved. No part of this material may be reproduced or copied in any form by any means (written, electronic, photocopying, recording or otherwise) without the prior consent of BioClever.

- *Change in inflammatory prognostic markers (ferritin, prealbumin, interleukin 6 (IL-6), D-dimer, C reactive protein (CRP) and leukocyte and lymphocyte counts) [Time Frame: Baseline and day 7 after reception of investigational product]*

A description of inflammatory prognostic markers at baseline, day 7 and the difference (Day 7 – baseline) will be presented separated by group of treatment and will be compared by the tests described in section 7.2.2. Afterwards, the change in inflammatory prognostic marjers within groups will be compared by mixed effects regression model:

- Dependent variable: difference (Day 7 – baseline)
- Independent variables:
  - Group (Experimental (convalescent plasma) and control (placebo)) as fixed effect;
  - Individual as random effect.
- Covariates: age, gender, BMI, days of symptom onset at baseline and risk factors.

The analysis will be also performed as describe above for the first endpoint: Model 1, adjusted for group of treatment only; Model 2 adjusted by group of treatment, and every covariate separately; Model 3 including all statistically significant variables of models 2; and Model 4 with the selected variables.

- *Intergroup comparison of absolute neutralization titers against SARS-CoV-2 in plasma of a subgroup of participants [Time Frame: Baseline and day 7 after reception of investigational product]*

These analyses will not be included in this statistical analysis, according to sponsor's decision. Data regarding neutralization antibody titers against SARS-CoV-2 in plasma of participants at baseline, day 7 and 60 will be analyzed by an independent statistician. Results of this sub-study will be presented in a separate article.

- *Change in titers of neutralizing antibodies against SARS-CoV-2 in plasma of a subgroup of participants [Time Frame: Baseline and day 60 after reception of investigational product]*

These analyses will not be included in this statistical analysis, according to sponsor's decision. Data regarding neutralization antibody titers against SARS-CoV-2 in plasma of participants at baseline, day 7 and 60 will be analyzed by an independent statistician. Results of this sub-study will be presented in a separate article.

- *Agreement and SARS-CoV-2 viral loads of self-collected middle turbinate (MT) swab and self-collected saliva compared to nasopharyngeal swabs collected by a healthcare worker [Time Frame: Baseline and day 7 after reception of investigational product].*

All rights reserved. No part of this material may be reproduced or copied in any form by any means (written, electronic, photocopying, recording or otherwise) without the prior consent of BioClever.

These analyses will not be included in this statistical analysis, according to sponsor's decision. Data regarding viral load of self-collected MT swab and saliva will be analyzed by an independent statistician. Results of this sub-study will be presented in a separate article.

- *Reduction of SARS-CoV-2 viral load in nasopharyngeal swabs at day 28 after start of treatment, as determined by RT-qPCR. [Time Frame: Up to 28 days after reception of investigational product].*

The reduction of SARS-CoV-2 viral load at day 28 will be analyzed as described in section 7.4.1.

## 7.5 Safety analysis

The safety analysis will be performed by

- Number and percentage of patients who reported any adverse events (AE)
- Number and percentage of patients who reported adverse events with grade ≥4.
- Number and percentage of patients who reported serious adverse events (SAE)
- Number and percentage of patients who reported unexpected adverse events (UAE)
- Number and percentage of patients who reported severe adverse events
- Number and percentage of patients who reported Suspected Unexpected Serious Adverse Reaction (SUSAR)
- Number and percentage of patients who reported any AE related to COVID-19
- Number and percentage of patients who reported any AE related to the treatment

For each endpoint described above, comparations between groups will be made by the tests defined in section 7.2.2. Moreover, we will present a list of all adverse events and its relationship with the treatment (covalescent plasma/placebo), with the disease (Covid-19), causality, severity, duration and action taken and any other information registered in eCRD.

## 7.6 Other analysis

- *Worst COVID-19 WHO Clinical scale score [Time Frame: Up to 60 days after reception of investigational product]*

We will present a description of the Covid-19 WHO worst scale score registered during the entire follow-up. We will describe the number of participants in each group, and the percentages, of each score (1-10) in both groups. Subsequently, the worst COVID-19 WHO scale score between groups will be compared by odds-ratio (OR), estimated by fitting the cumulative logit proportional odds-model using PROC LOGISTIC in multinomial model, which will basically carry:

- Dependent variable: Worst Covid-19 WHO scale score; an ordinal variable.

All rights reserved. No part of this material may be reproduced or copied in any form by any means (written, electronic, photocopying, recording or otherwise) without the prior consent of BioClever.

-82:1547 .7;36893 0-) ,#%"//*$ ,%%" $',' ($! &,*./,&+'%+'



- Independent variables:
  - Group (Experimental (convalescent plasma) and control (placebo));
  - Covariates: age, gender, days of symptom onset and risk factors.

The analysis will be similarly performed as presented to assess the primary objectives: model 1 with only the basic variables; model 2 containing the basic variables plus covariates separately; model 3 with the basic variables and the statistically significant covariates of Model 2 all together; and model 4 with the selected variables, which will include the basic variables and the statistically significant variables of model 3. It is worth noting in the case of no significant variables are found in models 2 (pvalue>0.1), model 3 and 4 will be dispensable seeing that model 1 would be the final model.

All rights reserved. No part of this material may be reproduced or copied in any form by any means (written, electronic, photocopying, recording or otherwise) without the prior consent of BioClever.

# 8. Refences


WHO Interim guidance 13 August 2020. https://www.who.int/emergencies/diseases/novel-coronavirus-2019?gclid=CjwKCAjwydP5BRBREiwA-qrCGrDAeovgOiCboUh2dKJhptzKhJd9Tkha3iH1ZOiKAfe9nOkDggkXjxoC9rsQAvD_BwE

Recommendations for Investigational COVID-19 Convalescent Plasma. US FDA. Published May 1, 2020. Accessed July 27th, 2020. https://www.fda.gov/vaccines-blood-biologics/investigational-newdrug-ind-or-deviceexemption-ide-process-cber/recommendations-investigational-covid-19-convalescent-plasma

Nguyen AA, Habiballah SB, Platt CD, Geha RS, Chou JS, McDonald DR. Immunoglobulins in the treatment of COVID-19 infection: Proceed with caution!. *Clin Immunol*. 2020;216:108459. doi:10.1016/j.clim.2020.108459

Li L, Zhang W, Hu Y, et al. Effect of Convalescent Plasma Therapy on Time to Clinical Improvement in Patients With Severe and Life-threatening COVID-19: A Randomized Clinical Trial [published online ahead of print, 2020 Jun 3]. *JAMA*. 2020;e2010044. doi:10.1001/jama.2020.10044

Centers for Disease Control and Prevention CDC – Covid-19. Updated Feb. 22, 2021. Accessed August 25th, 2021. https://www.cdc.gov/coronavirus/2019-ncov/symptoms-testing/symptoms.html.


All rights reserved. No part of this material may be reproduced or copied in any form by any means (written, electronic, photocopying, recording or otherwise) without the prior consent of BioClever.

# 9. List of tables that will be presented in the report

## 9.1 Analysis sets

**Tabla 1.1.1 Analysis sets - Patients recruited**

| | **Total (n=xx)** |
|---|---|
| Number of patients in ITT: | xx (xx.x%) |
| Number of patients excluded from ITT by reason for exclusion: | xx (xx.x%) |
| Not randomized patient | xx (xx.x%) |
| | |
| Number of patients in PP | xx (xx.x%) |
| Number of patients excluded from PP by reason for exclusion*: | |
| Individuals whose age <50 | xx (xx.x%) |
| In women of childbearing potential, not negative pregnancy test at inclusion/baseline visit | xx (xx.x%) |
| Not confirmed SARS-CoV-2 infection | xx (xx.x%) |
| Not symptomatic with mild or moderate COVID-19 with symptoms onset date < 7 days prior to inclusion/baseline visit | xx (xx.x%) |
| Not willing to comply with the requirements of the protocol and available for follow-up for the planned duration of the study | xx (xx.x%) |
| Has not understood the information provided and capable of giving informed consent | xx (xx.x%) |
| Female, pregnant or breastfeeding, or planning a pregnancy during the study | xx (xx.x%) |
| Severe or critical COVID-19 | xx (xx.x%) |
| Current hospital admission for any cause | xx (xx.x%) |
| History of previous confirmed SARS-CoV-2 infection | xx (xx.x%) |
| History of significantly abnormal liver function (Child Pugh C.) | xx (xx.x%) |
| History of chronic kidney disease (CKD) ≥ stage 4, or need of dialysis treatment | xx (xx.x%) |
| Any pre-existing condition that increases risk of thrombosis | xx (xx.x%) |
| History of allergic reactions to blood or plasma products or methylene blue | xx (xx.x%) |
| Known IgA deficiency with anti-IgA antibodies | xx (xx.x%) |

All rights reserved. No part of this material may be reproduced or copied in any form by any means (written, electronic, photocopying, recording or otherwise) without the prior consent of BioClever.

| | Total (n=xx) |
|---|---|
| Medical conditions for which 250ml of intravenous fluid is considered dangerous (i.e., decompensated heart failure or renal failure with fluid overload) | xx (xx.x%) |
| Inability to consent and/or comply with study protocol, in the opinion of the investigator | xx (xx.x%) |
| Currently participating or planning to participate in any interventional study for the treatment of COVID-19 or SARS-CoV-2 infection until day 60 | xx (xx.x%) |
| Potocol deviation | xx (xx.x%) |
| Not correctly randomized | xx (xx.x%) |
| | |
| Number of patients in SAF | xx (xx.x%) |
| Number of patients excluded from SAF by reason for exclusion: | xx (xx.x%) |
| Patients who did not receive investigational product (convalescent plasma or placebo) | xx (xx.x%) |

*Patiens may present more than one reason of exclusion

## Tabla 1.1.2 Visits - ITT

| | | A (n=xx) | B (n=xx) |
|---|---|---|---|
| Baseline | | | |
| Total no-missing | n | xx | xx |
| Yes | n (%) | xx (xx.x%) | xx (xx.x%) |
| No | n (%) | xx (xx.x%) | xx (xx.x%) |
| Missing | n | xx | xx |
| | | | |
| Day 3 - telephone | | | |
| Total no-missing | n | xx | xx |
| Yes | n (%) | xx (xx.x%) | xx (xx.x%) |
| No | n (%) | xx (xx.x%) | xx (xx.x%) |

All rights reserved. No part of this material may be reproduced or copied in any form by any means (written, electronic, photocopying, recording or otherwise) without the prior consent of BioClever.

| | | **A (n=xx)** | **B (n=xx)** |
|---|---|---|---|
| Missing | n | xx | Xx |
| | | | |
| Day 7 - home | | | |
| Total no-missing | n | xx | xx |
| Yes | n (%) | xx (xx.x%) | xx (xx.x%) |
| No | n (%) | xx (xx.x%) | xx (xx.x%) |
| Missing | n | xx | Xx |
| | | | |
| Day 14 - telephone | | | |
| Total no-missing | n | xx | xx |
| Yes | n (%) | xx (xx.x%) | xx (xx.x%) |
| No | n (%) | xx (xx.x%) | xx (xx.x%) |
| Missing | n | xx | Xx |
| | | | |
| Day 28 - hospital | | | |
| Total no-missing | n | xx | Xx |
| Yes | n (%) | xx (xx.x%) | xx (xx.x%) |
| No | n (%) | xx (xx.x%) | xx (xx.x%) |
| Missing | n | xx | xx |
| | | | |
| Day 60 - telephone | | | |
| Total no-missing | n | xx | Xx |
| Yes | n (%) | xx (xx.x%) | xx (xx.x%) |
| No | n (%) | xx (xx.x%) | xx (xx.x%) |
| Missing | n | xx | xx |

All rights reserved. No part of this material may be reproduced or copied in any form by any means (written, electronic, photocopying, recording or otherwise) without the prior consent of BioClever.

## Tabla 1.1.3 Extra visits - ITT

| | | A (n=xx) | B (n=xx) |
|---|---|---|---|
| Visit Extra 1 | | | |
| Total no-missing | n | xx | xx |
| Yes | n (%) | xx (xx.x%) | xx (xx.x%) |
| No | n (%) | xx (xx.x%) | xx (xx.x%) |
| Missing | n | xx | xx |
| Visit Extra 2 | | | |
| Total no-missing | n | xx | xx |
| Yes | n (%) | xx (xx.x%) | xx (xx.x%) |
| No | n (%) | xx (xx.x%) | xx (xx.x%) |
| Missing | n | xx | xx |
| … | | | |
| Visit Extra 10 | | | |
| Total no-missing | n | xx | xx |
| Yes | n (%) | xx (xx.x%) | xx (xx.x%) |
| No | n (%) | xx (xx.x%) | xx (xx.x%) |
| Missing | n | xx | xx |

All rights reserved. No part of this material may be reproduced or copied in any form by any means (written, electronic, photocopying, recording or otherwise) without the prior consent of BioClever.

## 9.2 Descriptive analysis

### 9.2.1 Descriptive analysis of participant baseline characteristics - ITT

**Tabla 2.1.1 Demographic data**

| | | **A**<br>**(n=xx)** | **B**<br>**(n=xx)** |
|---|---|---|---|
| Gender | | | |
| Total no-missing | n | xx | xx |
| Male | n (%) | xx (xx.x%) | xx (xx.x%) |
| Female | n (%) | xx (xx.x%) | xx (xx.x%) |
| Missing | n | xx | xx |
| | pvalue | x.xxxx | |
| Age (years) | n | xx | xx |
| | Mean (SD) | xx.x (xx.x) | xx.x (xx.x) |
| | Median (Q1, Q3) | xx.x (xx.x, xx.x) | xx.x (xx.x, xx.x) |
| | Min, Max | xx, xx | xx, xx |
| | Missing | xx | xx |
| | pvalue | x.xxxx | |
| Weight (kg) | n | xx | xx |
| | Mean (SD) | xx.x (xx.x) | xx.x (xx.x) |
| | Median (Q1, Q3) | xx.x (xx.x, xx.x) | xx.x (xx.x, xx.x) |
| | Min, Max | xx, xx | xx, xx |
| | Missing | xx | xx |
| | pvalue | x.xxxx | |
| Height (cm) | n | xx | xx |
| | Mean (SD) | xx.x (xx.x) | xx.x (xx.x) |

All rights reserved. No part of this material may be reproduced or copied in any form by any means (written, electronic, photocopying, recording or otherwise) without the prior consent of BioClever.

| | | A (n=xx) | B (n=xx) |
|---|---|---|---|
| | Median (Q1, Q3) | xx.x (xx.x, xx.x) | xx.x (xx.x, xx.x) |
| | Min, Max | xx, xx | xx, xx |
| | Missing | xx | xx |
| | pvalue | x.xxxx | |
| BMI | n | xx | xx |
| | Mean (SD) | xx.x (xx.x) | xx.x (xx.x) |
| | Median (Q1, Q3) | xx.x (xx.x, xx.x) | xx.x (xx.x, xx.x) |
| | Min, Max | xx, xx | xx, xx |
| | Missing | xx | xx |
| | pvalue | x.xxxx | |

**Tabla 2.1.2 Physical characteristics**

| | | A (n=xx) | B (n=xx) |
|---|---|---|---|
| General state | | | |
| Total no-missing | n | xx | xx |
| Normal | n (%) | xx (xx.x%) | xx (xx.x%) |
| Abnormal | n (%) | xx (xx.x%) | xx (xx.x%) |
| Missing | n | xx | xx |
| | pvalue | x.xxxx | |
| Skin | | | |
| Total no-missing | n | xx | xx |
| Normal | n (%) | xx (xx.x%) | xx (xx.x%) |
| Abnormal | n (%) | xx (xx.x%) | xx (xx.x%) |

All rights reserved. No part of this material may be reproduced or copied in any form by any means (written, electronic, photocopying, recording or otherwise) without the prior consent of BioClever.

| | | A (n=xx) | B (n=xx) |
|---|---|---|---|
| Missing | n | xx | xx |
| | pvalue | x.xxxx | |
| Cardiac auscultation | | | |
| Total no-missing | n | xx | xx |
| Normal | n (%) | xx (xx.x%) | xx (xx.x%) |
| Abnormal | n (%) | xx (xx.x%) | xx (xx.x%) |
| Missing | n | xx | xx |
| | pvalue | x.xxxx | |
| Respiratory auscultation | | | |
| Total no-missing | n | xx | xx |
| Normal | n (%) | xx (xx.x%) | xx (xx.x%) |
| Abnormal | n (%) | xx (xx.x%) | xx (xx.x%) |
| Missing | n | xx | xx |
| | pvalue | x.xxxx | |
| Abdominal examination | | | |
| Total no-missing | n | xx | xx |
| Normal | n (%) | xx (xx.x%) | xx (xx.x%) |
| Abnormal | n (%) | xx (xx.x%) | xx (xx.x%) |
| Missing | n | xx | xx |
| | pvalue | x.xxxx | |
| Extremities and peripheralpulses | | | |
| Total no-missing | n | xx | xx |

All rights reserved. No part of this material may be reproduced or copied in any form by any means (written, electronic, photocopying, recording or otherwise) without the prior consent of BioClever.

| | | A (n=xx) | B (n=xx) |
|---|---|---|---|
| Normal | n (%) | xx (xx.x%) | xx (xx.x%) |
| Abnormal | n (%) | xx (xx.x%) | xx (xx.x%) |
| Missing | n | xx | xx |
| | pvalue | x.xxxx | |
| The participant presents signs of decompensated heart failure, negrotic syndrome, hemodynamic instability, or ascitic-edematous decompensation? | | | |
| Total no-missing | n | xx | xx |
| Yes | n (%) | xx (xx.x%) | xx (xx.x%) |
| No | n (%) | xx (xx.x%) | xx (xx.x%) |
| Missing | n | xx | xx |
| | pvalue | x.xxxx | |

## Tabla 2.1.3 Vital signs

| | | A (n=xx) | B (n=xx) |
|---|---|---|---|
| Temperature | n | xx | xx |
| | Mean (SD) | xx.x (xx.x) | xx.x (xx.x) |
| | Median (Q1, Q3) | xx.x (xx.x, xx.x) | xx.x (xx.x, xx.x) |
| | Min, Max | xx, xx | xx, xx |
| | Missing | xx | xx |
| | pvalue | x.xxxx | |
| $SpO_2$ | n | xx | xx |

All rights reserved. No part of this material may be reproduced or copied in any form by any means (written, electronic, photocopying, recording or otherwise) without the prior consent of BioClever.

- 82 : 1 47 . 7 ; 3 893 0 - ) , #%" / / * $ , %%" $ ' , ' ( $ ! & , * . / , & + ' %+ '



| | | **A**<br>**(n=xx)** | **B**<br>**(n=xx)** |
|---|---|---|---|
| | Mean (SD) | xx.x (xx.x) | xx.x (xx.x) |
| | Median (Q1, Q3) | xx.x (xx.x, xx.x) | xx.x (xx.x, xx.x) |
| | Min, Max | xx, xx | xx, xx |
| | Missing | xx | xx |
| | pvalue | x.xxxx | |
| Respiratory frequency | n | xx | xx |
| | Mean (SD) | xx.x (xx.x) | xx.x (xx.x) |
| | Median (Q1, Q3) | xx.x (xx.x, xx.x) | xx.x (xx.x, xx.x) |
| | Min, Max | xx, xx | xx, xx |
| | Missing | xx | xx |
| | pvalue | x.xxxx | |
| Cardiac frequency | | | |
| Total no-missing | n | xx | xx |
| Male | n (%) | xx (xx.x%) | xx (xx.x%) |
| Female | n (%) | xx (xx.x%) | xx (xx.x%) |
| Missing | n | xx | xx |
| | pvalue | x.xxxx | |

**Tabla 2.1.4 Risk factors**

| | | **A**<br>**(n=xx)** | **B**<br>**(n=xx)** |
|---|---|---|---|
| Number of comorbities - numerical | n | xx | xx |
| | Mean (SD) | xx.x (xx.x) | xx.x (xx.x) |
| | Median (Q1, Q3) | xx.x (xx.x, xx.x) | xx.x (xx.x, xx.x) |

All rights reserved. No part of this material may be reproduced or copied in any form by any means (written, electronic, photocopying, recording or otherwise) without the prior consent of BioClever.

| | | A (n=xx) | B (n=xx) |
|---|---|---|---|
| | Min, Max | xx, xx | xx, xx |
| | Missing | xx | xx |
| | pvalue | x.xxxx | |
| Number of comorbidities - categorical | | | |
| Total no-missing | n | xx | xx |
| 0 | n (%) | xx (xx.x%) | xx (xx.x%) |
| 1 | n (%) | xx (xx.x%) | xx (xx.x%) |
| 2 | n (%) | xx (xx.x%) | xx (xx.x%) |
| 3 or more | n (%) | xx (xx.x%) | xx (xx.x%) |
| Missing | n | xx | xx |
| | pvalue | x.xxxx | |
| Smoke habits | | | |
| Total no-missing | n | xx | xx |
| Not smoker | n (%) | xx (xx.x%) | xx (xx.x%) |
| Smoker | n (%) | xx (xx.x%) | xx (xx.x%) |
| Ex-smoker | n (%) | xx (xx.x%) | xx (xx.x%) |
| Missing | n | xx | xx |
| | pvalue | x.xxxx | |
| Number of cigarettes (smokers and ex-smokers) | n | xx | xx |
| | Mean (SD) | xx.x (xx.x) | xx.x (xx.x) |
| | Median (Q1, Q3) | xx.x (xx.x, xx.x) | xx.x (xx.x, xx.x) |
| | Min, Max | xx, xx | xx, xx |
| | Missing | xx | xx |
| | pvalue | x.xxxx | |

All rights reserved. No part of this material may be reproduced or copied in any form by any means (written, electronic, photocopying, recording or otherwise) without the prior consent of BioClever.

-82:1547 .7;36893 0-) ,#%"//*$ ,%%" $',' ($! &,*./,&+'%+'



| | | A (n=xx) | B (n=xx) |
|---|---|---|---|
| Obesity | | | |
| Total no-missing | n | xx | xx |
| Yes | n (%) | xx (xx.x%) | xx (xx.x%) |
| No | n (%) | xx (xx.x%) | xx (xx.x%) |
| Missing | n | xx | xx |
| | pvalue | x.xxxx | |
| Cardiac disease | | | |
| Total no-missing | n | xx | xx |
| Yes | n (%) | xx (xx.x%) | xx (xx.x%) |
| No | n (%) | xx (xx.x%) | xx (xx.x%) |
| Missing | n | xx | xx |
| | pvalue | x.xxxx | |
| Cardiac disease - specification | | | |
| Total no-missing | n | xx | xx |
| Type 1 | n (%) | xx (xx.x%) | xx (xx.x%) |
| .. | n (%) | xx (xx.x%) | xx (xx.x%) |
| Missing | n | xx | xx |
| Lung disease | | | |
| Total no-missing | n | xx | xx |
| Yes | n (%) | xx (xx.x%) | xx (xx.x%) |
| No | n (%) | xx (xx.x%) | xx (xx.x%) |
| Missing | n | xx | xx |
| | pvalue | x.xxxx | |

All rights reserved. No part of this material may be reproduced or copied in any form by any means (written, electronic, photocopying, recording or otherwise) without the prior consent of BioClever.

| | | **A** (n=xx) | **B** (n=xx) |
|---|---|---|---|
| Lung disease - specification | | | |
| Total no-missing | n | xx | xx |
| Type 1 | n (%) | xx (xx.x%) | xx (xx.x%) |
| .. | n (%) | xx (xx.x%) | xx (xx.x%) |
| Missing | n | xx | xx |
| Diabetes Mellitus | | | |
| Total no-missing | n | xx | xx |
| Yes | n (%) | xx (xx.x%) | xx (xx.x%) |
| No | n (%) | xx (xx.x%) | xx (xx.x%) |
| Missing | n | xx | xx |
| | pvalue | x.xxxx | |
| Diabetes - specification | | | |
| Total no-missing | n | xx | xx |
| Type 1 | n (%) | xx (xx.x%) | xx (xx.x%) |
| .. | n (%) | xx (xx.x%) | xx (xx.x%) |
| Missing | n | xx | xx |
| Chronic renal insuficiency | | | |
| Total no-missing | n | xx | xx |
| Yes | n (%) | xx (xx.x%) | xx (xx.x%) |
| No | n (%) | xx (xx.x%) | xx (xx.x%) |
| Missing | n | xx | xx |
| | pvalue | x.xxxx | |
| Chronic renal insuficiency - specification | | | |
| Total no-missing | n | xx | xx |

All rights reserved. No part of this material may be reproduced or copied in any form by any means (written, electronic, photocopying, recording or otherwise) without the prior consent of BioClever.

- 82 : 1؟47 . 7 ; 3؟893 0 - ) , #%" / / * $ , %%" $ ' , ' ($! &, * . / , &+ ' %+ '



| | | A (n=xx) | B (n=xx) |
|---|---|---|---|
| Type 1 | n (%) | xx (xx.x%) | xx (xx.x%) |
| .. | n (%) | xx (xx.x%) | xx (xx.x%) |
| Missing | n | xx | xx |
| | | | |
| Neurological disease | | | |
| Total no-missing | n | xx | xx |
| Yes | n (%) | xx (xx.x%) | xx (xx.x%) |
| No | n (%) | xx (xx.x%) | xx (xx.x%) |
| Missing | n | xx | xx |
| | pvalue | x.xxxx | |
| Neurological disease - specification | | | |
| Total no-missing | n | xx | xx |
| Type 1 | n (%) | xx (xx.x%) | xx (xx.x%) |
| .. | n (%) | xx (xx.x%) | xx (xx.x%) |
| Missing | n | xx | xx |
| | | | |
| Past oncologic illness | | | |
| Total no-missing | n | xx | xx |
| Yes | n (%) | xx (xx.x%) | xx (xx.x%) |
| No | n (%) | xx (xx.x%) | xx (xx.x%) |
| Missing | n | xx | xx |
| | pvalue | x.xxxx | |
| Past oncologic illness - specification | | | |
| Total no-missing | n | xx | xx |
| Type 1 | n (%) | xx (xx.x%) | xx (xx.x%) |
| .. | n (%) | xx (xx.x%) | xx (xx.x%) |

All rights reserved. No part of this material may be reproduced or copied in any form by any means (written, electronic, photocopying, recording or otherwise) without the prior consent of BioClever.

| | | A<br>(n=xx) | B<br>(n=xx) |
|---|---|---|---|
| Missing | n | xx | xx |
| Liver disease | | | |
| Total no-missing | n | xx | xx |
| Yes | n (%) | xx (xx.x%) | xx (xx.x%) |
| No | n (%) | xx (xx.x%) | xx (xx.x%) |
| Missing | n | xx | xx |
| | pvalue | x.xxxx | |
| Liver disease - specification | | | |
| Total no-missing | n | xx | xx |
| Type 1 | n (%) | xx (xx.x%) | xx (xx.x%) |
| .. | n (%) | xx (xx.x%) | xx (xx.x%) |
| Missing | n | xx | xx |
| Immunodeficiency | | | |
| Total no-missing | n | xx | xx |
| Yes | n (%) | xx (xx.x%) | xx (xx.x%) |
| No | n (%) | xx (xx.x%) | xx (xx.x%) |
| Missing | n | xx | xx |
| | pvalue | x.xxxx | |
| Immunodeficiency - specification | | | |
| Total no-missing | n | xx | xx |
| Type 1 | n (%) | xx (xx.x%) | xx (xx.x%) |
| .. | n (%) | xx (xx.x%) | xx (xx.x%) |
| Missing | n | xx | xx |

[1]Subjects can present more than one risk factor.

All rights reserved. No part of this material may be reproduced or copied in any form by any means (written, electronic, photocopying, recording or otherwise) without the prior consent of BioClever.

## Tabla 2.1.5 Women characteristics regarding sterilization, menstruation and pregnancy test result

| | | A (women) (n=xx) | B (women) (n=xx) |
|---|---|---|---|
| History of permanent sterilization surgery | | | |
| Total no-missing | n | xx | xx |
| Yes | n (%) | xx (xx.x%) | xx (xx.x%) |
| No | n (%) | xx (xx.x%) | xx (xx.x%) |
| Missing | n | xx | xx |
| | pvalue | x.xxxx | |
| Menstruation during the last year | | | |
| Total no-missing | n | xx | xx |
| Yes | n (%) | xx (xx.x%) | xx (xx.x%) |
| No | n (%) | xx (xx.x%) | xx (xx.x%) |
| Missing | n | xx | xx |
| | pvalue | x.xxxx | |
| Pregnancy test result | | | |
| Total no-missing | n | xx | xx |
| Negative | n (%) | xx (xx.x%) | xx (xx.x%) |
| Positive | n (%) | xx (xx.x%) | xx (xx.x%) |
| Missing | n | xx | xx |
| | pvalue | x.xxxx | |

All rights reserved. No part of this material may be reproduced or copied in any form by any means (written, electronic, photocopying, recording or otherwise) without the prior consent of BioClever.

### Tabla 2.1.6 Covid-19 severity at baseline

| | | A (n=xx) | B (n=xx) |
|---|---|---|---|
| Covid-19 severity | | | |
| Total no-missing | n | xx | xx |
| Mild | n (%) | xx (xx.x%) | xx (xx.x%) |
| Moderate | n (%) | xx (xx.x%) | xx (xx.x%) |
| Severe | n (%) | xx (xx.x%) | xx (xx.x%) |
| Critical | n (%) | xx (xx.x%) | xx (xx.x%) |
| Missing | n | xx | xx |
| | pvalue | x.xxxx | |
| Covid-19 severity based on WHO scale | | | |
| Total no-missing | n | xx | xx |
| No infection (0-1) | n (%) | xx (xx.x%) | xx (xx.x%) |
| Mild (2-3) | n (%) | xx (xx.x%) | xx (xx.x%) |
| Moderate (4-5) | n (%) | xx (xx.x%) | xx (xx.x%) |
| Severe (from 6-9) | n (%) | xx (xx.x%) | xx (xx.x%) |
| Dead (10) | n (%) | xx (xx.x%) | xx (xx.x%) |
| Missing | n | xx | xx |
| | pvalue | x.xxxx | |


All rights reserved. No part of this material may be reproduced or copied in any form by any means (written, electronic, photocopying, recording or otherwise) without the prior consent of BioClever.

**Tabla 2.1.7 Symptoms onset at baseline**

| | | A (n=xx) | B (n=xx) |
|---|---|---|---|
| Days of symptom onset at baseline visit (days) | n | xx | xx |
| | Mean (SD) | xx.x (xx.x) | xx.x (xx.x) |
| | Median (Q1,Q3) | xx (xx, xx) | xx (xx, xx) |
| | (Min,Max) | (xx, xx) | (xx, xx) |
| | Missing | xx | xx |
| | pvalue | x.xxxx | |
| Days since positive test at baseline visit (days) | n | xx | xx |
| | Mean (SD) | xx.x (xx.x) | xx.x (xx.x) |
| | Median (Q1,Q3) | xx (xx, xx) | xx (xx, xx) |
| | (Min,Max) | (xx, xx) | (xx, xx) |
| | Missing | xx | xx |
| | pvalue | x.xxxx | |

## 9.2.2 Convalescent plasma infused volume

**Tabla 2.2.1 Treatment infused volume received**

| | | Total (treatment) (n=xx) |
|---|---|---|
| Infused volume (bag's volume) | n | xx |
| | Mean (SD) | xx.x (xx.x) |
| | Median (Q1,Q3) | xx (xx, xx) |
| | (Min,Max) | (xx, xx) |
| | Missing | xx |

All rights reserved. No part of this material may be reproduced or copied in any form by any means (written, electronic, photocopying, recording or otherwise) without the prior consent of BioClever.

### 9.2.3 Symptoms before treatment infusion

**Tabla 2.3.1 Frequency of Covid-19 symptoms registered by the medical staff**

| | | A (n=xx) | B (n=xx) |
|---|---|---|---|
| Symptom 1 | n (%) | xx (xx.x%) | xx (xx.x%) |
| Symptom 2 | n (%) | xx (xx.x%) | xx (xx.x%) |
| … | | | |

All rights reserved. No part of this material may be reproduced or copied in any form by any means (written, electronic, photocopying, recording or otherwise) without the prior consent of BioClever.

## Tabla 2.3.2 Covid-19 symptoms registered by the medical staff – I – (A)

| | | | | Seriousness | | Grade | | | | | Related to COVID-19 | |
|---|---|---|---|---|---|---|---|---|---|---|---|---|
| | **Nº pat. (%)** | **Nº AE** | **Duration (days)*** | **Serious** | **Not Serious** | **Mild** | **Moderate** | **Severe** | **Vital risk** | **Death** | **Not Related** | **Related** |
| **Overall** | xx (xx.x) | xx | x.x (x.x) | xx | xx | xx | xx | xx | xx | xx | xx | xx |
| **SOC 1** | xx (xx.x) | xx | x.x (x.x) | xx | xx | xx | xx | xx | xx | xx | xx | xx |
| PT 1 | xx (xx.x) | xx | x.x (x.x) | xx | xx | xx | xx | xx | xx | xx | xx | xx |
| PT 2 | xx (xx.x) | xx | x.x (x.x) | xx | xx | xx | xx | xx | xx | xx | xx | xx |
| … | | | | | | | | | | | | |
| **SOC 2** | xx (xx.x) | xx | x.x (x.x) | xx | xx | xx | xx | xx | xx | xx | xx | xx |
| PT 1 | xx (xx.x) | xx | x.x (x.x) | xx | xx | xx | xx | xx | xx | xx | xx | xx |
| PT 2 | xx (xx.x) | xx | x.x (x.x) | xx | xx | xx | xx | xx | xx | xx | xx | xx |
| … | | | | | | | | | | | | |
| … | | | | | | | | | | | | |

*SOC and PT: coding with the MedDRA dictionary

All rights reserved. No part of this material may be reproduced or copied in any form by any means (written, electronic, photocopying, recording or otherwise) without the prior consent of BioClever.

### Tabla 2.3.3 Covid-19 symptoms registered by the medical staff – II – (A)

| | | | Expected | | Necessity for medication | | Resolution | | | | Active | |
|---|---|---|---|---|---|---|---|---|---|---|---|---|
| | Nº pat. (%) | Nº AE | Yes | No | Yes | No | Resolved | Stabilized | Basal condition | Unresolved | Yes | No |
| **Overall** | xx (xx.x) | xx | xx | xx | xx | xx | xx | xx | xx | xx | xx | xx |
| **SOC 1** | xx (xx.x) | xx | xx | xx | xx | xx | xx | xx | xx | xx | xx | xx |
| PT 1 | xx (xx.x) | xx | xx | xx | xx | xx | xx | xx | xx | xx | xx | xx |
| PT 2 | xx (xx.x) | xx | xx | xx | xx | xx | xx | xx | xx | xx | xx | xx |
| … | | | | | | | | | | | | |
| **SOC 2** | xx (xx.x) | xx | xx | xx | xx | xx | xx | xx | xx | xx | xx | xx |
| PT 1 | xx (xx.x) | xx | xx | xx | xx | xx | xx | xx | xx | xx | xx | xx |
| PT 2 | xx (xx.x) | xx | xx | xx | xx | xx | xx | xx | xx | xx | xx | xx |
| … | | | | | | | | | | | | |
| … | | | | | | | | | | | | |

All rights reserved. No part of this material may be reproduced or copied in any form by any means (written, electronic, photocopying, recording or otherwise) without the prior consent of BioClever.

### Tabla 2.3.4 Covid-19 symptoms registered by the medical staff – III – (A)

| | | | Action taken | | | Grade change | |
|---|---|---|---|---|---|---|---|
| | **Nº pat. (%)** | **Nº AE** | **None** | **Temporal interruption** | **Permanent interruption** | **Yes** | **no** |
| **Overall** | xx (xx.x) | xx | xx | xx | xx | xx | xx |
| **SOC 1** | xx (xx.x) | xx | xx | xx | xx | xx | xx |
| PT 1 | xx (xx.x) | xx | xx | xx | xx | xx | xx |
| PT 2 | xx (xx.x) | xx | xx | xx | xx | xx | xx |
| … | | | | | | | |
| **SOC 2** | xx (xx.x) | xx | xx | xx | xx | xx | xx |
| PT 1 | xx (xx.x) | xx | xx | xx | xx | xx | xx |
| PT 2 | xx (xx.x) | xx | xx | xx | xx | xx | xx |
| … | | | | | | | |
| … | | | | | | | |

All rights reserved. No part of this material may be reproduced or copied in any form by any means (written, electronic, photocopying, recording or otherwise) without the prior consent of BioClever.

**Tabla 2.3.5 Covid-19 symptoms registered by the medical staff – I – (B)**
**Tabla 2.3.6 Covid-19 symptoms registered by the medical staff – II – (B)**
**Tabla 2.3.7 Covid-19 symptoms registered by the medical staff – III – (B)**

All rights reserved. No part of this material may be reproduced or copied in any form by any means (written, electronic, photocopying, recording or otherwise) without the prior consent of BioClever.

## 9.3 Efficacy primary objective

### 9.3.1 Intention-to-treat set (ITT)

#### 9.3.1.1 Hospitalization rate

**Tabla 3.1.1 Hospitalization through day 28 per group**

| | | A (n=xx) | B (n=xx) |
|---|---|---|---|
| **Hospitalisation** | | | |
| Total no-missing | n | xx | xx |
| Yes | n (%) | xx (xx.x%) | xx (xx.x%) |
| No | n (%) | xx (xx.x%) | xx (xx.x%) |
| Missing | n | xx | xx |
| | pvalue | x.xxxx | |

**Tabla 3.1.2 Log-binomial regression model 1 – basic model**

| | n | Missing | RR, 95% CI | pvalue |
|---|---|---|---|---|
| **Model** | xx | xx | | |
| Group of treatment | | | x.xxx (x.xxx, x.xxx) | x.xxxx |

**Tabla 3.1.3 Log-binomial regression model 2 – one-covariate model**

| | n | Missing | RR, 95% CI | pvalue |
|---|---|---|---|---|
| **Model** | xx | xx | | |
| Group of treatment | | | x.xxx (x.xxx, x.xxx) | x.xxxx |
| Variable I* | | | x.xxx (x.xxx, x.xxx) | x.xxxx |

* Every independent variable at a time.

All rights reserved. No part of this material may be reproduced or copied in any form by any means (written, electronic, photocopying, recording or otherwise) without the prior consent of BioClever.

**Tabla 3.1.4 Log-binomial regression model 3 – final selected model**

| | n | Missing | RR, 95% CI | pvalue |
|---|---|---|---|---|
| **Model** | xx | xx | | |
| Groupf of treatment | | | x.xxx (x.xxx, x.xxx) | x.xxxx |
| Variable 1 | | | x.xxx (x.xxx, x.xxx) | x.xxxx |
| Variable 2 | | | x.xxx (x.xxx, x.xxx) | x.xxxx |
| … | | | x.xxx (x.xxx, x.xxx) | x.xxxx |

**Tabla 3.1.5 Log-binomial regression model 4 – final model**

| | n | Missing | RR, 95% CI | pvalue |
|---|---|---|---|---|
| **Model** | xx | xx | | |
| Groupf of treatment | | | x.xxx (x.xxx, x.xxx) | x.xxxx |
| Variable 1 | | | x.xxx (x.xxx, x.xxx) | x.xxxx |
| Variable 2 | | | x.xxx (x.xxx, x.xxx) | x.xxxx |
| … | | | x.xxx (x.xxx, x.xxx) | x.xxxx |

### 9.3.1.1.1. Subgrups analysis: according to serostatus at baseline

**Tabla 3.1.6 Hospitalization through day 28 per group - Seropositives**

**Tabla 3.1.7 Log-binomial regression model 1 – basic model**

**Tabla 3.1.8 Log-binomial regression model 2 – one-covariate model**

* Every independent variable at a time.

**Tabla 3.1.9 Log-binomial regression model 3 – final selected model**

**Tabla 3.1.10Log-binomial regression model 4 – final model**

**Tabla 3.1.11 Hospitalization through day 28 per group - Seronegatives**

**Tabla 3.1.12 Log-binomial regression model 1 – basic model**

**Tabla 3.1.13 Log-binomial regression model 2 – one-covariate model**

* Every independent variable at a time.

**Tabla 3.1.14 Log-binomial regression model 3 – final selected model**

**Tabla 3.1.15 Log-binomial regression model 4 – final model**

All rights reserved. No part of this material may be reproduced or copied in any form by any means (written, electronic, photocopying, recording or otherwise) without the prior consent of BioClever.

#### 9.3.1.1.2. Subgroups according to duration of illness

**Tabla 3.1.16 Hospitalization through day 28 per group - ≤3 days**

**Tabla 3.1.17 Log-binomial regression model 1 – basic model**

**Tabla 3.1.18 Log-binomial regression model 2 – one-covariate model**

* Every independent variable at a time.

**Tabla 3.1.19 Log-binomial regression model 3 – final selected model**

**Tabla 3.1.20 Log-binomial regression model 4 – final model**

**Tabla 3.1.21 Hospitalization through day 28 per group - >3 days**

**Tabla 3.1.22 Log-binomial regression model 1 – basic model**

**Tabla 3.1.23 Log-binomial regression model 2 – one-covariate model**

* Every independent variable at a time.

**Tabla 3.1.24 Log-binomial regression model 3 – final selected model**

**Tabla 3.1.25 Log-binomial regression model 4 – final model**

#### 9.3.1.1.3. Subgroups according to plasma neutralization activity

**Tabla 3.1.26 Hospitalization through day 28 per group – ID50>250**

**Tabla 3.1.27 Log-binomial regression model 1 – basic model**

**Tabla 3.1.28 Log-binomial regression model 2 – one-covariate model**

* Every independent variable at a time.

**Tabla 3.1.29 Log-binomial regression model 3 – final selected model**

**Tabla 3.1.30 Log-binomial regression model 4 – final model**

**Tabla 3.1.31 Hospitalization through day 28 per group – ID50 ≤250**

**Tabla 3.1.32 Log-binomial regression model 1 – basic model**

**Tabla 3.1.33 Log-binomial regression model 2 – one-covariate model**

* Every independent variable at a time.

**Tabla 3.1.34 Log-binomial regression model 3 – final selected model**

**Tabla 3.1.35 Log-binomial regression model 4 – final model**

All rights reserved. No part of this material may be reproduced or copied in any form by any means (written, electronic, photocopying, recording or otherwise) without the prior consent of BioClever.

### 9.3.1.2 Viral load

**Tabla 3.1.36 Positives, negatives and undetectables at each time point**

| | | **Group A (n=xx)** | **Group B (n=xx)** |
|---|---|---|---|
| Baseline | | | |
| Total no-missing | n | xx | xx |
| Negative | n (%) | xx ( x.x%) | xx ( xx.x%) |
| Positive | n (%) | xx ( xx.x%) | xx ( xx.x%) |
| Undetectable | n (%) | xx ( xx.x%) | xx ( xx.x%) |
| Missing | n | xx | xx |
| Day 7 | | | |
| Total no-missing | n | xx | xx |
| Negative | n (%) | xx ( x.x%) | xx ( x.x%) |
| Positive | n (%) | xx ( xx.x%) | xx ( xx.x%) |
| Undetectable | n (%) | xx ( xx.x%) | xx ( xx.x%) |
| Missing | n | xx | xx |
| Day 28 | | | |
| Total no-missing | n | xx | xx |
| Negative | n (%) | xx ( x.x%) | xx ( x.x%) |
| Positive | n (%) | xx ( xx.x%) | xx ( xx.x%) |
| Undetectable | n (%) | xx ( xx.x%) | xx ( xx.x%) |
| Missing | n | xx | xx |

All rights reserved. No part of this material may be reproduced or copied in any form by any means (written, electronic, photocopying, recording or otherwise) without the prior consent of BioClever.

-82:1547 .7;3893 0-) ,#%"//*$ ,%%"$',' ($! &,*./,&+'%+'



## Tabla 3.1.37 Viral load* reduction by group in logarithm$_{10}$ scale

| | n | Mean (SD) | Median (Q1, Q3) | Min, Max | Missing |
|---|---|---|---|---|---|
| Group A | | | | | |
| Baseline | xx | xx.x (xx.x) | xx (xx, xx) | xx, xx | xx |
| Day 7 | xx | xx.x (xx.x) | xx (xx, xx) | xx, xx | xx |
| Day 28 | xx | xx.x (xx.x) | xx (xx, xx) | xx, xx | xx |
| Reduction (Day 7 - baseline) | xx | xx.x (xx.x) | xx (xx, xx) | xx, xx | xx |
| Reduction (Day 28 - baseline) | xx | xx.x (xx.x) | xx (xx, xx) | xx, xx | xx |
| Group B | | | | | |
| Baseline | xx | xx.x (xx.x) | xx (xx, xx) | xx, xx | xx |
| Day 7 | xx | xx.x (xx.x) | xx (xx, xx) | xx, xx | xx |
| Day 28 | xx | xx.x (xx.x) | xx (xx, xx) | xx, xx | xx |
| Reduction (Day 7 - baseline) | xx | xx.x (xx.x) | xx (xx, xx) | xx, xx | xx |
| Reduction (Day 28 - baseline) | xx | xx.x (xx.x) | xx (xx, xx) | xx, xx | xx |

*Viral load (Copies/ml) in logarithm scale

## Figure 1 Reduction of logarithm of Viral load by group

## Tabla 3.1.38 Viral load reduction – Model 1 – basic model

| | n | Missing | Estimate (CI, 95%) | pvalue |
|---|---|---|---|---|
| **Model 1** | xx | xx | | |
| Group: A vs B | | | xx.x (xx.x, xx.x) | x.xxxx |
| Visit day | | | xx.x (xx.x, xx.x) | x.xxxx |
| Group*visit day | | | xx.x (xx.x, xx.x) | x.xxxx |

## Tabla 3.1.39 Viral load reduction – Model 2 – one covariate

| | n | Missing | Estimate (CI, 95%) | pvalue |
|---|---|---|---|---|

All rights reserved. No part of this material may be reproduced or copied in any form by any means (written, electronic, photocopying, recording or otherwise) without the prior consent of BioClever.

| | n | Missing | Estimate (CI, 95%) | pvalue |
|---|---|---|---|---|
| **Model 2** | xx | xx | | |
| Covariate I* | | | xx.x (xx.x, xx.x) | x.xxxx |

* Every independent covariate at a time adjusted by group, visit day and group*visit day.

**Tabla 3.1.40 Viral load reduction – Model 3 – final selected model**

| | n | Missing | Estimate (CI, 95%) | pvalue |
|---|---|---|---|---|
| **Model 3** | xx | xx | | |
| Group: A vs B | | | xx.x (xx.x, xx.x) | x.xxxx |
| Visit day | | | xx.x (xx.x, xx.x) | x.xxxx |
| Group*visit day | | | xx.x (xx.x, xx.x) | x.xxxx |
| Covariate I | | | xx.x (xx.x, xx.x) | x.xxxx |
| … | | | xx.x (xx.x, xx.x) | x.xxxx |

**Tabla 3.1.41 Viral load reduction – Model 4 – final model**

| | n | Missing | Estimate (CI, 95%) | pvalue |
|---|---|---|---|---|
| **Model 4** | xx | xx | | |
| Group: A vs B | | | xx.x (xx.x, xx.x) | x.xxxx |
| Visit day | | | xx.x (xx.x, xx.x) | x.xxxx |
| Group*visit day | | | xx.x (xx.x, xx.x) | x.xxxx |
| Covariate I | | | xx.x (xx.x, xx.x) | x.xxxx |
| … | | | xx.x (xx.x, xx.x) | x.xxxx |

## 9.3.1.1.4. Subgrups analysis: according to serostatus at baseline

**Tabla 3.1.42Viral load* reduction by group in logarithm$_{10}$ scale - Seropositives**

**Tabla 3.1.43Viral load reduction – Model 1 – basic model**

**Tabla 3.1.44Viral load reduction – Model 2 – one covariate**

**Tabla 3.1.45 Viral load reduction – Model 3 – final selected model**

All rights reserved. No part of this material may be reproduced or copied in any form by any means (written, electronic, photocopying, recording or otherwise) without the prior consent of BioClever.

**Tabla 3.1.46 Viral load reduction – Model 4 – final model**

**Tabla 3.1.47Viral load* reduction by group in logarithm$_{10}$ scale - Seronegatives**
**Tabla 3.1.48Viral load reduction – Model 1 – basic model**
**Tabla 3.1.49Viral load reduction – Model 2 – one covariate**
**Tabla 3.1.50 Viral load reduction – Model 3 – final selected model**
**Tabla 3.1.51 Viral load reduction – Model 4 – final model**

### 9.3.1.1.5. Subgrups analysis: according to duration of illness

**Tabla 3.1.52Viral load* reduction by group in logarithm$_{10}$ scale - ≤ 3 days**
**Tabla 3.1.53Viral load reduction – Model 1 – basic model**
**Tabla 3.1.54Viral load reduction – Model 2 – one covariate**
**Tabla 3.1.55 Viral load reduction – Model 3 – final selected model**
**Tabla 3.1.56 Viral load reduction – Model 4 – final model**

**Tabla 3.1.57Viral load* reduction by group in logarithm$_{10}$ scale - >3 days**
**Tabla 3.1.58Viral load reduction – Model 1 – basic model**
**Tabla 3.1.59Viral load reduction – Model 2 – one covariate**
**Tabla 3.1.60 Viral load reduction – Model 3 – final selected model**
**Tabla 3.1.61 Viral load reduction – Model 4 – final model**

### 9.3.1.1.6. Subgrups analysis: according to plasma neutralization activity

**Tabla 3.1.62Viral load* reduction by group in logarithm$_{10}$ scale – ID50>250**
**Tabla 3.1.63Viral load reduction – Model 1 – basic model**
**Tabla 3.1.64Viral load reduction – Model 2 – one covariate**
**Tabla 3.1.65 Viral load reduction – Model 3 – final selected model**
**Tabla 3.1.66 Viral load reduction – Model 4 – final model**

**Tabla 3.1.67Viral load* reduction by group in logarithm$_{10}$ scale - ID50 ≤250**
**Tabla 3.1.68Viral load reduction – Model 1 – basic model**
**Tabla 3.1.69Viral load reduction – Model 2 – one covariate**
**Tabla 3.1.70 Viral load reduction – Model 3 – final selected model**
**Tabla 3.1.71 Viral load reduction – Model 4 – final model**

All rights reserved. No part of this material may be reproduced or copied in any form by any means (written, electronic, photocopying, recording or otherwise) without the prior consent of BioClever.

### 9.3.2 Per protocol (PP)

#### 9.3.2.1 Hospitalisation rate

**Tabla 3.2.1 Hospitalization per group**

**Tabla 3.2.2 Log-binomial regression model 1 – basic model**

**Tabla 3.2.3 Log-binomial regression model 2 – one-covariate model**

* Every independent variable at a time.

**Tabla 3.2.4 Log-binomial regression model 3 – final selected model**

**Tabla 3.2.5 Log-binomial regression model 4 – final model**

#### 9.3.2.2 Viral load

**Tabla 3.2.6 Positives, negatives and undetectable according to original values of CT**

**Tabla 3.2.7 Viral load* reduction by group in logarithm scale**

*Viral load (Copies/ml) in logarithm scale

**Figure 2 Reduction of logarithm of Viral load by group**

**Tabla 3.2.8 Viral load reduction – Model 1 – basic model**

**Tabla 3.2.9 Viral load* reduction – Model 2 – one covariate**

* Every independent covariate at a time adjusted by group, visit day and group*visit day.

**Tabla 3.2.10 Viral load reduction – Model 3 – final selected model**

**Tabla 3.2.11 Viral load reduction – Model 4 – final model**

## 9.4 Efficacy secondary objectives – ITT

### 9.4.1 COVID-19 WHO scale score progression

**Tabla 4.1.1 Covid-19 WHO scale score progression I**

All rights reserved. No part of this material may be reproduced or copied in any form by any means (written, electronic, photocopying, recording or otherwise) without the prior consent of BioClever.

| | | **A**<br>**(n=xx)** | **B**<br>**(n=xx)** |
|---|---|---|---|
| Baseline | | | |
| Total no-missing | n | xx | xx |
| 0: Uninfected. No viral RNA detected. | n (%) | xx (xx.x%) | xx (xx.x%) |
| 1: Asymptomatic. Viral RNA detected. | n (%) | xx (xx.x%) | xx (xx.x%) |
| 2: Symptomatic. Independent. | n (%) | xx (xx.x%) | xx (xx.x%) |
| 3: Symptomatic. Assistance needed. | n (%) | xx (xx.x%) | xx (xx.x%) |
| 4: Hospitalized. No oxygen therapy. | n (%) | xx (xx.x%) | xx (xx.x%) |
| 5: Hospitalized. Oxygen by mask or nasal prongs. | n (%) | xx (xx.x%) | xx (xx.x%) |
| 6: Hospitalized. Oxygen by NIV or high flow. | n (%) | xx (xx.x%) | xx (xx.x%) |
| 7: Intubation and mechanical ventilation. $pO_2/FIO_2$ ≥150 or $SpO_2/FI0_2$ ≥ 200 | n (%) | xx (xx.x%) | xx (xx.x%) |
| 8: Mechanical ventilation $pO_2/FIO_2$< 150 ($SpO_2/FI0_2$ < 200) or vasopressors. | n (%) | xx (xx.x%) | xx (xx.x%) |
| 9: Mechanical ventilation $pO_2/FIO_2$ <150 and vasopressors, dialysis, or ECMO | n (%) | xx (xx.x%) | xx (xx.x%) |
| 10: Death. | n (%) | xx (xx.x%) | xx (xx.x%) |
| Missing | n | xx | xx |
| | pvalue | x.xxxx | |
| Day 3 - telephone | | | |
| Total no-missing | n | xx | xx |
| 0: Uninfected. No viral RNA detected. | n (%) | xx (xx.x%) | xx (xx.x%) |
| 1: Asymptomatic. Viral RNA detected. | n (%) | xx (xx.x%) | xx (xx.x%) |
| 2: Symptomatic. Independent. | n (%) | xx (xx.x%) | xx (xx.x%) |
| 3: Symptomatic. Assistance needed. | n (%) | xx (xx.x%) | xx (xx.x%) |
| 4: Hospitalized. No oxygen therapy. | n (%) | xx (xx.x%) | xx (xx.x%) |
| 5: Hospitalized. Oxygen by mask or nasal prongs. | n (%) | xx (xx.x%) | xx (xx.x%) |

All rights reserved. No part of this material may be reproduced or copied in any form by any means (written, electronic, photocopying, recording or otherwise) without the prior consent of BioClever.

| | | A (n=xx) | B (n=xx) |
|---|---|---|---|
| 6: Hospitalized. Oxygen by NIV or high flow. | n (%) | xx (xx.x%) | xx (xx.x%) |
| 7: Intubation and mechanical ventilation. $pO_2/FIO_2$ ≥150 or $SpO_2/FIO_2$ ≥ 200 | n (%) | xx (xx.x%) | xx (xx.x%) |
| 8: Mechanical ventilation $pO_2/FIO_2$< 150 ($SpO_2/FIO_2$ < 200) or vasopressors. | n (%) | xx (xx.x%) | xx (xx.x%) |
| 9: Mechanical ventilation $pO_2/FIO_2$ <150 and vasopressors, dialysis, or ECMO | n (%) | xx (xx.x%) | xx (xx.x%) |
| 10: Death. | n (%) | xx (xx.x%) | xx (xx.x%) |
| Missing | n | xx | xx |
| | pvalue | x.xxxx | |
| | | | |
| Day 7 - home | | | |
| Total no-missing | n | xx | xx |
| 0: Uninfected. No viral RNA detected. | n (%) | xx (xx.x%) | xx (xx.x%) |
| 1: Asymptomatic. Viral RNA detected. | n (%) | xx (xx.x%) | xx (xx.x%) |
| 2: Symptomatic. Independent. | n (%) | xx (xx.x%) | xx (xx.x%) |
| 3: Symptomatic. Assistance needed. | n (%) | xx (xx.x%) | xx (xx.x%) |
| 4: Hospitalized. No oxygen therapy. | n (%) | xx (xx.x%) | xx (xx.x%) |
| 5: Hospitalized. Oxygen by mask or nasal prongs. | n (%) | xx (xx.x%) | xx (xx.x%) |
| 6: Hospitalized. Oxygen by NIV or high flow. | n (%) | xx (xx.x%) | xx (xx.x%) |
| 7: Intubation and mechanical ventilation. $pO_2/FIO_2$ ≥150 or $SpO_2/FIO_2$ ≥ 200 | n (%) | xx (xx.x%) | xx (xx.x%) |
| 8: Mechanical ventilation $pO_2/FIO_2$< 150 ($SpO_2/FIO_2$ < 200) or vasopressors. | n (%) | xx (xx.x%) | xx (xx.x%) |
| 9: Mechanical ventilation $pO_2/FIO_2$ <150 and vasopressors, dialysis, or ECMO | n (%) | xx (xx.x%) | xx (xx.x%) |
| 10: Death. | n (%) | xx (xx.x%) | xx (xx.x%) |
| Missing | n | xx | xx |

All rights reserved. No part of this material may be reproduced or copied in any form by any means (written, electronic, photocopying, recording or otherwise) without the prior consent of BioClever.

| | | A (n=xx) | B (n=xx) |
|---|---|---|---|
| | pvalue | x.xxxx | |
| Day 14 - telephone | | | |
| Total no-missing | n | xx | xx |
| 0: Uninfected. No viral RNA detected. | n (%) | xx (xx.x%) | xx (xx.x%) |
| 1: Asymptomatic. Viral RNA detected. | n (%) | xx (xx.x%) | xx (xx.x%) |
| 2: Symptomatic. Independent. | n (%) | xx (xx.x%) | xx (xx.x%) |
| 3: Symptomatic. Assistance needed. | n (%) | xx (xx.x%) | xx (xx.x%) |
| 4: Hospitalized. No oxygen therapy. | n (%) | xx (xx.x%) | xx (xx.x%) |
| 5: Hospitalized. Oxygen by mask or nasal prongs. | n (%) | xx (xx.x%) | xx (xx.x%) |
| 6: Hospitalized. Oxygen by NIV or high flow. | n (%) | xx (xx.x%) | xx (xx.x%) |
| 7: Intubation and mechanical ventilation. $pO_2/FIO_2$ ≥150 or $SpO_2/FI0_2$ ≥ 200 | n (%) | xx (xx.x%) | xx (xx.x%) |
| 8: Mechanical ventilation $pO_2/FIO_2$< 150 ($SpO_2/FI0_2$ < 200) or vasopressors. | n (%) | xx (xx.x%) | xx (xx.x%) |
| 9: Mechanical ventilation $pO_2/FIO_2$ <150 and vasopressors, dialysis, or ECMO | n (%) | xx (xx.x%) | xx (xx.x%) |
| 10: Death. | n (%) | xx (xx.x%) | xx (xx.x%) |
| Missing | n | xx | xx |
| | pvalue | x.xxxx | |
| Day 28 - hospital | | | |
| Total no-missing | n | xx | xx |
| 0: Uninfected. No viral RNA detected. | n (%) | xx (xx.x%) | xx (xx.x%) |
| 1: Asymptomatic. Viral RNA detected. | n (%) | xx (xx.x%) | xx (xx.x%) |
| 2: Symptomatic. Independent. | n (%) | xx (xx.x%) | xx (xx.x%) |
| 3: Symptomatic. Assistance needed. | n (%) | xx (xx.x%) | xx (xx.x%) |

All rights reserved. No part of this material may be reproduced or copied in any form by any means (written, electronic, photocopying, recording or otherwise) without the prior consent of BioClever.

| | | **A** (n=xx) | **B** (n=xx) |
|---|---|---|---|
| 4: Hospitalized. No oxygen therapy. | n (%) | xx (xx.x%) | xx (xx.x%) |
| 5: Hospitalized. Oxygen by mask or nasal prongs. | n (%) | xx (xx.x%) | xx (xx.x%) |
| 6: Hospitalized. Oxygen by NIV or high flow. | n (%) | xx (xx.x%) | xx (xx.x%) |
| 7: Intubation and mechanical ventilation. $pO_2/FIO_2$ ≥150 or $SpO_2/FI0_2$ ≥ 200 | n (%) | xx (xx.x%) | xx (xx.x%) |
| 8: Mechanical ventilation $pO_2/FIO_2$< 150 ($SpO_2/FI0_2$ < 200) or vasopressors. | n (%) | xx (xx.x%) | xx (xx.x%) |
| 9: Mechanical ventilation $pO_2/FIO_2$ <150 and vasopressors, dialysis, or ECMO | n (%) | xx (xx.x%) | xx (xx.x%) |
| 10: Death. | n (%) | xx (xx.x%) | xx (xx.x%) |
| Missing | n | xx | xx |
| | pvalue | x.xxxx | |
| | | | |
| Day 60 - telephone | | | |
| Total no-missing | n | xx | xx |
| 0: Uninfected. No viral RNA detected. | n (%) | xx (xx.x%) | xx (xx.x%) |
| 1: Asymptomatic. Viral RNA detected. | n (%) | xx (xx.x%) | xx (xx.x%) |
| 2: Symptomatic. Independent. | n (%) | xx (xx.x%) | xx (xx.x%) |
| 3: Symptomatic. Assistance needed. | n (%) | xx (xx.x%) | xx (xx.x%) |
| 4: Hospitalized. No oxygen therapy. | n (%) | xx (xx.x%) | xx (xx.x%) |
| 5: Hospitalized. Oxygen by mask or nasal prongs. | n (%) | xx (xx.x%) | xx (xx.x%) |
| 6: Hospitalized. Oxygen by NIV or high flow. | n (%) | xx (xx.x%) | xx (xx.x%) |
| 7: Intubation and mechanical ventilation. $pO_2/FIO_2$ ≥150 or $SpO_2/FI0_2$ ≥ 200 | n (%) | xx (xx.x%) | xx (xx.x%) |
| 8: Mechanical ventilation $pO_2/FIO_2$< 150 ($SpO_2/FI0_2$ < 200) or vasopressors. | n (%) | xx (xx.x%) | xx (xx.x%) |
| 9: Mechanical ventilation $pO_2/FIO_2$ <150 and vasopressors, dialysis, or ECMO | n (%) | xx (xx.x%) | xx (xx.x%) |

All rights reserved. No part of this material may be reproduced or copied in any form by any means (written, electronic, photocopying, recording or otherwise) without the prior consent of BioClever.

| | | A (n=xx) | B (n=xx) |
|---|---|---|---|
| 10: Death. | n (%) | xx (xx.x%) | xx (xx.x%) |
| Missing | n | xx | xx |
| | pvalue | x.xxxx | |

**Tabla 4.1.2 Covid-19 WHO scale progression I – extra visits**

| | | A (n=xx) | B (n=xx) |
|---|---|---|---|
| Visit Extra 1 | | | |
| Total no-missing | n | xx | xx |
| 0: Uninfected. No viral RNA detected. | n (%) | xx (xx.x%) | xx (xx.x%) |
| 1: Asymptomatic. Viral RNA detected. | n (%) | xx (xx.x%) | xx (xx.x%) |
| 2: Symptomatic. Independent. | n (%) | xx (xx.x%) | xx (xx.x%) |
| 3: Symptomatic. Assistance needed. | n (%) | xx (xx.x%) | xx (xx.x%) |
| 4: Hospitalized. No oxygen therapy. | n (%) | xx (xx.x%) | xx (xx.x%) |
| 5: Hospitalized. Oxygen by mask or nasal prongs. | n (%) | xx (xx.x%) | xx (xx.x%) |
| 6: Hospitalized. Oxygen by NIV or high flow. | n (%) | xx (xx.x%) | xx (xx.x%) |
| 7: Intubation and mechanical ventilation. $pO_2/FIO_2 \geq 150$ or $SpO_2/FI0_2 \geq 200$ | n (%) | xx (xx.x%) | xx (xx.x%) |
| 8: Mechanical ventilation $pO_2/FIO_2 < 150$ ($SpO_2/FI0_2 < 200$) or vasopressors. | n (%) | xx (xx.x%) | xx (xx.x%) |
| 9: Mechanical ventilation $pO_2/FIO_2 < 150$ and vasopressors, dialysis, or ECMO | n (%) | xx (xx.x%) | xx (xx.x%) |
| 10: Death. | n (%) | xx (xx.x%) | xx (xx.x%) |
| Missing | n | xx | xx |
| Visit Extra 2 | | | |

All rights reserved. No part of this material may be reproduced or copied in any form by any means (written, electronic, photocopying, recording or otherwise) without the prior consent of BioClever.

| | | A (n=xx) | B (n=xx) |
|---|---|---|---|
| Total no-missing | n | xx | xx |
| 0: Uninfected. No viral RNA detected. | n (%) | xx (xx.x%) | xx (xx.x%) |
| 1: Asymptomatic. Viral RNA detected. | n (%) | xx (xx.x%) | xx (xx.x%) |
| 2: Symptomatic. Independent. | n (%) | xx (xx.x%) | xx (xx.x%) |
| 3: Symptomatic. Assistance needed. | n (%) | xx (xx.x%) | xx (xx.x%) |
| 4: Hospitalized. No oxygen therapy. | n (%) | xx (xx.x%) | xx (xx.x%) |
| 5: Hospitalized. Oxygen by mask or nasal prongs. | n (%) | xx (xx.x%) | xx (xx.x%) |
| 6: Hospitalized. Oxygen by NIV or high flow. | n (%) | xx (xx.x%) | xx (xx.x%) |
| 7: Intubation and mechanical ventilation. $pO_2/FIO_2 \geq 150$ or $SpO_2/FI0_2 \geq 200$ | n (%) | xx (xx.x%) | xx (xx.x%) |
| 8: Mechanical ventilation $pO_2/FIO_2 < 150$ ($SpO_2/FI0_2 < 200$) or vasopressors. | n (%) | xx (xx.x%) | xx (xx.x%) |
| 9: Mechanical ventilation $pO_2/FIO_2 < 150$ and vasopressors, dialysis, or ECMO | n (%) | xx (xx.x%) | xx (xx.x%) |
| 10: Death. | n (%) | xx (xx.x%) | xx (xx.x%) |
| Missing | n | xx | xx |
| … | | | |
| Visit Extra 10 | | | |
| Total no-missing | n | xx | xx |
| 0: Uninfected. No viral RNA detected. | n (%) | xx (xx.x%) | xx (xx.x%) |
| 1: Asymptomatic. Viral RNA detected. | n (%) | xx (xx.x%) | xx (xx.x%) |
| 2: Symptomatic. Independent. | n (%) | xx (xx.x%) | xx (xx.x%) |
| 3: Symptomatic. Assistance needed. | n (%) | xx (xx.x%) | xx (xx.x%) |
| 4: Hospitalized. No oxygen therapy. | n (%) | xx (xx.x%) | xx (xx.x%) |
| 5: Hospitalized. Oxygen by mask or nasal prongs. | n (%) | xx (xx.x%) | xx (xx.x%) |
| 6: Hospitalized. Oxygen by NIV or high flow. | n (%) | xx (xx.x%) | xx (xx.x%) |

All rights reserved. No part of this material may be reproduced or copied in any form by any means (written, electronic, photocopying, recording or otherwise) without the prior consent of BioClever.

| | | A (n=xx) | B (n=xx) |
|---|---|---|---|
| 7: Intubation and mechanical ventilation. $pO_2/FIO_2$ ≥150 or $SpO_2/FIO_2$ ≥ 200 | n (%) | xx (xx.x%) | xx (xx.x%) |
| 8: Mechanical ventilation $pO_2/FIO_2$< 150 ($SpO_2/FIO_2$ < 200) or vasopressors. | n (%) | xx (xx.x%) | xx (xx.x%) |
| 9: Mechanical ventilation $pO_2/FIO_2$ <150 and vasopressors, dialysis, or ECMO | n (%) | xx (xx.x%) | xx (xx.x%) |
| 10: Death. | n (%) | xx (xx.x%) | xx (xx.x%) |
| Missing | n | xx | xx |

### Tabla 4.1.3 Covid-19 WHO scale score progression II

| | | A (n=xx) | B (n=xx) |
|---|---|---|---|
| Baseline | | | |
| Total no-missing | n | xx | xx |
| No infection (0 or 1) | n (%) | xx (xx.x%) | xx (xx.x%) |
| Mild (2 or 3) | n (%) | xx (xx.x%) | xx (xx.x%) |
| Moderate (4 or 5) | n (%) | xx (xx.x%) | xx (xx.x%) |
| Severe (from 6 to 9) | n (%) | xx (xx.x%) | xx (xx.x%) |
| Death (10) | n (%) | xx (xx.x%) | xx (xx.x%) |
| Missing | n | xx | xx |
| | pvalue | x.xxxx | |
| Day 3 - telephone | | | |
| Total no-missing | n | xx | xx |
| No infection (0 or 1) | n (%) | xx (xx.x%) | xx (xx.x%) |
| Mild (2 or 3) | n (%) | xx (xx.x%) | xx (xx.x%) |
| Moderate (4 or 5) | n (%) | xx (xx.x%) | xx (xx.x%) |

All rights reserved. No part of this material may be reproduced or copied in any form by any means (written, electronic, photocopying, recording or otherwise) without the prior consent of BioClever.

| | | A (n=xx) | B (n=xx) |
|---|---|---|---|
| Severe (from 6 to 9) | n (%) | xx (xx.x%) | xx (xx.x%) |
| Death (10) | n (%) | xx (xx.x%) | xx (xx.x%) |
| Missing | n | xx | xx |
| | pvalue | x.xxxx | |
| Day 7 - home | | | |
| Total no-missing | n | xx | xx |
| No infection (0 or 1) | n (%) | xx (xx.x%) | xx (xx.x%) |
| Mild (2 or 3) | n (%) | xx (xx.x%) | xx (xx.x%) |
| Moderate (4 or 5) | n (%) | xx (xx.x%) | xx (xx.x%) |
| Severe (from 6 to 9) | n (%) | xx (xx.x%) | xx (xx.x%) |
| Death (10) | n (%) | xx (xx.x%) | xx (xx.x%) |
| Missing | n | xx | xx |
| | pvalue | x.xxxx | |
| Day 14 - telephone | | | |
| Total no-missing | n | xx | xx |
| No infection (0 or 1) | n (%) | xx (xx.x%) | xx (xx.x%) |
| Mild (2 or 3) | n (%) | xx (xx.x%) | xx (xx.x%) |
| Moderate (4 or 5) | n (%) | xx (xx.x%) | xx (xx.x%) |
| Severe (from 6 to 9) | n (%) | xx (xx.x%) | xx (xx.x%) |
| Death (10) | n (%) | xx (xx.x%) | xx (xx.x%) |
| Missing | n | xx | xx |
| | pvalue | x.xxxx | |
| Day 28 - hospital | | | |

All rights reserved. No part of this material may be reproduced or copied in any form by any means (written, electronic, photocopying, recording or otherwise) without the prior consent of BioClever.

| | | **A**<br>**(n=xx)** | **B**<br>**(n=xx)** |
|---|---|---|---|
| Total no-missing | n | xx | xx |
| No infection (0 or 1) | n (%) | xx (xx.x%) | xx (xx.x%) |
| Mild (2 or 3) | n (%) | xx (xx.x%) | xx (xx.x%) |
| Moderate (4 or 5) | n (%) | xx (xx.x%) | xx (xx.x%) |
| Severe (from 6 to 9) | n (%) | xx (xx.x%) | xx (xx.x%) |
| Death (10) | n (%) | xx (xx.x%) | xx (xx.x%) |
| Missing | n | xx | xx |
| | pvalue | x.xxxx | |
| Day 60 - telephone | | | |
| Total no-missing | n | xx | xx |
| No infection (0 or 1) | n (%) | xx (xx.x%) | xx (xx.x%) |
| Mild (2 or 3) | n (%) | xx (xx.x%) | xx (xx.x%) |
| Moderate (4 or 5) | n (%) | xx (xx.x%) | xx (xx.x%) |
| Severe (from 6 to 9) | n (%) | xx (xx.x%) | xx (xx.x%) |
| Death (10) | n (%) | xx (xx.x%) | xx (xx.x%) |
| Missing | n | xx | xx |
| | pvalue | x.xxxx | |

**Tabla 4.1.4 Covid-19 WHO scale progression by group – extra visits**

| | | **A**<br>**(n=xx)** | **B**<br>**(n=xx)** |
|---|---|---|---|
| Visit Extra 1 | | | |
| Total no-missing | n | xx | xx |
| No infection (0 or 1) | n (%) | xx (xx.x%) | xx (xx.x%) |
| Mild (2 or 3) | n (%) | xx (xx.x%) | xx (xx.x%) |

All rights reserved. No part of this material may be reproduced or copied in any form by any means (written, electronic, photocopying, recording or otherwise) without the prior consent of BioClever.

| | | A (n=xx) | B (n=xx) |
|---|---|---|---|
| Moderate (4 or 5) | n (%) | xx (xx.x%) | xx (xx.x%) |
| Severe (from 6 to 9) | n (%) | xx (xx.x%) | xx (xx.x%) |
| Death (10) | n (%) | xx (xx.x%) | xx (xx.x%) |
| Missing | n | xx | xx |
| | | | |
| Visit Extra 2 | | | |
| Total no-missing | n | xx | xx |
| No infection (0 or 1) | n (%) | xx (xx.x%) | xx (xx.x%) |
| Mild (2 or 3) | n (%) | xx (xx.x%) | xx (xx.x%) |
| Moderate (4 or 5) | n (%) | xx (xx.x%) | xx (xx.x%) |
| Severe (from 6 to 9) | n (%) | xx (xx.x%) | xx (xx.x%) |
| Death (10) | n (%) | xx (xx.x%) | xx (xx.x%) |
| Missing | n | xx | xx |
| | | | |
| … | | | |
| Visit Extra 10 | | | |
| Total no-missing | n | xx | xx |
| No infection (0 or 1) | n (%) | xx (xx.x%) | xx (xx.x%) |
| Mild (2 or 3) | n (%) | xx (xx.x%) | xx (xx.x%) |
| Moderate (4 or 5) | n (%) | xx (xx.x%) | xx (xx.x%) |
| Severe (from 6 to 9) | n (%) | xx (xx.x%) | xx (xx.x%) |
| Death (10) | n (%) | xx (xx.x%) | xx (xx.x%) |
| Missing | n | xx | xx |

Figure 3 Longitudinal display of patients COVID-19 WHO score progression by group

### Tabla 4.1.5 WHO scale score by mixed model - Model 1

All rights reserved. No part of this material may be reproduced or copied in any form by any means (written, electronic, photocopying, recording or otherwise) without the prior consent of BioClever.

| | n | Missing | OR (IC 95%) | pvalue |
|---|---|---|---|---|
| **Model** | xx | xx | | |
| Intercept I | | | | x.xxxx |
| Intercept II | | | | x.xxxx |
| … | | | | x.xxxx |
| Group: A vs B | | | x.xx (x.xx, x.xx) | x.xxxx |

### Tabla 4.1.6 WHO scale score by mixed model – Model 2

| | n | Missing | OR (IC 95%) | pvalue |
|---|---|---|---|---|
| **Model** | xx | xx | | |
| Intercept I | | | | x.xxxx |
| Intercept II | | | | x.xxxx |
| … | | | | x.xxxx |
| Group: A vs B | | | x.xx (x.xx, x.xx) | x.xxxx |
| Covariate I* | | | x.xx (x.xx, x.xx) | x.xxxx |

* Every independent covariate at a time.

### Tabla 4.1.7 WHO scale score by mixed model – Model 3

| | n | Missing | OR (IC 95%) | pvalue |
|---|---|---|---|---|
| **Model** | xx | xx | | |
| Intercept I | | | | x.xxxx |
| Intercept II | | | | x.xxxx |
| … | | | | x.xxxx |
| Group: A vs B | | | x.xx (x.xx, x.xx) | x.xxxx |
| Covariate I* | | | x.xx (x.xx, x.xx) | x.xxxx |
| … | | | | |

### Tabla 4.1.8 WHO scale score by mixed model – Model 4

All rights reserved. No part of this material may be reproduced or copied in any form by any means (written, electronic, photocopying, recording or otherwise) without the prior consent of BioClever.

| | n | Missing | OR (IC 95%) | pvalue |
|---|---|---|---|---|
| **Model** | xx | xx | | |
| Intercept I | | | | x.xxxx |
| Intercept II | | | | x.xxxx |
| … | | | | x.xxxx |
| Group: A vs B | | | x.xx (x.xx, x.xx) | x.xxxx |
| Covariate I | | | x.xx (x.xx, x.xx) | x.xxxx |
| … | | | x.xx (x.xx, x.xx) | x.xxxx |

### 9.4.2 Time to COVID-19 symptoms resolution

**Tabla 4.2.1 Time to symptoms complete resolution**

| | | | A<br>(n=xx) | B<br>(n=xx) |
|---|---|---|---|---|
| **Symptoms complete resolution** | | | | |
| | Total no-missing | n | xx | xx |
| | Yes | n (%) | xx (xx.x%) | xx (xx.x%) |
| | No | n (%) | xx (xx.x%) | xx (xx.x%) |
| | Missing | n | xx | xx |
| Time to symptoms complete resolution | | pvalue[1] | x.xxxx | |
| | | P25 (CI95%) | xx.x (xx.x, xx.x) | xx.x (xx.x, xx.x) |
| | | P50 (CI95%) | xx.x (xx.x, xx.x) | xx.x (xx.x, xx.x) |
| | | P75 (CI95%) | xx.x (xx.x, xx.x) | xx.x (xx.x, xx.x) |

[1] **Log-rank test**

**Figure 4 Time to symptoms complete resolution (Kaplan Meyer)**

All rights reserved. No part of this material may be reproduced or copied in any form by any means (written, electronic, photocopying, recording or otherwise) without the prior consent of BioClever.

**Tabla 4.2.2 Symptoms complete resolution: Hazard Ratio (HR) by Cox proportional hazard model**

| | Hazard Ratio | IC 95% |
|---|---|---|
| Group A vs B | xx.x | (xx.x, xx.x) |

**Tabla 4.2.3 Symptoms completely resolved within the first 28 days after infusion**

| | | Group A (n=xx) | Group B (n=xx) |
|---|---|---|---|
| Symptoms resolved within the first 28 days after infusion | | | |
| Total no-missing | n | xx | xx |
| Not resolved | n (%) | xx ( xx.x%) | xx ( xx.x%) |
| Resolved | n (%) | xx ( xx.x%) | xx ( xx.x%) |
| Missing | n | xx | xx |
| | pvalue | x.xxxx | |

### 9.4.3 Death rate

**Tabla 4.3.1 Death per group**

| | | A (n=xx) | B (n=xx) |
|---|---|---|---|
| **Death** | | | |
| Total no-missing | n | xx | xx |
| Yes | n (%) | xx (xx.x%) | xx (xx.x%) |
| No | n (%) | xx (xx.x%) | xx (xx.x%) |
| Missing | n | xx | xx |
| | pvalue | x.xxxx | |

**Tabla 4.3.2 Log-binomial regression model 1 – basic model**

All rights reserved. No part of this material may be reproduced or copied in any form by any means (written, electronic, photocopying, recording or otherwise) without the prior consent of BioClever.

| | n | Missing | RR, 95% CI | pvalue |
|---|---|---|---|---|
| **Model** | xx | xx | | |
| Group of treatment | | | x.xxx (x.xxx, x.xxx) | x.xxxx |

**Tabla 4.3.3 Log-binomial regression model 2 – one-covariate model**

| | n | Missing | RR, 95% CI | pvalue |
|---|---|---|---|---|
| **Model** | xx | xx | | |
| Group of treatment | | | x.xxx (x.xxx, x.xxx) | x.xxxx |
| Variable I* | | | x.xxx (x.xxx, x.xxx) | x.xxxx |

* Every independent variable at a time.

**Tabla 4.3.4 Log-binomial regression model 3 – final selected model**

| | n | Missing | RR, 95% CI | pvalue |
|---|---|---|---|---|
| **Model** | xx | xx | | |
| Groupf of treatment | | | x.xxx (x.xxx, x.xxx) | x.xxxx |
| Variable 1 | | | x.xxx (x.xxx, x.xxx) | x.xxxx |
| Variable 2 | | | x.xxx (x.xxx, x.xxx) | x.xxxx |
| … | | | x.xxx (x.xxx, x.xxx) | x.xxxx |

**Tabla 4.3.5 Log-binomial regression model 4 – final model**

| | n | Missing | RR, 95% CI | pvalue |
|---|---|---|---|---|
| **Model** | xx | xx | | |
| Groupf of treatment | | | x.xxx (x.xxx, x.xxx) | x.xxxx |
| Variable 1 | | | x.xxx (x.xxx, x.xxx) | x.xxxx |
| Variable 2 | | | x.xxx (x.xxx, x.xxx) | x.xxxx |
| … | | | x.xxx (x.xxx, x.xxx) | x.xxxx |

All rights reserved. No part of this material may be reproduced or copied in any form by any means (written, electronic, photocopying, recording or otherwise) without the prior consent of BioClever.

### 9.4.4 Inflammatory prognostic markers

**Tabla 4.4.1 Change in D-dimer**

| | n | Mean (SD) | Median (Q1, Q3) | Min, Max | Missing | pvalue |
|---|---|---|---|---|---|---|
| Group A | | | | | | |
| Baseline | xx | xx.x (xx.x) | xx (xx, xx) | xx, xx | xx | |
| Day 7 | xx | xx.x (xx.x) | xx (xx, xx) | xx, xx | xx | |
| Change (Day 7 - baseline) | xx | xx.x (xx.x) | xx (xx, xx) | xx, xx | xx | x.xxxx |
| Group B | | | | | | |
| Baseline | xx | xx.x (xx.x) | xx (xx, xx) | xx, xx | xx | |
| Day 7 | xx | xx.x (xx.x) | xx (xx, xx) | xx, xx | xx | |
| Change (Day 7 - baseline) | xx | xx.x (xx.x) | xx (xx, xx) | xx, xx | xx | x.xxxx |

**Figure 5 Change in D-dimer by group**

**Tabla 4.4.2 Change in D-dimer – Model 1 – basic model**

| | n | Missing | Estimate (CI, 95%) | pvalue |
|---|---|---|---|---|
| **Model 1** | xx | xx | | |
| Group: A vs B | | | xx.x (xx.x, xx.x) | x.xxxx |
| Visit day | | | xx.x (xx.x, xx.x) | x.xxxx |
| Group*visit day | | | xx.x (xx.x, xx.x) | x.xxxx |

**Tabla 4.4.3 Change in D-dimer – Model 2 – one covariate**

All rights reserved. No part of this material may be reproduced or copied in any form by any means (written, electronic, photocopying, recording or otherwise) without the prior consent of BioClever.

| | n | Missing | Estimate (CI, 95%) | pvalue |
|---|---|---|---|---|
| **Model 2** | xx | xx | | |
| Covariate I* | | | xx.x (xx.x, xx.x) | x.xxxx |

* Every independent covariate at a time adjusted by group, visit day and group*visit day.

### Tabla 4.4.4 Change in D-dimer – Model 3 – final selected model

| | n | Missing | Estimate (CI, 95%) | pvalue |
|---|---|---|---|---|
| **Model 3** | xx | xx | | |
| Group: A vs B | | | xx.x (xx.x, xx.x) | x.xxxx |
| Visit day | | | xx.x (xx.x, xx.x) | x.xxxx |
| Group*visit day | | | xx.x (xx.x, xx.x) | x.xxxx |
| Covariate I | | | xx.x (xx.x, xx.x) | x.xxxx |
| … | | | xx.x (xx.x, xx.x) | x.xxxx |

### Tabla 4.4.5 Change in D-dimer – Model 4 – final model

| | n | Missing | Estimate (CI, 95%) | pvalue |
|---|---|---|---|---|
| **Model 4** | xx | xx | | |
| Group: A vs B | | | xx.x (xx.x, xx.x) | x.xxxx |
| Visit day | | | xx.x (xx.x, xx.x) | x.xxxx |
| Group*visit day | | | xx.x (xx.x, xx.x) | x.xxxx |
| Covariate I | | | xx.x (xx.x, xx.x) | x.xxxx |
| … | | | xx.x (xx.x, xx.x) | x.xxxx |

Same analysis for every inflammatory marker.

**Tabla 4.4.6 Change in Ferritin by group**
**Figure 6 Change in Ferritin by group**
**Tabla 4.4.7 Change in Ferritin – Model 1 – basic model**
**Tabla 4.4.8 Change in Ferritin – Model 2 – one covariate**
**Tabla 4.4.9 Change in Ferritin – Model 3 – final selected model**
**Tabla 4.4.10 Change in Ferritin – Model 4 – final model**

All rights reserved. No part of this material may be reproduced or copied in any form by any means (written, electronic, photocopying, recording or otherwise) without the prior consent of BioClever.

-82:1547 .7;36893 0-) ,#%"//*$ ,%%" $',' ($! &,*./,&+'%+'



**Tabla 4.4.11 Change in IL-6 by group**
**Figure 7 Change in IL6 by group**
**Tabla 4.4.12 Change in IL-6 – Model 1 – basic model**
**Tabla 4.4.13 Change in IL-6 – Model 2 – one covariate**
**Tabla 4.4.14 Change in IL-6 – Model 3 – final selected model**
**Tabla 4.4.15 Change in IL-6 – Model 4 – final model**

**Tabla 4.4.16 Change in Lynphocites by group**
**Figure 8 Change in Lynphocites by group**
**Tabla 4.4.17 Change in Lynphocites – Model 1 – basic model**
**Tabla 4.4.18 Change in Lynphocites – Model 2 – one covariate**
**Tabla 4.4.19 Change in Lynphocites – Model 3 – final selected model**
**Tabla 4.4.20 Change in Lynphocites – Model 4 – final model**

**Tabla 4.4.21 Change in PCR by group**
**Figure 9 Change in PCR by group**
**Tabla 4.4.22 Change in PCR – Model 1 – basic model**
**Tabla 4.4.23 Change in PCR – Model 2 – one covariate**
**Tabla 4.4.24 Change in PCR – Model 3 – final selected model**
**Tabla 4.4.25 Change in PCR – Model 4 – final model**

**Tabla 4.4.26 Change in Pre-albumin by group**
**Figure 10 Change in Pre-albumin by group**
**Tabla 4.4.27 Change in Pre-albumin – Model 1 – basic model**
**Tabla 4.4.28 Change in Pre-albumin – Model 2 – one covariate**
**Tabla 4.4.29 Change in Pre-albumin – Model 3 – final selected model**
**Tabla 4.4.30 Change in Pre-albumin – Model 4 – final model**

All rights reserved. No part of this material may be reproduced or copied in any form by any means (written, electronic, photocopying, recording or otherwise) without the prior consent of BioClever.

-82:1§47 .7;3§893 0-) ,#%"//*$ ,%%" $',' ($! &,*./,&+'%+'



## 9.5 Safety primary objective - SAF

**Tabla 5.1.1 General summary of adverse events – SAF**

| | A (n=xx) | | B (n=xx) | | |
|---|---|---|---|---|---|
| | **Number of patients n (%)** | **Number of events n** | **Number of patients n (%)** | **Number of events n** | **pvalue*** |
| Any adverse event (AE) | xx (xx.x%) | xx | xx (xx.x%) | xx | x.xxxx |
| Any adverse event with grade ≥4 | xx (xx.x%) | xx | xx (xx.x%) | xx | x.xxxx |
| Any serious adverse event (SAE) | xx (xx.x%) | xx | xx (xx.x%) | xx | x.xxxx |
| Unexpected Adverse Event (UAE) | xx (xx.x%) | xx | xx (xx.x%) | xx | x.xxxx |
| Any severe adverse event | xx (xx.x%) | xx | xx (xx.x%) | xx | x.xxxx |
| Suspected Unexpected Serious Adverse Reaction (SUSAR) | xx (xx.x%) | xx | xx (xx.x%) | xx | x.xxxx |
| Related to COVID-19 | xx (xx.x%) | xx | xx (xx.x%) | xx | x.xxxx |
| Related to the treatment | xx (xx.x%) | xx | xx (xx.x%) | xx | x.xxxx |

***Comparation between number of patients**

All rights reserved. No part of this material may be reproduced or copied in any form by any means (written, electronic, photocopying, recording or otherwise) without the prior consent of BioClever.

**Tabla 5.1.2 Adverse events I (A) - SAF**

| | | | | Seriousness | | Grade | | | | | Related to study medication | | Related to COVID-19 | |
|---|---|---|---|---|---|---|---|---|---|---|---|---|---|---|
| | Nº pat. (%) | Nº AE | Duration (days)* | Serious | Not Serious | Mild | Moderate | Severe | Vital risk | Death | Not Related | Related | Not Related | Related |
| **Overall** | xx (xx.x) | xx | x.x (x.x) | xx | xx | xx | xx | xx | xx | xx | xx | xx | xx | xx |
| **SOC 1** | xx (xx.x) | xx | x.x (x.x) | xx | xx | xx | xx | xx | xx | xx | xx | xx | xx | xx |
| PT 1 | xx (xx.x) | xx | x.x (x.x) | xx | xx | xx | xx | xx | xx | xx | xx | xx | xx | xx |
| PT 2 | xx (xx.x) | xx | x.x (x.x) | xx | xx | xx | xx | xx | xx | xx | xx | xx | xx | xx |
| … | | | | | | | | | | | | | | |
| **SOC 2** | xx (xx.x) | xx | x.x (x.x) | xx | xx | xx | xx | xx | xx | xx | xx | xx | xx | xx |
| PT 1 | xx (xx.x) | xx | x.x (x.x) | xx | xx | xx | xx | xx | xx | xx | xx | xx | xx | xx |
| PT 2 | xx (xx.x) | xx | x.x (x.x) | xx | xx | xx | xx | xx | xx | xx | xx | xx | xx | xx |
| … | | | | | | | | | | | | | | |
| … | | | | | | | | | | | | | | |

*SOC and PT: coding with the MedDRA dictionary

All rights reserved. No part of this material may be reproduced or copied in any form by any means (written, electronic, photocopying, recording or otherwise) without the prior consent of BioClever.

## Tabla 5.1.3 Adverse events II (A) - SAF

| | | | Expected | | Necessity for medication | | Resolution | | | | Active | |
|---|---|---|---|---|---|---|---|---|---|---|---|---|
| | Nº pat. (%) | Nº AE | Yes | No | Yes | No | Resolved | Stabilized | Basal condition | Unresolved | Yes | No |
| **Overall** | xx (xx.x) | xx | xx | xx | xx | xx | xx | xx | xx | xx | xx | xx |
| **SOC 1** | xx (xx.x) | xx | xx | xx | xx | xx | xx | xx | xx | xx | xx | xx |
| PT 1 | xx (xx.x) | xx | xx | xx | xx | xx | xx | xx | xx | xx | xx | xx |
| PT 2 | xx (xx.x) | xx | xx | xx | xx | xx | xx | xx | xx | xx | xx | xx |
| … | | | | | | | | | | | | |
| **SOC 2** | xx (xx.x) | xx | xx | xx | xx | xx | xx | xx | xx | xx | xx | xx |
| PT 1 | xx (xx.x) | xx | xx | xx | xx | xx | xx | xx | xx | xx | xx | xx |
| PT 2 | xx (xx.x) | xx | xx | xx | xx | xx | xx | xx | xx | xx | xx | xx |
| … | | | | | | | | | | | | |
| … | | | | | | | | | | | | |

All rights reserved. No part of this material may be reproduced or copied in any form by any means (written, electronic, photocopying, recording or otherwise) without the prior consent of BioClever.

## Tabla 5.1.4 Adverse events III (A) - SAF

| | | | Action taken | | | Grade change | |
|---|---|---|---|---|---|---|---|
| | Nº pat. (%) | Nº AE | None | Temporal interruption | Permanent interruption | Yes | no |
| **Overall** | xx (xx.x) | xx | xx | xx | xx | xx | xx |
| **SOC 1** | xx (xx.x) | xx | xx | xx | xx | xx | xx |
| PT 1 | xx (xx.x) | xx | xx | xx | xx | xx | xx |
| PT 2 | xx (xx.x) | xx | xx | xx | xx | xx | xx |
| … | | | | | | | |
| **SOC 2** | xx (xx.x) | xx | xx | xx | xx | xx | xx |
| PT 1 | xx (xx.x) | xx | xx | xx | xx | xx | xx |
| PT 2 | xx (xx.x) | xx | xx | xx | xx | xx | xx |
| … | | | | | | | |
| … | | | | | | | |

## Tabla 5.1.5 Adverse events I (B) - SAF

## Tabla 5.1.6 Advere events II (B) - SAF

## Tabla 5.1.7 Advere events III (B) - SAF

All rights reserved. No part of this material may be reproduced or copied in any form by any means (written, electronic, photocopying, recording or otherwise) without the prior consent of BioClever.

## 9.6 Others analysis

### 9.6.1 Worst COVID-19 WHO scale score

**Tabla 6.1.1 Worst COVID-19 WHO scale score by group**

| | | **A (n=xx)** | **B (n=xx)** |
|---|---|---|---|
| Worst COVID-19 scale score I | | | |
| Total no-missing | n | xx | xx |
| 0: Uninfected. No viral RNA detected. | n (%) | xx (xx.x%) | xx (xx.x%) |
| 1: Asymptomatic. Viral RNA detected. | n (%) | xx (xx.x%) | xx (xx.x%) |
| 2: Symptomatic. Independent. | n (%) | xx (xx.x%) | xx (xx.x%) |
| 3: Symptomatic. Assistance needed. | n (%) | xx (xx.x%) | xx (xx.x%) |
| 4: Hospitalized. No oxygen therapy. | n (%) | xx (xx.x%) | xx (xx.x%) |
| 5: Hospitalized. Oxygen by mask or nasal prongs. | n (%) | xx (xx.x%) | xx (xx.x%) |
| 6: Hospitalized. Oxygen by NIV or high flow. | n (%) | xx (xx.x%) | xx (xx.x%) |
| 7: Intubation and mechanical ventilation. $pO_2/FIO_2$ ≥150 or $SpO_2/FIO_2$ ≥ 200 | n (%) | xx (xx.x%) | xx (xx.x%) |
| 8: Mechanical ventilation $pO_2/FIO_2$< 150 ($SpO_2/FIO_2$ < 200) or vasopressors. | n (%) | xx (xx.x%) | xx (xx.x%) |
| 9: Mechanical ventilation $pO_2/FIO_2$ <150 and vasopressors, dialysis, or ECMO | n (%) | xx (xx.x%) | xx (xx.x%) |
| 10: Death. | n (%) | xx (xx.x%) | xx (xx.x%) |
| Missing | n | xx | xx |
| | pvalue | x.xxxx | |
| | | | |
| Worst COVID-19 scale score II | | | |
| Total no-missing | n | xx | xx |
| No infection (0 or 1) | n (%) | xx (xx.x%) | xx (xx.x%) |
| Mild (2 or 3) | n (%) | xx (xx.x%) | xx (xx.x%) |

All rights reserved. No part of this material may be reproduced or copied in any form by any means (written, electronic, photocopying, recording or otherwise) without the prior consent of BioClever.

| | | A (n=xx) | B (n=xx) |
|---|---|---|---|
| Moderate (4 or 5) | n (%) | xx (xx.x%) | xx (xx.x%) |
| Severe (from 6 to 9) | n (%) | xx (xx.x%) | xx (xx.x%) |
| Death (10) | n (%) | xx (xx.x%) | xx (xx.x%) |
| Missing | n | xx | xx |
| | pvalue | x.xxxx | |

**Tabla 6.1.2 Worst by proportional-odds - model 1 – basic model**

| | n | Missing | OR, 95% CI | pvalue |
|---|---|---|---|---|
| **Model** | xx | xx | | |
| Group: A vs B | | | x.xxx (x.xxx, x.xxx) | x.xxxx |

**Tabla 6.1.3 Worst by proportional-odds - model 2 – one-covariate model**

| | n | Missing | OR, 95% CI | pvalue |
|---|---|---|---|---|
| **Model** | xx | xx | | |
| Group: A vs B | | | x.xxx (x.xxx, x.xxx) | x.xxxx |
| Variable I* | | | x.xxx (x.xxx, x.xxx) | x.xxxx |

* Every independent variable at a time.

**Tabla 6.1.4 Worst by proportional-odds - model 3 – final selected model**

| | n | Missing | OR, 95% CI | pvalue |
|---|---|---|---|---|
| **Model** | xx | xx | | |
| Group: A vs B | | | x.xxx (x.xxx, x.xxx) | x.xxxx |
| Variable 1 | | | x.xxx (x.xxx, x.xxx) | x.xxxx |
| Variable 2 | | | x.xxx (x.xxx, x.xxx) | x.xxxx |
| … | | | x.xxx (x.xxx, x.xxx) | x.xxxx |

All rights reserved. No part of this material may be reproduced or copied in any form by any means (written, electronic, photocopying, recording or otherwise) without the prior consent of BioClever.

-82:1547 .7;3893 0-) ,#%"//*$ ,%%" $',' ($! &,*./,&+'%+'



**Tabla 6.1.5 Worst by proportional-odds - model 4 – final model**

| | n | Missing | OR, 95% CI | pvalue |
|---|---|---|---|---|
| **Model** | xx | xx | | |
| Group: A vs B | | | x.xxx (x.xxx, x.xxx) | x.xxxx |
| Variable 1 | | | x.xxx (x.xxx, x.xxx) | x.xxxx |
| Variable 2 | | | x.xxx (x.xxx, x.xxx) | x.xxxx |
| … | | | x.xxx (x.xxx, x.xxx) | x.xxxx |

## 9.6.2 Hospitalisation

**Tabla 6.2.1 Distribution of gender, age and days of symptom onset by hospitalisation whitin groups**

| **Group** | | **A** | | **B** | |
|---|---|---|---|---|---|
| **Hospitalisation** | | **Yes (n=xx)** | **No (n=xx)** | **Yes (n=xx)** | **No (n=xx)** |
| Gender | | | | | |
| Total no-missing | n | xx | xx | xx | xx |
| Male | n (%) | xx (xx.x%) | xx (xx.x%) | xx (xx.x%) | xx (xx.x%) |
| Female | n (%) | xx (xx.x%) | xx (xx.x%) | xx (xx.x%) | xx (xx.x%) |
| Missing | n | xx | xx | xx | xx |
| Age | n | xx | xx | xx | xx |
| | Mean (SD) | xx.x (xx.x) | xx.x (xx.x) | xx.x (xx.x) | xx.x (xx.x) |
| | Median (Q1,Q3) | xx (xx, xx) | xx (xx, xx) | xx (xx, xx) | xx (xx, xx) |
| | (Min,Max) | (xx, xx) | (xx, xx) | (xx, xx) | (xx, xx) |
| | Missing | xx | xx | xx | xx |
| Days of symptom onset at baseline visit | n | xx | xx | xx | xx |
| | Mean (SD) | xx.x (xx.x) | xx.x (xx.x) | xx.x (xx.x) | xx.x (xx.x) |

All rights reserved. No part of this material may be reproduced or copied in any form by any means (written, electronic, photocopying, recording or otherwise) without the prior consent of BioClever.

- 82 : 1 47 . 7 ; 3 893 0 - ) , #%" / / * $ , %%" $ ' , ' ( $ !  & , * . / , & + ' % + '



| **Group** | | **A** | | **B** | |
|---|---|---|---|---|---|
| **Hospitalisation** | | **Yes (n=xx)** | **No (n=xx)** | **Yes (n=xx)** | **No (n=xx)** |
| | Median (Q1,Q3) | xx (xx, xx) | xx (xx, xx) | xx (xx, xx) | xx (xx, xx) |
| | (Min,Max) | (xx, xx) | (xx, xx) | (xx, xx) | (xx, xx) |
| | Missing | xx | xx | xx | xx |

All rights reserved. No part of this material may be reproduced or copied in any form by any means (written, electronic, photocopying, recording or otherwise) without the prior consent of BioClever.

**This supplement on the CoV-Early protocol contains the following items:**

1. PROTOCOL: Original and last amendment (nr. 4) with a summary of changes
   - Page 2 CoV-Early Original protocol (version 2.0, 22th September 2020)
   - Page 56 CoV-Early final protocol (version 6.0, Amendement 4, 1th May 2021)
   - Page 112 Summary of changes

2. Statistical analysis plan : The original and final SAP are incorporated in the original protocol (paragraph 10) and final amendment 4 (paragraph 10) of the protocol
   - Page 115 Summary of changes of the SAP

# Early Convalescent Plasma Therapy for high-risk patients with COVID-19 in primary care (the CoV-*Early* Study)

## *A randomized clinical trial*

**PROTOCOL**

| | | |
|---|---|---|
| **Version** | **:** | **2.0** |
| **Date** | **:** | **22 September 2020** |
| **Principal investigator EMC** | **:** | **Bart Rijnders and Casper Rokx** |
| **Principal investigator LUMC** | **:** | **Jaap Jan Zwaginga** |
| **Sponsor** | **:** | **Erasmus MC** |
| **Funder** | **:** | **ZONMW** |
| **Clinical research organization** | **:** | **HOVON Data Center** |
| **ID** | **:** | **NL74972.078.20** |

| Responsibility | Name | Affiliation/Address |
|---|---|---|
| Principal Investigators | Bart Rijnders and Casper Rokx<br><br>Jaap Jan Zwaginga | Erasmus MC<br><br>LUMC |
| Coordinating investigator | Bart Rijnders | Erasmus MC |
| Sponsor | Erasmus MC | |
| Subsidising party | ZONMW | |
| Independent expert | Dr. J.L.Nouwen | Erasmus MC |
| Co-investigators | Arvind Gharbharan<br>Carlijn Jordans<br>Casper Rokx<br>Mischa Huson<br>Yvonne Muller<br>Peter Katsikis<br>Jelle Miedema<br>Menno van der Eerden<br>Pierluigi Ciet<br>Marion Koopmans<br>Corine Geurtsvankessel<br>J.G. van der Bom<br>S.P. Mooijaart<br>G.H. Groeneveld<br>C. Wife<br>Romy Meier<br>M.J. van Wordragen-Vlaswinkel<br>W.E. Benckhuijsen<br>Francis Swaneveld<br>Ellen van der Schoot | Erasmus MC<br>Erasmus MC<br>Erasmus MC<br>Erasmus MC<br>Erasmus MC<br>Erasmus MC<br>Erasmus MC<br>Erasmus MC<br>Erasmus MC<br>Erasmus MC<br>Erasmus MC<br>LUMC<br>LUMC<br>LUMC<br>LUMC<br>LUMC<br>LUMC<br>LUMC<br>Sanquin<br>Sanquin |
| Statisticians | Dr. Nan van Geloven<br>Dr. Erik van Zwet<br>Grigorios Papageorgiou | LUMC<br>LUMC<br>Erasmus MC |
| Monitoring | Nephrology research unit | Erasmus MC |

| | | |
|---|---|---|
| Serious Adverse Events (SAEs) notification | HOVON Safety Desk<br>Bart Rijnders, Casper Rokx | HOVON Data Center<br>Erasmus MC |

| | |
|---|---|
| Collaborators at Erasmus MC | Dept of Internal Medicine and Infectious Diseases<br>Dr. Casper Rokx and dr. Mischa Huson<br><br>Dept. Of Immunology<br>Prof.dr. Peter Katsikis and dr. Yvonne Muller<br><br>Dept of pulmonary Medicine<br>Drs Jelle Miedema and dr. Menno van der Eerden<br><br>Dept. Of Virosciences<br>Dr. Corine GeurtsvanKessel and prof.dr Marion Koopmans<br><br>Dept. Of Biostatistics<br>Drs. Grigorios Papageorgiou<br><br>Dept. of Radiology and Nuclear Medicine<br>Dr. Pierluigi Ciet |
| Collaborators at LUMC | Dept. of Biomedical Data Sciences<br>Dr Nan van Geloven<br>Dr Erik van Zwet<br><br>Dept. of Clinical Epidemiology<br>Prof. dr. J.G. van der Bom<br><br>Dept of Internal Medicine, subdepartment of Gerontology and Geriatrics<br>Dr. S.P. Mooijaart<br><br>Dept. of Infectious disease and/or acute internal medicine<br>Dr. G.H. Groeneveld |
| Collaborators at Sanquin | Drs. Francis Swaneveld<br>Prof. dr. Ellen van der Schoot |
| Other Collaborators | Mw. prof.dr. J. Gussekloo<br>Dept. of Public Health and Primary Care, LUMC<br>Prof. dr. T.J.M. Verheij<br>Julius Center for Health Sciences and Primary Care, UMCU |
| Study sites and PI | 1. Erasmus MC – Bart Rijnders/Casper Rokx<br>2. LUMC Jaap Jan Zwaginga |

| | |
|---|---|
| | 3. Martini Groningen – Imro Vlasveld<br>4. Reinier de Graaf – Eduardus Posthuma<br>5. Haaglanden MC – Femke Mollema<br>6. Rijnstate Arnhem – Robert-Jan Hassing<br>7. Catherina Ziekenhuis Eindhoven – Heidi Ammerlaan<br>8. OLVG – Janneke Stalenhoef<br>9. Maasstadziekenhuis – Jan den Hollander<br>10. Groene Hart Ziekenhuis – Faiz Karim<br>11. Spaarne Gasthuis – Robin Soetekouw<br>12. Antonius Nieuwegein – Elena Monica van Leeuwen-Sergarceanu<br>13. Amphia Ziekenhuis Breda, Ronald van Etten<br>14. Medisch centrum Leeuwarden, Linda Kampschreur<br>15. Ziekenhuis Bernhoven, Inge Ludwig<br>16. Meander Medisch Centrum, Eefje Jong<br>17. UMCG, Douwe Postma<br>18. Decision regarding 7 more sites is pending |

## Scheme of study

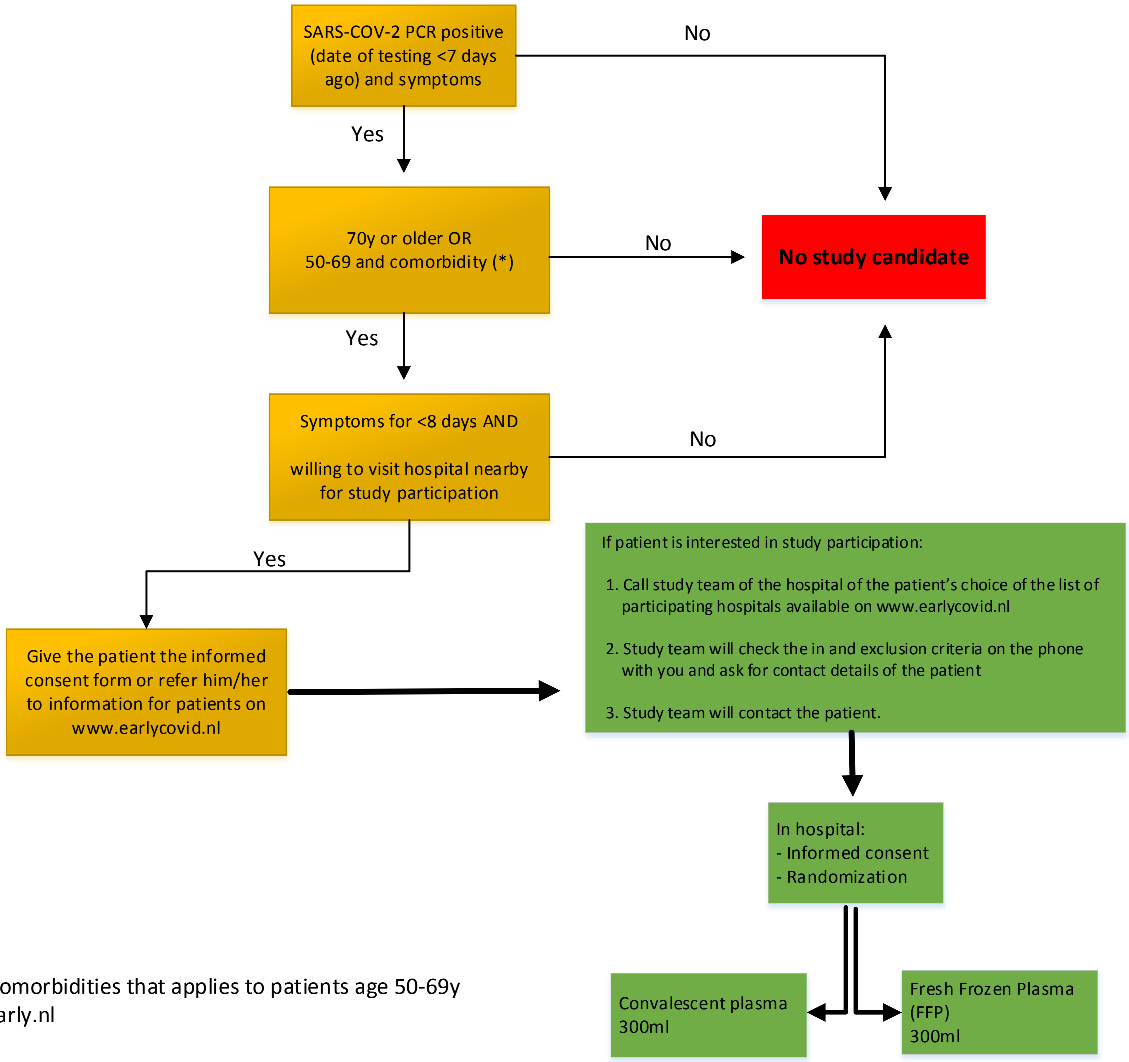


(*) For the list of comorbidities that applies to patients age 50-69y see www.covearly.nl

# TABLE OF CONTENTS

Page

**1 INTRODUCTION AND RATIONALE ... 15**

**2 STUDY OBJECTIVES ... 17**

2.1 Primary objectives ... 17
2.2 Secondary (exploratory) objectives ... 17

**3 STUDY DESIGN ... 18**

**4 STUDY POPULATION ... 19**

4.1 Population base ... 19
4.2 Inclusion criteria ... 19
4.3 Exclusion criteria ... 20
4.4 Sample size calculation ... 20

**5 TREATMENT ... 20**

5.1 Intervention: infusion of plasma ... 20
5.2 Use of co-intervention (if applicable) ... 22
5.3 Escape medication (if applicable) ... 22

**6 PLASMA RETRIEVAL, ADMINISTRATION AND DETERMINATION OF SARS-COV-2 ANTIBODIES .. 22**

6.1 Assessment of SARS-CoV-2 antibodies in donor serum ... 22

**7 NON-INVESTIGATIONAL PRODUCT ... 23**

**8 METHODS ... 23**

8.1 Endpoints ... 23
8.1.1 Primary endpoint ... 23
8.1.2 Secondary endpoints ... 24
8.1.3 Exploratory endpoints: ... 25
8.2 Randomisation, blinding and allocation ... 25
8.2.1 Regulatory documentation ... 25
8.2.2 Registration and randomization ... 25
8.2.3 Unblinding procedures ... 25
8.3 Study procedures ... 26
8.3.1 Time of clinical evaluations ... 26
8.3.2 Required investigations ... 26
8.3.3 Specification of required investigations ... 29
8.4 Withdrawal of individual patients from protocol treatment ... 30
8.5 Replacemnts of individual subjects after withdrawal ... 30
8.6 Follow up of patients withdrawn from protocol treatment ... 30
8.7 Premature termination of the study ... 30
8.8 Data collection ... 31
8.8.1 Rapid reporting ... 31

**9 SAFETY ... 32**

9.1 Temporary halt for reasons of subject safety ... 32
9.2 AEs and SAEs ... 32
9.2.1 Adverse events (AEs) ... 32
9.2.2 Serious adverse events (SAEs) ... 32
9.2.3 Suspected unexpected serious adverse reactions (SUSARs) ... 33
9.3 Annual safety report ... 33
9.4 Follow-up of adverse events ... 34
9.5 Data Safety Monitoring Board (DSMB) ... 34

**10 STATISTICAL CONSIDERATIONS ... 35**

10.1 Patient numbers and power considerations ..... 35
10.2 Primary endpoint analysis ..... 36
10.3 Secondary endpoint analysis ..... 36
10.4 Exploratory endpoints: ..... 38
10.5 Interim efficacy and safety analysis ..... 38
10.6 Stopping rules ..... 38

**11 ETHICAL CONSIDERATIONS ..... 39**

11.1 Regulations statement ..... 39
11.2 Recruitment and consent ..... 39
11.3 Objection by minors or incapacitated subjects ..... 40
11.4 Benefits and risks assessment ..... 40
11.5 Compensation for injury ..... 41
11.6 Incentives ..... 41

**12 ADMINISTRATIVE ASPECTS AND PUBLICATION ..... 41**

12.1 Handling and storage of data and documents ..... 41
12.1.1 Patient confidentiality ..... 41
12.1.2 Filing of essential documents ..... 42
12.1.3 Record retention ..... 42
12.1.4 Storage and sharing of data ..... 42
12.1.5 Storage of samples ..... 42
12.2 Monitoring and quality insurance ..... 43
12.3 Amendments ..... 43
12.4 Annual progress report ..... 44
12.5 Temporary halt and (prematurely) end of trial report ..... 44
12.6 Public disclosure and publication policy ..... 44

**13 STRUCTURED RISK ANALYSIS ..... 44**

13.1 Potential issues of concern ..... 44
13.2 Synthesis ..... 46

**14 REFERENCES ..... 47**

**A. RISK FACTORS FOR SEVERE COVID-19 ..... 49**

**B. RIVM DATA ..... 50**

**C. LABELING OF PLASMA ..... 51**

**D. COMMON TERMINOLOGY CRITERIA FOR ADVERSE EVENTS ..... 52**

**E. DONOR CRITERIA USED BY SANQUIN ..... 52**

**F. HEMOVIGILANCE ..... 53**

## LIST OF ABBREVIATIONS AND RELEVANT DEFINITIONS

A(D)R: Adverse (Drug) Reaction
ADCC: Antibody-Dependent Cell-mediated Cytotoxicity
ADE: Antibody-mediated enhancement of infection
AE: Adverse Event/Adverse Experience
ARDS: adult respiratory distress syndrome
BAL: Broncho Alveolar Lavage
CA: Competent Authority
CDC: United States Centers for Disease Control and Prevention
COI: Conflict of Interest
ConvP: Convalescent Plasma
COPD: Chronic obstructive pulmonary disease
COVID-19: Coronavirus Disease 2019
CRF: Case Report Form
CRP: C-reactive Protein
CTCAE: Common Terminology Criteria for Adverse Events
DMC: Data Management Center
DSMB: Data and Safety Monitoring Board
EUA: Emergency Use Authorization
ECG: Electrocardiogram
FFP: fresh frozen plasma
GCP: Good Clinical Practice
GDPR: General Data Protection Regulation
HAV: Hepatitis A virus
HBV: Hepatitis B virus
HCV: Hepatitis C virus
HIV: Human immunodeficiency virus
HTLV: Human T-cell lymphotropic virus
IC(F): Informed Consent (Form)
ICH: International Conference on Harmonization
ICU: Intensive Care Unit
IMP(D): Investigational Medicinal Product (Dossier)
IRB: Institutional review board
ISBT: International Society of Blood Transfusion
ISM: Independent Safety Monitor
IWRS :Interactive web response system
LDH: Lactate Dehydrogenase
LOS: Length of Stay
MERS: Middle East Respiratory Syndrome
METC: Medical Ethical Review Committee
MOF: mult Organ Failure
NA: Nuclear antibody
NP: Nasopharyngeal
NYHA: New York Heart Association
OP: Oropharyngeal
OS: Overall Survival
RT-PCR: Reverse Transcriptase Real-Time Polymerase chain reaction
PB: Peripheral Blood
PK: Pharmacokinetic
PRTN-50: The concentration of serum to reduce the number of plaques by 50% compared to the serum free virus
QP: Qualified person
SAE: Serious adverse event

SARS: Severe Acute Respiratory Syndrome
SARS-CoV-2: Severe Acute Respiratory Syndrome Coronavirus 2
SC: Sub-Cutaneous
SD: Stable Disease
SOFA: sequential organ failure assessment
SOP: Standard Operating Procedure
SUSAR: Suspected unexpected seriouw adverse reaction
TACO: Transfusion-associated circulatory overload
TRALI: Transfusion-related acute lung injury
UIP: Usual Interstitial Pneumonia
WHO: World Health Organisation
WMO: Wet Medisch-Wetenschappelijk Onderzoek met mensen

**Enrolled:** patients consented to participate until designated as a screen failure or until they discontinued the study or completed it.

**Randomized:** when a study plasma is ordered and released from the institutional blood bank for a patient or a patient is determined as control

**Screen Failures:** referred for inclusion, but then determined to be ineligible or withdraws before being enrolled

**Discontinued:** randomized having ordered for (or received) convalescent plasma or not (control), but then withdrawn by investigator or withdrawn consent

**Completed:** Subjects are considered to have completed when they are followed through day 28 or have had an adverse event or death occurred prior to day 28.

## SUMMARY

| | |
|---|---|
| **Rationale** | An effective, widely available, and safe treatment that can decrease the duration, severity and fatality of COVID-19 is urgently needed. Also, in the most affected regions the pressure on health care systems including ventilator support capacity can be a limiting factor for survival. Initial studies including from our group indicate that administering convalescent plasma containing high titers of neutralizing antibodies to COVID-19 patients who are already relatively late during the disease course after hospital admission is not effective, which can be explained by high titers of autologous antibodies present in patients. Thus, the antiviral capacity of convalescent plasma is hypothesized to be best postioned early in the disease course and in patients at increased risk for a severe disease course. If effective, any treatment that decreases the need for hospital admission is very valuable but so far, no COVID-19 treatment has been shown to prevent clinical deterioration when given before patients are admitted to the hospital. |
| **Study objectives** | **Primary objectives**<br>♦ To evaluate the efficacy, feasibility and safety following the administration of convalescent plasma (ConvP) as a therapy for outpatients diagnosed with COVID-19 at increased risk for an unfavourable clinical outcome and within 7 days after symptom onset.<br><br>**Secondary (exploratory) objectives**<br>♦ To evaluate the impact of 300mL convP on mortality<br>♦ To evaluate the impact of 300mL convP on hospital admission<br>♦ To evaluate the impact of 300 mL convP on admission to ICU |

| | |
|---|---|
| | ♦ To evaluate the impact of 300mL convP on duration of symptoms<br>♦ To evaluate the impact of 300mL convP in relation to the age and clinical frailty of the patient |
| **Study design** | This trial is a nationwide multicenter, double blind, randomized controlled trial in the Netherlands. Patients will be randomized between the transfusion of 300mL of convP versus regular fresh frozen plasma (FFP). |
| **Patient population** | Patients with PCR confirmed COVID disease with less than 8 days of symptoms, age 70 or older or 50-69 years with at least 1 additional risk factor for severe COVID-19 are eligible. A total of 690 patients will be included.<br>Expected duration of accrual: 18-24 months |
| **Intervention** | 300mL of convP with a minimum level of neutralizing antibodies (see chapter 6).<br>Duration of follow up :Day 28 for the primary endpoint |
| **Main study endpoints** | **Primary endpoints**<br>♦ Highest disease status on the 5-point ordinal disease severity scale in the 28 days following transfusion of convP versus FFP.<br>**Secondary (exploratory)endpoints**<br>♦ Number (%) of deaths in the 28 days following transfusion of convP versus FFP<br>♦ Number (%) of hospital admissions in the 28 days following transfusion of convP versus FFP<br>♦ Number (%) of ICU admissions in the 28 days following transfusion of convP versus FFP<br>♦ Disease duration in days of symptoms in the 28 days following transfusion of convP versus FFP<br>♦ Age and clinical frailty scale stratified analysis of number of primary outcome. |

| | |
|---|---|
| Benefit and nature and extent of the burden and risks associated with participation | Benefits of this study may include a shorter disease course, a lower hospital admission rate and a decrease in mortality. In elderly patients, earlier recovery may also translate into being less frail after recovery. On the other hand, possible disease excacerbation by the antibodies has also been suggested. A blinded and randomized approach comparing FFP with convalescent plasma is therefore chosen, yielding unbiassed assessment of all endpoints. The risks of plasma infusion are small and widely known. These include transfusion reactions, transfusion related acute lung injury, circulatory overload and transmission of (unkown) transmittable diseases. |
| Planned interim analysis and DSMB | Continuous monitoring of the primary endpoint (every 50 patients or 3 months) with reporting to the DSMB in case of high posterior probabilities of either a positive effect (>97.5%) or a negative effect (>80%). |

# 1 Introduction and rationale

COVID-19 is causing substantial morbidity and mortality worldwide. Older people and people with comorbidities are most affected, and the ones most likely to be admitted to the hospital and to die of COVID-19. Despite this, intervention trials that focus on patients early on in the disease course, before hospital admission and in which a substantial number of older patients are included are very scarce. However, a treatment that turns out to be effective in the outpatient setting could result in a reduced hospital admission rate of the group of patients most affected and decrease the pressure on hospital and ICU beds. Given that patients with risk factors for admission also represent the ones who, once admitted to the ICU, will stay there for an average of 3 weeks, any intervention that prevents disease progression substantially will result in a major relieve of scarce healthcare systems during this pandemic.

A recently completed clinical trial demonstrated that *anti-inflammatory* therapy with dexamethasone significantly decreases overall mortality.[1] However, no effect was observed in patients not requiring supplemental oxygen therapy so the intervention is unlikely to be effective in the outpatient setting. In a randomized trial a shortened time to clinical recovery in patients treated with remdesivir was observed and comparable results were observed in a trial on interferon beta-1b, lopinavir–ritonavir, and ribavirin.[2] However, it remains to be seen if any of these antiviral therapies will decrease mortality and none of the interventions have been tested, or feasible to administer, in the outpatient setting.[3,4] Also, these drugs are not widely available and the rapid distribution to hospitals across the world is extremely challenging.[5] Therefore, other readily available, affordable and effective antiviral therapies are needed.

Convalescent plasma (ConvP), that contains virus neutralizing antibodies, could be an alternative treatment option for SARS-CoV-2 patients. A similar strategy has been pursued during the 2003 SARS and later MERS outbreaks.[6] Conclusive evidence for the effectivity of ConvP as a treatment for human coronavirus infections has yet not been documented in large randomized clinical trials. Preclinical research however indicated a protective effect of *hamster* ConvP when given to hamsters infected with SARS-CoV-2 early in the disease course. More recently *human* ConvP was shown to protect hamsters from COVID-19 disease as long as plasma with a high neutralizing antibody titer was used.[7] (B Rockx, personal communication) Although large volumes of ConvP can have indirect effects as well, we assume that key to the efficacy of ConvP through direct antiviral effect is the presence of high titers of virus neutralizing antibodies. Following this rationale, benefit can only be expected if it is administered to patients with as yet little or no autologous neutralizing antibodies. Although ConvP seems to be safe, no overall clinical benefit of ConvP therapy was observed in a prematurely interrupted randomized trial from China. That study though, was clearly too small to draw

definite conclusions.[8,9] Moreover, in this study, ConvP was administered extremely late in the disease course as patients had been symptomatic for 30 days on average when they received ConvP. The recent observations showing that close to 100% of patients have detectable neutralizing antibodies three weeks after symptom onset may explain the lack of a therapeutic effect in late disease.[10,11] All other evidence on the possible efficacy of ConvP comes from uncontrolled case series or with historical patients as comparator. [12-14] The Dutch ConCOVID trial, a randomized clinical trial that aimed to study the effect of 300ml of ConvP for hospitalized patients showed that already 80% of the patients had autologous neutralizing antibodies at the time of inclusion in the study. This was observed despite the fact that patients had been symptomatic for only 10 days. Not unexpectedly, patients who were still antibody negative at study inclusion had been sick for a shorter time (6 days) compared with antibody positive patients (10 days, $p<0.001$). These observations led to the discontinuation of the ConCOVID trial when 86 patients had been enrolled as the study team and DSMB considered it too unlikely that a large therapeutic effect would be observed after enrollment of all 426 planned patients[15]. In the ConCOVID trial ConvP was obtained from patients who had recovered from a RT-PCR confirmed COVID-19 for at least 2 weeks. As plasma with the highest neutralizing antibody titer available was used for each newly enrolled patient, the median neutralizing antibody titer in the ConvP that was administered was as high as 1:640, a titer observed in approximately 20% of the 115 donors who were tested. As the Dutch blood bank (Sanquin Blood Supply) has already collected ConvP from more than 1000 donors, the stock of ConvP with such high titers of neutralizing antibodies should therefore be large enough to allow for a large randomized clinical trial. Moreover, each high titer donor can donate up to 8 units of 300ml plasma in 4 weeks.

However, the decision to end the ConCOVID study does not mean that ConvP cannot have a small but clinically relevant therapeutic effect also for hospitalized patients. Clinically relevant differences in the characteristics of the early autologous antibodies in patients and the antibodies present in donor plasma may indeed exist. To this regard, it was shown that the level of fucosylation of anti-SARS-CoV2 antibodies in ConvP is higher than antibodies present during COVID-19 disease. Antibodies with low level of Fc-fucosylation have 50x fold higher affinity for FcyRIIIa on macrophages and therefore induce much higher cytokine production in these macrophages. ICU patients were found to have significantly higher levels of afucosylated anti-SARS-CoV2 antibodies (anti-spik antibodies) than the antibodies present in convalescent plasmas.[16] Theoretically, if the highly fucosylated donor antibodies outcompete the endogeneous antibodies they could induce a negative feedback on the cytokine storm. Two very large randomized trials continue to study ConvP for hospitalized patients and will be able to detect small therapeutic effects as well (NCT02735707, NTC04381936). Also, a real-time meta-analysis of ongoing and prematurally discontinued clinical trials in the USA, Europe and India has been initiated as well and could help in finding small but still clinically relevant effects of ConvP in hospitalized patients.[17]

The insights described above led to the design of the CoV-Early study described below where the principle aim is to employ ConvP in an outpatient setting early in the disease course to patients unlikely to have developed autologous virus neutralizing antibodies and study its potency to prevent deterioration and improve clinical condition. ConvP will specifically be targeted at a population at increased risk for a severe disease course, where elderly will be overrepresented and the effectiveness of convP might even be more pronounced to prevent disability and death.

# 2 Study objectives

## 2.1 Primary objectives

To evaluate the efficacy, feasibility and safety following the administration of ConvP as a therapy for outpatients diagnosed with COVID-19 at increased risk for an unfavourable clinical outcome and within 7 days after symptom onset.

**Efficacy:**
To determine if ConvP prevents COVID-19 disease progression when it is given early in the disease course.
To evaluate whether the use of ConvP has an impact on the long-term daily functioning, pulmonary condition, cellular and humoral immunity against SARS-CoV-2.

**Feasibility:**
To evaluate the feasibility of ConvP administration in the outpatient setting with its associated challenges (e.g. performing appropriate testing, infection prevention measures required, transportation of often frail and older patient to the hospital for plasma transfusion)

**Safety:**
To evaluate the safety of ConvP as compared with FFP administration regarding transfusion related reactions (in particular TRALI and TACO) and possible unwanted side effects of virus specific antibodies present in ConvP.

## 2.2 Secondary (exploratory) objectives

Evaluated in all patients:

- To evaluate the impact of 300mL convP on mortality
- To evaluate the impact of 300mL convP on hospital admission

- ♦ To evaluate the impact of 300 mL convP on admission to ICU
- ♦ To evaluate the impact of 300mL convP on duration of symptoms
- ♦ To evaluate the impact of 300mL convP in relation to the age and clinical frailty of the patient

Evaluated in subgroups of patients:

- ♦ To evaluate the impact of 300 mL convP on functional decline in patients aged 70 or older
- ♦ To evaluate the impact of 300 mL convP on the pulmonary condition and daily functioning
- ♦ To evaluate the duration of viral shedding in patients with and without convp and according to the presence of neutralizing antibodies at baseline
- ♦ To evaluate the impact of convP on the primary outcome in patients with and without neutralizing antibodies at baseline
- ♦ To evaluate the kinetics of infection and development of cellular and humoral anti-SARS-CoV-2 immune responses including memory immunity development.
- ♦ To evaluate the difference in efficacy of convP in relation to the duration of symptoms
- ♦ To evaluate the feasibility of recruiting COVID-19 patients, administering convP and perform study follow-up in an outpatient setting
- ♦ To evaluate cost-effectiveness of convP in an outpatient setting compared to routine care

# 3 Study design

Nationwide multicentre 1:1 randomized double-blind clinical trial to compare the efficacy and safety of ConvP compared to regular FFP in COVID-19 patients considered at high risk for disease progression but in whom symptoms have started less than 8 days before the patient is enrolled in the study.

Patients and investigators will be blinded for the intervention. The most straightforward masked placebo was considered FFP. It has the same color and temperature (plasma is thawed before use) as ConvP. The different labelling of ConvP and regular plasma will be masked by the local transfusion lab personal with the use of an opaque bag wrapped around the plasma bag.

Randomization will be done via a web-based system (ALEA) provided by HOVON. In this system the investigator can randomize the patient while the result of the randomization will not be disclosed to the investigator but to a designated unblinded person from the transfusion lab in the hospital.

The primary endpoint is the highest disease status on the 5-point ordinal disease severity scale in the 28 days following transfusion of convP versus FFP. The study intervention is anticipated to improve the score on the 5-point ordinal scale (i.e. result in a shift from the higher towards the lower

categories). In particular, the intervention is expected to lower the risk of a hospital admission, need for mechanical ventilation and death by 33%, translating in an odds ratio of 0.63 which we also assume for the shift in the other categories. More information is provided in chapter 8.1 and 10 below.

Disease status is measured with a 5-point ordinal scale in which
1 = Fully recovered (no symptoms) within 7 days after transfusion
2 = Continued symptoms attributable to COVID-19 on day 7 after transfusion
3 = Admitted to hospital but no invasive ventilation needed
4 = Admitted to hospital and invasive ventilation needed
5 = Death

# 4 Study population

## 4.1 Population base

Eligible patients are adults 50 years of age or older, with symptomatic COVID-19 disease for less then 8 days and have a high risk of disease progression as defined below.

Patients will be informed on the possibility of a referral for study participation by any physician aware of the study who is a treating physician of the patient. This will almost always be the general practitioner (GP) of the patient because outpatients in the Netherlands are typically tested by the GGD “teststraat” or by the GP him/herself. All positive test results of a SARS-CoV-2 test performed by the GGD are communicated to the patients and also to the patient’s GP as a standard of care procedure.

If the patient considers to participate and gives their physician the permission to inform the study team, the GP or another physician will inform the study team and the patient will be contacted by the study team, that provides further information, checks inclusion and exclusion criteria and if the patient is still interested invites the patient to the nearest participating hospital/site. In the hospital, written informed consent will be obtained. A minimum of 10 GP practices will be approached to provide feedback on the feasibility of this recruitment method.

## 4.2 Inclusion criteria

In order to be eligible to participate in this study, a subject must meet all of the following criteria:

- ♦ RT-PCR-confirmed COVID-19

- Symptomatic (e.g but not limited to fatigue, fever, cough, dyspnoe, loss of taste or smell, diarrhea, falls or confusion)
- 70 years or older OR 50-69 years and 1 or more of the risk factors described in Appendix A

The risk factors for a bad outcome of COVID-19 are described in Appendix A are based on results described in several recent observational studies[18-21]

### 4.3 Exclusion criteria

A potential subject who meets any of the following criteria will be excluded from participation in this study

- Life expectancy <28 days in the opinion of the treating physician
- Patient or legal representative is unable to provide written informed consent
- Symptomatic for 8 days or more
- Being admitted to the hospital at the informed consent procedure
- Known previous history of transfusion-related acute lung injury
- Known IgA deficiency

### 4.4 Sample size calculation

See 10.1

## 5 Treatment

### 5.1 Intervention: infusion of plasma

Convalescent plasma will be infused that was collected from donors with a history of PCR proven symptomatic COVID-19 and who have recovered from COVID-19 for at least 14 days. The donors are recruited by Sanquin and will have to fulfill the standard plasma donor criteria used by Sanquin (Appendix E). Furthermore, only plasma from donors in whom a sufficiently high neutralizing antibody titer can be demonstrated will be used (see 6.1).

For all patients, all aspects considered necessary within routine care should be conducted before during and after the intervention.

Patients will be randomized with the use of blocks with variable length (block sizes up to 12 [2 4 6 8 10 12] into a group of

- 345 patients who will receive 300ml of thawed convP

- 345 patients who will receive 300ml of thawed non-convalescent plasma (FFP = fresh frozen plasma)

The investigator nor the patient will be informed about the treatment arm the patient was allocated to. Plasma will be administered according to the standard operating procedures of the hospital regarding the administration of blood products.

**Blood group compatibility of plasma:**

Plasma shall be transfused preferably ABO-identical. If the study plasma inventory does not hold an ABO identical then ABO-compatible plasma is acceptable. ABO compatibility is according to the following table:

*Table 6: Transfusion therapy with plasma: selection of the ABO phenotype of units to transfuse*

| ABO PHENOTYPE OF THE RECIPIENT | ABO PHENOTYPE OF UNITS TO TRANSFUSE (IN ORDER OF PREFERENCE) |
|---|---|
| O | O, A, B, AB |
| A | A, AB |
| B | B, AB |
| AB | AB |

Therefore, adherence to timing of study plasma transfusion according to the protocol has priority over ABO-identical transfusions.

Grade 1-2 allergic reactions are the most common possible side effects of plasma and are allowed according to institutional guidelines to be prevented or treated with antihistamins.

Risk based prevention or treatment of TACO is obligatory e.g. by treating pre-existing volume overload; additional administration of diuretics and or applying lower transfusion rates is allowed.

All plasma transfusions will be started after standard institutional identification of unit and patient. Similarly, after start of administration monitoring of vital signs and presence of care-personel need to be in place as are standard in Dutch hospitals and as described in Dutch Blood Transfusion guidelines.

### 5.2 Use of co-intervention (if applicable)

NOT APPLICABLE

### 5.3 Escape medication (if applicable)

NOT APPLICABLE

## 6 Plasma retrieval, administration and determination of SARS-CoV-2 antibodies

ConvP will be retrieved from donors at Sanquin Blood Supply according to their standard procedures for collection of fresh frozen plasma. Each donation 600 ml plasma will be collected in 2 bags of 300ml each. The donor will be asked (but is not obliged) to give plasma multiple times with at least a one-week interval. Plasma will be stored at minus 25 degrees Celsius or colder and tested for pathogens during routine procedures. There will be no pathogen reduction procedure. In contrast to FFP there is no quarantaine period before release of the Conv-P.

Anti-COVID-19 plasma has its own product code: E9740 = Apheresis CONVALESCENT PLASMA|Citrate/XX/≤-18°C|COVID-19. The two products of each donation will receive product codes E9740VA0 and E9740VB0 and will be labelled according to ISBT128 (see appendix C).
Before delivery of the anti-COVID-19 plasma to the participating sites by Sanquin Blood Supply an informed consent regarding a magisterial blood product (‘Bewustzijnsverklaring magistraal bloedproduct’) will be signed by the ordering physician.

ConvP will be labeled according to appendix C. Every plasma unit will have a unique identification number, a so-called EIN (Eenheid Identificatie Nummer), by which the product can always be traced back to the donor. When plasma is administered, this number will be registered in the patient file.

### 6.1 Assessment of SARS-CoV-2 antibodies in donor serum

Sanquin will select convalescent plasma with a quantitative IgG ELISA that has been shown to correlate with virus neutralizing antibody titers (plaque reduction neutralization test, PRNT; gold standard for detection of neutralizing antibodies). The ELISA threshold will be set at 60 AU that is predictive for a PNTR50 titer above 1:160 according to the method developed by the RIVM laboratory (a test calibrated in the European HORIZON2020 project Support-E). The neutralizing antibody titers will be measured on all convP that were selected based on the 60 AU ELISA screening criterium.

The PRNT50 assay will be performed at the RIVM and/or at viroscience lab in Erasmus MC. The department of virology at Erasmus MC has developed its own method for PRNT50 measurement. This PRNT test is done with the SARS-CoV-2 virus (German isolate; GISAID ID EPI_ISL 406862; European Virus Archive Global #026V-03883) and the methods were described previously.[10]

The RIVM and Viroscience methods have been compared and this comparison showed that the titers observed with the RIVM method are approximately 1 dilution lower than titers observed with the viroscience method. Therefore, only those plasma donors with a titer of 1/320 (Viroscience method) or 1/160 (Sanquin method) will be used.

Of each individual plasma also the antibodies against total spike protein and nucleocapsid will be quantified,[22] as well as the isotype (IgG, IgA and IgM) and subclass (IgG1, IgG3) will be quantified.

When the results of the phase 1 pharmacokinetics study become available, the data will be discussed within the entire study team. If the study team considers it likely that a change in the antibody titer cut-off is needed, a proposal of change will be discussed with the DSMB.

# 7 Non-investigational product

NOT APPLICABLE

# 8 Methods

## 8.1 Endpoints

### 8.1.1 Primary endpoint

- Highest disease status on the 5-point ordinal disease severity scale in the 28 days following transfusion of convP versus FFP.

  Disease status is measured with a 5-point ordinal scale in which
  1 = Fully recovered (no symptoms) within 7 days after transfusion
  2 = Continued symptoms attributable to COVID-19 on day 7 after transfusion
  3 = Admitted to hospital but no invasive ventilation needed
  4 = Admitted to hospital and invasive ventilation needed
  5 = Death

**Rationale behind primary endpoint:**

The CoV-Early primary efficacy endpoint is an ordinal outcome with the 5 categories as described above and evaluated on day 28. These endpoints encompass in our opininon the most relevant outcomes for patients infected with COVID-19, namely being sick as short as possible, not needing hospitalization, or when hospitalization is needed whether or not admitted to the ICU for mechanical ventilation is needed as well as the survival status one month after inclusion in the trial.

### 8.1.2 Secondary endpoints

*Evaluated in all patients:*

- Number (%) of deaths in the 28 days following transfusion of convP versus FFP.
- Number (%) of hospital admissions in the 28 days following transfusion of convP versus FFP
- Number (%) of ICU admissions in the 28 days following transfusion of convP versus FFP
- Disease duration in days of symptoms in the 28 days following transfusion of convP versus FFP
- Age and clinical frailty score stratified analysis of number (%) of primary endpoint following transfusion of convP versus FFP.

*Evaluated in subgroups of patients in which these data can be captured.*

Please note that no predefined sample size is in place for these subgroup analyses because patients and hospitals will be allowed to opt-in or opt-out for the substudies in which these data will be collected.

- Change in functional decline in patients over 70 years between inclusion, day 28 and month 6 following transfusion of convP versus FFP
- Change in functional respiratory imaging, FVC and DLCOc, validated QoL questionnaires between day 28, month 3, 6 and 12 following transfusion of convP versus FFP
- Change in proportion of detectable SARS-CoV-2 RT-PCR results at day 3, 7, 14 and 28 following transfusion of convP versus FFP.
- Change in number (%) of anti-SARS-CoV-2 specific B-cell and CTL memory responses at d1, d14, d28, m3, m6, m12 followin transfusion of convP versus FFP
- Number (%) of patients who fulfill the in- and exclusion criteria, number (%) of patients asked to participate in the study, number (%) of patients who do and do not participate and reasons to decline participation.
- Cost-effectiveness of convP compared to FFP will be assessed by calculating the mean costs of the intervention in relation to the relative healthcare savings of convP compared to FFP.

#### 8.1.3 Exploratory endpoints:

- ♦ Analysis of primary endpoint following transfusion of convP versus FFP stratified by the presence of neutralizing antibodies at baseline and by symptom duration at baseline.
- ♦ Change in proportion of detectable SARS-CoV-2 RT-PCR results at day 3, 7, 14 and 28 following transfusion according to the presence of neutralizing antibodies at baseline

### 8.2 Randomisation, blinding and allocation

#### 8.2.1 Regulatory documentation

Required regulatory and administrative documents must be provided to the sponsor before enrolment of the first patient. This will always include an ethics committee approval for the investigational site. Each investigational site will be notified when all requirements are met, and enrolment can start

#### 8.2.2 Registration and randomization

Eligible patients should be registered before start of treatment. Patients need to be registered in the electronic case record form. In principle the registration and randomization procedure can be modified in line with institutional specifics but should be similar to the following logistics:

All eligibility criteria will be checked with a checklist before the patient is included and randomized. Patients will be randomized with the use of an online randomization system (ALEA) using blocks of up to 12 in size {2 4 6 8 10 12}. Each patient will be given a unique patient study number (a sequence number by order of enrolment in the trial). Patient study number will be given immediately by the online registration database and confirmed by email. The result of the randomization will not be disclosed to the investigator but to a designated unblinded person from the transfusion lab in the hospital. The responsible physician will order the study plasma under the received study number.

#### 8.2.3 Unblinding procedures

While the safety of patients should always take priority, maintenance of blinding is crucial to the integrity of a double-blind trial. The blind for a specific patient should only be broken when information about the patient's protocol treatment is considered necessary to manage Serious Adverse Events (emergency unblinding). Unblinding procedures should preferably be initiated only after consultation of the (co) principal investigator or his/her representative. Emergency unblinding can be done by the site using the Emergency Unblinding form in the eCRF database. After completing and submitting the

eCRF form, ALEA will show the kits assigned to the patient along with the treatment the patient was assigned to. The HOVON Data Center will automatically receive an email that the patient has been unblinded. After unblinding, the patient goes off protocol treatment

## 8.3 Study procedures

### 8.3.1 Time of clinical evaluations

- ♦ Pre-screening
- ♦ Screening and baseline (day 1)
- ♦ Day 7, 14, 28 telephone contact for 5-point disease status (primary endpoint)
- ♦ Optional viro-immunological and clinical evaluations on 100 patients after additional consent as indicated in the tabel in 8.3.2

Viro-immunological evaluations will be performed in at least 100 and up to 400 patients (approximately half from both study arms) on the condition that the patient provides additional consent:

- ♦ Antibody titer measurement, one 6ml serumtube and fingerprick dried blot spot sample on day 1, 3, 7, 14, 28
- ♦ Nasopharyngeal swab for real-time quantitative PCR on day 1, 3, 7, 14, 28
- ♦ Immune response evaluation; four 9mL natrium heparine tubes, one 6mL EDTA tube and two 6mL serum tubes, on day 1, 14, 28 and on month 3, 6, 12

Additional clinical evaluations for patients who provided additional consent

- ♦ At least 100 and up to 400 patients aged 70 or older (approximately half from both study arms): Geriatric functional evaluation, by telephone contact, on day 1, 28, at month 3 and 6.
- ♦ At least 100 and up to 400 patients (approximately half from both study arms): Long-term lung damage with a lung function test at week 6, month 3 and, a low-dose CT on month 3 and a questionnaire on day 28, week 6, month 3. If the pulmonologist considers it necessary to continue follow up after month 3, a follow up CT-scan, lung function test and questionnaires will be done at these timepoints at 6 and 12 months (following routine care as determined by the NVALT)

### 8.3.2 Required investigations

Required investigations at entry, during treatment and during follow up:

| | **Prescreening with referring physician (*)** | **Screening Day 1** | **Baseline Day 1** (immediately following screening) | **D3** | **D7** | **D14** | **D28** | **M3** | **M6** | **M12** |
|---|---|---|---|---|---|---|---|---|---|---|
| Eligibility check (*) | x | x | | | | | | | | |
| Informed consent | | x | | | | | | | | |
| Lab (blood group, antibody) | | x | | | | | | | | |
| Plasma infusion | | | x | | | | | | | |
| Transcutaneous O2 saturation without administration of supplemental oxygen | | | x | | | | | | | |
| Registration of baseline characteristics and comorbidities defined in Appendix A | | x | | | | | | | | |
| COVID disease severity scale | | x | | | | | | | | |
| Telephone contact (1) | | | | | x | x | x | | | |

The items below are optional and only for patients and sites who choose to participate in certain sub-studies(x)

| | | | | | | | | | | |
|---|---|---|---|---|---|---|---|---|---|---|
| Antibody titer measurement (2) | | | x | x | x | x | x | | | |
| Nasopharyngeal swab (3) | | | x | x | x | x | x | | | |
| Immune response evaluation (4) (For cellular immunity sub-study sites) | | | x | | | x | x | x | x | x |
| Geriatric functional evaluation (5) | | | x | | | | x | x | x | |
| Pulmonology assessments (6) (For lung damage sub-study sites) | | | | | | | x(%) | x | x($) | x($) |
| CT lungs (6) (For lung damage sub-study sites) | | | | | | | | x | x($) | x($) |
| QoL questionnaires (6) (For lung damage sub-study sites) | | | | | | | x | x | x($) | x($) |

(x) = Optional: aim to include up to 400 patients to consent to (2), (3) and (4). Including all >70 years who consent to (5). Aim to include 50 patients to consent to (5) and (6).

(*) During a telephone call with the referring physician, the in -and exclusion criteria are checked.

(1) Disease status is checked by designated person from the project group and registered as

1= Recovered with no symptoms on date xx/xx/xxxx) OR

2= Continued symptoms attributable to COVID-19 OR

3= Admitted to hospital but no invasive ventilation needed OR

4= Admitted and invasive ventilation needed OR

5= Death

If admitted, also additional anti-SARSCOV2 treatment is registered.

In grey are the optional investigations for selected patients and selected study sites;

(2) Antibody titer measurement: 1 serum tube of 6mL is collected and stored at minus 70 on site

(3) The SARS-COV-2 PCR is done in the virology lab at the study site. Therefore, this can only be done if the local virology lab is able to report on the RT- PCR Cycle Treshold values of the PCR.

(4) Immune response evaluation: 4 natrium heparine tubes of 9mL each, 1 EDTA tube of 6mL, 2 serum tubes of 6mL, on day 1, 14, 28 and **optional** on month 3, 6, 12

These samples have to arrive at Erasmus MC in Rotterdam before 13h00. Samplings is therefore only possible between 8 and 11h00 and the study team should be contacted 48hrs in advance to plan a pickup

(5) Geriatric functional evaluation will be done by the LUMC study team via a telephone contact with the patient and include KATZ-ADL, LAWTON-IADL, living situation and EQ5D.

(6) Lung function is done at d28 and month 3, questionnaire is done at day 28, month 3, a low-dose CT is done 3 months after inclusion in the study. If it is abnormal, the investigator and patient will decide if further follow-up is planned according to NVALT guideline at month 6 and month 12 which includes a questionnaire and a CT/lung function outside the context of the study. The daily functioning questionnaires SGRQ and KBUILD, and the QoL EQ5D questionnaire will be evaluated in consenting individuals.

Notes on the pulmonary assessments:

(%) Can only be done if the patient no longer needs to be seen in contact isolation

($) Will only be done if the investigator decides that further follow-up after the 3-month visit is needed as part of routine care

### 8.3.3 Specification of required investigations

**Inclusion**

**Medical history**

- Sex and age
- Etnicity
- Socio-economic status
- BMI at inclusion into the study
- Date of first day of symptoms of SARS-CoV-2 infection
- Underlying medical illnesses at the time of first day of SARS-CoV-2 RT-PCR positive
- Charlson Comorbidity Index
- Clinical Frailty Scale for patients aged 70 or older
- If available from previous testing in the preceding 72 hours; CRP
- If available from previous testing in the preceding 7 days; SARS-CoV-2 RT-PCR Ct value

**Physical examination at baseline**

- Transcutaneous O2 saturation without administration of supplemental oxygen
- Respiratory rate, blood pressure, pulse, temperature

**Lab**

- Bloodgroup
- Antibody testing at t=0: Presence of anti-SARS-CoV-2 RBD-protein and anti-SARS-CoV-2 nucleocapsid protein antibodies will be tested in a highly sensitive briding assay (Wantei test or equivalent test). In the positive samples the antibodies will be specified and quantified in IgG, IgA and IgM ELISA's against RBD and Nucleocapsid protein
- Nasopharynx swab for SARS-CoV-2 RT-PCR testing
- Is the patient is able to produce sputum: Sputum sample will be collected and stored for future research

**Follow-up**

**Clinical**

- 5-point disease severity status (see primary endpoint) by telephone on day 7, 14 and 28
- Any other anti-SARS-CoV-2 treatments that were given

**Laboratory and pulmonary (after additional consent by the patient)**

- Antibody testing using finger prick blood (sampled in outpatient setting or at home): Antibodies against RBD and nucleocapsid (IgG, IgM and IgA)

- SARS-CoV-2 RT-PCR Ct values
- Viral culture
  - Cellular and humoral anti-SARS-CoV-2 immunity (Nucleocapsid and Spike protein specific CD4+ cells using previously selected peptide pools,[23] CD8+ cells using classI tetramaeric complexes as presently being developed at Sanquin,[24] antigen specific B cells by FCM using tetrameric spike and nucleocapsid complexes[25] geriatric evaluation with questionnaires
- CT-lungs
- Spirometry
- Lung evaluation with questionnaires

## 8.4 Withdrawal of individual patients from protocol treatment

Patients should be withdrawn from protocol treatment if any of the following criteria for withdrawal are met:

♦ Potentially life-threatening transfusion reaction during plasma infusion.

Patients can leave the study at any time for any reason if they wish to do so without any consequences. The investigator can also decide to withdraw a patient from protocol treatment for urgent medical reasons. Patients who are withdrawn from protocol treatment will receive medical care according to local practice. Urgent medical reasons can force unblinding the treatment by the investigators (e.g. when a high clinical suspicion of unwanted plasma contamination by pathogens is suspected). Patients will remain in the analysis then.

## 8.5 Replacemnts of individual subjects after withdrawal

## 8.6 Follow up of patients withdrawn from protocol treatment

Patients who are withdrawn from treatment for other reasons than death will be followed for disease status as described in 8.3.2 if they consent. SAE information will be collected as described in 9.2.

## 8.7 Premature termination of the study

The sponsor may decide to terminate the study prematurely based on the following criteria:

♦ There is evidence of unacceptable risks for study patients (i.e. safety issue);

♦ There is ample evidence of efficacy

♦ There is reason to conclude that continuation of the study cannot serve a scientific purpose

The sponsor will promptly notify all concerned investigators, the DSMB, the ethics committee(s) and the regulatory authorities of the decision to terminate the study. The sponsor will provide information regarding the timelines of study termination and instructions regarding treatment and data collection of enrolled patients.

## 8.8 Data collection

Data will be collected on electronic case report forms (CRF, ALEA) to document eligibility, safety and efficacy parameters, compliance to treatment schedules and parameters necessary to evaluate the study endpoints. Data collected on the CRF are derived from the protocol and will include at least:

- Inclusion and exclusion criteria;
- Baseline status of patient including medical history and stage of disease;
- Timing and dosage of protocol treatment;
- Any other parameters necessary to evaluate the study endpoints;
- Survival status of patient;

Each CRF page will be identified by a trial number, and a combination of patient study number (assigned at registration) and hospital name.
The e-CRF will be completed on site by the investigator or sub-investigator or an authorized staff member. All CRF entries must be based on source documents.

Data collected on the CRF will be verified for accuracy. If necessary, queries will be sent to the investigational site to clarify the data on the CRF. The investigator should answer data queries within the specified timeline.

### 8.8.1 Rapid reporting

To enable continuous monitoring as described in chapter 9.5, all sites shall submit the results of the ordinal outcome (primary endpoint) in the eCRF within 7 days after the patient has passed the 28 days follow up time point. This will enable the described continuous monitoring and with it the analysis of possible superiority or inferiority of one of the treatment arms.

# 9 Safety

## 9.1 Temporary halt for reasons of subject safety

In accordance to section 10, subsection 4, of the WMO, the sponsor will suspend the study if there is sufficient ground that continuation of the study will jeopardise subject health or safety. The sponsor will notify the accredited METC without undue delay of a temporary halt including the reason for such an action. The study will be suspended pending a further positive decision by the accredited METC. The investigator will take care that all subjects are kept informed.

## 9.2 AEs and SAEs

### 9.2.1 Adverse events (AEs)

Adverse events are defined as any undesirable experience occurring to a subject during the study, whether or not considered related to the trial procedure/ the experimental intervention.

However, only AE at least possibly related to the plasma transfusion will be registered. AE grade 1 will not be reported.

AE from plasma infusion until 30 days following plasma will be reported and are defined in detail in appendix F. They will only be reported if considered at least possibly related to the plasma infusion. Adverse events have to be reported on the Adverse Events CRF. Adverse events will be scored according to the National Cancer Institute (NCI) Common Terminology Criteria for Adverse Events (CTCAE), version 5.0 (see appendix D).

### 9.2.2 Serious adverse events (SAEs)

A serious adverse event is any untoward medical occurrence or effect that

- results in death;
- is life threatening (at the time of the event);
- requires hospitalisation or prolongation of existing inpatients' hospitalisation;
- results in persistent or significant disability or incapacity;
- any other important medical event that did not result in any of the outcomes listed above due to medical or surgical intervention but could have been based upon appropriate judgement by the investigator.

An elective hospital admission will not be considered as a serious adverse event.

The registration of SAE will be limited to the following:

- ♦ Death
- ♦ Life threatening transfusion reactions
- ♦ Hospitalisation or prolongation of existing inpatients' hospitalisation;

However, the following events do not require to be reported as a serious adverse event:

- ♦ Hospitalization for protocol therapy administration. Hospitalization or prolonged hospitalization for a complication of therapy administration will be reported as an SAE.
- ♦ Hospitalization for diagnostic investigations (e.g., scans, endoscopy, sampling for laboratory tests, bone marrow sampling) that are not related to an adverse event. Hospitalization or prolonged hospitalization for a complication of such procedures remains a reportable serious adverse event.
- ♦ Prolonged hospitalization for technical, practical, or social reasons, in absence of an adverse event.
- ♦ Hospitalization for a procedure that was planned prior to study participation (i.e. prior to registration or randomization). This should be recorded in the source documents. Prolonged hospitalization for a complication of such procedures remains a reportable SAE.

Serious Adverse Events (SAEs) will be reported from the moment of plasma infusion according to protocol until 60 days following infusion.
SAEs (including death) occurring after 60 days and/or after infusion should also be reported if considered at least possibly related to the plasma infusion.
The investigator will report the SAEs to the HOVON Safety Desk **within 24 hours** after obtaining knowledge of the events using the SAE report form provided.

HOVON Safety Desk will report the SAEs through the web portal ToetsingOnline to the accredited METC that approved the protocol, within 7 days of first knowledge for SAEs that result in death or are life threatening followed by a period of maximum of 8 days to complete the initial preliminary report. All other SAEs will be reported within a period of maximum 15 days after the sponsor has first knowledge of the serious adverse events.

#### 9.2.3 Suspected unexpected serious adverse reactions (SUSARs)

NOT APPLICABLE

### 9.3 Annual safety report

NOT APPLICABLE

## 9.4 Follow-up of adverse events

All AEs and SAEs will be followed until they have abated, or until a stable situation has been reached. Depending on the event, follow up may require additional tests or medical procedures as indicated, and/or referral to the general physician or a medical specialist.
SAEs need to be reported till end of study within the Netherlands, as defined in the protocol

## 9.5 Data Safety Monitoring Board (DSMB)

A data safety monitoring board will be implemented to review the available study data regarding SAEs observed during the trial, review the safety of the participants on a regular basis and recommend the study team regarding the further conduct of the study. The Bayesian design allows for multiple evaluations of the data without the need to adjust the global alpha level for multiplicity. Therefore, the posterior distribution of the primary efficacy parameter (common log odds ratio) will be monitored in an unblinded fashion by one of the trial statisticians and reported to the DSMB after every 50 patients or 3 months (whatever comes first). If the posterior probability of a positive effect (OR lower than 1) reaches 97.5% or the posterior probability of a negative effect (OR greater than one) reaches 80%, the DSMB will be informed and advise the study team about the continuation of the study.

**Importantly**, to make this possible, all sites shall submit the results of the ordinal outcome (primary endpoint) in the eCRF within 7 days after the patient has passed the 28 days follow up time point. This will enable the described continuous monitoring and with it the analysis of possible superiority or inferiority of one of the treatment arms.

The DSMB will receive the first review of SAEs after the outcomes of 20 patients are available. Then this will be continued, after the outcomes e.g. of every 50 patients at the discretion of the DSMB’s wishes. The DSMB will consist of a biostatistician (prof. Bossuyt), an infectious diseases specialist (dr. JL Nouwen), a medical ethicist (prof MC de Vries)

**Missing Data**

Although the covariates to be used for the analysis of the primary endpoint are unlikely to be missing, this is always a possibility. At the time of planned analyses, we will assess the data for missingness. If necessary multiple imputation will be used to impute missing covariate data. Both the results of the imputed and non-imputed data will be presented.

# 10 Statistical considerations

## 10.1 Patient numbers and power considerations

The study intervention is anticipated to improve the score on the 5-point ordinal scale (i.e. result in a shift from the higher towards the lower categories). In particular, the intervention is expected to lower the risk of a hospital admission, need for mechanical ventilation and death by 33%, translating in an odds ratio of 0.63 which we also assume for the shift in the other categories.

With the inclusion criteria mentioned above, we are targeting a patient population in whom the risk of hospital admission or death is estimated to be at least 20% in the 28 days following transfusion. The primary endpoint analysis will be based on an adjusted version of the WHO 10-point COVID-19 disease severity score ordinal scale specifically developed for this study as the full WHO 10-point ordinal scale is not suited to study outpatients. Furthermore, we are using an endpoint that allows for a large pragmatic trial design by which the data can be collected by a telephone interview on day 7, 14 and 28 if the category 3 to 5 is not observed in the patient.

1 = Recovered (no symptoms) within 7 days after transfusion
2 = Continued symptoms attributable to COVID-19 on day 8 after transfusion
3 = Admitted to the hospital but no invasive ventilation needed
4 = Admitted to the hospital and invasive ventilation needed
5 = Death

The event rates that we estimate for each of the groups are based on RIVM data (table 2), on a Chinese study in which the COVID-19 clinical evolution in a large group of patients are described (also in the subgroup of 50-59 in the supplemental data of that paper) and on data from the ConCOVID study.[15,26]

Assuming a common odds ratio of 0.63, using a two-sided test in a proportional odds model, assuming 15% attrition (loss to follow up and admission events occurring before inclusion) a sample size of 690 is needed for a study with a power of 80% (923 for 90% power).

| | Recovered within 7 days | Continued symptoms beyond 7 days | Hospital admission, not invasive ventilation | Hospital admission with invasive ventilation | Death |
|---|---|---|---|---|---|
| Control (FFP) | 22% | 63% | 3% | 3% | 9% |
| Treatment (ConvP) | 31% | 59% | 2.1% | 2.1% | 5.9% |

## 10.2 Primary endpoint analysis

The highest disease status on the 5-point ordinal scale in the 28 days following transfusion will be analyzed using the intention-to-treat principle. A Bayesian proportional odds model will be used for the analysis of the highest disease status on the 5-point ordinal scale in the 28 days following transfusion. Apart from treatment, the following factors will be included in the model: age, sex, BMI, number of comorbidities, o2 saturation at inclusion, and duration of symptoms at inclusion. The specification of the prior distributions for the parameters in the model is as follows:

- Efficacy will be assessed using a skeptical prior. More specifically for the common log odds ratio of treatment we will use a normal distribution with zero-mean and a standard deviation of 0.42. This ensures that the prior probability of the OR to be greater than 2 is 0.05 and that the prior probability of the OR to be less than ½ is 0.05, in order to quantify our prior belief that a very large effect might be unlikely.

- A Dirichlet (1, 1, 1, 1, 1) non-informative prior will be used for the 5 categories of the ordinal outcome in the control arm.

- Vague priors will be used for the log odds ratios of the covariates included in the model: age, sex, BMI, o2 saturation at inclusion, number of comorbidities and duration of symptoms at inclusion. More specifically, normal distributions with mean zero and standard deviation of 0.5.

## 10.3 Secondary endpoint analysis

1. The adjusted hazard ratio of death over the follow-up period of 28 days between convP and FFP will be estimated using a multivariable proportional hazards model.
2. The adjusted cause-specific hazard ratio of hospital admission over the follow-up period of 28 days between convP and FFP will be estimated using a multivariable proportional hazards model, censoring at death. To describe the absolute risk of hospital admission, cumulative incidence curves for hospital admission in both convP and FFP groups will be presented.
3. The adjusted cause-specific hazard ratio of ICU admission over the follow-up period of 28 days between convP and FFP will be estimated using a multivariable proportional hazards model, censoring at death. To describe the absolute risk of ICU admission, cumulative incidence curves for hospital admission in both convP and FFP groups will be presented.
4. The difference in median duration of symptoms over the follow-up period of 28 days between convP and FFP will be estimated using quantile regression on the subset of alive patients.

5. Differences in the effect of treatment (quantified by the adjusted OR for the primary endpoint), will be investigated by age and clinical frailty score between convP and FFP. For this, the proportional odds model from the primary endpoint will be extended with an interaction term between treatment and age (as a continuous variable) and an interaction term between treatment and clinical frailty score to capture differences in the effect of treatment per unit change in age and clinical frailty score.

In all above analyses (1-5) we will adjust for the following covariates, if the outcome frequency allows: age, sex, BMI, o2 saturation at inclusion, number of comorbidities and duration of symptoms at inclusion. In case of a low observed event per variable (EPV) rate (e.g. EPV < 10) a subset of these factors will be included in the model.

Evaluated in subgroups of patients:

1. The impact of 300ml convP on functional decline (frailty, cognitive functioning) in patients aged 70 or older. Functional decline will be measured with the use of the following questionnaires: Katz-ADL, Lawton-IADL, living situation and EQ5D at d1, 28, m3, m6. Data from each questionnaire will be analyzed with appropriate regression models according to outcome distribution and its features.
2. FVC (% predicted of normal for age) and lung diffusion (DLCOc, % predicted of normal for age) are continuous quantitative measure of lung function and structural lung damage is assessed by a continuous quantative measure using a validated automated functional respiratory imaging (FRI) to evaluate the patient’s airway and lung geometry (Fluidda®, ref https://www.fluidda.com/publications/). Mixed-effect models will be constructed to evaluate changes in FVC, DLCOc, FRI over time and compare these changes between the treatment groups (interaction between time and treatment group). We will adjust for age, sex, BMI, o2 saturation at inclusion, number of comorbidities and duration of symptoms at inclusion.
3. The probability of SARS CoV 2 genome detected by RT PCR over time (measurements at day 3, 7 and 14 and 28) will be compared between convP and FFP. For this analysis a Generalized Estimating Equations (GEE) model will be used with an interaction term between time and treatment and an interaction term between treatment and presence of neutralizing antibodies at inclusion. The latter interaction terms will allow to assess differences in the OR between people with and without neutralizing antibodies at inclusion (exploratory analysis).
4. Evolution of anti-SARS-CoV-2 memory humoral and cellular immunological memory measured as anti-SARS-COV2 specific B-cell and CD8Tcell responses. A mixed effects model will be used for the analysis of serial measurements adjusted for age and sex, covid disease severity (outpatient or hospital admission or ICU admission <28 days), use of immunosuppressive medication, and having an immune disorder.

5. The mean incremental cost effectiveness ratio will be calculated by the costs of the invervention divided by the costs saved based on prevented care by convP compared to FFP. The following parameters will be taken into account in this analysis; QOL, mortality, hospital days on ward and on ICU and for patients with a payed job at the time of COVID disease onset also the loss-of-income data up to 6 months after inclusion.

## 10.4 Exploratory endpoints:

Differences in the effect of treatment (quantified by the adjusted OR for the primary endpoint), will be investigated by the presence of neutralizing antibodies at baseline and by the duration of symptoms at baseline. For this, the proportional odds model from the primary endpoint will be extended with an interaction term between treatment and presence of neutralizing antibodies and an interaction term between treatment and duration of symptoms (as a continuous variable) to capture differences in the effect of treatment with these two factors.

## 10.5 Interim efficacy and safety analysis

See chapter 9.5

## 10.6 Stopping rules

See DSMB chapter 9.5 above. In addition, circumstances that may cause an DSMB's advise to terminate the study include, but are not limited to:

- newly emerging effective treatment for Covid-19 which would substantially change the risk- benefit assessment of the investigational approach in this trial compared to alternative options.
- Determination of unexpected, significant, or unacceptable risk to patients
- Failure to recruit patients at an acceptable rate
- Insufficient adherence to protocol requirements
- Insufficient complete and/or evaluable data
- Serious non-compliance to ICH-GCP standards
- Plans to modify, suspend or discontinue the development of the study drug
- Unexpected, significant, or unacceptable risk to patients

Written notification documenting the reason(s) for study termination advice will be provided to the investigator by the relevant party (DSMB, interim analysis statistician, monitoring agencies).

a. Institutional investigators may terminate their centre's participation to the study. If this occurs, they should provide a written statement of the reasons for terminating participation but remain obligated to manage all included patients according to the study protocol and the signed PIFs and should provide all relevant data to the Sponsor (i.e. the by the Sponsor designated person).

b. The sponsor may also decide to terminate participation of an investigator or study centre for the following reasons:

- Breach of agreement
- Insufficient patient recruitment

The sponsor will promptly notify all concerned investigators, the ethics committee(s) and the regulatory authorities of the decision to terminate the study. The sponsor will provide information regarding the timelines of study termination and instructions regarding treatment and data collection of enrolled patients.

# 11 Ethical considerations

## 11.1 Regulations statement

The study will be conducted in accordance with the ethical principles of the Declaration of Helsinki (2013), the ICH-GCP Guidelines, the EU Clinical Trial Directive (2001/20/EG), and applicable regulatory requirements. The site investigator is responsible for the proper conduct of the study at the study site.

## 11.2 Recruitment and consent

In general, patients presenting at the GGD or GP with the diagnosis under study and possibly qualifying for participation will be informed about the trial by the GP and asked if they are interested to participate. If the patient is interested the GP will inform the site investigator of the nearby participating hospital.

Specific to this study protocol, patients diagnosed with COVID-19 can be informed about the possibility to be referred to a study site by anyone. If the patient agrees, his/her treating physician (typically his GP) will contact the study team and if the study in -and exclusion criteria seem to be fulfilled, a member of the study team will call the patient to inform him/her about the study.

Written informed consent of patients is required before enrolment in the trial and before any study related procedure takes place. ICH-GCP and other applicable regulations must be followed in informing the patient and obtaining consent. It should be taken into consideration if the patient is capable of giving informed consent. If not, a legal representative may provide informed consent. Before informed consent may be obtained, the patient (and if relevant his/her legal representative) should be given ample time and opportunity to inquire about details of the trial and to decide whether or not to participate in the trial. All questions about the trial should be answered to the satisfaction of the patient. There is no set time limit for the patient to decide. The investigator should inform each patient if there is a specific reason why he/she must decide within a limited time frame, for example if the patient is close to the <8 days since symptom onset period or if the trial is scheduled to close for enrolment.

The content of the patient information letter, informed consent form and any other written information to be provided to patients will be in compliance with ICH-GCP, GDPR and other applicable regulations and should be approved by the ethics committee in advance of use.
The patient information letter, informed consent form and any other written information to be provided to patients will be revised whenever important new information becomes available that may be relevant to the patient’s consent. Any substantially revised informed consent form and written information should be approved by the ethics committee in advance of use. The patient should be informed in a timely manner if new information becomes available that might be relevant to the patient’s willingness to continue participation in the trial. The communication of this information should be documented.

## 11.3 Objection by minors or incapacitated subjects

If a study person for whom a legal representative has given informed consent at any time objects to a study procedure (e.g. blood sampling), the procedure will be stopped. If this makes further study participation impossible, the patient will be withdrawn from the study.

## 11.4 Benefits and risks assessment.

A potential benefit may be a lower risk for a bad outcome (hospital admission, death) and a shorter disease course. This however, remains to be proven. No additional blood samples will be taken (apart from a small proportion of patients who provide separate consent to inclusion for immunological follow up). No additional visits are required. The risk of plasma infusion is comparable to the risk associated with blood transfusions. These include transfusion reactions, transfusion related acute lung injury (TRALI) and the transmission of as yet unknown infectious or other transmittable diseases. The precautions as taken by the Sanquin Blood Supply regarding the prevention of infectious and non-

infectious complications of blood product transfusion are taken in this study. These include, matching the donor and recipient for blood group, testing for infectious agents as well as testing for irregular antibodies and when indicated HLA- and HNA-antibody testing in the donor.

### 11.5 Compensation for injury

The sponsor/investigator has a liability insurance which is in accordance with article 7 of the WMO.

The sponsor (also) has an insurance which is in accordance with the legal requirements in the Netherlands (Article 7 WMO). This insurance provides cover for damage to research subjects through injury or death caused by the study.
The insurance applies to the damage that becomes apparent during the study or within 4 years after the end of the study.

### 11.6 Incentives

Patient will not be compensated for participation in the trial.
However, patients participating in the sub-studies will receive compensation for travel expenses.

## 12 Administrative aspects and publication

### 12.1 Handling and storage of data and documents

Data and documents will be controlled and processed conform the EU General Data Protection Regulation (GDPR) and the Dutch Act on Implementation of the General Data Protection Regulation. (in Dutch: Uitvoeringswet AVG, UAVG).

#### 12.1.1 Patient confidentiality

Each patient is assigned a unique patient study number at enrolment. In trial documents the patient's identity is coded by patient study number as assigned at enrolment. The site investigator will keep a subject enrolment and identification log that contains the key to the code, i.e. a record of the personal identification data linked to each patient study number. This record is filed at the investigational site and should only be accessed by the investigator and the supporting hospital staff, and by representatives of the sponsor or a regulatory agency for the purpose of monitoring visits or audits and inspections. Patients confidentiality will be ensured in compliance with EU regulation and the Dutch Act on Implementation of the General Data Protection Regulation.

#### 12.1.2 Filing of essential documents

Essential documents are those documents that permit evaluation of the conduct of a trial and the quality of the data produced. The essential documents may be subject to, and should be available for, audit by the sponsor's auditor and inspection by the regulatory authority(ies)
The investigator should file all essential documents relevant to the conduct of the trial on site. The sponsor will file all essential documents relevant to the overall conduct of the trial. Essential documents should be filed in such a manner that they are protected from accidental loss and can be easily retrieved for review.

#### 12.1.3 Record retention

Essential documents should be retained for 25 years after the end of the trial. They should be destroyed after this time, unless a longer record retention period is required by site specific regulations.
Source documents (i.e. medical records) of patients should be retained for at least 15 years after the end of the trial described in section 12.5. Record retention and destruction after this time is subject to the site's guidelines regarding medical records.

#### 12.1.4 Storage and sharing of data

Electronic patient data collected in the e-CRF will be stored at the sponsor for 25 years.
The data collected by questionnaires filled in by the consenting donors will be stored for 25 years. Encoded data may be shared with other study groups for research purposes, for example with the EU COVID initiative (https://joinup.ec.europa.eu/collection/digital-response-covid-19/news/european-covid-19-data-platform). If data are sent to countries outside de EU, patients confidentiality will be ensured at an equal level of EU regulation and the Dutch Act on Implementation of the General Data Protection Regulation.

#### 12.1.5 Storage of samples

Biological samples should only be stored for the purpose of additional research if the patient has given consent. If no informed consent was obtained, samples should be destroyed after the patient has completed all protocol treatment and procedures. Sampling in participating sites is possible but only if done according to the CoV-Early standard operating procedure for this. After sampling, the samples from participating non-academic sites should be shipped and stored to a designated storage facility for biological samples from one of the academic partners within the CoV-Early group. Sample processing is done according to uniform standard operating procedures. Samples that are shipped to another facility (e.g. a central laboratory) for a purpose as described in this protocol or for additional scientific

research, should be stripped from any identifying information and labeled with a code (trial name or number and patient study number as assigned at enrolment).

## 12.2 Monitoring and quality insurance

The sponsor will perform on-site monitoring visits to verify that the rights and well-being of patients are protected, the reported trial data are accurate, complete, and verifiable from source documents and the conduct of the trial is in compliance with the currently approved protocol/amendment(s), with GCP, and with the applicable regulatory requirement(s). Monitoring visits will take place according to the study specific monitoring plan.

Direct access to source documentation (medical records) must be allowed for the purpose of verifying that the data recorded in the CRF are consistent with the original source data. The sponsor expects that during monitoring visits the relevant investigational staff will be available, the source documentation will be available, and a suitable environment will be provided for review of study-related documents.

In accordance with regulatory guidelines, audits may be carried out for this study. The investigator is required to facilitate an audit by means of a site visit.
These audits will require access to all study records, including source documents, for inspection and comparison with the CRFs. Patient privacy must, however, be respected.
Similar auditing procedures may also be conducted by agents of any regulatory body reviewing the results of this study. The investigator should immediately notify the sponsor if they have been contacted by a regulatory agency concerning an upcoming inspection.

## 12.3 Amendments

A ‘substantial amendment’ is defined as an amendment to the terms of the ethics committee application, or to the protocol or any other supporting documentation, that is likely to affect to a significant degree:

- the safety or physical or mental integrity of the patients of the trial;
- the scientific value of the trial;
- the conduct or management of the trial; or
- the quality or safety of any intervention used in the trial.

All substantial amendments will be submitted to the ethics committee.

Non-substantial amendments will not be submitted but will be recorded and filed by the sponsor.

### 12.4 Annual progress report

The sponsor will submit a summary of the progress of the trial to the accredited ethics committee once a year. Interim reports will be generated more frequently on the inclusion rate (weekly) and main outcomes/complications (quarterly) for evaluations by the project group and DSMB. The first report for the METC is sent one year after the first approval date of the trial. Subsequent reports are sent annually until end of trial. Information will be provided on the date of inclusion of the first patient, numbers of patients included and numbers of patients that have completed the trial, serious adverse events/ serious adverse reactions, other problems, and amendments.

### 12.5 Temporary halt and (prematurely) end of trial report

The sponsor will notify the accredited ethics committee and the competent authority of the end of the trial within a period of 8 weeks. The end of the trial is defined as the last patient's last visit.

The sponsor will notify the METC immediately of a temporary halt of the study, including the reason of such an action.

In case the study is ended prematurely, the sponsor will notify the accredited ethics within 15 days, including the reasons for the premature termination.

Within one year after the end of the trial, the sponsor will submit an end of trial report with the results of the study, including any publications/abstracts of the study, to the accredited ethics committee.

### 12.6 Public disclosure and publication policy

Trial results will always be submitted for publication in a peer reviewed scientific journal regardless of the outcome of the trial – unless the trial was terminated prematurely and did not yield sufficient data for a publication. Reports will also be shared through preprint servers as soon as possible.

## 13 Structured risk analysis

### 13.1 Potential issues of concern

The only concern is the concern that exists for the use of any human blood products.

No other concerns are in place. No SAE or AE occurred with the use of convP for COVID-19 in the previous Dutch ConCOVID RCT on convP.

a. Level of knowledge about mechanism of action
See introduction paragraph of the protocol

b. Previous exposure of human beings with the test product(s) and/or products with a similar biological mechanism.
See introduction paragraph of the protocol

c. Can the primary or secondary mechanism be induced in animals and/or in ex-vivo human cell material?
Yes, animal model in hamsters

d. Selectivity of the mechanism to target tissue in animals and/or human beings
Unknown

e. Analysis of potential effect
See introduction paragraph of the protocol

f. Pharmacokinetic considerations
Unknown for SARS-Cov-2 antibodies at this time

g. Study population
SARS-CoV-2 infected patients not admitted to the hospital but at risk for disease progression

h. Interaction with other products
None

i. Predictability of effect
Not predictable at this time

j. Can effects be managed?
Transfusion reactions are managed with standard transfusion reaction management protocols

## 13.2 Synthesis

All standard blood product safety measures are in place except for the 4-month quarantine period in ConvP that is normally adhered to. The overall risk of a single allogeneic plasma transfusion is low. To reduce this risk further, we will only use plasma from male donors who have no history of blood transfusion, or female donors / donors with a history of blood transfusion before 01-01-1980 when tested negative for HLA- and HNA-antibodies. Given the estimated 20% risk of hospital admission or dead of the disease in the population under study the risk is considered acceptable.

# 14 References


1. Dexamethasone in Hospitalized Patients with Covid-19 — Preliminary Report. *New England Journal of Medicine.* 2020.
2. Hung IF-N, Lung K-C, Tso EY-K, et al. Triple combination of interferon beta-1b, lopinavir–ritonavir, and ribavirin in the treatment of patients admitted to hospital with COVID-19: an open-label, randomised, phase 2 trial. *The Lancet.* 2020;395(10238):1695-1704.
3. Beigel JH, Tomashek KM, Dodd LE, et al. Remdesivir for the Treatment of Covid-19 - Preliminary Report. *N Engl J Med.* 2020.
4. Hung IF-N, Lung K-C, Tso EY-K, et al. Triple combination of interferon beta-1b, lopinavir–ritonavir, and ribavirin in the treatment of patients admitted to hospital with COVID-19: an open-label, randomised, phase 2 trial. *The Lancet.* 2020;395(10238):1695-1704.
5. Horby P, Lim WS, Emberson J, et al. Effect of Dexamethasone in Hospitalized Patients with COVID-19: Preliminary Report. *medRxiv.* 2020:2020.2006.2022.20137273.
6. Mair-Jenkins J, Saavedra-Campos M, Baillie JK, et al. The effectiveness of convalescent plasma and hyperimmune immunoglobulin for the treatment of severe acute respiratory infections of viral etiology: a systematic review and exploratory meta-analysis. *J Infect Dis.* 2015;211(1):80-90.
7. Imai M, Iwatsuki-Horimoto K, Hatta M, et al. Syrian hamsters as a small animal model for SARS-CoV-2 infection and countermeasure development. *Proc Natl Acad Sci U S A.* 2020.
8. Shen C, Wang Z, Zhao F, et al. Treatment of 5 Critically Ill Patients With COVID-19 With Convalescent Plasma. *Jama.* 2020;323(16):1582-1589.
9. Joyner MJ, Wright RS, Fairweather D, et al. Early safety indicators of COVID-19 convalescent plasma in 5,000 patients. *J Clin Invest.* 2020.
10. Okba NMA, Müller MA, Li W, et al. Severe Acute Respiratory Syndrome Coronavirus 2-Specific Antibody Responses in Coronavirus Disease 2019 Patients. *Emerg Infect Dis.* 2020;26(7).
11. Wang X, Guo X, Xin Q, et al. Neutralizing Antibodies Responses to SARS-CoV-2 in COVID-19 Inpatients and Convalescent Patients. *Clin Infect Dis.* 2020.
12. Liu STH, Lin H-M, Baine I, et al. Convalescent plasma treatment of severe COVID-19: A matched control study. *medRxiv.* 2020:2020.2005.2020.20102236.
13. Perotti C, Baldanti F, Bruno R, et al. Mortality reduction in 46 severe Covid-19 patients treated with hyperimmune plasma. A proof of concept single arm multicenter interventional trial. *medRxiv.* 2020:2020.2005.2026.20113373.
14. Duan K, Liu B, Li C, et al. Effectiveness of convalescent plasma therapy in severe COVID-19 patients. *Proceedings of the National Academy of Sciences.* 2020;117(17):9490-9496.
15. gharbharan Aea. Convalescent Plasma for COVID-19. A randomized clinical trial. *Medxriv.* 2020.
16. Larsen MD, de Graaf EL, Sonneveld ME, et al. Afucosylated immunoglobulin G responses are a hallmark of enveloped virus infections and show an exacerbated phenotype in COVID-19. *bioRxiv.* 2020:2020.2005.2018.099507.
17. Petkova E, Antman EM, Troxel AB. Pooling Data From Individual Clinical Trials in the COVID-19 Era. *Jama.* 2020;324(6):543-545.
18. Magleby R, Westblade LF, Trzebucki A, et al. Impact of SARS-CoV-2 Viral Load on Risk of Intubation and Mortality Among Hospitalized Patients with Coronavirus Disease 2019. *Clin Infect Dis.* 2020.
19. Petrilli CM, Jones SA, Yang J, et al. Factors associated with hospital admission and critical illness among 5279 people with coronavirus disease 2019 in New York City: prospective cohort study. *BMJ.* 2020;369:m1966.
20. Wang G, Wu C, Zhang Q, et al. C-Reactive Protein Level May Predict the Risk of COVID-19 Aggravation. *Open Forum Infect Dis.* 2020;7(5):ofaa153.

21. Williamson EJ, Walker AJ, Bhaskaran K, et al. OpenSAFELY: factors associated with COVID-19 death in 17 million patients. *Nature.* 2020.
22. Vogelzang EH, Loeff FC, Derksen NIL, et al. Development of a SARS-CoV-2 total antibody assay and the dynamics of antibody response over time in hospitalized and non-hospitalized patients with COVID-19. *medRxiv.* 2020:2020.2006.2017.20133793.
23. Oja AE, Saris A, Ghandour CA, et al. Divergent SARS-CoV-2-specific T and B cell responses in severe but not mild COVID-19. *bioRxiv.* 2020:2020.2006.2018.159202.
24. Ferretti AP, Kula T, Wang Y, et al. COVID-19 Patients Form Memory CD8+ T Cells that Recognize a Small Set of Shared Immunodominant Epitopes in SARS-CoV-2. *medRxiv.* 2020:2020.2007.2024.20161653.
25. Brouwer PJM, Caniels TG, van der Straten K, et al. Potent neutralizing antibodies from COVID-19 patients define multiple targets of vulnerability. *Science.* 2020;369(6504):643.
26. Bi Q, Wu Y, Mei S, et al. Epidemiology and transmission of COVID-19 in 391 cases and 1286 of their close contacts in Shenzhen, China: a retrospective cohort study. *Lancet Infect Dis.* 2020.

## A. Risk factors for severe COVID-19

**Age 70 or older:**

Patients aged 70 or older are at sufficient risk and can be included regardless of medical history or lab results as long as they fulfill all other inclusion criteria.

**Age 50-69:**

For patients age 50-69, one of the following risk factors has to be present. This can be according to the medical history of the patient or a lab-based risk factor (if available)

**1. A/ Medical history**

- Obesity with BMI 35 or higher
- Born as a male person
- History of cardiac or pulmonary disease (e.g. but not limited to atrial fibrillation, coronary artery disease, heart failure, COPD, asthma)
- History of neurological disease (e.g. a history of stroke or any other chronic debilitating neurological disease)
- Diabetes for which medical therapy is needed
- Chronic kidney disease with GFR <60 ml/min
- Reumatic disease (e.g. reumatoid arthritis, Systemic lupus erythematosus, psoriatric artritis)
- Immunodeficiency (e.g. organ or allogeneic transplantation, systemic immunosuppressive drugs)
- Cancer not in complete remission for >1 year (excluding baso -or spinocellular skin cancers)
- Untreated HIV and CD4 T-cells <200/microliter
- Chronic liver disease being liver cirrhosis child pugh A/B/C or other disease leading to liver dysfunction

**1. B/ Lab results (if available)**

- CRP > 30
- SARS-CoV-2 RT-PCR Ct value <25

## B. RIVM data

An example of data from the early epidemic in the Netherlands. We also used more recent data that RIVM provided to base our power and sample size calculations on.

### 4 Leeftijdsverdeling en man-vrouwverdeling van COVID-19 patiënten vanaf 4 mei 2020

Tabel 3: Leeftijdsverdeling van bij de GGD'en gemelde COVID-19 patiënten, van in het ziekenhuis opgenomen COVID-19 patiënten en van overleden COVID-19 patiënten vanaf 4 mei 2020[1,2]

| Leeftijdsgroep | Totaal gemeld | % | Ziekenhuisopname | % | Overleden | % |
|---|---|---|---|---|---|---|
| Totaal gemeld | 9803 | | 452 | | 807 | |
| 0-4 | 83 | 0.8 | 7 | 1.5 | 0 | 0.0 |
| 5-9 | 94 | 1.0 | 0 | 0.0 | 0 | 0.0 |
| 10-14 | 178 | 1.8 | 1 | 0.2 | 0 | 0.0 |
| 15-19 | 344 | 3.5 | 4 | 0.9 | 0 | 0.0 |
| 20-24 | 735 | 7.5 | 10 | 2.2 | 0 | 0.0 |
| 25-29 | 866 | 8.8 | 11 | 2.4 | 0 | 0.0 |
| 30-34 | 768 | 7.8 | 10 | 2.2 | 1 | 0.1 |
| 35-39 | 623 | 6.4 | 11 | 2.4 | 1 | 0.1 |
| 40-44 | 650 | 6.6 | 14 | 3.1 | 2 | 0.2 |
| 45-49 | 745 | 7.6 | 33 | 7.3 | 6 | 0.7 |
| 50-54 | 845 | 8.6 | 43 | 9.5 | 9 | 1.1 |
| 55-59 | 842 | 8.6 | 62 | 13.7 | 14 | 1.7 |
| 60-64 | 635 | 6.5 | 46 | 10.2 | 18 | 2.2 |
| 65-69 | 312 | 3.2 | 40 | 8.8 | 36 | 4.5 |
| 70-74 | 292 | 3.0 | 35 | 7.7 | 69 | 8.6 |
| 75-79 | 332 | 3.4 | 38 | 8.4 | 88 | 10.9 |
| 80-84 | 441 | 4.5 | 41 | 9.1 | 148 | 18.3 |
| 85-89 | 545 | 5.6 | 30 | 6.6 | 205 | 25.4 |
| 90-94 | 360 | 3.7 | 15 | 3.3 | 154 | 19.1 |
| 95+ | 112 | 1.1 | 1 | 0.2 | 56 | 6.9 |
| Niet vermeld | 1 | 0.0 | 0 | 0.0 | 0 | 0.0 |

[1] Sinds 1 juni kan iedereen zich met klachten laten testen. Toch is het aannemelijk dat niet alle COVID-19 patiënten getest worden. De werkelijke aantallen in Nederland zijn daarom waarschijnlijk hoger dan de aantallen die hier genoemd worden. Het werkelijke aantal COVID-19 patiënten opgenomen in het ziekenhuis of overleden is hoger dan het aantal opgenomen of overleden patiënten gemeld in de surveillance, omdat de surveillance gebaseerd is op de informatie op het moment van melding. Ziekenhuisopname na melding is niet altijd bekend. Aan het RIVM wordt niet gemeld wie hersteld is.

[2] De leeftijd van de gemelde patiënten is gemiddeld lager dan de leeftijd van de in het ziekenhuis opgenomen of overleden patiënten. Dit is een weergave van het testbeleid.

## C. Labeling of plasma

### Convalescent plasma

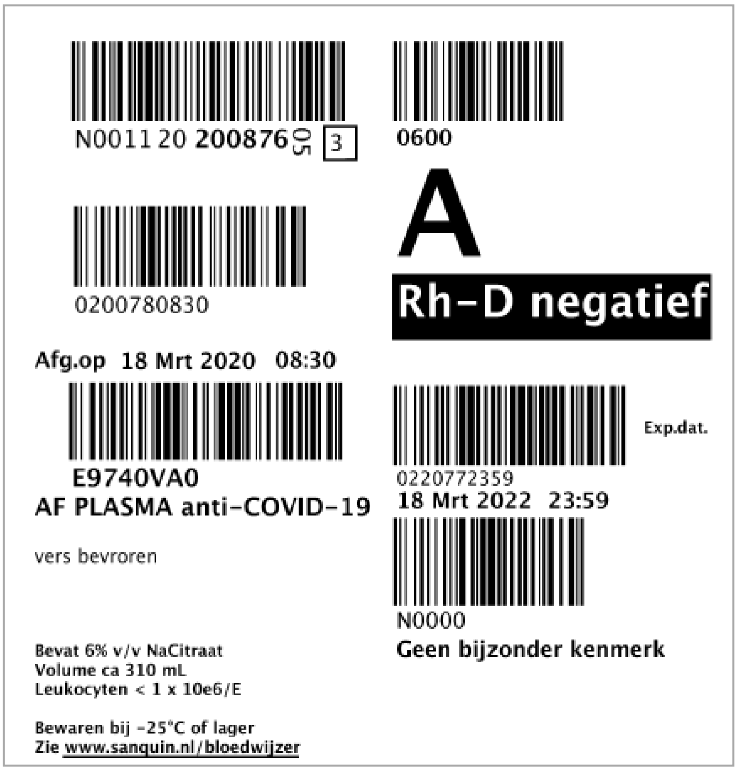

## D. Common Terminology Criteria for Adverse Events

The grading of adverse events will be done using the NCI Common Terminology Criteria for Adverse Events, CTCAE version *5.0*

## E. Donor criteria used by Sanquin

Additional donor criteria specific for COVID-19 convalescent plasma donors:
History of COVID-19 disease comfirmed by SARS-COV-2 PCR and asymptomatic for at least 14 days

**Standard plasma donor criteria**

**Inclusion criteria**

- ♦ Known ABO-Resus(D) blood group
- ♦ A screening for irregular antibodies with a titer ≤ 1:32
- ♦ Written informed consent regarding the plasmapheresis procedure
- ♦ Tested negative for HIV, HBV, HCV, HEV, HTLV and syfilis

**Exclusion criteria**

- ♦ Age <18 years or age >65 years (80 in donors who were already registered as a donor at Sanquin before the age of 65 years)
- ♦ Weight <50kg
- ♦ Medical history of heart failure
- ♦ History of transfusion with red blood cells, platelets or plasma after 01-01-1980
- ♦ History of organ- or tissue transplant
- ♦ A cumulative stay in the United Kingdom of ≥ 6 months in the period between 01-01-1980 and 31-12-1996
- ♦ A history of i.v. drug use
- ♦ Insulin dependant diabetes
- ♦ An underlying severe chronic illness (i.e. history of heart failure, cancer or stroke)
- ♦ Tested positive for HLA- or HNA-antibodies

## F. Hemovigilance

**Post treatment assessments - Adverse event assessment of plasma infusion:** Concerning the occurrence of possible plasma infusion related Adverse Events AE, SAE, AR, SUSAR will be actively screened for by the treating physician and the institutional haemovigilance employee(s), the latter is adviced to be a member of each institutional study team. .

**Specific organization of the plasma haemoviglance**

Most of transfusion related side-effects are respiratory symptoms [82]. Notwithstanding the additional importance of also other symptoms, it is especially important to carefull monitor all new or changing respiratory symptoms. Only then, it will become possible to determine if such symptoms are more likely caused by the course of COVID-19 or by the plasma transfusion.

The question of causality or imputability of (S)AEs occurring after plasma transfusion, in this respect can only be answered by an aggregate of much more information that in case of such an (S)AE needs to be collected. Examples of such information are the patient’s medical history, his/her previous transfusion history, vital signs before, during and after transfusion of the plasma, chest examination and imaging (before and after an expected transfusion reaction), but also the details of non-blood fluids given the fluid balance chart, details of co-medication and interventional medication given (including diuretics) and the response to measures and recovery of symptoms. Additionally, each transfusion reaction is followed by blood tests, imaging etc. according to national haemovigilance guidelines and are subsequently categorized, graded for severeity and imputability. All transfusion related reactions – irrespective of potential causality or not- in our study patients will be recorded and analysed and reported according to national blood transfusion and haemovigilance guidelines which are standard and operational in every Dutch hospital. This is done by the institutional haemovigilance employee of each hospital as they do for all blood products administered in Dutch hospitals.

Some characteristics and grades of known transfusion related side effects are:

**Allergic/Anaphylactic reaction:**
Mild, Transient flushing, urticaria or rash.
Moderate Wheeze or angioedema with or without flushing/urticaria/rash but without respiratory compromise or hypotension.
Severe Bronchospasm, stridor, angioedema or circulatory problems which require urgent medical intervention AND/OR, directly result in or prolong hospital stay, or anaphylaxis (severe, life-threatening, generalised or systemic hypersensitivity reaction with rapidly developing airway and/or breathing and/or circulation problems, usually associated with skin and and mucosal changes)

**Transfusion-related acute lung injury (TRALI)**

acute dyspnoea with hypoxia and bilateral pulmonary infiltrates during or within 6 hours of transfusion, in the absence of circulatory overload or other likely causes, or in the presence of human leucocyte antigen (HLA) or human neutrophil antigen (HNA) antibodies cognate with the recipient.

**Transfusion-associated circulatory overload (TACO)**

Should exhibit at least one required criterion* with onset during or up to 12 hours after transfusion, and a total of 3 or more criteria i.e. *A and/or B, and total of at least 3 (A to E)

***** Required criteria (A and/or B)

A. Acute or worsening respiratory compromise and/or

B. Evidence of acute or worsening pulmonary oedema based on:

• clinical physical examination, and/or

• radiographic chest imaging and/or other non-invasive assessment of cardiac function

Additional criteria

C. Evidence for cardiovascular system changes not explained by the patient's underlying medical condition, including development of tachycardia, hypertension, jugular venous distension, enlarged cardiac silhouette and/or peripheral oedema

D. Evidence of fluid overload including any of the following: a positive fluid balance; clinical improvement
following diuresis

E. Supportive result of a relevant biomarker, e.g. an increase in N-terminal-pro brain natriuretic peptide (NT-pro BNP) to greater than 1.5 times the pre-transfusion value

**Transfusion-Associated Dyspnoea or TAD** is characterised by respiratory distress within 24 hours of transfusion that does not meet the criteria for transfusion-related acute lung injury (TRALI) or transfusion-associated circulatory overload (TACO) or allergic reaction. Respiratory distress in such cases should not be adequately explained by the patient's underlying condition.

# Early Convalescent Plasma Therapy for high-risk patients with COVID-19 in primary care (the CoV-*Early* Study)

## *A randomized clinical trial*

**PROTOCOL**

| | | |
|---|---|---|
| **Version** | **:** | **6.0, Amendment 4** |
| **Date** | **:** | **10 June 2021** |
| **Principal investigator EMC** | **:** | **Bart Rijnders and Casper Rokx** |
| **Principal investigator LUMC** | **:** | **Jaap Jan Zwaginga** |
| **Sponsor** | **:** | **Erasmus MC** |
| **Funder** | **:** | **ZONMW** |
| **Clinical research organization** | **:** | **HOVON Data Center** |
| **ID** | **:** | **NL74972.078.20** |
| **Clinicaltrials.gov** | **:** | **NCT04589949** |

| Responsibility | Name | Affiliation/Address |
|---|---|---|
| Principal Investigators | Bart Rijnders and Casper Rokx<br><br>Jaap Jan Zwaginga | Erasmus MC<br><br>LUMC |
| Coordinating investigator | Bart Rijnders | Erasmus MC |
| Sponsor | Erasmus MC | |
| Subsidising party | ZONMW | |
| Independent expert | Dr. J.L.Nouwen | Erasmus MC |
| Co-investigators | Arvind Gharbharan<br>Carlijn Jordans<br>Casper Rokx<br>Mischa Huson<br>Yvonne Muller<br>Peter Katsikis<br>Jelle Miedema<br>Menno van der Eerden<br>Pierluigi Ciet<br>Marion Koopmans<br>Corine Geurtsvankessel<br>Rob J.C.G. Verdonschot<br>Henrieke Prins<br>J.G. van der Bom<br>S.P. Mooijaart<br>G.H. Groeneveld<br>C. Wife<br>Romy Meier<br>M.J. van Wordragen-Vlaswinkel<br>W.E. Benckhuijsen<br>Francis Swaneveld<br>Ellen van der Schoot<br>Hannelore Gotz | Erasmus MC<br>Erasmus MC<br>Erasmus MC<br>Erasmus MC<br>Erasmus MC<br>Erasmus MC<br>Erasmus MC<br>Erasmus MC<br>Erasmus MC<br>Erasmus MC<br>Erasmus MC<br>Erasmus MC<br>Erasmus MC<br>LUMC<br>LUMC<br>LUMC<br>LUMC<br>LUMC<br>LUMC<br>LUMC<br>Sanquin<br>Sanquin<br>GGD Regio Rijnmond |
| Statisticians | Dr. Nan van Geloven<br>Dr. Erik van Zwet<br>Grigorios Papageorgiou | LUMC<br>LUMC<br>Erasmus MC |
| Monitoring | Nephrology research unit | Erasmus MC |

| | | |
|---|---|---|
| Serious Adverse Events (SAEs) notification | HOVON Safety Desk<br>Bart Rijnders, Casper Rokx | HOVON Data Center<br>Erasmus MC |

| | |
|---|---|
| Collaborators at Erasmus MC | Dept of Internal Medicine and Infectious Diseases<br>Dr. Casper Rokx and dr. Mischa Huson<br><br>Dept. Of Immunology<br>Prof.dr. Peter Katsikis and dr. Yvonne Muller<br><br>Dept of pulmonary Medicine<br>Drs Jelle Miedema and dr. Menno van der Eerden<br><br>Dept. Of Virosciences<br>Dr. Corine GeurtsvanKessel and prof.dr Marion Koopmans<br><br>Dept. Of Biostatistics<br>Drs. Grigorios Papageorgiou<br><br>Dept. of Radiology and Nuclear Medicine<br>Dr. Pierluigi Ciet |
| Collaborators at LUMC | Dept. of Biomedical Data Sciences<br>Dr Nan van Geloven<br>Dr Erik van Zwet<br><br>Dept. of Clinical Epidemiology<br>Prof. dr. J.G. van der Bom<br><br>Dept of Internal Medicine, subdepartment of Gerontology and Geriatrics<br>Dr. S.P. Mooijaart<br><br>Dept. of Infectious disease and/or acute internal medicine<br>Dr. G.H. Groeneveld |
| Collaborators at Sanquin | Drs. Francis Swaneveld<br>Prof. dr. Ellen van der Schoot |
| Other Collaborators | Drs. Lotte Rokx-Niemantsverdriet en Drs. Jelle Struik<br>GCML Huisartsen centrum/HAGRO Rotterdam centrum<br>Mw. prof.dr. J. Gussekloo<br>Dept. of Public Health and Primary Care, LUMC<br>Prof. dr. T.J.M. Verheij<br>Julius Center for Health Sciences and Primary Care, UMCU |

| | |
|---|---|
| Study sites and PI | 1. Erasmus MC – Bart Rijnders/Casper Rokx<br>2. LUMC Jaap Jan Zwaginga<br>3. Maasstadziekenhuis – Jan den Hollander<br>4. Groene Hart Ziekenhuis – Faiz Karim<br>5. Spaarne Gasthuis – Robin Soetekouw<br>6. Antonius Nieuwegein – Elena Monica van Leeuwen-Sergarceanu<br>7. Amphia Ziekenhuis Breda, Ronald van Etten<br>8. Medisch centrum Leeuwarden, Linda Kampschreur<br>9. UMCG, Douwe Postma<br>10. Isala Zwolle, Jolanda Lammers<br>11. Hagaziekenhuis, Jean Louis, Kerkhoffs<br>12. OLVG, Janneke Stalenhoef |

**PROTOCOL SIGNATURE SHEET**

| Name | Signature | Date |
|---|---|---|
| **Sponsor or legal representative:**<br>*Erasmus MC*<br><br>**Head of Department:**<br>*Robin Peeters* | DocuSigned by:<br>Prof. Dr. R.P. Peeters<br>E47F73BC78EF43B... | 15-jun-21 |
| **Coordinating Investigator/Principal Investigator Erasmus MC:**<br>*Bart Rijnders* | DocuSigned by:<br>Dr. B.J.A. Rijnders<br>1D30E140F9CF485... | 16-Jun-21 |

## SITE INVESTIGATOR SIGNATURE PAGE

Local site name: ______________________________

______________________________ ____________

Signature of site investigator Date

______________________________

Printed name of site investigator

By my signature, I agree to personally supervise the conduct of this study in my affiliation and to ensure its conduct in compliance with the protocol, informed consent, IRB/EC procedures, the Declaration of Helsinki, ICH Good Clinical Practices guideline, the EU directive Good Clinical Practice (2001-20-EG), and local regulations governing the conduct of clinical studies.

## Scheme of study

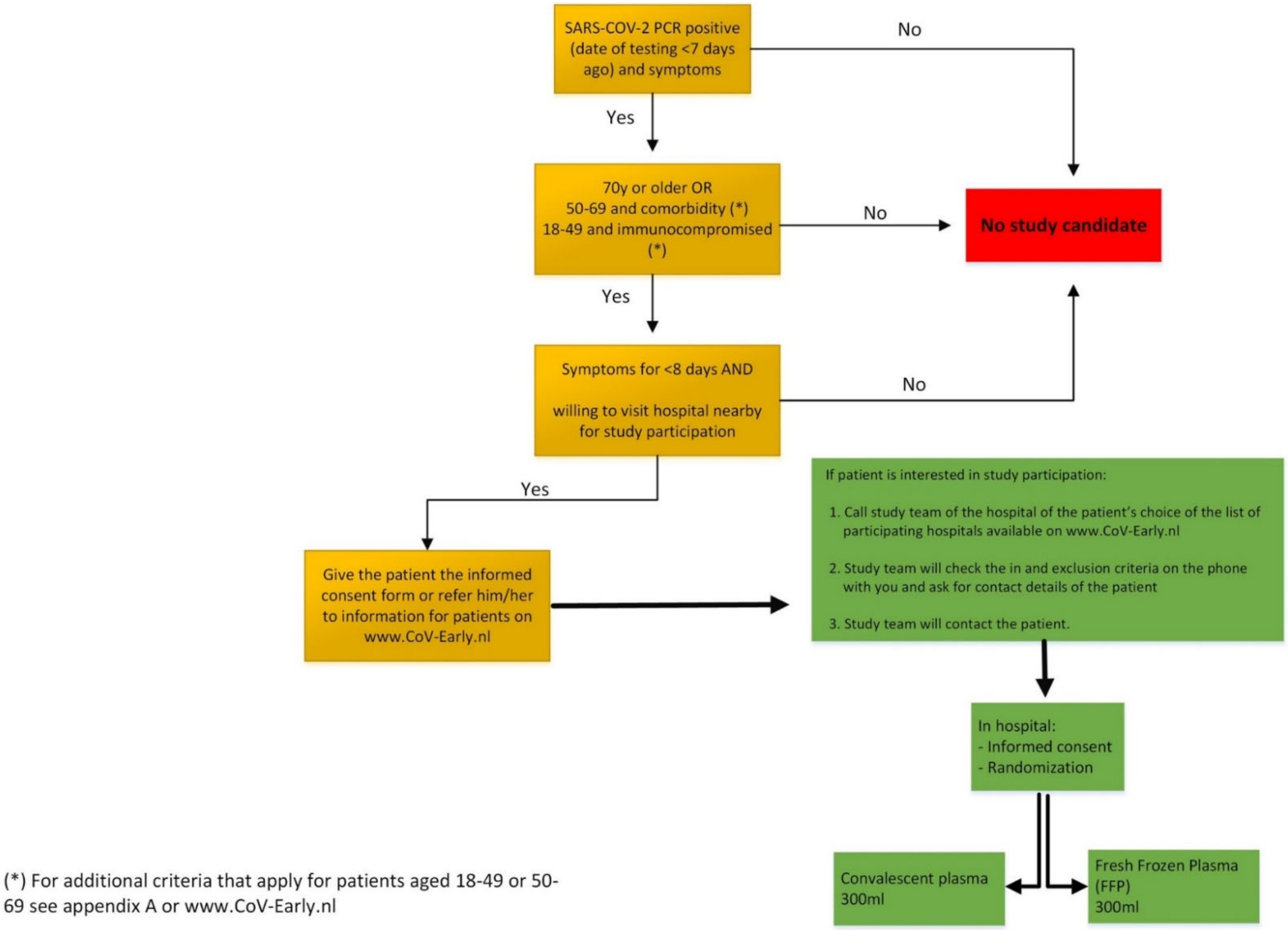

# TABLE OF CONTENTS

Page


1 INTRODUCTION AND RATIONALE ..... 15

2 STUDY OBJECTIVES ..... 17

2.1 Primary objectives ..... 17
2.2 Secondary (exploratory) objectives ..... 17

3 STUDY DESIGN ..... 18

4 STUDY POPULATION ..... 19

4.1 Population base ..... 19
4.2 Inclusion criteria ..... 20
4.3 Exclusion criteria ..... 20
4.4 Sample size calculation ..... 20

5 TREATMENT ..... 20

5.1 Intervention: infusion of plasma ..... 20
5.2 Use of co-intervention (if applicable) ..... 22
5.3 Escape medication (if applicable) ..... 22

6 PLASMA RETRIEVAL, ADMINISTRATION AND DETERMINATION OF SARS-COV-2 ANTIBODIES. 22

6.1 Assessment of SARS-CoV-2 antibodies in donor serum ..... 22

7 NON-INVESTIGATIONAL PRODUCT ..... 23

8 METHODS ..... 23

8.1 Endpoints ..... 23
8.1.1 Primary endpoint ..... 23
8.1.2 Secondary endpoints ..... 24
8.1.3 Exploratory endpoints: ..... 25
8.2 Randomisation, blinding and allocation ..... 25
8.2.1 Regulatory documentation ..... 25
8.2.2 Registration and randomization ..... 25
8.2.3 Unblinding procedures ..... 26
8.3 Study procedures ..... 26
8.3.1 Time of clinical evaluations ..... 26
8.3.2 Required investigations ..... 27
8.3.3 Specification of required investigations ..... 30
8.4 Withdrawal of individual patients from protocol treatment ..... 31
8.5 Replacemnts of individual subjects after withdrawal ..... 31
8.6 Follow up of patients withdrawn from protocol treatment ..... 31
8.7 Premature termination of the study ..... 31
8.8 Data collection ..... 32
8.8.1 Rapid reporting ..... 32

9 SAFETY ..... 33

9.1 Temporary halt for reasons of subject safety ..... 33
9.2 AEs and SAEs ..... 33
9.2.1 Adverse events (AEs) ..... 33
9.2.2 Serious adverse events (SAEs) ..... 33
9.2.3 Suspected unexpected serious adverse reactions (SUSARs) ..... 34
9.3 Annual safety report ..... 34
9.4 Follow-up of adverse events ..... 35
9.5 Data Safety Monitoring Board (DSMB) ..... 35

10 STATISTICAL CONSIDERATIONS ..... 36

10.1 Patient numbers and power considerations ..... 36
10.2 Primary endpoint analysis ..... 37
10.3 Secondary endpoint analysis ..... 37
10.4 Exploratory endpoints: ..... 39
10.5 Interim efficacy and safety analysis ..... 40
10.6 Stopping rules ..... 40

**11 ETHICAL CONSIDERATIONS ..... 41**

11.1 Regulations statement ..... 41
11.2 Recruitment and consent ..... 41
11.3 Objection by minors or incapacitated subjects ..... 42
11.4 Benefits and risks assessment. ..... 42
11.5 Compensation for injury ..... 42
11.6 Incentives ..... 43~~42~~

**12 ADMINISTRATIVE ASPECTS AND PUBLICATION ..... 43**

12.1 Handling and storage of data and documents ..... 43
12.1.1 Patient confidentiality ..... 43
12.1.2 Filing of essential documents ..... 43
12.1.3 Record retention ..... 44~~43~~
12.1.4 Storage and sharing of data ..... 44
12.1.5 Storage of samples ..... 44
12.2 Monitoring and quality insurance ..... 44
12.3 Amendments ..... 45
12.4 Annual progress report ..... 45
12.5 Temporary halt and (prematurely) end of trial report ..... 46~~45~~
12.6 Public disclosure and publication policy ..... 46

**13 STRUCTURED RISK ANALYSIS ..... 46**

13.1 Potential issues of concern ..... 46
13.2 Synthesis ..... 47

**14 REFERENCES ..... 48**

**A. RISK FACTORS FOR SEVERE COVID-19 ..... 50**

**B. RIVM DATA ..... 52**

**C. LABELING OF PLASMA ..... 53**

**D. COMMON TERMINOLOGY CRITERIA FOR ADVERSE EVENTS ..... 54**

**E. DONOR CRITERIA USED BY SANQUIN ..... 54**

**F. HEMOVIGILANCE ..... 55**

## LIST OF ABBREVIATIONS AND RELEVANT DEFINITIONS

A(D)R: Adverse (Drug) Reaction
ADCC: Antibody-Dependent Cell-mediated Cytotoxicity
ADE: Antibody-mediated enhancement of infection
AE: Adverse Event/Adverse Experience
ARDS: adult respiratory distress syndrome
BAL: Broncho Alveolar Lavage
CA: Competent Authority
CDC: United States Centers for Disease Control and Prevention
COI: Conflict of Interest
ConvP: Convalescent Plasma
COPD: Chronic obstructive pulmonary disease
COVID-19: Coronavirus Disease 2019
CRF: Case Report Form
CRP: C-reactive Protein
CTCAE: Common Terminology Criteria for Adverse Events
DMC: Data Management Center
DSMB: Data and Safety Monitoring Board
EUA: Emergency Use Authorization
ECG: Electrocardiogram
FFP: fresh frozen plasma
GCP: Good Clinical Practice
GDPR: General Data Protection Regulation
HAV: Hepatitis A virus
HBV: Hepatitis B virus
HCV: Hepatitis C virus
HIV: Human immunodeficiency virus
HTLV: Human T-cell lymphotropic virus
IC(F): Informed Consent (Form)
ICH: International Conference on Harmonization
ICU: Intensive Care Unit
IMP(D): Investigational Medicinal Product (Dossier)
IRB: Institutional review board
ISBT: International Society of Blood Transfusion
ISM: Independent Safety Monitor
IWRS :Interactive web response system
LDH: Lactate Dehydrogenase
LOS: Length of Stay
MERS: Middle East Respiratory Syndrome
METC: Medical Ethical Review Committee
MOF: mult Organ Failure
NA: Nuclear antibody
NP: Nasopharyngeal
NYHA: New York Heart Association
OP: Oropharyngeal
OS: Overall Survival
RT-PCR: Reverse Transcriptase Real-Time Polymerase chain reaction
PB: Peripheral Blood
PK: Pharmacokinetic
PRTN-50: The concentration of serum to reduce the number of plaques by 50% compared to the serum free virus
QP: Qualified person
SAE: Serious adverse event

SARS: Severe Acute Respiratory Syndrome
SARS-CoV-2: Severe Acute Respiratory Syndrome Coronavirus 2
SC: Sub-Cutaneous
SD: Stable Disease
SOFA: sequential organ failure assessment
SOP: Standard Operating Procedure
SUSAR: Suspected unexpected seriouw adverse reaction
TACO: Transfusion-associated circulatory overload
TRALI: Transfusion-related acute lung injury
UIP: Usual Interstitial Pneumonia
WHO: World Health Organisation
WMO: Wet Medisch-Wetenschappelijk Onderzoek met mensen

**Enrolled:** patients consented to participate until designated as a screen failure or until they discontinued the study or completed it.

**Randomized:** when a study plasma is ordered and released from the institutional blood bank for a patient or a patient is determined as control

**Screen Failures:** referred for inclusion, but then determined to be ineligible or withdraws before being enrolled

**Discontinued:** randomized having ordered for (or received) convalescent plasma or not (control), but then withdrawn by investigator or withdrawn consent

**Completed:** Subjects are considered to have completed when they are followed through day 28 or have had an adverse event or death occurred prior to day 28.

## SUMMARY

| | |
|---|---|
| **Rationale** | An effective, widely available, and safe treatment that can decrease the duration, severity and fatality of COVID-19 is urgently needed. Also, in the most affected regions the pressure on health care systems including ventilator support capacity can be a limiting factor for survival. Initial studies including from our group indicate that administering convalescent plasma containing high titers of neutralizing antibodies to COVID-19 patients who are already relatively late during the disease course after hospital admission is not effective, which can be explained by high titers of autologous antibodies present in patients. Thus, the antiviral capacity of convalescent plasma is hypothesized to be best postioned early in the disease course and in patients at increased risk for a severe disease course. If effective, any treatment that decreases the need for hospital admission is very valuable but so far, no COVID-19 treatment has been shown to prevent clinical deterioration when given before patients are admitted to the hospital. |
| **Study objectives** | **Primary objectives**<br>♦ To evaluate the efficacy, feasibility and safety following the administration of convalescent plasma (ConvP) as a therapy for outpatients diagnosed with COVID-19 at increased risk for an unfavourable clinical outcome and within 7 days after symptom onset.<br><br>**Secondary (exploratory) objectives**<br>♦ To evaluate the impact of 300mL convP on mortality<br>♦ To evaluate the impact of 300mL convP on hospital admission<br>♦ To evaluate the impact of 300 mL convP on admission to ICU |

| | |
|---|---|
| | ♦ To evaluate the impact of 300mL convP on duration of symptoms<br>♦ To evaluate the impact of 300mL convP in relation to the age, clinical frailty and immunocompromised state of the patient |
| **Study design** | This trial is a nationwide multicenter, double blind, randomized controlled trial in the Netherlands. Patients will be randomized between the transfusion of 300mL of convP versus regular fresh frozen plasma (FFP). |
| **Patient population** | Patients with COVID-19, confirmed by PCR or antigen testing and with less than 8 days of symptoms, age 70 or older OR 50-69 years with at least 1 additional risk factor for severe COVID-19 OR 18-49 and severely immunocompromised are eligible.<br>A total of 690 patients will be included.<br>Expected duration of accrual: 18-24 months |
| **Intervention** | 300mL of convP with a minimum level of neutralizing antibodies (see chapter 6).<br>Duration of follow up :Day 28 for the primary endpoint |
| **Main study endpoints** | **Primary endpoints**<br>♦ Highest disease status on the 5-point ordinal disease severity scale in the 28 days following transfusion of convP versus FFP.<br>**Secondary (exploratory)endpoints**<br>♦ Number (%) of deaths in the 28 days following transfusion of convP versus FFP<br>♦ Number (%) of hospital admissions in the 28 days following transfusion of convP versus FFP<br>♦ Number (%) of ICU admissions in the 28 days following transfusion of convP versus FFP<br>♦ Disease duration in days of symptoms in the 28 days following transfusion of convP versus FFP |

| | |
|---|---|
| | ♦ Age, clinical frailty scale and immunocompromised state stratified analysis of number of primary outcome. |
| Benefit and nature and extent of the burden and risks associated with participation | Benefits of this study may include a shorter disease course, a lower hospital admission rate and a decrease in mortality. In elderly patients, earlier recovery may also translate into being less frail after recovery. On the other hand, possible disease excacerbation by the antibodies has also been suggested. A blinded and randomized approach comparing FFP with convalescent plasma is therefore chosen, yielding unbiassed assessment of all endpoints. The risks of plasma infusion are small and widely known. These include transfusion reactions, transfusion related acute lung injury, circulatory overload and transmission of (unkown) transmittable diseases. |
| Planned interim analysis and DSMB | Continuous monitoring of the primary endpoint (every 50 patients or 3 months) with reporting to the DSMB in case of high posterior probabilities of either a positive effect (>97.5%) or a negative effect (>80%). |

# 1 Introduction and rationale

COVID-19 is causing substantial morbidity and mortality worldwide. Older people and people with comorbidities are most affected, and the ones most likely to be admitted to the hospital and to die of COVID-19. Despite this, intervention trials that focus on patients early on in the disease course, before hospital admission and in which a substantial number of older patients are included are very scarce. However, a treatment that turns out to be effective in the outpatient setting could result in a reduced hospital admission rate of the group of patients most affected and decrease the pressure on hospital and ICU beds. Given that patients with risk factors for admission also represent the ones who, once admitted to the ICU, will stay there for an average of 3 weeks, any intervention that prevents disease progression substantially will result in a major relieve of scarce healthcare systems during this pandemic.

A recently completed clinical trial demonstrated that *anti-inflammatory* therapy with dexamethasone significantly decreases overall mortality.[1] However, no effect was observed in patients not requiring supplemental oxygen therapy so the intervention is unlikely to be effective in the outpatient setting. In a randomized trial a shortened time to clinical recovery in patients treated with remdesivir was observed and comparable results were observed in a trial on interferon beta-1b, lopinavir–ritonavir, and ribavirin.[2] However, it remains to be seen if any of these antiviral therapies will decrease mortality and none of the interventions have been tested, or feasible to administer, in the outpatient setting.[3,4] Also, these drugs are not widely available and the rapid distribution to hospitals across the world is extremely challenging.[5] Therefore, other readily available, affordable and effective antiviral therapies are needed.

Convalescent plasma (ConvP), that contains virus neutralizing antibodies, could be an alternative treatment option for SARS-CoV-2 patients. A similar strategy has been pursued during the 2003 SARS and later MERS outbreaks.[6] Conclusive evidence for the effectivity of ConvP as a treatment for human coronavirus infections has yet not been documented in large randomized clinical trials. Preclinical research however indicated a protective effect of *hamster* ConvP when given to hamsters infected with SARS-CoV-2 early in the disease course. More recently *human* ConvP was shown to protect hamsters from COVID-19 disease as long as plasma with a high neutralizing antibody titer was used.[7] (B Rockx, personal communication). Although large volumes of ConvP can have indirect effects as well, we assume that key to the efficacy of ConvP through direct antiviral effect is the presence of high titers of virus neutralizing antibodies. Following this rationale, benefit can only be expected if it is administered to patients with as yet little or no autologous neutralizing antibodies. Although ConvP seems to be safe, no overall clinical benefit of ConvP therapy was observed in a prematurely interrupted randomized trial from China. That study though, was clearly too small to draw

definite conclusions.[8,9] Moreover, in this study, ConvP was administered extremely late in the disease course as patients had been symptomatic for 30 days on average when they received ConvP. The recent observations showing that close to 100% of patients have detectable neutralizing antibodies three weeks after symptom onset may explain the lack of a therapeutic effect in late disease.[10,11] All other evidence on the possible efficacy of ConvP comes from uncontrolled case series or with historical patients as comparator. [12-14] The Dutch ConCOVID trial, a randomized clinical trial that aimed to study the effect of 300ml of ConvP for hospitalized patients showed that already 80% of the patients had autologous neutralizing antibodies at the time of inclusion in the study. This was observed despite the fact that patients had been symptomatic for only 10 days. Not unexpectedly, patients who were still antibody negative at study inclusion had been sick for a shorter time (6 days) compared with antibody positive patients (10 days, $p<0.001$). These observations led to the discontinuation of the ConCOVID trial when 86 patients had been enrolled as the study team and DSMB considered it too unlikely that a large therapeutic effect would be observed after enrollment of all 426 planned patients[15]. In the ConCOVID trial ConvP was obtained from patients who had recovered from a RT-PCR confirmed COVID-19 for at least 2 weeks. As plasma with the highest neutralizing antibody titer available was used for each newly enrolled patient, the median neutralizing antibody titer in the ConvP that was administered was as high as 1:640, a titer observed in approximately 20% of the 115 donors who were tested. As the Dutch blood bank (Sanquin Blood Supply) has already collected ConvP from more than 1000 donors, the stock of ConvP with such high titers of neutralizing antibodies should therefore be large enough to allow for a large randomized clinical trial. Moreover, each high titer donor can donate up to 8 units of 300ml plasma in 4 weeks.

However, the decision to end the ConCOVID study does not mean that ConvP cannot have a small but clinically relevant therapeutic effect also for hospitalized patients. Clinically relevant differences in the characteristics of the early autologous antibodies in patients and the antibodies present in donor plasma may indeed exist. To this regard, it was shown that the level of fucosylation of anti-SARS-CoV2 antibodies in ConvP is higher than antibodies present during COVID-19 disease. Antibodies with low level of Fc-fucosylation have 50x fold higher affinity for FcγRIIIa on macrophages and therefore induce much higher cytokine production in these macrophages. ICU patients were found to have significantly higher levels of afucosylated anti-SARS-CoV2 antibodies (anti-spik antibodies) than the antibodies present in convalescent plasmas.[16] Theoretically, if the highly fucosylated donor antibodies outcompete the endogeneous antibodies they could induce a negative feedback on the cytokine storm. Two very large randomized trials continue to study ConvP for hospitalized patients and will be able to detect small therapeutic effects as well (NCT02735707, NTC04381936). Also, a real-time meta-analysis of ongoing and prematurally discontinued clinical trials in the USA and Europe has been initiated as well and could help in finding small but still clinically relevant effects of ConvP in hospitalized patients.[17]

The insights described above led to the design of the CoV-Early study described below where the principle aim is to employ ConvP in an outpatient setting early in the disease course to patients unlikely to have developed autologous virus neutralizing antibodies and study its potency to prevent deterioration and improve clinical condition. ConvP will specifically be targeted at a population at increased risk for a severe disease course, where elderly will be overrepresented and the effectiveness of convP might even be more pronounced to prevent disability and death.

# 2 Study objectives

## 2.1 Primary objectives

To evaluate the efficacy, feasibility and safety following the administration of ConvP as a therapy for outpatients diagnosed with COVID-19 at increased risk for an unfavourable clinical outcome and within 7 days after symptom onset.

**Efficacy:**
To determine if ConvP prevents COVID-19 disease progression when it is given early in the disease course.
To evaluate whether the use of ConvP has an impact on the long-term daily functioning, pulmonary condition, cellular and humoral immunity against SARS-CoV-2.

**Feasibility:**
To evaluate the feasibility of ConvP administration in the outpatient setting with its associated challenges (e.g. performing appropriate testing, infection prevention measures required, transportation of often frail and older patient to the hospital for plasma transfusion)

**Safety:**
To evaluate the safety of ConvP as compared with FFP administration regarding transfusion related reactions (in particular TRALI and TACO) and possible unwanted side effects of virus specific antibodies present in ConvP.

## 2.2 Secondary (exploratory) objectives

Evaluated in all patients:

- ♦ To evaluate the impact of 300mL convP on mortality

- To evaluate the impact of 300mL convP on hospital admission
- To evaluate the impact of 300 mL convP on admission to ICU
- To evaluate the impact of 300mL convP on duration of symptoms
- To evaluate the impact of 300mL convP in relation to the age, clinical frailty and immunocompromised state of the patient

Evaluated in subgroups of patients:

- To evaluate the impact of 300 mL convP on functional decline in patients aged 70 or older
- To evaluate the impact of 300 mL convP on the pulmonary condition and daily functioning
- To evaluate the duration of viral shedding in patients with and without convP and according to the presence of neutralizing antibodies at baseline
- To evaluate the impact of convP on the primary outcome in patients with and without neutralizing antibodies at baseline
- To evaluate the kinetics of infection and development of cellular and humoral anti-SARS-CoV-2 immune responses including memory immunity development.
- To evaluate the difference in efficacy of convP in relation to the duration of symptoms
- To evaluate the feasibility of recruiting COVID-19 patients, administering convP and perform study follow-up in an outpatient setting
- To evaluate cost-effectiveness of convP in an outpatient setting compared to routine care
- To evaluate the effect of ConvP therapy on the medium-term (3 months) immunity against SARS-CoV-2

# 3 Study design

Nationwide multicentre 1:1 randomized double-blind clinical trial to compare the efficacy and safety of ConvP compared to regular FFP in COVID-19 patients considered at high risk for disease progression but in whom symptoms have started less than 8 days before the patient is enrolled in the study.

Patients and investigators will be blinded for the intervention. The most straightforward masked placebo was considered FFP. It has the same color and temperature (plasma is thawed before use) as ConvP. The different labelling of ConvP and regular plasma will be masked by the local transfusion lab personal with the use of an opaque bag wrapped around the plasma bag.

Randomization will be done via a web-based system (ALEA) provided by HOVON. In this system the investigator can randomize the patient while the result of the randomization will not be disclosed to the investigator but to a designated unblinded person from the transfusion lab in the hospital.

The primary endpoint is  the highest disease status on the 5-point ordinal disease severity scale in the 28 days following transfusion of convP versus FFP. The study intervention is anticipated to improve the score on the 5-point ordinal scale (i.e. result in a shift from the higher towards the lower categories). In particular, the intervention is expected to lower the risk of a hospital admission, need for mechanical ventilation and death by 33%, translating in an odds ratio of 0.63 which we also assume for the shift in the other categories. More information is provided in chapter 8.1 and 10 below.

Disease status is measured with a 5-point ordinal scale in which
1 = Fully recovered (no symptoms) within 7 days after transfusion
2 = Continued symptoms attributable to COVID-19 on day 7 after transfusion
3 = Admitted to hospital but no invasive ventilation needed
4 = Admitted to hospital and invasive ventilation needed
5 = Death

# 4 Study population

## 4.1 Population base

Eligible patients are adults 18 years of age or older, with symptomatic COVID-19 disease for less then 8 days and have a high risk of disease progression as defined below.

Patients will be informed on the possibility of a referral for study participation by any physician aware of the study who is a treating physician of the patient. This will almost always be the general practitioner (GP) of the patient because outpatients in the Netherlands are typically tested by the GGD “teststraat” or by the GP him/herself. All positive test results of a SARS-CoV-2 test performed by the GGD are communicated to the patients and also to the patient’s GP as a standard of care procedure.

If the patient considers to participate and gives their physician the permission to inform the study team, the GP or another physician will inform the study team and the patient will be contacted by the study team, that provides further information, checks inclusion and exclusion criteria and if the patient is still interested invites the patient to the nearest participating hospital/site. In the hospital, written informed consent will be obtained. A minimum of 10 GP practices will be approached to provide feedback on the feasibility of this recruitment method.

### 4.2 Inclusion criteria

In order to be eligible to participate in this study, a subject must meet all of the following criteria:

- ♦ COVID-19, confirmed by PCR or CE-marked antigen test
- ♦ Symptomatic (e.g but not limited to fatigue, fever, cough, dyspnoe, loss of taste or smell, diarrhea, falls or confusion)
- ♦ 70 years or older OR 50-69 years and 1 or more of the risk factors described in Appendix A OR 18-49 and severly immunocompromised as described in Appendix A

The risk factors for a bad outcome of COVID-19 are described in Appendix A are based on results described in several recent observational studies[18-21]

### 4.3 Exclusion criteria

A potential subject who meets any of the following criteria will be excluded from participation in this study

- ♦ Life expectancy <28 days in the opinion of the treating physician
- ♦ Patient or legal representative is unable to provide written informed consent
- ♦ Symptomatic for 8 days or more at the time of screening. Please note that on the day the patient is randomized he/she is allowed to be on day 8 of symptoms
- ♦ COVID-19 related symptoms are already improving
- ♦ Being admitted to the hospital at the informed consent procedure
- ♦ Known previous history of transfusion-related acute lung injury
- ♦ Known IgA deficiency

### 4.4 Sample size calculation

See 10.1

## 5 Treatment

### 5.1 Intervention: infusion of plasma

ConvP will be infused that was collected from donors with a history of PCR proven symptomatic COVID-19 and who have recovered from COVID-19 for at least 14 days. The donors are recruited by Sanquin and will have to fulfill the standard plasma donor criteria used by Sanquin (Appendix E). Furthermore, only plasma from donors in whom a sufficiently high neutralizing antibody titer can be demonstrated will be used (see 6.1).

For all patients, all aspects considered necessary within routine care should be conducted before during and after the intervention.

Patients will be randomized with the use of blocks with variable length (block sizes up to 12 [2 4 6 8 10 12] into a group of

- 345 patients who will receive 300ml of thawed convP
- 345 patients who will receive 300ml of thawed non-convalescent plasma (FFP = fresh frozen plasma)

The investigator nor the patient will be informed about the treatment arm the patient was allocated to. Plasma will be administered according to the standard operating procedures of the hospital regarding the administration of blood products.

**Blood group compatibility of plasma:**

Plasma shall be transfused preferably ABO-identical. If the study plasma inventory does not hold an ABO identical then ABO-compatible plasma is acceptable. ABO compatibility is according to the following table:

*Table 1: Transfusion therapy with plasma: selection of the ABO phenotype of units to transfuse*

| ABO PHENOTYPE OF THE RECIPIENT | ABO PHENOTYPE OF UNITS TO TRANSFUSE (IN ORDER OF PREFERENCE) |
|---|---|
| O | O, A, B, AB |
| A | A, AB |
| B | B, AB |
| AB | AB |

Therefore, adherence to timing of study plasma transfusion according to the protocol has priority over ABO-identical transfusions.

Grade 1-2 allergic reactions are the most common possible side effects of plasma and are allowed according to institutional guidelines to be prevented or treated with antihistamins.

Risk based prevention or treatment of TACO is obligatory e.g. by treating pre-existing volume overload; additional administration of diuretics and or applying lower transfusion rates is allowed.

All plasma transfusions will be started after standard institutional identification of unit and patient. Similarly, after start of administration monitoring of vital signs and presence of care-personel need to be in place as are standard in Dutch hospitals and as described in Dutch Blood Transfusion guidelines.

### 5.2 Use of co-intervention (if applicable)

NOT APPLICABLE

### 5.3 Escape medication (if applicable)

NOT APPLICABLE

## 6 Plasma retrieval, administration and determination of SARS-CoV-2 antibodies

ConvP will be retrieved from donors at Sanquin Blood Supply according to their standard procedures for collection of fresh frozen plasma. Each donation 600 ml plasma will be collected in 2 bags of 300ml each. The donor will be asked (but is not obliged) to give plasma multiple times with at least a one-week interval. Plasma will be stored at minus 25 degrees Celsius or colder and tested for pathogens during routine procedures. There will be no pathogen reduction procedure. In contrast to FFP there is no quarantaine period before release of the Conv-P.

Anti-COVID-19 plasma has its own product code: E9740 = Apheresis CONVALESCENT PLASMA|Citrate/XX/≤-18°C|COVID-19. The two products of each donation will receive product codes E9740VA0 and E9740VB0 and will be labelled according to ISBT128 (see appendix C).

ConvP will be labeled according to appendix C. Every plasma unit will have a unique identification number, a so-called EIN (Eenheid Identificatie Nummer), by which the product can always be traced back to the donor. When plasma is administered, this number will be registered in the patient file.

### 6.1 Assessment of SARS-CoV-2 antibodies in donor serum

Sanquin will select convalescent plasma with a quantitative IgG ELISA that has been shown to correlate with virus neutralizing antibody titers (plaque reduction neutralization test, PRNT; gold standard for detection of neutralizing antibodies). The ELISA threshold will be set at 60 AU that is predictive for a PNTR50 titer above 1:160 according to the method developed by the RIVM laboratory

(a test calibrated in the European HORIZON2020 project Support-E). The neutralizing antibody titers will be measured on all convP that were selected based on the 60 AU ELISA screening criterium.

The PRNT50 assay will be performed at the RIVM and/or at viroscience lab in Erasmus MC. The department of virology at Erasmus MC has developed its own method for PRNT50 measurement. This PRNT test is done with the SARS-CoV-2 virus (German isolate; GISAID ID EPI_ISL 406862; European Virus Archive Global #026V-03883) and the methods were described previously.[10]

The RIVM and Viroscience methods have been compared and this comparison showed that the titers observed with the RIVM method are approximately 1 dilution lower than titers observed with the viroscience method. Therefore, only those plasma donors with a titer of 1/320 (Viroscience method) or 1/160 (Sanquin method) will be used.

Of each individual plasma also the antibodies against total spike protein and nucleocapsid will be quantified,[22] as well as the isotype (IgG, IgA and IgM) and subclass (IgG1, IgG3) will be quantified.

When the results of the phase 1 pharmacokinetics study become available, the data will be discussed within the entire study team. If the study team considers it likely that a change in the antibody titer cut-off is needed, a proposal of change will be discussed with the DSMB.

# 7 Non-investigational product

NOT APPLICABLE

# 8 Methods

## 8.1 Endpoints

### 8.1.1 Primary endpoint

- Highest disease status on the 5-point ordinal disease severity scale in the 28 days following transfusion of convP versus FFP.

  Disease status is measured with a 5-point ordinal scale in which
  1 = Fully recovered (no symptoms) within 7 days after transfusion
  2 = Continued symptoms attributable to COVID-19 on day 7 after transfusion
  3 = Admitted to hospital but no invasive ventilation needed

4 = Admitted to hospital and invasive ventilation needed

5 = Death

**Rationale behind primary endpoint:**

The CoV-Early primary efficacy endpoint is an ordinal outcome with the 5 categories as described above and evaluated on day 28. These endpoints encompass in our opininon the most relevant outcomes for patients infected with COVID-19, namely being sick as short as possible, not needing hospitalization, or when hospitalization is needed whether or not admitted to the ICU for mechanical ventilation is needed as well as the survival status one month after inclusion in the trial.

### 8.1.2 Secondary endpoints

*Evaluated in all patients:*

- ♦ Number (%) of deaths in the 28 days following transfusion of convP versus FFP.
- ♦ Number (%) of hospital admissions in the 28 days following transfusion of convP versus FFP
- ♦ Number (%) of ICU admissions in the 28 days following transfusion of convP versus FFP
- ♦ Disease duration in days of symptoms in the 28 days following transfusion of convP versus FFP
- ♦ Age and clinical frailty score and immunocompromised state stratified analysis of number (%) of primary endpoint following transfusion of convP versus FFP.

*Evaluated in subgroups of patients in which these data can be captured.*

Please note that no predefined sample size is in place for these subgroup analyses because patients and hospitals will be allowed to opt-in or opt-out for the substudies in which these data will be collected.

- ♦ Change in functional decline in patients over 70 years between inclusion, day 28 and month 6 following transfusion of convP versus FFP
- ♦ Change in functional respiratory imaging, FVC and DLCOc, validated QoL questionnaires between day 28, month 3, 6 and 12 following transfusion of convP versus FFP
- ♦ Change in proportion of detectable SARS-CoV-2 RT-PCR results at day 3, 7, 14 and 28 following transfusion of convP versus FFP.
- ♦ Change in number (%) of anti-SARS-CoV-2 specific B-cell and CTL memory responses at d1, d14, d28, m3, m6, m12 followin transfusion of convP versus FFP
- ♦ Number (%) of patients who fulfill the in- and exclusion criteria, number (%) of patients asked to participate in the study, number (%) of patients who do and do not participate and reasons to decline participation.

- Cost-effectiveness of convP compared to FFP will be assessed by calculating the mean costs of the intervention in relation to the relative healthcare savings of convP compared to FFP.

### 8.1.3 Exploratory endpoints:

- Analysis of primary endpoint following transfusion of convP versus FFP stratified by the presence of neutralizing antibodies at baseline, by the height of the neutralizing antibody titer in the plasma that was transfused and by symptom duration at baseline.
- Change in proportion of detectable SARS-CoV-2 RT-PCR results at day 3, 7, 14 and 28 following transfusion according to the presence of neutralizing antibodies at baseline
- The effect of ConvP therapy on the medium-term (3 months) immunity against SARS-CoV-2

## 8.2 Randomisation, blinding and allocation

### 8.2.1 Regulatory documentation

Required regulatory and administrative documents must be provided to the sponsor before enrolment of the first patient. This will always include an ethics committee approval for the investigational site. Each investigational site will be notified when all requirements are met, and enrolment can start

### 8.2.2 Registration and randomization

Eligible patients should be registered before start of treatment. Patients need to be registered in the electronic case record form. In principle the registration and randomization procedure can be modified in line with institutional specifics but should be similar to the following logistics:

All eligibility criteria will be checked with a checklist before the patient is included and randomized. Patients will be randomized with the use of an online randomization system (ALEA) using blocks of up to 12 in size {2 4 6 8 10 12}. Each patient will be given a unique patient study number (a sequence number by order of enrolment in the trial). Patient study number will be given immediately by the online registration database and confirmed by email. The result of the randomization will not be disclosed to the investigator but to a designated unblinded person from the transfusion lab in the hospital. The responsible physician will order the study plasma under the received study number.

### 8.2.3 Unblinding procedures

While the safety of patients should always take priority, maintenance of blinding is crucial to the integrity of a double-blind trial. The blind for a specific patient should only be broken when information about the patient's protocol treatment is considered necessary to manage Serious Adverse Events (emergency unblinding). Unblinding procedures should preferably be initiated only after consultation of the (co) principal investigator or his/her representative. Emergency unblinding can be done by the sponsor so please contact the sponsor if this would be needed.

## 8.3 Study procedures

### 8.3.1 Time of clinical evaluations

- ♦ Pre-screening
- ♦ Screening and baseline (day 1)
- ♦ Day 7, 14, 28 telephone contact for 5-point disease status (primary endpoint)
- ♦ Serum sample collection on month 3, 6 and 12 of the study (optional)
- ♦ Optional viro-immunological and clinical evaluations on 100 patients after additional consent as indicated in the tabel in 8.3.2

Viro-immunological evaluations will be performed in at least 100 and up to 400 patients (approximately half from both study arms) on the condition that the patient provides additional consent:

- ♦ Antibody titer measurement, one 6ml serumtube and fingerprick dried blot spot sample on day 1, 3, 7, 14, 28
- ♦ Nasopharyngeal swab for real-time quantitative PCR on day 1, 3, 7, 14, 28
- ♦ Immune response evaluation; four 9mL natrium heparine tubes, one 6mL EDTA tube and two 6mL serum tubes, on day 1, 14, 28 and on month 3, 6, 12

Additional clinical evaluations for patients who provided additional consent

- ♦ At least 100 and up to 400 patients aged 70 or older (approximately half from both study arms): Geriatric functional evaluation, by telephone contact, on day 1, 28, at month 3 and 6.
- ♦ At least 100 and up to 400 patients (approximately half from both study arms): Long-term lung damage with a lung function test at month 3 and a low-dose CT on month 3 and a questionnaire on month 3. If the pulmonologist considers it necessary to continue follow up after month 3, a follow up CT-scan, lung function test and questionnaires will be done at these timepoints at 6 and 12 months (following routine care as determined by the NVALT)

#### 8.3.2 Required investigations

Required investigations at entry, during treatment and during follow up:

| | **Prescreening with referring physician (*)** | **Screening Day 1** | **Baseline Day 1** (immediately following screening) | **D3** | **D7** | **D14** | **D28** | **M3** | **M6** | **M12** |
|---|---|---|---|---|---|---|---|---|---|---|
| Eligibility check (*) | x | x | | | | | | | | |
| Informed consent | | x | | | | | | | | |
| Lab (blood group, antibody) testing) + nasopharynx swab for PCR | | X(&) | | | | | | X7 | X7 | X7 |
| Plasma infusion | | | x | | | | | | | |
| Transcutaneous O2 saturation without administration of supplemental oxygen | | | x | | | | | | | |
| Registration of baseline characteristics and comorbidities defined in Appendix A | | x | | | | | | | | |
| Telephone contact (1) | | | | | x | x | x | | | |

The items below are optional and only for patients and sites who choose to participate in certain sub-studies(x)

| | **Prescreening with referring** | **Screening Day 1** | **Baseline Day 1** (immediately following | **D3** | **D7** | **D14** | **D28** | **M3** | **M6** | **M12** |
|---|---|---|---|---|---|---|---|---|---|---|
| Antibody titer measurement (2) | | | x | x | x | x | x | | | |
| Nasopharyngeal swab (3) | | | x | x | x | x | x | | | |
| Immune response evaluation (4) (For cellular immunity sub-study sites) | | | x | | | x | x | x | x | x |
| Geriatric functional evaluation (5) | | | x | | | | x | x | x | |
| Pulmonology assessments (6) (For lung damage sub-study sites) | | | | | | | | x | x($) | x($) |
| CT lungs (6) (For lung damage sub-study sites) | | | | | | | | x | x($) | x($) |
| QoL questionnaires (6) (For lung damage sub-study sites) | | | | | | | x | x | x($) | x($) |

(&) The test will be performed centrally. The serum and swab will be pick-up by Sanquin (specific instructions will be provided)

(*) During a telephone call with the referring physician, the in -and exclusion criteria are checked.

(1) Disease status is checked by designated person from the project group and registered as

1= Recovered with no symptoms on date xx/xx/xxxx) OR

2= Continued symptoms attributable to COVID-19 OR

3= Admitted to hospital but no invasive ventilation needed OR

4= Admitted and invasive ventilation needed OR

5= Death

If admitted, also additional anti-SARSCOV2 treatment is registered.

(x) In grey are the optional investigations for selected patients and selected study sites;

(2) Antibody titer measurement: 1 serum tube of 6mL is collected and stored at minus 70 on site

(3) The SARS-COV-2 PCR is done in the virology lab at the study site. Therefore, this can only be done if the local virology lab is able to report on the RT- PCR Cycle Treshold values of the PCR.

(4) Immune response evaluation: 4 natrium heparine tubes of 9mL each, 1 EDTA tube of 6mL, 2 serum tubes of 6mL, on day 1, 14, 28 and **optional** on month 3, 6, 12

These samples have to arrive at Erasmus MC in Rotterdam before 13h00. Samplings is therefore only possible between 8 and 11h00 and the study team should be contacted 48hrs in advance to plan a pickup

(5) Geriatric functional evaluation will be done by the LUMC study team via a telephone contact with the patient and include KATZ-ADL, LAWTON-IADL, living situation and EQ5D.

(6) Lung function is done at d28 and month 3, questionnaire is done at day 28, month 3, a low-dose CT is done 3 months after inclusion in the study. If it is abnormal, the investigator and patient will decide if further follow-up is planned according to NVALT guideline at month 6 and month 12 which includes a questionnaire and a CT/lung function outside the context of the study. The daily functioning questionnaires SGRQ and KBUILD, and the QoL EQ5D questionnaire will be evaluated in consenting individuals.

(7) Optional blood collection: 1 serum and 1 plasma and 4 natrium heparine tubes of 9ml each

Notes on the pulmonary assessments:

($) Will only be done if the investigator decides that further follow-up after the 3-month visit is needed as part of routine care

### 8.3.3 Specification of required investigations

**Inclusion**

**Medical history**

- How was the patient referred for the study
- Sex and age
- Etnicity
- Socio-economic status
- BMI at inclusion into the study
- Date of first day of symptoms of SARS-CoV-2 infection
- Underlying medical illnesses at the time of first day of SARS-CoV-2 RT-PCR positive
- Charlson Comorbidity Index
- Clinical Frailty Scale for patients aged 70 or older
- If available from previous testing in the preceding 72 hours; CRP
- If available from previous testing in the preceding 7 days; SARS-CoV-2 RT-PCR Ct value

**Physical examination at baseline**

- Transcutaneous O2 saturation without administration of supplemental oxygen
- Respiratory rate, blood pressure, pulse, temperature

**Lab**

- Bloodgroup
- Antibody testing at t=0: Presence of anti-SARS-CoV-2 RBD-protein and anti-SARS-CoV-2 nucleocapsid protein antibodies will be tested in a highly sensitive briding assay (Wantei test or equivalent test). In the positive samples the antibodies will be specified and quantified in IgG, IgA and IgM ELISA's against RBD and Nucleocapsid protein
- Nasopharynx swab for SARS-CoV-2 RT-PCR testing
- Is the patient is able to produce sputum: Sputum sample will be collected and stored for future research

**Follow-up**

**Clinical**

- 5-point disease severity status (see primary endpoint) by telephone on day 7, 14 and 28
- Any other anti-SARS-CoV-2 treatments that were given

**Laboratory and pulmonary (after additional consent by the patient)**

- Antibody testing using finger prick blood (sampled in outpatient setting or at home): Antibodies against RBD and nucleocapsid (IgG, IgM and IgA)
- SARS-CoV-2 RT-PCR Ct values
- Viral culture
    - Cellular and humoral anti-SARS-CoV-2 immunity (Nucleocapsid and Spike protein specific CD4+ cells using previously selected peptide pools,[23] CD8+ cells using classI tetramaeric complexes as presently being developed at Sanquin,[24] antigen specific B cells by FCM using tetrameric spike and nucleocapsid complexes[25] geriatric evaluation with questionnaires
- CT-lungs
- Spirometry: FEV1, FVC, DLCOc in liter and procent predicted for age
- Lung evaluation with questionnaires

## 8.4 Withdrawal of individual patients from protocol treatment

Patients should be withdrawn from protocol treatment if any of the following criteria for withdrawal are met:

♦ Potentially life-threatening transfusion reaction during plasma infusion.

Patients can leave the study at any time for any reason if they wish to do so without any consequences. The investigator can also decide to withdraw a patient from protocol treatment for urgent medical reasons. Patients who are withdrawn from protocol treatment will receive medical care according to local practice. Urgent medical reasons can force unblinding the treatment by the investigators (e.g. when a high clinical suspicion of unwanted plasma contamination by pathogens is suspected). Patients will remain in the analysis then.

## 8.5 Replacements of individual subjects after withdrawal

## 8.6 Follow up of patients withdrawn from protocol treatment

Patients who are withdrawn from treatment for other reasons than death will be followed for disease status as described in 8.3.2 if they consent. SAE information will be collected as described in 9.2.

## 8.7 Premature termination of the study

The sponsor may decide to terminate the study prematurely based on the following criteria:

♦ There is evidence of unacceptable risks for study patients (i.e. safety issue);

♦ There is ample evidence of efficacy

- There is reason to conclude that continuation of the study cannot serve a scientific purpose

The sponsor will promptly notify all concerned investigators, the DSMB, the ethics committee(s) and the regulatory authorities of the decision to terminate the study. The sponsor will provide information regarding the timelines of study termination and instructions regarding treatment and data collection of enrolled patients.

## 8.8 Data collection

Data will be collected on electronic case report forms (CRF, ALEA) to document eligibility, safety and efficacy parameters, compliance to treatment schedules and parameters necessary to evaluate the study endpoints. Data collected on the CRF are derived from the protocol and will include at least:

- Inclusion and exclusion criteria;
- Baseline status of patient including medical history and stage of disease;
- Timing and dosage of protocol treatment;
- Any other parameters necessary to evaluate the study endpoints;
- Survival status of patient;

Each CRF page will be identified by a trial number, and a combination of patient study number (assigned at registration) and hospital name.
The e-CRF will be completed on site by the investigator or sub-investigator or an authorized staff member. All CRF entries must be based on source documents.

Data collected on the CRF will be verified for accuracy. If necessary, queries will be sent to the investigational site to clarify the data on the CRF. The investigator should answer data queries within the specified timeline.

### 8.8.1 Rapid reporting

To enable continuous monitoring as described in chapter 9.5, all sites shall submit the results of the ordinal outcome (primary endpoint) in the eCRF within 7 days after the patient has passed the 28 days follow up time point. This will enable the described continuous monitoring and with it the analysis of possible superiority or inferiority of one of the treatment arms.

# 9 Safety

## 9.1 Temporary halt for reasons of subject safety

In accordance to section 10, subsection 4, of the WMO, the sponsor will suspend the study if there is sufficient ground that continuation of the study will jeopardise subject health or safety. The sponsor will notify the accredited METC without undue delay of a temporary halt including the reason for such an action. The study will be suspended pending a further positive decision by the accredited METC. The investigator will take care that all subjects are kept informed.

## 9.2 AEs and SAEs

### 9.2.1 Adverse events (AEs)

Adverse events are defined as any undesirable experience occurring to a subject during the study, whether or not considered related to the trial procedure/ the experimental intervention.

However, only AE at least possibly related to the plasma transfusion will be registered. AE grade 1 will not be reported.

AE from plasma infusion until 30 days following plasma will be reported and are defined in detail in appendix F. They will only be reported if considered at least possibly related to the plasma infusion. Adverse events have to be reported on the Adverse Events CRF. Adverse events will be scored according to the National Cancer Institute (NCI) Common Terminology Criteria for Adverse Events (CTCAE), version 5.0 (see appendix D).

### 9.2.2 Serious adverse events (SAEs)

A serious adverse event is any untoward medical occurrence or effect that

- results in death;
- is life threatening (at the time of the event);
- requires hospitalisation or prolongation of existing inpatients' hospitalisation;
- results in persistent or significant disability or incapacity;
- any other important medical event that did not result in any of the outcomes listed above due to medical or surgical intervention but could have been based upon appropriate judgement by the investigator.

An elective hospital admission will not be considered as a serious adverse event.

The registration of SAE will be limited to the following:

- Death
- Life threatening transfusion reactions
- Hospitalisation or prolongation of existing inpatients' hospitalisation;

However, the following events do not require to be reported as a serious adverse event:

- Hospitalization for protocol therapy administration. Hospitalization or prolonged hospitalization for a complication of therapy administration will be reported as an SAE.
- Hospitalization for diagnostic investigations (e.g., scans, endoscopy, sampling for laboratory tests, bone marrow sampling) that are not related to an adverse event. Hospitalization or prolonged hospitalization for a complication of such procedures remains a reportable serious adverse event.
- Prolonged hospitalization for technical, practical, or social reasons, in absence of an adverse event.
- Hospitalization for a procedure that was planned prior to study participation (i.e. prior to registration or randomization). This should be recorded in the source documents. Prolonged hospitalization for a complication of such procedures remains a reportable SAE.

Serious Adverse Events (SAEs) will be reported from the moment of plasma infusion according to protocol until 30 days following infusion.
SAEs (including death) occurring after 30 days and/or after infusion should also be reported if considered at least possibly related to the plasma infusion.
The investigator will report the SAEs to the HOVON Safety Desk **within 24 hours** after obtaining knowledge of the events using the SAE report form provided.

HOVON Safety Desk will report the SAEs through the web portal ToetsingOnline to the accredited METC that approved the protocol, within 7 days of first knowledge for SAEs that result in death or are life threatening followed by a period of maximum of 8 days to complete the initial preliminary report. All other SAEs will be reported within a period of maximum 15 days after the sponsor has first knowledge of the serious adverse events.

#### 9.2.3 Suspected unexpected serious adverse reactions (SUSARs)

NOT APPLICABLE

### 9.3 Annual safety report

NOT APPLICABLE

## 9.4 Follow-up of adverse events

All AEs and SAEs will be followed until they have abated, or until a stable situation has been reached. Depending on the event, follow up may require additional tests or medical procedures as indicated, and/or referral to the general physician or a medical specialist.
SAEs need to be reported till end of study within the Netherlands, as defined in the protocol

## 9.5 Data Safety Monitoring Board (DSMB)

A data safety monitoring board will be implemented to review the available study data regarding SAEs observed during the trial, review the safety of the participants on a regular basis and recommend the study team regarding the further conduct of the study. The Bayesian design allows for multiple evaluations of the data without the need to adjust the global alpha level for multiplicity. Therefore, the posterior distribution of the primary efficacy parameter (common log odds ratio) will be monitored in an unblinded fashion by one of the trial statisticians and reported to the DSMB after every 50 patients or 3 months (whatever comes first). If the posterior probability of a positive effect (OR lower than 1) reaches 97.5% or the posterior probability of a negative effect (OR greater than one) reaches 80%, the DSMB will be informed and advise the study team about the continuation of the study.

**Importantly**, to make this possible, all sites shall submit the results of the ordinal outcome (primary endpoint) in the eCRF within 7 days after the patient has passed the 28 days follow up time point. This will enable the described continuous monitoring and with it the analysis of possible superiority or inferiority of one of the treatment arms.

The DSMB will receive the first review of SAEs after the outcomes of 20 patients are available. Then this will be continued, after the outcomes e.g. of every 50 patients at the discretion of the DSMB's wishes. The DSMB will consist of a biostatistician (prof. Bossuyt), an infectious diseases specialist (dr. JL Nouwen), a medical ethicist (prof MC de Vries)

**Missing Data**

Although the covariates to be used for the analysis of the primary endpoint are unlikely to be missing, this is always a possibility. At the time of planned analyses, we will assess the data for missingness. If necessary multiple imputation will be used to impute missing covariate data. Both the results of the imputed and non-imputed data will be presented.

If patients are lost to follow-up before day 28, the highest score that was measured before the patient was lost to follow-up will be used for the analysis of the day 28 5-point ordinal scale score (highest score observed carried forward).

# 10 Statistical considerations

## 10.1 Patient numbers and power considerations

The study intervention is anticipated to improve the score on the 5-point ordinal scale (i.e. result in a shift from the higher towards the lower categories). In particular, the intervention is expected to lower the risk of a hospital admission, need for mechanical ventilation and death by 33%, translating in an odds ratio of 0.63 which we also assume for the shift in the other categories.

With the inclusion criteria mentioned above, we are targeting a patient population in whom the risk of hospital admission or death is estimated to be at least 20% in the 28 days following transfusion. The primary endpoint analysis will be based on an adjusted version of the WHO 10-point COVID-19 disease severity score ordinal scale specifically developed for this study as the full WHO 10-point ordinal scale is not suited to study outpatients. Furthermore, we are using an endpoint that allows for a large pragmatic trial design by which the data can be collected by a telephone interview on day 7, 14 and 28 if the category 3 to 5 is not observed in the patient.

1 = Recovered (no symptoms) within 7 days after transfusion
2 = Continued symptoms attributable to COVID-19 on day 8 after transfusion
3 = Admitted to the hospital but no invasive ventilation needed
4 = Admitted to the hospital and invasive ventilation needed
5 = Death

The event rates that we estimate for each of the groups are based on RIVM data (appendix B), on a Chinese study in which the COVID-19 clinical evolution in a large group of patients are described (also in the subgroup of 50-59 in the supplemental data of that paper) and on data from the ConCOVID study.[15,26] For the patients aged 18-49, the higher risk of a complicated COVID19 disease course is based on published data on COVID19 in transplant patients and patients with B-cell deficiency[27-29]

Assuming a common odds ratio of 0.63, using a two-sided test in a proportional odds model, assuming 15% attrition (loss to follow up and admission events occurring before inclusion) a sample size of 690 is needed for a study with a power of 80% (923 for 90% power).

| | Recovered within 7 days | Continued symptoms beyond 7 days | Hospital admission, not invasive ventilation | Hospital admission with invasive ventilation | Death |
|---|---|---|---|---|---|
| Control (FFP) | 22% | 63% | 3% | 3% | 9% |
| Treatment (ConvP) | 31% | 59% | 2.1% | 2.1% | 5.9% |

## 10.2 Primary endpoint analysis

The highest disease status on the 5-point ordinal scale in the 28 days following transfusion will be analyzed using the intention-to-treat principle. Occasionally several hours may pass between randomization and the time that plasma becomes ready for transfusion on the ward. Indeed, an intravascular access needs to be placed and blood group matching of plasma and patient takes time. Also, COVID-19 occasionaly has a rapidly progressive disease course in the population under study. Therefore, sporadically patients may be randomized but will not receive the plasma transfusion because upon clinical reevaluation the local investigator may decide that hospital admission has become indicated. These randomized but untreated patients will not be included in the intention to treat population.

A Bayesian proportional odds model will be used for the analysis of the highest disease status on the 5-point ordinal scale in the 28 days following transfusion. Apart from treatment, the following factors will be included in the model: age, sex, BMI, immunocompromised or not according to table 2, number of comorbidities, O2 saturation at inclusion, and duration of symptoms at inclusion. The specification of the prior distributions for the parameters in the model is as follows:

- Efficacy will be assessed using a skeptical prior. More specifically for the common log odds ratio of treatment we will use a normal distribution with zero-mean and a standard deviation of 0.42. This ensures that the prior probability of the OR to be greater than 2 is 0.05 and that the prior probability of the OR to be less than ½ is 0.05, in order to quantify our prior belief that a very large effect might be unlikely.

- A Dirichlet (1, 1, 1, 1, 1) non-informative prior will be used for the 5 categories of the ordinal outcome in the control arm.

- Vague priors will be used for the log odds ratios of the covariates included in the model: age, sex, BMI, O2 saturation at inclusion, immunocompromised or not according to table 2, number of comorbidities and duration of symptoms at inclusion. More specifically, normal distributions with mean zero and standard deviation of 0.5.

## 10.3 Secondary endpoint analysis

1. The adjusted hazard ratio of death over the follow-up period of 28 days between convP and FFP will be estimated using a multivariable proportional hazards model.
2. The adjusted cause-specific hazard ratio of hospital admission over the follow-up period of 28 days between convP and FFP will be estimated using a multivariable proportional hazards

model, censoring at death. To describe the absolute risk of hospital admission, cumulative incidence curves for hospital admission in both convP and FFP groups will be presented.

3. The adjusted cause-specific hazard ratio of ICU admission over the follow-up period of 28 days between convP and FFP will be estimated using a multivariable proportional hazards model, censoring at death. To describe the absolute risk of ICU admission, cumulative incidence curves for hospital admission in both convP and FFP groups will be presented.
4. The difference in median duration of symptoms over the follow-up period of 28 days between convP and FFP will be estimated using quantile regression on the subset of alive patients.
5. Differences in the effect of treatment (quantified by the adjusted OR for the primary endpoint), will be investigated by age, being immunocompromised or not according to table 2 and clinical frailty score between convP and FFP. For this, the proportional odds model from the primary endpoint will be extended with an interaction term between treatment and age (as a continuous variable), an interaction term between treatment and immunocopromised status and an interaction term between treatment and clinical frailty score to capture differences in the effect of treatment between patients who are immunocompromised or not according to table 2 and per unit change in age and clinical frailty score.

In all above analyses (1-5) we will adjust for the following covariates, if the outcome frequency allows: age, sex, BMI, O2 saturation at inclusion, immunocompromised or not according to table 2, number of comorbidities and duration of symptoms at inclusion. In case of a low observed event per variable (EPV) rate (e.g. EPV < 10) a subset of these factors will be included in the model.

Evaluated in subgroups of patients:

1. The impact of 300ml convP on functional decline (frailty, cognitive functioning) in patients aged 70 or older. Functional decline will be measured with the use of the following questionnaires: Katz-ADL, Lawton-IADL, living situation and EQ5D at d1, 28, m3, m6. Data from each questionnaire will be analyzed with appropriate regression models according to outcome distribution and its features.

2. FVC (% predicted of normal for age) and lung diffusion (DLCOc, % predicted of normal for age) are continuous quantitative measure of lung function and structural lung damage is assessed by a continuous quantative measure using a validated automated functional respiratory imaging (FRI) to evaluate the patient's airway and lung geometry (Fluidda®, ref https://www.fluidda.com/publications/). Mixed-effect models will be constructed to evaluate changes in FVC, DLCOc, FRI over time and compare these changes between the treatment groups (interaction between time and treatment group). We will adjust for age, sex, BMI, o2

saturation at inclusion, being immunocompromised or not according to table 2, number of comorbidities and duration of symptoms at inclusion.

3. The probability of SARS CoV 2 genome detected by RT PCR over time (measurements at day 3, 7 and 14 and 28) will be compared between convP and FFP. For this analysis a Generalized Estimating Equations (GEE) model will be used with an interaction term between time and treatment and an interaction term between treatment and presence of neutralizing antibodies at inclusion. The latter interaction terms will allow to assess differences in the OR between people with and without neutralizing antibodies at inclusion (exploratory analysis).

4. Evolution of anti-SARS-CoV-2 memory humoral and cellular immunological memory measured as anti-SARS-COV2 specific B-cell and CD8Tcell responses. A mixed effects model will be used for the analysis of serial measurements adjusted for age and sex, covid disease severity (outpatient or hospital admission or ICU admission <28 days), use of immunosuppressive medication, and having an immune disorder.

5. The mean incremental cost effectiveness ratio will be calculated by the costs of the invervention divided by the costs saved based on prevented care by convP compared to FFP. The following parameters will be taken into account in this analysis; QOL, mortality, hospital days on ward and on ICU and for patients with a payed job at the time of COVID disease onset also the loss-of-income data up to 6 months after inclusion.

## 10.4 Exploratory endpoints:

Differences in the effect of treatment (quantified by the adjusted OR for the primary endpoint), will be investigated by the presence of neutralizing antibodies at baseline, by the height of the neutralizing antibody titer in the plasma that was transfused and by the duration of symptoms at baseline. For this, the proportional odds model from the primary endpoint will be extended with an interaction term between treatment and presence of neutralizing antibodies, an interaction term between treatment and titer of antibodies in the plasma that was transfused and an interaction term between treatment and duration of symptoms (the latter two as continuous variables) to capture differences in the effect of treatment with these factors.
`

The effect of ConvP therapy on the medium-term (3 months) immunity against SARS-CoV-2 will be evaluated by comparing SARS-CoV-2 recepter binding domain antibody titers and T-cell immunity against SARS-CoV-2 (method do be decided) between both groups. Only patients that are not immunocompromised and that have not yet been vaccinated at 3 months will be included in this

analysis. The way the T-cell results will be analyzed will be decided upon when the method of T-cell immunity measurement has been chosen.-Antibody titer amount will be used to measure medium-term (3 months) immunity against SARS-CoV-2. A linear regression model with antibody titer as the dependent variable will be used to estimate the effect of ConvP on the medium-term (3 months) immunity against SARS-CoV-2. The model will be further adjusted for antibody titer at baseline, age and sex.

## 10.5 Interim efficacy and safety analysis

See chapter 9.5

## 10.6 Stopping rules

See DSMB chapter 9.5 above. In addition, circumstances that may cause an DSMB's advise to terminate the study include, but are not limited to:

- newly emerging effective treatment for Covid-19 which would substantially change the risk- benefit assessment of the investigational approach in this trial compared to alternative options.
- Determination of unexpected, significant, or unacceptable risk to patients
- Failure to recruit patients at an acceptable rate
- Insufficient adherence to protocol requirements
- Insufficient complete and/or evaluable data
- Serious non-compliance to ICH-GCP standards
- Plans to modify, suspend or discontinue the development of the study drug
- Unexpected, significant, or unacceptable risk to patients

Written notification documenting the reason(s) for study termination advice will be provided to the investigator by the relevant party (DSMB, interim analysis statistician, monitoring agencies).

a. Institutional investigators may terminate their centre's participation to the study. If this occurs, they should provide a written statement of the reasons for terminating participation but remain obligated to manage all included patients according to the study protocol and the signed PIFs and should provide all relevant data to the Sponsor (i.e. the by the Sponsor designated person).

b. The sponsor may also decide to terminate participation of an investigator or study centre for the following reasons:

- Breach of agreement

- Insufficient patient recruitment

The sponsor will promptly notify all concerned investigators, the ethics committee(s) and the regulatory authorities of the decision to terminate the study. The sponsor will provide information regarding the timelines of study termination and instructions regarding treatment and data collection of enrolled patients.

# 11 Ethical considerations

## 11.1 Regulations statement

The study will be conducted in accordance with the ethical principles of the Declaration of Helsinki (2013), the ICH-GCP Guidelines, the EU Clinical Trial Directive (2001/20/EG), and applicable regulatory requirements. The site investigator is responsible for the proper conduct of the study at the study site.

## 11.2 Recruitment and consent

In general, patients presenting at the GGD or GP with the diagnosis under study and possibly qualifying for participation will be informed about the trial by the GP and asked if they are interested to participate. If the patient is interested the GP will inform the site investigator of the nearby participating hospital.

Specific to this study protocol, patients diagnosed with COVID-19 can be informed about the possibility to be referred to a study site by anyone. If the patient agrees, his/her treating physician (typically his GP) will contact the study team and if the study in -and exclusion criteria seem to be fulfilled, a member of the study team will call the patient to inform him/her about the study.

Written informed consent of patients is required before enrolment in the trial and before any study related procedure takes place. ICH-GCP and other applicable regulations must be followed in informing the patient and obtaining consent. It should be taken into consideration if the patient is capable of giving informed consent. If not, a legal representative may provide informed consent. Before informed consent may be obtained, the patient (and if relevant his/her legal representative) should be given ample time and opportunity to inquire about details of the trial and to decide whether or not to participate in the trial. All questions about the trial should be answered to the satisfaction of the patient. There is no set time limit for the patient to decide. The investigator should inform each patient if there is a specific reason why he/she must decide within a limited time frame, for example if

the patient is close to the <8 days since symptom onset period or if the trial is scheduled to close for enrolment.

The content of the patient information letter, informed consent form and any other written information to be provided to patients will be in compliance with ICH-GCP, GDPR and other applicable regulations and should be approved by the ethics committee in advance of use.
The patient information letter, informed consent form and any other written information to be provided to patients will be revised whenever important new information becomes available that may be relevant to the patient's consent. Any substantially revised informed consent form and written information should be approved by the ethics committee in advance of use. The patient should be informed in a timely manner if new information becomes available that might be relevant to the patient's willingness to continue participation in the trial. The communication of this information should be documented.

## 11.3 Objection by minors or incapacitated subjects

If a study person for whom a legal representative has given informed consent at any time objects to a study procedure (e.g. blood sampling), the procedure will be stopped. If this makes further study participation impossible, the patient will be withdrawn from the study.

## 11.4 Benefits and risks assessment.

A potential benefit may be a lower risk for a bad outcome (hospital admission, death) and a shorter disease course. This however, remains to be proven. No additional blood samples will be taken (apart from a small proportion of patients who provide separate consent to inclusion for immunological follow up). No additional visits are required. The risk of plasma infusion is comparable to the risk associated with blood transfusions. These include transfusion reactions, transfusion related acute lung injury (TRALI) and the transmission of as yet unknown infectious or other transmittable diseases. The precautions as taken by the Sanquin Blood Supply regarding the prevention of infectious and non-infectious complications of blood product transfusion are taken in this study. These include, matching the donor and recipient for blood group, testing for infectious agents as well as testing for irregular antibodies and when indicated HLA- and HNA-antibody testing in the donor.

## 11.5 Compensation for injury

The sponsor/investigator has a liability insurance which is in accordance with article 7 of the WMO.

The sponsor (also) has an insurance which is in accordance with the legal requirements in the Netherlands (Article 7 WMO). This insurance provides cover for damage to research subjects through injury or death caused by the study.
The insurance applies to the damage that becomes apparent during the study or within 4 years after the end of the study.

### 11.6 Incentives

Patient will not be compensated for participation in the trial.
However, patients participating in the sub-studies will receive compensation for travel expenses.

## 12 Administrative aspects and publication

### 12.1 Handling and storage of data and documents

Data and documents will be controlled and processed conform the EU General Data Protection Regulation (GDPR) and the Dutch Act on Implementation of the General Data Protection Regulation. (in Dutch: Uitvoeringswet AVG, UAVG).

#### 12.1.1 Patient confidentiality

Each patient is assigned a unique patient study number at enrolment. In trial documents the patient’s identity is coded by patient study number as assigned at enrolment. The site investigator will keep a subject enrolment and identification log that contains the key to the code, i.e. a record of the personal identification data linked to each patient study number. This record is filed at the investigational site and should only be accessed by the investigator and the supporting hospital staff, and by representatives of the sponsor or a regulatory agency for the purpose of monitoring visits or audits and inspections. Patients confidentiality will be ensured in compliance with EU regulation and the Dutch Act on Implementation of the General Data Protection Regulation.

#### 12.1.2 Filing of essential documents

Essential documents are those documents that permit evaluation of the conduct of a trial and the quality of the data produced. The essential documents may be subject to, and should be available for, audit by the sponsor’s auditor and inspection by the regulatory authority(ies)
The investigator should file all essential documents relevant to the conduct of the trial on site. The sponsor will file all essential documents relevant to the overall conduct of the trial. Essential documents should be filed in such a manner that they are protected from accidental loss and can be easily retrieved for review.

#### 12.1.3 Record retention

Essential documents should be retained for 25 years after the end of the trial. They should be destroyed after this time, unless a longer record retention period is required by site specific regulations.
Source documents (i.e. medical records) of patients should be retained for at least 15 years after the end of the trial described in section 12.5. Record retention and destruction after this time is subject to the site's guidelines regarding medical records.

#### 12.1.4 Storage and sharing of data

Electronic patient data collected in the e-CRF will be stored at the sponsor for 25 years.
The data collected by questionnaires filled in by the consenting donors will be stored for 25 years. Encoded data may be shared with other study groups for research purposes, for example with the EU COVID initiative (https://joinup.ec.europa.eu/collection/digital-response-covid-19/news/european-covid-19-data-platform). If data are sent to countries outside de EU, patients confidentiality will be ensured at an equal level of EU regulation and the Dutch Act on Implementation of the General Data Protection Regulation.

#### 12.1.5 Storage of samples

Biological samples should only be stored for the purpose of additional research if the patient has given consent. If no informed consent was obtained, samples should be destroyed after the patient has completed all protocol treatment and procedures. Sampling in participating sites is possible but only if done according to the CoV-Early standard operating procedure for this. After sampling, the samples from participating non-academic sites should be shipped and stored to a designated storage facility for biological samples from one of the academic partners within the CoV-Early group. Sample processing is done according to uniform standard operating procedures. Samples that are shipped to another facility (e.g. a central laboratory) for a purpose as described in this protocol or for additional scientific research, should be stripped from any identifying information and labeled with a code (trial name or number and patient study number as assigned at enrolment).

### 12.2 Monitoring and quality insurance

The sponsor will perform on-site monitoring visits to verify that the rights and well-being of patients are protected, the reported trial data are accurate, complete, and verifiable from source documents and the conduct of the trial is in compliance with the currently approved protocol/amendment(s), with

GCP, and with the applicable regulatory requirement(s). Monitoring visits will take place according to the study specific monitoring plan.

Direct access to source documentation (medical records) must be allowed for the purpose of verifying that the data recorded in the CRF are consistent with the original source data. The sponsor expects that during monitoring visits the relevant investigational staff will be available, the source documentation will be available, and a suitable environment will be provided for review of study-related documents.

In accordance with regulatory guidelines, audits may be carried out for this study. The investigator is required to facilitate an audit by means of a site visit.
These audits will require access to all study records, including source documents, for inspection and comparison with the CRFs. Patient privacy must, however, be respected.
Similar auditing procedures may also be conducted by agents of any regulatory body reviewing the results of this study. The investigator should immediately notify the sponsor if they have been contacted by a regulatory agency concerning an upcoming inspection.

## 12.3 Amendments

A ‘substantial amendment’ is defined as an amendment to the terms of the ethics committee application, or to the protocol or any other supporting documentation, that is likely to affect to a significant degree:

- the safety or physical or mental integrity of the patients of the trial;
- the scientific value of the trial;
- the conduct or management of the trial; or
- the quality or safety of any intervention used in the trial.

All substantial amendments will be submitted to the ethics committee.

Non-substantial amendments will not be submitted but will be recorded and filed by the sponsor.

## 12.4 Annual progress report

The sponsor will submit a summary of the progress of the trial to the accredited ethics committee once a year. Interim reports will be generated more frequently on the inclusion rate (weekly) and main outcomes/complications (quarterly) for evaluations by the project group and DSMB. The first report for the METC is sent one year after the first approval date of the trial. Subsequent reports are sent annually until end of trial. Information will be provided on the date of inclusion of the first patient,

numbers of patients included and numbers of patients that have completed the trial, serious adverse events/ serious adverse reactions, other problems, and amendments.

### 12.5 Temporary halt and (prematurely) end of trial report

The sponsor will notify the accredited ethics committee and the competent authority of the end of the trial within a period of 8 weeks. The end of the trial is defined as the last patient's last visit.

The sponsor will notify the METC immediately of a temporary halt of the study, including the reason of such an action.

In case the study is ended prematurely, the sponsor will notify the accredited ethics within 15 days, including the reasons for the premature termination.

Within one year after the end of the trial, the sponsor will submit an end of trial report with the results of the study, including any publications/abstracts of the study, to the accredited ethics committee.

### 12.6 Public disclosure and publication policy

Trial results will always be submitted for publication in a peer reviewed scientific journal regardless of the outcome of the trial – unless the trial was terminated prematurely and did not yield sufficient data for a publication. Reports will also be shared through preprint servers as soon as possible.

## 13 Structured risk analysis

### 13.1 Potential issues of concern

The only concern is the concern that exists for the use of any human blood products.
No other concerns are in place. No SAE or AE occurred with the use of convP for COVID-19 in the previous Dutch ConCOVID RCT on convP.

a. Level of knowledge about mechanism of action
See introduction paragraph of the protocol

b. Previous exposure of human beings with the test product(s) and/or products with a similar biological mechanism.
See introduction paragraph of the protocol

c. Can the primary or secondary mechanism be induced in animals and/or in ex-vivo human cell material?
Yes, animal model in hamsters

d. Selectivity of the mechanism to target tissue in animals and/or human beings
Unknown

e. Analysis of potential effect
See introduction paragraph of the protocol

f. Pharmacokinetic considerations
Unknown for SARS-Cov-2 antibodies at this time

g. Study population
SARS-CoV-2 infected patients not admitted to the hospital but at risk for disease progression

h. Interaction with other products
None

i. Predictability of effect
Not predictable at this time

j. Can effects be managed?
Transfusion reactions are managed with standard transfusion reaction management protocols

## 13.2 Synthesis

All standard blood product safety measures are in place except for the 4-month quarantine period in ConvP that is normally adhered to. The overall risk of a single allogeneic plasma transfusion is low. To reduce this risk further, we will only use plasma from male donors who have no history of blood transfusion, or female donors / donors with a history of blood transfusion before 01-01-1980 when tested negative for HLA- and HNA-antibodies. Given the estimated 20% risk of hospital admission or dead of the disease in the population under study the risk is considered acceptable.

# 14 References

1. Dexamethasone in Hospitalized Patients with Covid-19 — Preliminary Report. *New England Journal of Medicine.* 2020.
2. Hung IF-N, Lung K-C, Tso EY-K, et al. Triple combination of interferon beta-1b, lopinavir–ritonavir, and ribavirin in the treatment of patients admitted to hospital with COVID-19: an open-label, randomised, phase 2 trial. *The Lancet.* 2020;395(10238):1695-1704.
3. Beigel JH, Tomashek KM, Dodd LE, et al. Remdesivir for the Treatment of Covid-19 - Preliminary Report. *N Engl J Med.* 2020.
4. Hung IF-N, Lung K-C, Tso EY-K, et al. Triple combination of interferon beta-1b, lopinavir–ritonavir, and ribavirin in the treatment of patients admitted to hospital with COVID-19: an open-label, randomised, phase 2 trial. *The Lancet.* 2020;395(10238):1695-1704.
5. Horby P, Lim WS, Emberson J, et al. Effect of Dexamethasone in Hospitalized Patients with COVID-19: Preliminary Report. *medRxiv.* 2020:2020.2006.2022.20137273.
6. Mair-Jenkins J, Saavedra-Campos M, Baillie JK, et al. The effectiveness of convalescent plasma and hyperimmune immunoglobulin for the treatment of severe acute respiratory infections of viral etiology: a systematic review and exploratory meta-analysis. *J Infect Dis.* 2015;211(1):80-90.
7. Imai M, Iwatsuki-Horimoto K, Hatta M, et al. Syrian hamsters as a small animal model for SARS-CoV-2 infection and countermeasure development. *Proc Natl Acad Sci U S A.* 2020.
8. Shen C, Wang Z, Zhao F, et al. Treatment of 5 Critically Ill Patients With COVID-19 With Convalescent Plasma. *Jama.* 2020;323(16):1582-1589.
9. Joyner MJ, Wright RS, Fairweather D, et al. Early safety indicators of COVID-19 convalescent plasma in 5,000 patients. *J Clin Invest.* 2020.
10. Okba NMA, Müller MA, Li W, et al. Severe Acute Respiratory Syndrome Coronavirus 2-Specific Antibody Responses in Coronavirus Disease 2019 Patients. *Emerg Infect Dis.* 2020;26(7).
11. Wang X, Guo X, Xin Q, et al. Neutralizing Antibodies Responses to SARS-CoV-2 in COVID-19 Inpatients and Convalescent Patients. *Clin Infect Dis.* 2020.
12. Liu STH, Lin H-M, Baine I, et al. Convalescent plasma treatment of severe COVID-19: A matched control study. *medRxiv.* 2020:2020.2005.2020.20102236.
13. Perotti C, Baldanti F, Bruno R, et al. Mortality reduction in 46 severe Covid-19 patients treated with hyperimmune plasma. A proof of concept single arm multicenter interventional trial. *medRxiv.* 2020:2020.2005.2026.20113373.
14. Duan K, Liu B, Li C, et al. Effectiveness of convalescent plasma therapy in severe COVID-19 patients. *Proceedings of the National Academy of Sciences.* 2020;117(17):9490-9496.
15. gharbharan Aea. Convalescent Plasma for COVID-19. A randomized clinical trial. *Medxriv.* 2020.
16. Larsen MD, de Graaf EL, Sonneveld ME, et al. Afucosylated immunoglobulin G responses are a hallmark of enveloped virus infections and show an exacerbated phenotype in COVID-19. *bioRxiv.* 2020:2020.2005.2018.099507.
17. Petkova E, Antman EM, Troxel AB. Pooling Data From Individual Clinical Trials in the COVID-19 Era. *Jama.* 2020;324(6):543-545.
18. Magleby R, Westblade LF, Trzebucki A, et al. Impact of SARS-CoV-2 Viral Load on Risk of Intubation and Mortality Among Hospitalized Patients with Coronavirus Disease 2019. *Clin Infect Dis.* 2020.
19. Petrilli CM, Jones SA, Yang J, et al. Factors associated with hospital admission and critical illness among 5279 people with coronavirus disease 2019 in New York City: prospective cohort study. *BMJ.* 2020;369:m1966.
20. Wang G, Wu C, Zhang Q, et al. C-Reactive Protein Level May Predict the Risk of COVID-19 Aggravation. *Open Forum Infect Dis.* 2020;7(5):ofaa153.

21. Williamson EJ, Walker AJ, Bhaskaran K, et al. OpenSAFELY: factors associated with COVID-19 death in 17 million patients. *Nature.* 2020.
22. Vogelzang EH, Loeff FC, Derksen NIL, et al. Development of a SARS-CoV-2 total antibody assay and the dynamics of antibody response over time in hospitalized and non-hospitalized patients with COVID-19. *medRxiv.* 2020:2020.2006.2017.20133793.
23. Oja AE, Saris A, Ghandour CA, et al. Divergent SARS-CoV-2-specific T and B cell responses in severe but not mild COVID-19. *bioRxiv.* 2020:2020.2006.2018.159202.
24. Ferretti AP, Kula T, Wang Y, et al. COVID-19 Patients Form Memory CD8+ T Cells that Recognize a Small Set of Shared Immunodominant Epitopes in SARS-CoV-2. *medRxiv.* 2020:2020.2007.2024.20161653.
25. Brouwer PJM, Caniels TG, van der Straten K, et al. Potent neutralizing antibodies from COVID-19 patients define multiple targets of vulnerability. *Science.* 2020;369(6504):643.
26. Bi Q, Wu Y, Mei S, et al. Epidemiology and transmission of COVID-19 in 391 cases and 1286 of their close contacts in Shenzhen, China: a retrospective cohort study. *Lancet Infect Dis.* 2020.
27. Coll E, Fernández-Ruiz M, Sánchez-Álvarez JE, et al. COVID-19 in transplant recipients: The Spanish experience. *Am J Transplant.* 2020.
28. Hoek RAS, Manintveld OC, Betjes MGH, et al. COVID-19 in solid organ transplant recipients: a single-center experience. *Transpl Int.* 2020.
29. Hueso T, Pouderoux C, Péré H, et al. Convalescent plasma therapy for B-cell-depleted patients with protracted COVID-19. *Blood.* 2020;136(20):2290-2295.

## A. Risk factors for severe COVID-19

**Age 18-49:**

Patients age 18-49 years with an inherited or acquired immunodeficiency as defined in table 2 are at increased risk for a complicated COVID19 disease course and are eligible for the study.

Table 2

| |
|---|
| Lymfopenia (<0.5 × $10^9$ neutrophils/L [<500 neutrophils/$mm^3$]) at the last lymphocyte count that was done preceding the COVID19<br>diagnosis and is present at the time of COVID19 diagnosis as well |
| History of an allogeneic stem cell transplant |
| History of a solid organ transplant |
| Use of corticosteroids at a therapeutic dose of ≥0.3 mg/kg corticosteroids at the time of COVID19 diagnosis |
| Treatment with T-cell immunosuppressants (e.g. calcineurin inhibitors, TNFa blockers, T-cell depleting monoclonal antibodies) |
| Treatment with recognized B-cell immunosuppressants, such as Bruton's tyrosine kinase inhibitors, eg, ibrutinib<br>Treatment with B-cell depleting agents, such as but not limited to rituximab, blinatumomab. |
| Inherited or acquired severe B-cell dysfunction leading to documenten hypo -or agammaglobulinemia<br>Inherited or acquired severe T-cell dysfunction |

**Age 50-69:**

For patients age 50-69, one of the following risk factors has to be present. This can be according to the medical history of the patient or a lab-based risk factor (if available)

**1. A/ Medical history**

- ♦ Obesity with BMI 35 or higher
- ♦ Born as a male person
- ♦ History of cardiac or pulmonary disease (e.g. but not limited to atrial fibrillation, coronary artery disease, heart failure, COPD, asthma)
- ♦ History of neurological disease (e.g. a history of stroke or any other chronic debilitating neurological disease)
- ♦ Diabetes for which medical therapy is needed
- ♦ Chronic kidney disease with GFR <60 ml/min
- ♦ Reumatic disease (e.g. reumatoid arthritis, Systemic lupus erythematosus, psoriatric artritis)

- Immunodeficiency (e.g. organ or allogeneic transplantation, systemic immunosuppressive drugs)
- Cancer not in complete remission for >1 year (excluding baso -or spinocellular skin cancers)
- Untreated HIV and CD4 T-cells <200/microliter
- Chronic liver disease being liver cirrhosis child pugh A/B/C or other disease leading to liver dysfunction

**1. B/ Lab results (if available)**

- CRP > 30
- SARS-CoV-2 RT-PCR Ct value <25

**Age 70 or older:**

Patients aged 70 or older are at sufficient risk and can be included regardless of medical history or lab results as long as they fulfill all other inclusion criteria.

## B. RIVM data

An example of data from the early epidemic in the Netherlands. We also used more recent data that RIVM provided to base our power and sample size calculations on.

### 4 Leeftijdsverdeling en man-vrouwverdeling van COVID-19 patiënten vanaf 4 mei 2020

Tabel 3: Leeftijdsverdeling van bij de GGD'en gemelde COVID-19 patiënten, van in het ziekenhuis opgenomen COVID-19 patiënten en van overleden COVID-19 patiënten vanaf 4 mei 2020[1,2]

| Leeftijdsgroep | Totaal gemeld | % | Ziekenhuisopname | % | Overleden | % |
|---|---|---|---|---|---|---|
| Totaal gemeld | 9803 | | 452 | | 807 | |
| 0-4 | 83 | 0.8 | 7 | 1.5 | 0 | 0.0 |
| 5-9 | 94 | 1.0 | 0 | 0.0 | 0 | 0.0 |
| 10-14 | 178 | 1.8 | 1 | 0.2 | 0 | 0.0 |
| 15-19 | 344 | 3.5 | 4 | 0.9 | 0 | 0.0 |
| 20-24 | 735 | 7.5 | 10 | 2.2 | 0 | 0.0 |
| 25-29 | 866 | 8.8 | 11 | 2.4 | 0 | 0.0 |
| 30-34 | 768 | 7.8 | 10 | 2.2 | 1 | 0.1 |
| 35-39 | 623 | 6.4 | 11 | 2.4 | 1 | 0.1 |
| 40-44 | 650 | 6.6 | 14 | 3.1 | 2 | 0.2 |
| 45-49 | 745 | 7.6 | 33 | 7.3 | 6 | 0.7 |
| 50-54 | 845 | 8.6 | 43 | 9.5 | 9 | 1.1 |
| 55-59 | 842 | 8.6 | 62 | 13.7 | 14 | 1.7 |
| 60-64 | 635 | 6.5 | 46 | 10.2 | 18 | 2.2 |
| 65-69 | 312 | 3.2 | 40 | 8.8 | 36 | 4.5 |
| 70-74 | 292 | 3.0 | 35 | 7.7 | 69 | 8.6 |
| 75-79 | 332 | 3.4 | 38 | 8.4 | 88 | 10.9 |
| 80-84 | 441 | 4.5 | 41 | 9.1 | 148 | 18.3 |
| 85-89 | 545 | 5.6 | 30 | 6.6 | 205 | 25.4 |
| 90-94 | 360 | 3.7 | 15 | 3.3 | 154 | 19.1 |
| 95+ | 112 | 1.1 | 1 | 0.2 | 56 | 6.9 |
| Niet vermeld | 1 | 0.0 | 0 | 0.0 | 0 | 0.0 |

[1] Sinds 1 juni kan iedereen zich met klachten laten testen. Toch is het aannemelijk dat niet alle COVID-19 patiënten getest worden. De werkelijke aantallen in Nederland zijn daarom waarschijnlijk hoger dan de aantallen die hier genoemd worden. Het werkelijke aantal COVID-19 patiënten opgenomen in het ziekenhuis of overleden is hoger dan het aantal opgenomen of overleden patiënten gemeld in de surveillance, omdat de surveillance gebaseerd is op de informatie op het moment van melding. Ziekenhuisopname na melding is niet altijd bekend. Aan het RIVM wordt niet gemeld wie hersteld is.

[2] De leeftijd van de gemelde patiënten is gemiddeld lager dan de leeftijd van de in het ziekenhuis opgenomen of overleden patiënten. Dit is een weergave van het testbeleid.

## C. Labeling of plasma

### Convalescent plasma

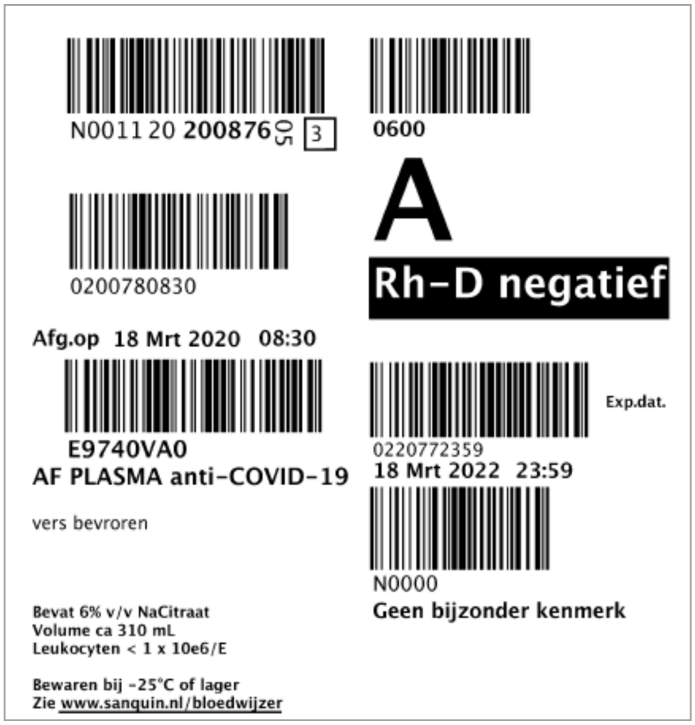

## D. Common Terminology Criteria for Adverse Events

The grading of adverse events will be done using the NCI Common Terminology Criteria for Adverse Events, CTCAE version *5.0*

## E. Donor criteria used by Sanquin

Additional donor criteria specific for COVID-19 convalescent plasma donors:
History of COVID-19 disease comfirmed by SARS-COV-2 PCR and asymptomatic for at least 14 days

**Standard plasma donor criteria**

**Inclusion criteria**

- Known ABO-Resus(D) blood group
- A screening for irregular antibodies with a titer ≤ 1:32
- Written informed consent regarding the plasmapheresis procedure
- Tested negative for HIV, HBV, HCV, HEV, HTLV and syfilis

**Exclusion criteria**

- Age <18 years or age >65 years (80 in donors who were already registered as a donor at Sanquin before the age of 65 years)
- Weight <50kg
- Medical history of heart failure
- History of transfusion with red blood cells, platelets or plasma after 01-01-1980
- History of organ- or tissue transplant
- A cumulative stay in the United Kingdom of ≥ 6 months in the period between 01-01-1980 and 31-12-1996
- A history of i.v. drug use
- Insulin dependant diabetes
- An underlying severe chronic illness (i.e. history of heart failure, cancer or stroke)
- Tested positive for HLA- or HNA-antibodies

## F. Hemovigilance

**Post treatment assessments - Adverse event assessment of plasma infusion:** Concerning the occurrence of possible plasma infusion related Adverse Events AE, SAE, AR, SUSAR will be actively screened for by the treating physician and the institutional haemovigilance employee(s), the latter is adviced to be a member of each institutional study team. .

**Specific organization of the plasma haemoviglance**

Most of transfusion related side-effects are respiratory symptoms [82]. Notwithstanding the additional importance of also other symptoms, it is especially important to carefull monitor all new or changing respiratory symptoms. Only then, it will become possible to determine if such symptoms are more likely caused by the course of COVID-19 or by the plasma transfusion.

The question of causality or imputability of (S)AEs occurring after plasma transfusion, in this respect can only be answered by an aggregate of much more information that in case of such an (S)AE needs to be collected. Examples of such information are the patient's medical history, his/her previous transfusion history, vital signs before, during and after transfusion of the plasma, chest examination and imaging (before and after an expected transfusion reaction), but also the details of non-blood fluids given the fluid balance chart, details of co-medication and interventional medication given (including diuretics) and the response to measures and recovery of symptoms. Additionally, each transfusion reaction is followed by blood tests, imaging etc. according to national haemovigilance guidelines and are subsequently categorized, graded for severeity and imputability. All transfusion related reactions – irrespective of potential causality or not- in our study patients will be recorded and analysed and reported according to national blood transfusion and haemovigilance guidelines which are standard and operational in every Dutch hospital. This is done by the institutional haemovigilance employee of each hospital as they do for all blood products administered in Dutch hospitals.

Some characteristics and grades of known transfusion related side effects are:

**Allergic/Anaphylactic reaction:**
Mild, Transient flushing, urticaria or rash.
Moderate Wheeze or angioedema with or without flushing/urticaria/rash but without respiratory compromise or hypotension.
Severe Bronchospasm, stridor, angioedema or circulatory problems which require urgent medical intervention AND/OR, directly result in or prolong hospital stay, or anaphylaxis (severe, life-

threatening, generalised or systemic hypersensitivity reaction with rapidly developing airway and/or breathing and/or circulation problems, usually associated with skin and and mucosal changes)

**Transfusion-related acute lung injury (TRALI)**
acute dyspnoea with hypoxia and bilateral pulmonary infiltrates during or within 6 hours of transfusion, in the absence of circulatory overload or other likely causes, or in the presence of human leucocyte antigen (HLA) or human neutrophil antigen (HNA) antibodies cognate with the recipient.

**Transfusion-associated circulatory overload (TACO)**
Should exhibit at least one required criterion* with onset during or up to 12 hours after transfusion, and a total of 3 or more criteria i.e. *A and/or B, and total of at least 3 (A to E)

* Required criteria (A and/or B)

A. Acute or worsening respiratory compromise and/or

B. Evidence of acute or worsening pulmonary oedema based on:

• clinical physical examination, and/or

• radiographic chest imaging and/or other non-invasive assessment of cardiac function

Additional criteria

C. Evidence for cardiovascular system changes not explained by the patient’s underlying medical condition, including development of tachycardia, hypertension, jugular venous distension, enlarged cardiac silhouette and/or peripheral oedema

D. Evidence of fluid overload including any of the following: a positive fluid balance; clinical improvement
following diuresis

E. Supportive result of a relevant biomarker, e.g. an increase in N-terminal-pro brain natriuretic peptide (NT-pro BNP) to greater than 1.5 times the pre-transfusion value

**Transfusion-Associated Dyspnoea or TAD** is characterised by respiratory distress within 24 hours of transfusion that does not meet the criteria for transfusion-related acute lung injury (TRALI) or transfusion-associated circulatory overload (TACO) or allergic reaction. Respiratory distress in such cases should not be adequately explained by the patient’s underlying condition.

# Summary of changes in Protocol in the approved amendements

**October 10, 2021**

| Section | First IRB approved version of the protocol (Version 2.0, 22nd Sep 2020) | List of all changes up to the last amendement nr. 4 (protocol Version 6.0, 10th Jun 2021) |
|---|---|---|
| Responsibilities, Names and Affiliations of the investigators and/or collaborators | | Rob J.C.G. Verdonschot Erasmus MC, Henrieke Prins Erasmus MC, Hannelore Gotz GGD Regio Rijnmond, Drs. Lotte Rokx-Niemantsverdriet, Drs. Jelle Struik GCML Huisartsen centrum/HAGRO Rotterdam centrum have been added as collaborators and/or investigators |
| Study sites and PIs | The following sites were registered as potential participating sites:<br>1. Erasmus MC – Bart Rijnders/Casper Rokx<br>2. LUMC Jaap Jan Zwaginga<br>3. Martini Groningen – Imro Vlasveld<br>4. Reinier de Graaf – Eduardus Posthuma<br>5. Haaglanden MC – Femke Mollema<br>6. Rijnstate Arnhem – Robert-Jan Hassing<br>7. Catherina Ziekenhuis Eindhoven – Heidi Ammerlaan<br>8. OLVG – Janneke Stalenhoef<br>9. Maasstadziekenhuis – Jan den Hollander<br>10. Groene Hart Ziekenhuis – Faiz Karim<br>11. Spaarne Gasthuis – Robin Soetekouw<br>12. Antonius Nieuwegein – Elena Monica van Leeuwen-Sergarceanu<br>13. Amphia Ziekenhuis Breda, Ronald van Etten<br>14. Medisch centrum Leeuwarden, Linda Kampschreur<br>15. Ziekenhuis Bernhoven, Inge Ludwig<br>16. Meander Medisch Centrum, Eefje Jong<br>17. UMCG, Douwe Postma | The following sites were registered as the final list of participating sites.<br>1. Erasmus MC – Bart Rijnders/Casper Rokx<br>2. LUMC Jaap Jan Zwaginga<br>3. Maasstadziekenhuis – Jan den Hollander<br>4. Groene Hart Ziekenhuis – Faiz Karim<br>5. Spaarne Gasthuis – Robin Soetekouw<br>6. Antonius Nieuwegein – Elena Monica van Leeuwen-Sergarceanu<br>7. Amphia Ziekenhuis Breda, Ronald van Etten<br>8. Medisch centrum Leeuwarden, Linda Kampschreur<br>9. UMCG, Douwe Postma<br>10. Isala Zwolle, Jolanda Lammers<br>11. Hagaziekenhuis, Jean Louis, Kerkhoffs<br>12. OLVG, Janneke Stalenhoef |
| 2.2 Secondary objectives | | Added the immunocompromised state of the patient as one of the factors for determining the impact of convalescent plasma. |

| | | |
|---|---|---|
| | | Added the objective: To evaluate the impact of convP on the primary outcome in patients with and without neutralizing antibodies at baseline<br><br>Added the objective: To evaluate the effect of ConvP therapy on the medium-term (3 months) immunity against SARS-CoV-2 |
| 4.1 Population base | Eligible patients are adults 50 years of age or older with a high risk of disease progressions | Eligible patients are adults 18 years of age or older with a high risk of disease progressions |
| 4.2 Inclusion criteria | RT-PCR confirmed COVID-19 | COVID-19 confirmed by PCR or CE-marked antigen test |
| | 70 years or older OR 50-69 years and 1 or more of the risk factors described in Appendix A | 70 years or older OR 50-69 years and 1 or more of the risk factors described in Appendix A<br><br>OR 18-49 and severely immunocompromised as described in Appendix A |
| 4.3 Exclusion criteria | Symptomatic for 8 days or more | Exclusion criterium added: *COVID-19 related symptoms are already improving*<br><br>Specification about one of the exclusion criteria was added: *Symptomatic for 8 days or more at the time of screening. Please note that on the day the patient is randomized he/she is allowed to be on day 8 of symptoms* |
| 6 Plasma retrieval, administration and determination of SARS-CoV-2 antibodies | Before delivery of the anti-COVID-19 plasma to the participating sites by Sanquin Blood Supply an informed consent regarding a magisterial blood product ('Bewustzijnsverklaring magistraal bloedproduct') will be signed by the ordering physician. | This section was removed in the following protocols because it was no longer required per local regulations |
| 8.1.2 Secondary endpoints | Age and clinical frailty score stratified analysis of number (%) of primary endpoint following transfusion of convP versus FFP. | This was changed into: Age and clinical frailty score and immunocompromised state stratified analysis of number (%) of primary endpoint following transfusion of convP versus FFP. |
| 8.1.3 Exploratory endpoints | | Added the following endpoint: The effect of ConvP therapy on the medium-term (3 months) immunity against SARS-CoV-2 |
| 8.2.3 Unblinding procedure | Emergency unblinding can be done by the site using the Emergency Unblinding form in the eCRF database. | The unblinding procedure has been changed into: Emergency unblinding can be done by the sponsor so please contact the sponsor if this would be needed. |
| 8.3.1 Time of Clinical evalution | | The following optional evalution was added: Serum sample |

| | | |
|---|---|---|
| | | collection on month 3, 6 and 12 of the study |
| | An optional evaluation of long-term lung damage with a lung function test at week 6, month 3 and a low-dose CT on month 3 and a questionnaire on day 28, week 6, month 3 | This was changed into: a lung function test, low dose CT-scan and a questionnaire on month 3 |
| Required investigations table | | Added month 3, months 6 and month 12 as optional blood collection moments.<br>Removed day 28 as an evaluation moment. |
| 8.3.3 Specifications of required investigations | | Added as an investigation: How was the patient referred for the study |
| | | Specified in the Spirometry that the FEV1, FVC and DLCOc should be investigated |
| 9.2.2 Serious adverse events | SAEs will be reported from the moment of plasma infusion until 60 days following infusion | SAEs will be reported from the moment of plasma infusion until 30 days following infusion (which is the end of patient follow-up) |
| 9.5 Data Safety Monitoring Board | | Added this section for missing data: If patients are lost to follow-up before day 28, the highest score that was measured before the patient was lost to follow-up will be used for the analysis of the day 28 5-point ordinal scale score (highest score observed carried forward). |
| Section A: Risk factors | | Specified the definition of immunodeficiency that should be used to classify a patient as severely immunocompromised |

# Summary of changes in the SAP in the approved amendments

**October 10, 2021**

**Paragraph 1.2**

The intention-to-treat population was described in more detail by adding this; *"Occasionally several hours may pass between randomization and the time that plasma becomes ready for transfusion on the ward. Indeed, an intravascular access needs to be placed and blood group matching of plasma and patient takes time. Also, COVID-19 occasionally has a rapidly progressive disease course in the population under study. Therefore, sporadically patients may be randomized but will not receive the plasma transfusion because upon clinical reevaluation the local investigator may decide that hospital admission has become indicated. These randomized but untreated patients will not be included in the intention to treat population."*

Immunocompromised state Y/N was added as a covariate in the model

**Paragraph 1.3**

Immunocompromised state Y/N was added in secondary endpoint 5 analysis

**Paragraph 1.4**

The height of the neutralizing antibody titers in the plasma that was transfused was added as an exploratory endpoint

The effect of ConvP therapy on the medium-term (3 months) immunity against SARS-CoV-2 was added as an exploratory endpoint.
