# Supplementary material for: Prospective individual patient data meta-analysis of two randomized trials on convalescent plasma for COVID-19 outpatients
Source: Nat Commun. 2022 May 11;13:2583. doi: 10.1038/s41467-022-29911-3 (PMC9095637; doi:10.1038/s41467-022-29911-3)
Supplement: Supplementary file 3 — Reporting Summary [file 41467_2022_29911_MOESM3_ESM.pdf]

nature portfolio

Corresponding author(s): Bart Rijnders

Last updated by author(s): Bart Rijnders 14-03-2022

Reporting Summary

Nature Portfolio wishes to improve the reproducibility of the work that we publish. This form provides structure for consistency and transparency in reporting. For further information on Nature Portfolio policies, see our Editorial Policies and the Editorial Policy Checklist.

Statistics

For all statistical analyses, confirm that the following items are present in the figure legend, table legend, main text, or Methods section.

n/a	Confirmed	
☐	☒	The exact sample size (*n*) for each experimental group/condition, given as a discrete number and unit of measurement
☐	☒	A statement on whether measurements were taken from distinct samples or whether the same sample was measured repeatedly
☐	☒	The statistical test(s) used AND whether they are one- or two-sided<br>*Only common tests should be described solely by name; describe more complex techniques in the Methods section.*
☐	☒	A description of all covariates tested
☐	☒	A description of any assumptions or corrections, such as tests of normality and adjustment for multiple comparisons
☐	☒	A full description of the statistical parameters including central tendency (e.g. means) or other basic estimates (e.g. regression coefficient) AND variation (e.g. standard deviation) or associated estimates of uncertainty (e.g. confidence intervals)
☐	☒	For null hypothesis testing, the test statistic (e.g. *F*, *t*, *r*) with confidence intervals, effect sizes, degrees of freedom and *P* value noted<br>*Give P values as exact values whenever suitable.*
☐	☒	For Bayesian analysis, information on the choice of priors and Markov chain Monte Carlo settings
☒	☐	For hierarchical and complex designs, identification of the appropriate level for tests and full reporting of outcomes
☒	☐	Estimates of effect sizes (e.g. Cohen's *d*, Pearson's *r*), indicating how they were calculated

Our web collection on statistics for biologists contains articles on many of the points above.

Software and code

Policy information about availability of computer code

Data collection	ENNOV Clinical version 8.2.20 and ALEA Clinical version 18.1
Data analysis	Prism 9.0.2 (GraphPad Software, USA)<br><br>R version 4.1.2.<br>The set seed and libraries used:<br><br>set.seed(1456)<br><br>library(readxl)<br>library(haven)<br>library(dplyr)<br>library(ggplot2)<br>library(cowplot)<br>library(tableone)<br>library(summarytools)<br>library(labelled)<br>library(kableExtra)<br>library(randomForest)<br>library(brms)<br>library(tsibble)<br>library(stringr)

library(MASS)

For manuscripts utilizing custom algorithms or software that are central to the research but not yet described in published literature, software must be made available to editors and reviewers. We strongly encourage code deposition in a community repository (e.g. GitHub). See the Nature Portfolio guidelines for submitting code & software for further information.

Data

Policy information about availability of data

All manuscripts must include a data availability statement. This statement should provide the following information, where applicable:

- Accession codes, unique identifiers, or web links for publicly available datasets
- A description of any restrictions on data availability
- For clinical datasets or third party data, please ensure that the statement adheres to our policy

The source data and generated data are available in the supplementary data files.

Field-specific reporting

Please select the one below that is the best fit for your research. If you are not sure, read the appropriate sections before making your selection.

☒ Life sciences ☐ Behavioural & social sciences ☐ Ecological, evolutionary & environmental sciences

For a reference copy of the document with all sections, see nature.com/documents/nr-reporting-summary-flat.pdf

Life sciences study design

All studies must disclose on these points even when the disclosure is negative.

Sample size	n=779. The study had a bayesian design with no predefined absolute sample size. Rather, the recruitment was planned to continue untill any of the following would occur:<br>1. A stopping rule had been reached and the DSMB would recommend to stop the trial<br>2. When all participating trials in the COMPILEhome initiative would have completed their enrollment<br><br>However, in reality the recruitment was stopped at the time when vaccination uptake had become 80% in both countries (which hampered further recruitment) as well as the occurence of the delta variant. This is described in the full paper and suppl data in more detail.
Data exclusions	12 subjects did not receive allocated treatment and were therefore excluded as per study protocol. 3 were excluded as their age was <50 as per protocol
Replication	The method section describes the selection of patients and the selection of convalescent plasma products. Since this is a prospective intervention trial we cannot replicate the results.
Randomization	Patients were randomized 1:1 to convalescent plasma or placebo.
Blinding	The investigators and patients were blinded to the treatment allocation.

Reporting for specific materials, systems and methods

We require information from authors about some types of materials, experimental systems and methods used in many studies. Here, indicate whether each material, system or method listed is relevant to your study. If you are not sure if a list item applies to your research, read the appropriate section before selecting a response.

Materials & experimental systems

n/a	Involved in the study	
☐	☒	Antibodies
☒	☐	Eukaryotic cell lines
☒	☐	Palaeontology and archaeology
☒	☐	Animals and other organisms
☐	☒	Human research participants
☐	☒	Clinical data
☒	☐	Dual use research of concern

Methods

n/a	Involved in the study	
☒	☐	ChIP-seq
☒	☐	Flow cytometry
☒	☐	MRI-based neuroimaging

Antibodies

Antibodies used	Antibodies present in convalescent plasma from recovered COVID-19 patients
Validation	Virus neutralization activity of antibodies was tested using different neutralizing assays. As both study labs used a different SARS-CoV-2 neutralizing antibody test, a panel of 15 plasma samples was provided for comparison by the Support-E consortium. This panel included a research reagent 20/130 obtained from the National Institute for Biological Standards and Control (NIBSC, United Kingdom), which had been assigned a unitage of 1,300 international units (IU)/mL of SARS-CoV-2-neutralising antibodies. A further dilution series of a high-titre convalescent plasma sample (initial neutralising antibody titre of 1:5120 provided as neat, and diluted in 1:10, 1:50 and 1:100) was calibrated in IU/mL against this research reagent, and used to assess the linearity of both assays. This allowed retrospective conversion of neutralizing antibody titers into international units (IU/mL) using linear regression formulae derived from assay calibration as shown below for each trials.<br><br>For COnV-ert study: Experimental neutralization titers were converted to IU/mL using the following regression formula (IU/mL = 4160/(2^(Log2(experimentalID50-13.962)/-0.9798) derived from assay calibration with the pre-quantified control.<br><br>For CoV-Early study: Experimental neutralization titers were converted to IU/mL using the following regression formula (IU/mL = 4160/(2^(Log2(experimentalID50-11.832)/-1.146) derived from assay calibration with the pre-quantified control.

Human research participants

Policy information about studies involving human research participants

Population characteristics	1) Participant of a trial that joined COMPILEhome, 2) Patients with confirmed COVID-19 using diagnostic PCR or antigen test, 3) Neither hospitalized or at the emergency room department of a hospital, 4) symptomatic with illness onset ≤ 7 days and 5) 50 years or older<br><br>Median age of partipants (IQR): 58 (53-64)<br>Male sex in percentage: 66.8%
Recruitment	Trials included had to be approved by the respective institutional review boards and competent authorities of the countries involved. All patients gave informed consent.<br><br>For COnV-ert: Study candidates were identified from two sources: (1) we actively screened the healthcare records of study sites for individuals with evidence of SARS-CoV-2 infection and (2) individuals who tested positive for SARS-CoV-2 infection during epidemiological surveillance could voluntarily register to an institutional website launched by the sponsor and the Catalan Institute of Health.<br><br>For CoV-Early: Outpatients diagnosed with COVID-19 by PCR or antigen testing and symptomatic for <8 days could be screened. Unless they were severely immunocompromised, they had to be at least 50 years old and have at least one risk factor associated with a higher risk of severe COVID-19. Further details can be found in the full protocol available as an online supplement.<br><br>The study was communicated with the Dutch public using all kinds of media including newspapers, medical journals for general practitioners, public health free-of-charge COVID test centers as well as social media. Patients aged 50 or older that tested positive for SARS-CoV-2 at a public health SARS-CoV-2 test centers were contacted by telephone about the positive result of their test by the test center and informed about the possibility of study participation at a nearby hospital. When they showed interest and agreed to be contacted by the study team, their telephone number was shared with the study team and the patient was contacted to get additional information. When the patient fulfilled the in- and exclusion criteria and wanted to participate, he/she received an appointment at the nearest study site the next day. Self-referral was possible as well via www.cov-early.nl or www.coronaplasmastudie.nl
Ethics oversight	Erasmus MC institutional review board

Note that full information on the approval of the study protocol must also be provided in the manuscript.

Clinical data

Policy information about clinical studies

All manuscripts should comply with the ICMJEguidelines for publication of clinical research and a completedCONSORT checklist must be included with all submissions.

Clinical trial registration	NCT04621123 and NCT04589949
Study protocol	https://clinicaltrials.gov/ct2/show/NCT04589949 and https://clinicaltrials.gov/ct2/show/NCT04621123
Data collection	Recruitment was between November 2020 and July 2021 at study sites in Spain and the Netherlands. Data collection was performed realtime on day 7, day 14 and day 28 after inclusion for both studies
Outcomes	The first primary endpoint incorporated the speed of recovery and the progression of COVID-19 that would lead to hospital or intensive care unit (ICU) admission or death. It was defined as the highest score on a 5-point ordinal disease severity

scale in the 28 days after randomization. Secondary endpoints were time to full symptom resolution (assessed by the blinded study team during a telephone contact on day 7, day 14, and day 28) and the safety of CP in outpatients with COVID-19.

The first primary endpoint was analyzed with a Bayesian proportional odds model with normally distributed priors. The second primary endpoint was analyzed with a Bayesian logistic model with a similar specification.
